# Knowledge-enhanced large language model construction for garlic cultivation using GraphRAG

**DOI:** 10.3389/fpls.2026.1912355

**Published:** 2026-08-18

**Authors:** Kai Zhou, Mengyao Dong, Xinkai Meng, Jiacheng Lv, Xiaojun Long, Rui Hou, Yuhua Li, Jialin Hou

**Affiliations:** 1 College of Mechanical and Electronic Engineering, Shandong Agricultural University, Tai’an, China; 2 School of Artificial Intelligence, Beijing University of Posts and Telecommunications, Beijing, China

**Keywords:** garlic cultivation, GraphRAG, knowledge graph, LLMs, prompt optimization

## Abstract

**Introduction:**

Rapid advances in smart agriculture have highlighted the potential of large language models (LLMs), while practical applications remain limited by privacy risks, high training costs, and hallucinations. Focusing on garlic cultivation, this study proposes a knowledge-graph-enhanced framework that improves domain-specific LLMs through graph-based retrieval.

**Methods:**

A hybrid clustering algorithm with intra-cluster multidimensional ranking is employed to identify ten core entity types from garlic-related corpora. Few-shot learning and chain-of-thought prompting are further integrated to optimize entity and relation extraction using Qwen 2.5:32B, leading to the construction of a structured garlic cultivation knowledge graph. The knowledge graph is integrated with LLMs via GraphRAG, while prompt reconstruction strategies are adopted to enrich contextual information and constrain generation, thereby improving domain grounding and reducing hallucinations.

**Results:**

Experimental results demonstrate absolute improvements of 13.0, 37.0, and 28.2 percentage points in precision, recall, and F1-score, respectively, for triple extraction compared with the baseline LLM, along with a 37.5 percentage point increase in retrieval-based question-answering accuracy.

**Discussion:**

The proposed approach is interpretable and locally deployable without dependence on commercial APIs, offering a transferable methodological reference for domain-specific knowledge graph construction and intelligent question answering across diverse crop systems.

## 1 Introduction

China is the world’s largest producer and exporter of garlic with a cultivation history spanning more than two millennia (Luan et al., 2019). Garlic serves not only as a crucial economic crop but also as a primary source of income for numerous rural households. While recent studies have begun applying AI to garlic-related tasks such as post-harvest damage detection (Gao et al., 2026), research addressing the full cultivation process — including knowledge organization, pest and disease management, and agronomic decision support — remains scarce, underscoring the need for domain-specific intelligent systems targeting the garlic cultivation domain. In recent years, alongside the rapid advancement of agricultural modernisation, the demographic structure of garlic cultivators has undergone a significant transition. It has shifted from a traditional reliance on experience-driven, multi-generational smallholder farmers towards a diversified landscape comprising emerging agricultural entities such as farmers’ cooperatives, family farms, and large-scale agribusinesses. However, this diversification and scaling of operational entities have not organically bridged the profound ‘knowledge gap’ within the sector. On the one hand, traditional smallholder farmers lack the underpinning of modern agricultural scientific theory. Consequently, when confronted with a complex and volatile environment—characterised by increasingly frequent extreme weather events and exacerbated mutations in pests and diseases—their production decisions tend to be highly arbitrary and heavily reliant on historical experience. On the other hand, although novel agricultural entities possess advantages in capital and scale and have integrated modern facilities alongside contemporary management philosophies, they generally lack crop-specific, in-depth agronomic knowledge regarding garlic. This deficiency hinders the realisation of genuinely refined and precision-driven field management. This severe mismatch between the provision of professional knowledge and practical production demands has resulted in the garlic industry facing persistent challenges. These include the accelerated degeneration of cultivars, low adoption rates of standardised cultivation techniques, and poor efficacy in integrated pest and disease management. Concurrently, agricultural knowledge remains highly fragmented across academic research papers, technical manuals, farmers’ production logs, and government reports, lacking a unified and structured organisational framework. Ultimately, these compounding issues constrain the widespread adoption of intelligent agricultural systems and impede the high-quality development of the garlic industry.

Traditionally, the organization and utilization of agricultural knowledge have relied on expert experience, technical manuals, and rule-based expert systems. These approaches provided valuable guidance in the early stages but became increasingly limited due to slow knowledge updates, restricted coverage, and limited capacity to address complex and dynamic cultivation environments. At the same time, the fragmented and unstructured nature of agricultural knowledge has hindered efficient information retrieval and system integration, making it difficult to meet the demands of intelligent decision-making. Against this backdrop, the breakthroughs achieved by large language models (LLMs) in various natural language processing tasks (Raiaan et al., 2024) have opened new possibilities for agricultural applications. Their potential applications in agriculture are increasingly recognized, particularly in providing accessible, interactive guidance for crop management. Vizniuk et al. (2025) analyzed the integration of LLMs into agricultural decision-support systems, highlighting their potential to optimize production processes. Shaikh et al. (2025) evaluated generative AI in smart agriculture, establishing performance benchmarks and research directions. Jiang J. et al. (2025) proposed a knowledge-guided agricultural LLM for decision-making, offering insights into domain adaptation. Kuska et al. (2024) discussed integrating LLMs with digital agricultural technologies and emphasized their potential in crop protection and consulting. Zhu et al. (2025b) developed a multimodal LLM for potato disease detection, while Zhang et al. (2025) proposed an agricultural LLM for pests and diseases management based on contextual reasoning. Together, these studies illustrate the growing promise of LLMs in agricultural applications, a trend comprehensively synthesized by Zhu et al. (2025a) in their review of large vision and language models across agricultural domains.

Despite their potential, LLMs face persistent challenges when applied to professional agricultural corpora. Two issues are particularly prominent: (1) hallucinations—outputs that lack factual grounding; and (2) limited domain adaptability, including insufficient understanding of agricultural terminology and weak ability to model cross-sentence relationships. Fisher (2024) noted that unconstrained LLM generation often produces fabricated content, though this problem can be mitigated when models are grounded in externally retrieved knowledge. These limitations underscore the importance of combining LLMs with structured domain knowledge.

The knowledge graph (KG) initially referred specifically to the knowledge base built by Google to support its semantic search capabilities. A KG is a graph-based data structure composed of nodes and edges, where each node represents an entity and the edges denote relationships between entities. As a structured semantic knowledge base, a KG represents entities, concepts, relations, and attributes in a graphical form, revealing rich interconnections and deeper meanings within data, thereby providing interpretable and fact-consistent representations of domain knowledge (Yang Z. et al., 2025; Li et al., 2025). By encoding real-world knowledge into triples, KGs have become a core component of many AI applications. In agriculture, early KGs were primarily expert-curated and exhibited limited scope. With advances in semantic technologies, ontology-driven and information extraction-based methods have been developed. Jiang T. et al. (2025) built a pig disease KG to improve knowledge management, Zhao et al. (2024) designed a jujube disease detection system integrating KGs with machine learning, Veena et al. (2023) explored semi-supervised triple extraction for agricultural texts, Wang and Zhao (2024) applied LLMs to entity and relation identification, Wang et al. (2024) constructed a knowledge graph for tomato leaf pests and diseases using the ALBERT-BiLSTM-CRF named entity recognition model, achieving a recall rate of 95.03% and enabling digital diagnosis through Neo4j-based visualization, and Lv et al. (2024) constructed multimodal KGs for cabbage and maize. These efforts demonstrate the promise of KGs for organizing agricultural knowledge and supporting decision-making.

Although significant progress has been achieved in knowledge graph construction research in recent years, existing studies have primarily focused on general-purpose knowledge graphs or knowledge organization for broad agricultural scenarios, while research on domain-specific knowledge graphs for particular economic crops remains relatively limited (Lin et al., 2021). Existing methods still exhibit considerable limitations in integrating multi-source information into typical knowledge graphs (Peng et al., 2023). In particular, within the garlic cultivation domain, the complexity of pest and disease types, the distinct stage characteristics of the growth cycle, and the strong temporal and regional dependency of cultivation management practices make it difficult for existing general agricultural knowledge graphs to accurately represent complex entity relationships and cross-stage semantic associations throughout the garlic cultivation process. Furthermore, current agricultural knowledge graph research mainly concentrates on major staple crops such as rice, wheat, and maize, whereas studies on domain-specific knowledge graph construction for the entire garlic cultivation process and its integration with LLMs remain insufficient. These compounding challenges collectively highlight the urgent need for a domain-specific, knowledge-grounded intelligent system that can systematically organize garlic cultivation knowledge, constrain LLM generation with structured domain facts, and deliver reliable decision support for complex agricultural scenarios.

Existing GraphRAG studies have demonstrated the effectiveness of integrating knowledge graphs with retrieval-augmented generation for domain-specific question answering. For example, (Jiang et al., 2026) developed a GraphRAG-based research assistant for wound dressing research, showing that graph-enhanced retrieval can improve domain-specific QA performance. However, previous studies have mainly focused on leveraging existing structured knowledge to enhance retrieval and generation, while the influence of knowledge extraction quality on GraphRAG performance remains insufficiently investigated. In this study, we address this issue by improving the upstream knowledge construction process through hybrid clustering-based schema induction, CoT-guided entity-relation extraction, and prompt optimization.

Building upon existing GraphRAG studies while addressing the aforementioned limitations, this study focuses on the garlic cultivation domain and develops a domain-specific KG integrated with LLMs through the GraphRAG framework. Rather than proposing a new GraphRAG architecture, our work enhances the overall knowledge graph construction pipeline to improve the quality of graph-grounded retrieval and downstream question answering.

Compared with existing GraphRAG- and knowledge graph-based approaches for agricultural question answering, the primary contributions of this study lie not in the GraphRAG framework itself, but in improving the quality of domain knowledge construction and graph-grounded retrieval through hybrid clustering, CoT-guided extraction, and prompt reconstruction. The main contributions are summarized as follows:

1. A hybrid clustering-based entity schema induction method for the garlic cultivation domain is proposed. To address the challenges of complex entity categories and the lack of unified classification standards in agricultural texts, Term Frequency–Inverse Document Frequency (TF-IDF) weighting, k-means clustering, and intra-cluster ranking strategies are combined to automatically induce candidate semantic categories from unlabeled agricultural corpora. This approach enables adaptive discovery and optimization of domain entity types, reduces the dependence on manually predefined entity types and relation schemas in traditional knowledge graph construction, and enhances the scalability and transferability of knowledge schema induction across different agricultural subdomains.
2. A triple extraction prompting framework integrating Few-shot learning and Chain-of-Thought (CoT) reasoning is constructed. To address the difficulty of identifying complex relationships in agricultural texts, multiple types of representative few-shot examples and step-by-step reasoning chain prompts are designed to explicitly decompose the processes of entity recognition, relation identification, and triple generation. This framework improves the model’s capability to understand domain-specific semantic relationships and enhances the accuracy and completeness of triple extraction.
3. A knowledge graph-enhanced question answering system for the garlic cultivation domain is developed and locally deployed. Based on the Ollama framework, the system deploys the Qwen2.5:32B model and a local embedding model in an RTX 4090D (24 GB) GPU environment, enabling the implementation of a GraphRAG system without external API dependencies. This design effectively reduces data privacy risks and deployment costs, allowing the system to operate independently in agricultural research institutions or edge computing environments. Experimental evaluations conducted on triple extraction and retrieval-augmented question answering tasks demonstrate that the proposed method outperforms baseline LLMs and traditional RAG methods in terms of Precision, Recall, and F1-score, thereby validating the effectiveness of the proposed framework for agricultural knowledge organization and intelligent question answering.

The overall workflow of this study is summarized as follows: (1) LLMs are configured as garlic domain experts to improve domain adaptability; (2) a hybrid clustering framework integrating TF-IDF weighting, k-means clustering, and intra-cluster ranking is employed to induce candidate semantic categories from unlabeled agricultural texts, thereby identifying entity types most relevant to garlic cultivation; (3) representative and diverse few-shot prompts (10 categories with 5 examples each) are designed (Brown et al., 2020), and the CoT method (Wu et al., 2023) is applied to decompose entity recognition, relation identification, and triple generation into several explicit reasoning steps; (4) the resulting structured knowledge is integrated into the GraphRAG framework, and prompt reconstruction is applied to enrich contextual information during retrieval and generation processes, while the constructed knowledge graph is visualized in Neo4j.

The complete workflow, combined with the GraphRAG framework, constructs a knowledge graph with strong semantic consistency and high entity coverage, injects graph knowledge into the retrieval and generation processes of LLMs, and optimizes the data extraction pipeline. Evaluation is conducted using Precision, Recall, and F1-score metrics for entity–relation triple extraction tasks, as well as retrieval-based question answering accuracy. Experimental results demonstrate that the proposed method achieves strong performance in knowledge triple extraction and retrieval-augmented question answering tasks within the garlic cultivation domain, enhances domain understanding capability, and effectively alleviates hallucination phenomena in LLMs.

## 2 Related work

In recent years, LLMs have shown strong potential across diverse tasks, particularly when paired with external knowledge to improve generation quality. Retrieval-Augmented Generation (RAG) has become a prominent strategy in this space. In specialized, fragmented domains such as agriculture, general-purpose LLMs often struggle to capture complex semantic and logical relations among entities; grounding the model in structured knowledge graphs can improve reasoning, factuality, and consistency. Building on this insight, a structured, hierarchical retrieval-enhanced generation paradigm is adopted, going beyond flat vector similarity, where graph structure is injected into the prompting and retrieval process to reduce hallucinations and improve contextual coherence. In parallel, recent LLMs such as Qwen 2.5:32B offer improved long-context handling, structured outputs, and strong Chinese-language performance, making them suitable backbones for deep integration with agricultural knowledge graphs and prompt engineering.

### 2.1 Qwen 2.5:32B

Qwen 2.5 (Yang A. et al., 2025) represents a substantial advancement over the previous Qwen 1.5 series with improved contextual understanding, multi-turn reasoning, and structured output. Public reports highlight strengths in long-context comprehension (up to very large context windows in available variants), JSON-style structured responses, and performance on Chinese-language tasks relative to widely used open-source baselines. These capabilities make Qwen 2.5 a practical choice for RAG workflows that require faithful grounding and controllable schema-constrained outputs.

**Figure 1:**
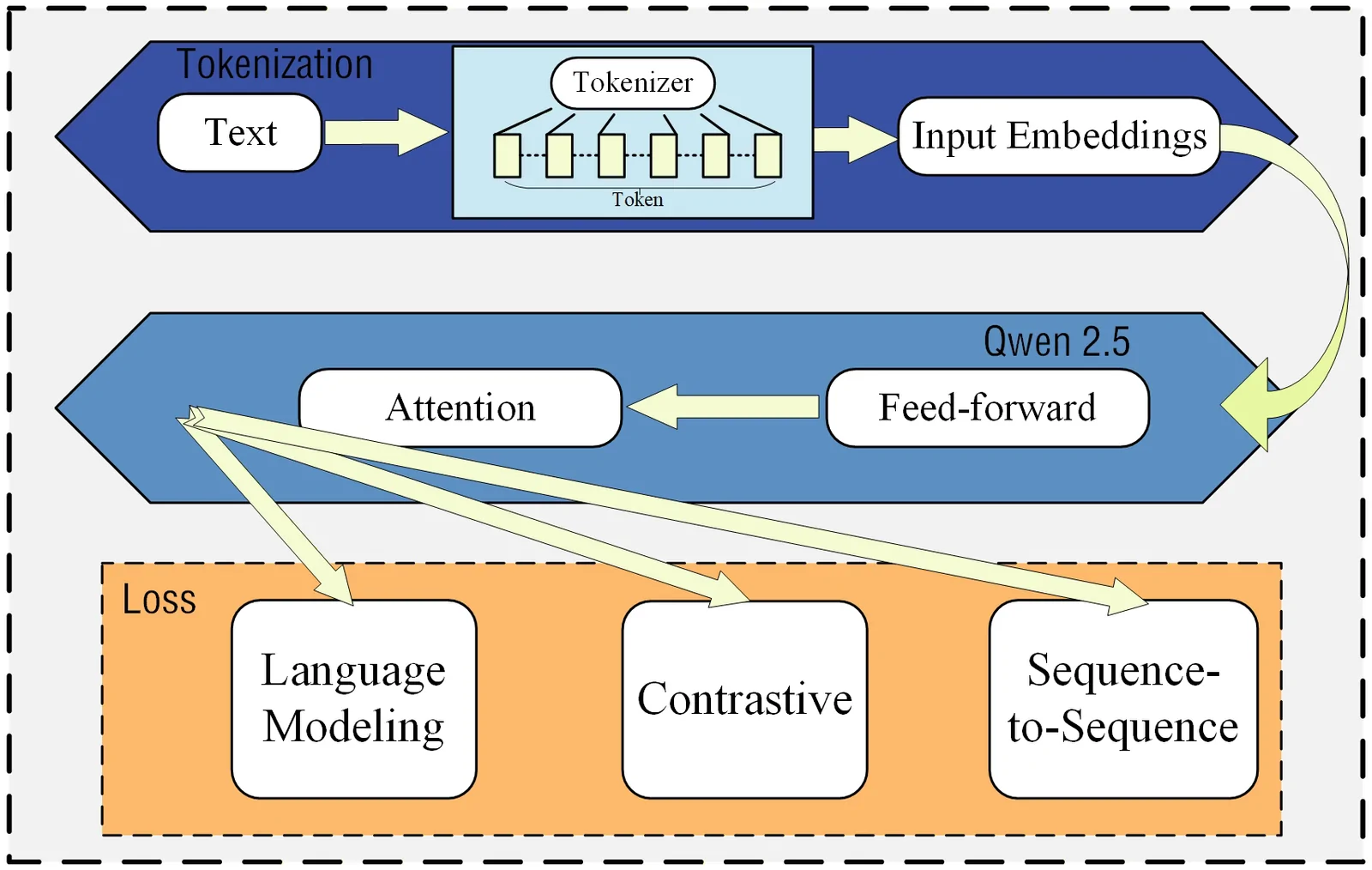
Qwen 2.5:32B overall architecture flow chart: The text is converted into embedded representation, processed by Qwen 2.5:32B model, and trained through three different types of loss functions to support multiple types of tasks.

Beyond raw modeling capacity, Qwen 2.5 shows better instruction following for system prompts and supports configuration for role-specialized tasks. When combined with CoT (Wei et al., 2022) prompting, it yields more coherent multi-step outputs for entity classification, relation extraction, and knowledge organization. In this study, we configure Qwen 2.5:32B as a “garlic-cultivation expert” and guide it with chained prompts to identify agricultural entities and domain-specific relations, producing structured triples suitable for graph construction. The working principle of Qwen 2.5:32B is illustrated in **Figure 1**.

### 2.2 GraphRAG research

**Figure 2:**
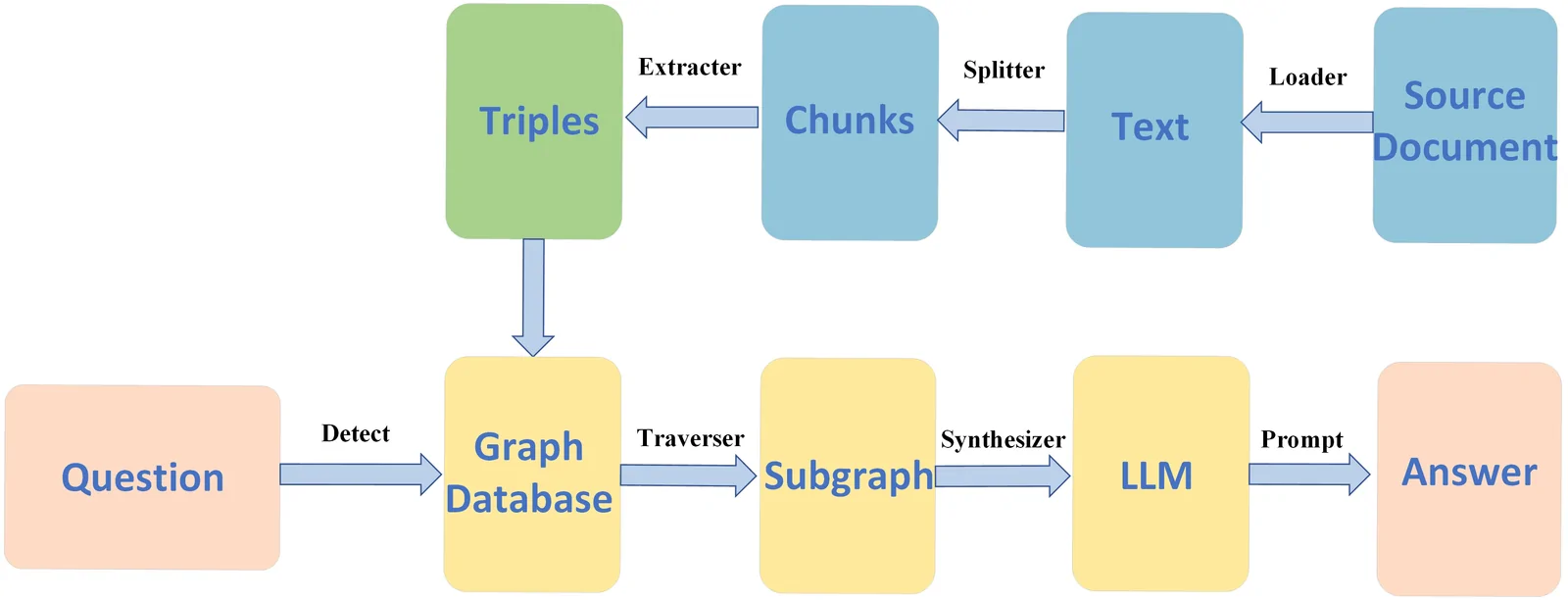
The working principle of GraphRAG: the upper layer is the knowledge extraction and storage stage, which extracts structured triples from documents and builds a graph database. The lower layer is the problem parsing and answering stage, which uses graph information to retrieve subgraphs and combines LLM to generate natural language answers.

GraphRAG extends conventional RAG by fusing vector retrieval with knowledge-graph structure, injecting high-quality, logically connected domain knowledge into the generation process. Concretely, text is chunked, entities and relations are extracted to form triples, and these are stored in a graph database. At query time, GraphRAG retrieves relevant subgraphs and composes graph-aware context prompts for the LLM, which then generates answers grounded in the retrieved structure. This pipeline typically reduces hallucinations and improves answer accuracy and consistency relative to flat vector recall. The working principle of GraphRAG is illustrated in **Figure 2**.

Recent work has explored graph-augmented or hybrid retrieval for domain applications. Yi et al. (2025) proposed a multi-stage cascaded LLM framework integrating multi-source retrieval, knowledge fusion, and domain adaptation for agricultural tasks. Zheng et al. (2025) combined LLMs with nested contract knowledge graphs via GraphRAG to support contract retrieval and reasoning. Wei et al. (2025) applied GraphRAG techniques in materials science to enhance chemical information retrieval and literature-based discovery. Wu et al. (2026) proposed Crop GraphRAG, integrating knowledge graphs with RAG for crop pest and disease question answering, demonstrating that graph-structured retrieval reduces hallucinations in agricultural QA. However, their work focuses on generalized crop categories rather than domain-specific cultivation management for particular economic crops such as garlic. These studies support the view that explicit graph structure strengthens grounding and enables more reliable reasoning than vector-only retrieval.

This study builds on these ideas by coupling GraphRAG with CoT-guided extraction and prompt reconstruction. We decompose entity recognition, relation inference, and triple generation into stepwise prompts that specify role, procedure, and output schema, with in-context examples to reduce uncontrolled generalization. Unlike traditional RAG, that relies on “soft” similarity matches, GraphRAG here establishes explicit edges among entities (e.g., linking “white rot” → “pests and diseases” → “effects on root development”), thereby improving the structural accuracy and coverage of retrieved evidence. For instance, for a query such as “How should spring drought be managed for Zhaosu six-clove garlic?”, the system can reason along the path garlic variety → regional climate → drought-management measures, outperforming static knowledge-base lookups or flat vector recall.

Although existing studies have demonstrated that RAG methods possess a certain degree of interpretability and scalability in knowledge-enhanced tasks, traditional vector retrieval-based RAG approaches encounter difficulties in effectively modeling complex multi-entity relationships and multi-hop reasoning structures within the agricultural domain. Therefore, GraphRAG is adopted as the foundational framework in this study to enhance the representation capability of structured associations among entities through the explicit construction of a knowledge graph. Furthermore, the integration of the CoT prompting mechanism improves the interpretability and logical consistency of reasoning paths, making the framework more suitable for agricultural knowledge question-answering (QA) scenarios.

## 3 Materials and methods

Within the research framework of integrating knowledge graphs with LLMs, constructing a high-quality knowledge graph tailored to the agricultural vertical domain and effectively guiding LLMs to perform structured knowledge extraction represents a key pathway for enhancing domain-specific reasoning capabilities. Focusing on garlic cultivation as a representative knowledge-intensive agricultural scenario, this study proposes a comprehensive graph-model collaborative methodology by introducing the GraphRAG framework to achieve knowledge graph-enhanced LLMs. In zero-shot and few-shot training settings, LLMs are capable of addressing various challenges, including knowledge graph construction, completion, reasoning, and question answering. Leveraging their information extraction capabilities under zero-shot or few-shot learning, LLMs can perform entity and relation extraction tasks from text or other data sources, reducing the time and cost of data annotation. Additionally, they can serve as supplementary knowledge sources to extract reliable information and complete knowledge graph enrichment.

**Figure 3:**
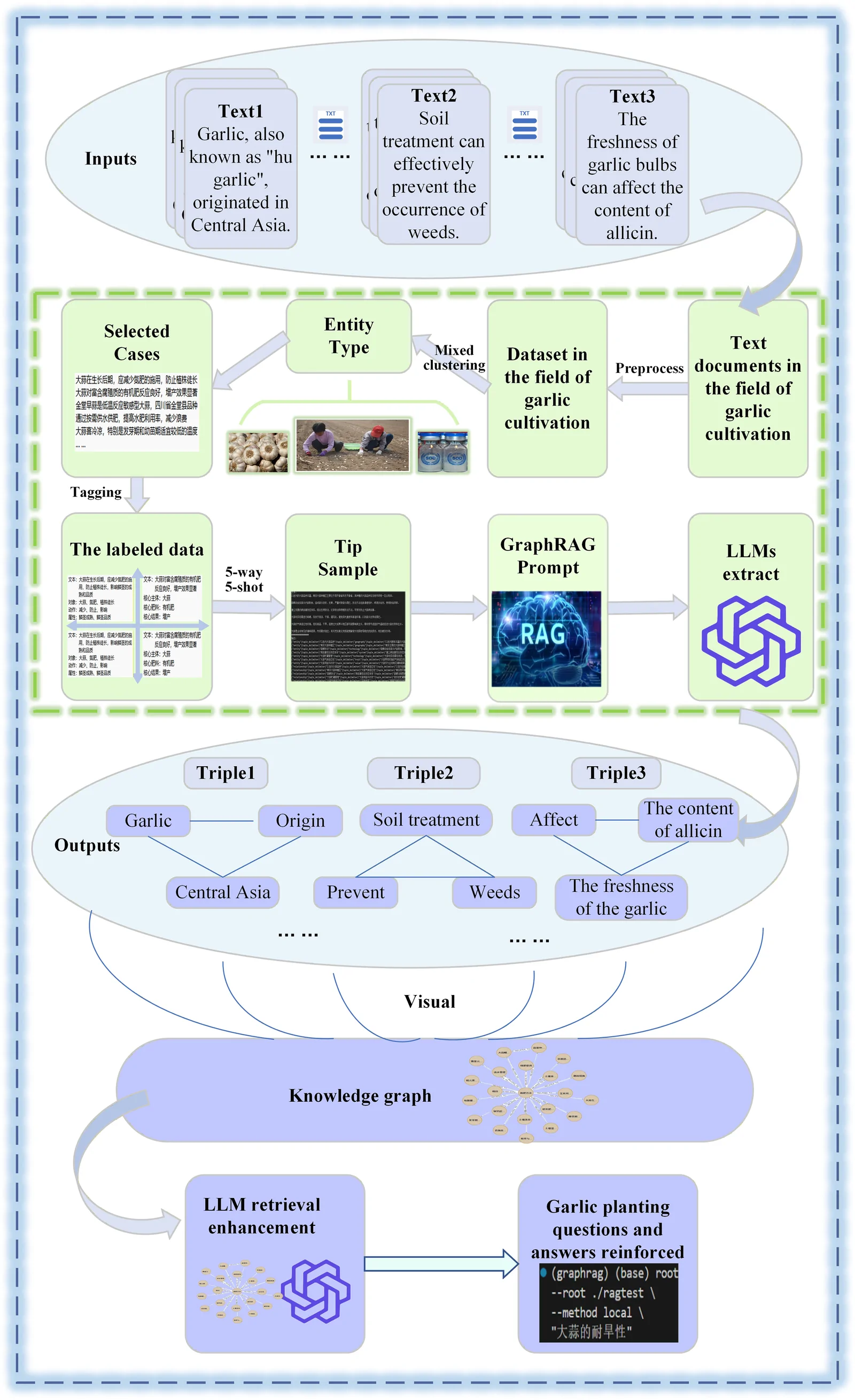
The overall framework diagram of this paper’s research: by using hybrid clustering to select high-quality texts and build prompt engineering, LLM and GraphRAG framework are used for triplet extraction and question answering optimization, and finally a knowledge graph is constructed to serve the intelligent question answering system in garlic planting field.

This chapter describes how a garlic-cultivation-corpus is assembled, domain entity types are induced, entities and relations are extracted with Qwen 2.5:32B, a Neo4j knowledge graph is built, and the graph is integrated with GraphRAG for retrieval-augmented question answering. It also details preprocessing, prompt design, evaluation, and implementation settings to ensure reproducibility. The research framework of this study is illustrated in **Figure 3**.

### 3.1 Dataset preparation

**Figure 4:**
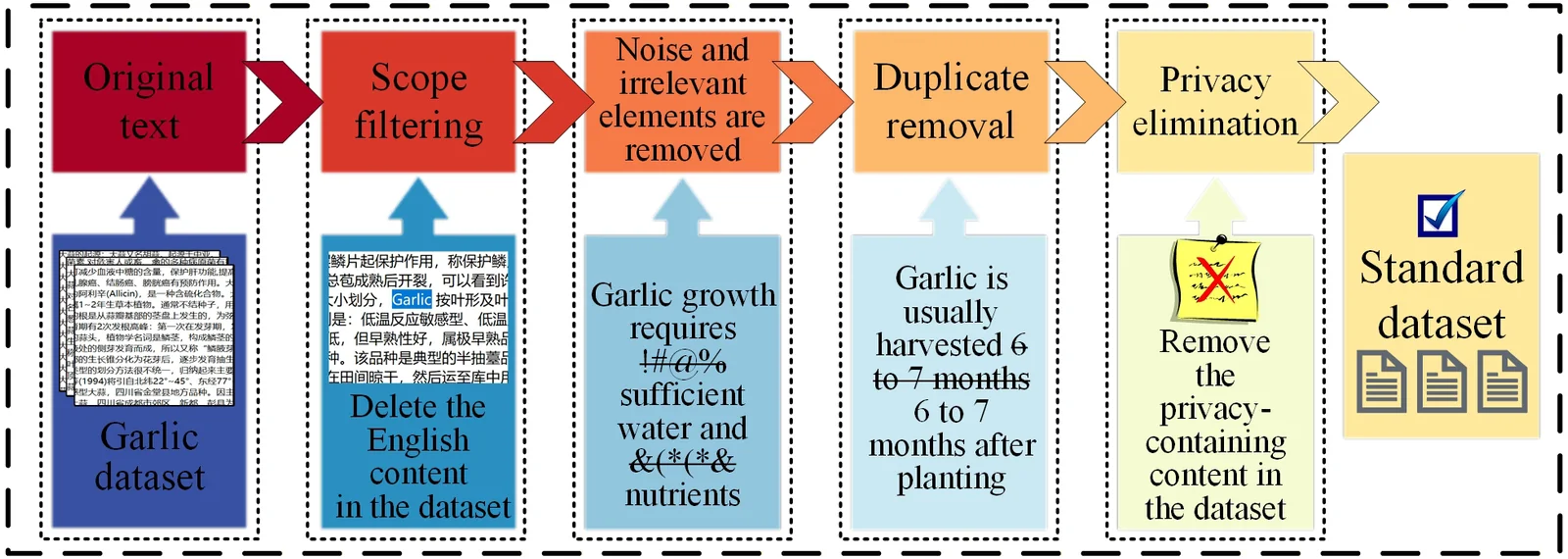
Preprocessing process diagram: it shows the complete processing process from raw data to standard data set, including key steps such as quality filtering, noise removal, deduplication and privacy protection.

The construction of a knowledge graph involves multiple stages, including data collection, data preprocessing, knowledge extraction, knowledge fusion, knowledge storage, and retrieval, among which data collection and preprocessing constitute the essential foundation for building a high-quality knowledge graph. The knowledge extraction process requires a large amount of domain-related textual data as support. Therefore, a self-constructed dataset was adopted in this study, in which textual content related to garlic cultivation was collected and extracted from multiple domain-specific agricultural reference books and technical manuals. Based on these sources, a garlic cultivation domain corpus containing information on cultivation periods, planting regions, cultivation techniques, and pest and disease control was established. Since the original texts contain inconsistent formatting, semantic redundancy, repetitive descriptions, and certain noisy information, data preprocessing is required prior to knowledge graph construction in order to improve the accuracy of subsequent knowledge extraction. The specific preprocessing workflow is illustrated in **Figure 4**.

The preprocessing consists of the following steps: (i) scope filtering—retaining Chinese content and discarding non-Chinese passages to preserve domain terminology; (ii) noise removal—eliminating hyperlinks, boilerplate, special characters, and other non-semantic artifacts via keyword and pattern rules; (iii) de-duplication—collapsing near-duplicate passages to avoid bias and enhance diversity; and (iv) privacy protection—identifying and anonymizing sensitive or personally identifiable information. Given the modest corpus size, automated rules were combined with manual screening to ensure high precision.

After preprocessing, the final corpus contained 2,300 text entries covering garlic varieties, phenology, cultivation methods, fertilization, and pests and diseases control. Because the GraphRAG pipeline relies on few-shot prompting rather than model fine-tuning, the full corpus was used as the evaluation pool for extraction and question answering.

### 3.2 Entity type construction via hybrid clustering

This section aims to automatically induce domain-specific entity schemas from agricultural corpus through hybrid clustering.

**Figure 5:**
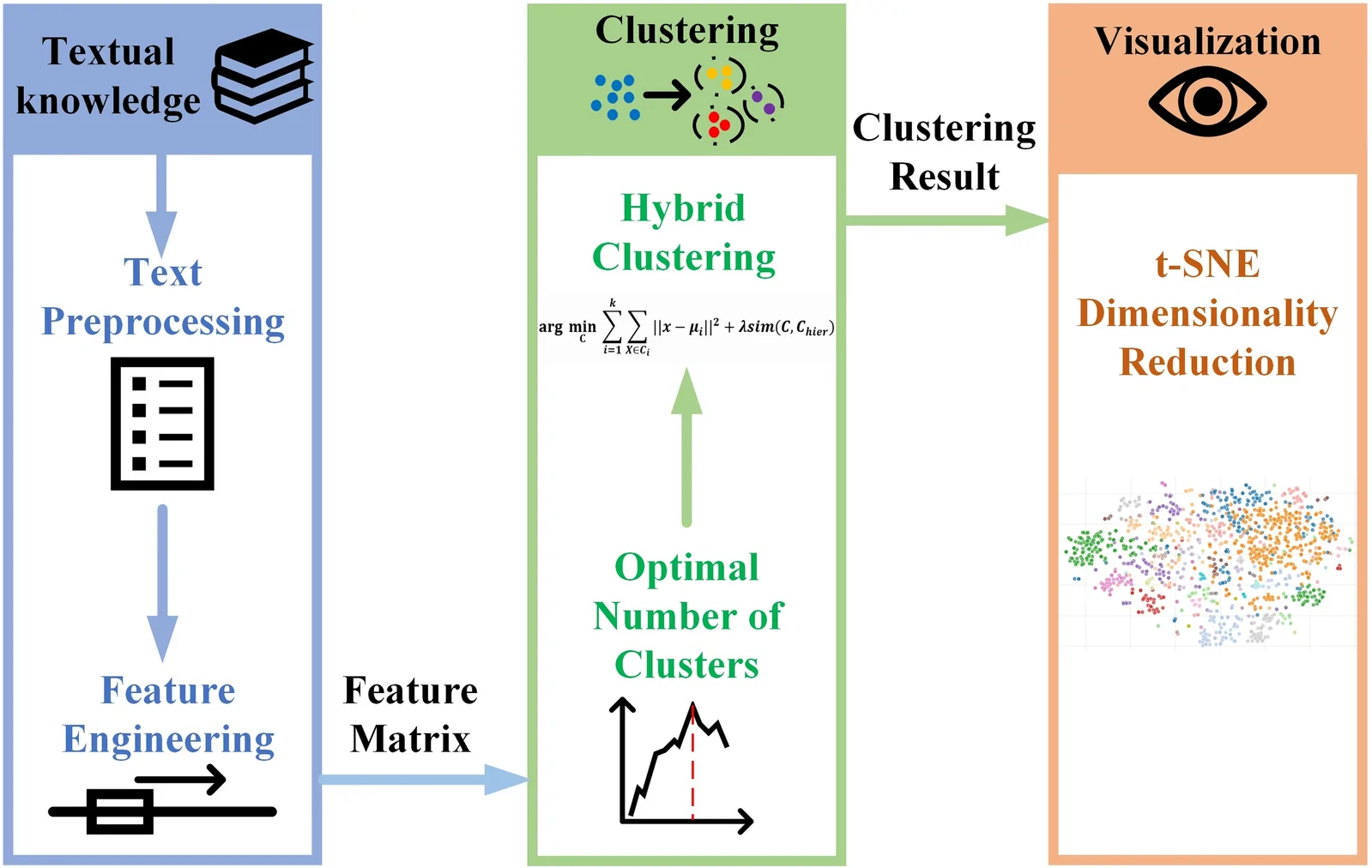
Flowchart of clustering processing: transform textual knowledge into feature matrices, perform clustering on the feature matrices, and visualize the final results through t-SNE dimensionality reduction.

To reduce expert burden and provide the LLM with a stable schema for extraction, domain entity types were induced directly from the corpus using a hybrid clustering framework, as shown in **Figure 5**. Clustering is an unsupervised learning method (Shafay et al., 2025). Text segments were vectorized with term-weighting features (TF-IDF over uni/bi/tri-grams), optionally reduced in dimension, and clustered in two stages: hierarchical clustering to capture global structure, followed by k-Means to refine local partitions. The number of clusters was selected using a combination of the elbow method, Gap statistic, and silhouette coefficient, balancing cohesion and separation.

Within each cluster, candidates were ranked by composite salience (termhood, TF-IDF weight, domain-keyword proximity), and cluster exemplars were reviewed by domain experts to assign clear, reusable labels. This process yielded ten core entity types: Pests and Diseases, Functional Role, Planting Time, Chemical Fertilizer, Variety, Technique, Characteristic, Management, Physiological State, and Environmental Condition. These types served as the schema for subsequent entity recognition, relation definition, and triple construction.

#### 3.2.1 Enhanced feature engineering

A dynamic n-gram-based TF-IDF feature extraction method was applied to improve feature discrimination through the following optimizations: (i) setting an N-gram range of 1–3 to capture phrase-level semantics; (ii) implementing L2 normalization to mitigate document length bias; and (iii) introducing sublinear TF scaling to suppress the weights of high-frequency terms. The construction of the feature matrix follows Equation 1:

(1) $$F_{ij}=\frac{tf(w_{j},d_{i})\cdot \log(\frac{N}{df(w_{j})+1})}{{\left\|d_{i}\right\|}_{2}}$$

where *F_ij_* denotes the final weight of term *w_j_* in document *d_i_*; *tf*(*w_j_*,*d_i_*) is the term frequency of *w_j_* in *d_i_*; *N* is the total number of documents; *df*(*w_j_*) denotes the number of documents containing the term *w_j_*; and ||*d_i_*||_2_ represents the L2 norm of document vector di. The experiment retained the top 200 features by TF-IDF weight, filtered rare terms with document frequency< 2, and removed highly generic terms occurring in > 85% of documents. This yielded a compact, domain-salient representation suitable for clustering and downstream extraction.

#### 3.2.2 Clustering optimization strategy

Determining the optimal number of clusters k* is a core challenge in text clustering (Sinaga and Yang, 2020). Single evaluation metrics (such as the elbow method or silhouette coefficient) are often affected by data distribution and noise, potentially leading to local optima. To address this, a threefold validation mechanism was introduced: first, gap statistics were used to quantify the deviation of actual data from a uniform reference distribution, addressing the subjectivity of traditional elbow methods; second, the silhouette coefficient assessed intra-cluster compactness and inter-cluster separability; and finally, the elbow method’s curve inflection point provided an additional reference. This multi-perspective strategy compensates for the limitations of any single metric. For example, when gap statistics recommended a suboptimal *k* due to data skewness, the silhouette and elbow coefficients jointly corrected the bias.

A reference distribution was generated using Monte Carlo sampling, and the logarithmic difference in within-cluster sum of squares (WCSS) between the actual data and the reference distribution was calculated. The degree of deviation between the actual data and the reference distribution was then used to objectively identify the presence of genuine cluster structures according to Equation 2.

(2) $$Gap(k)=\frac{1}{B}\sum_{b=1}^{B}\log(W_{k}^{*(b)})-\log(W_{k})$$

Where *W_k_* is the WCSS of k-means clustering; *B* is the number of bootstrap samples. When *Gap*(*k*) first reaches a plateau satisfying *Gap*(*k*)≥*Gap*(*k* + 1)−*S_k+1_*, the corresponding *k* is identified as the optimal solution.

The silhouette coefficient evaluates clustering results according to Equation 3 by calculating the average distance between a data point and other points within the same cluster, as well as the average distance to points in different clusters (Shi et al., 2021), thereby addressing the limitations of statistical hypothesis testing methods such as the Gap statistic.

(3) $$\;s(k)=\frac{1}{n}\sum_{i=1}^{n}\frac{b(i)-a(i)}{\max\left\{a(i),b(i)\right\}}$$

Here, *a*(*i*) denotes the average distance from sample i to other samples within the same cluster (reflecting intra-cluster cohesion), while *b*(*i*) represents the average distance from sample *i* to samples in the nearest neighboring cluster (reflecting inter-cluster separation).

The Elbow Method, recognized as a classical approach for determining the optimal number of clusters in clustering analysis (Liu and Deng, 2021), is fundamentally based on the monotonicity characteristic of clustering quality as the number of clusters increases. Instead of relying on subjective visual inspection, this method applies numerical analysis by computing the first-order difference Δ(*k*) and the second-order difference Δ’(*k*) of the within-cluster sum of squares (WCSS) as defined in Equations 4 and 5.

(4) Δ(k)=WCSS(k)−WCSS(k+1)

(5) $$\Delta^{\prime }\left(k\right)=\Delta \left(k\right)-\Delta (k-1)$$

When Δ′(*k*) first falls below a threshold θ (experimentally set as *θ*=− 0.05 × max(Δ′)), it is identified as an effective inflection point. Finally, by jointly considering the maximum Gap statistic, the highest silhouette coefficient value *s*(*k*), and the elbow coefficient, the optimal cluster number k* is selected as the final clustering solution.

#### 3.2.3 Hybrid clustering algorithm

Hybrid clustering algorithms combining different distance measures effectively handle mixed-attribute data (numerical, categorical, textual, etc.). In this study, a hybrid model integrating hierarchical clustering and k-means was designed: (1) average-linkage hierarchical clustering was first used to generate initial cluster centers, ensuring that centers are located in the density cores of actual clusters and overcoming k-means initialization sensitivity; and (2) the hierarchical cluster center is used as the initialization seed of k-means++ for optimization, and the fine division ability of k-means is fully utilized. This strategy combined the noise robustness of hierarchical clustering with the efficient iteration of k-means, improving both robustness and efficiency.

#### 3.2.4 Evaluation and visualization

**Figure 6:**
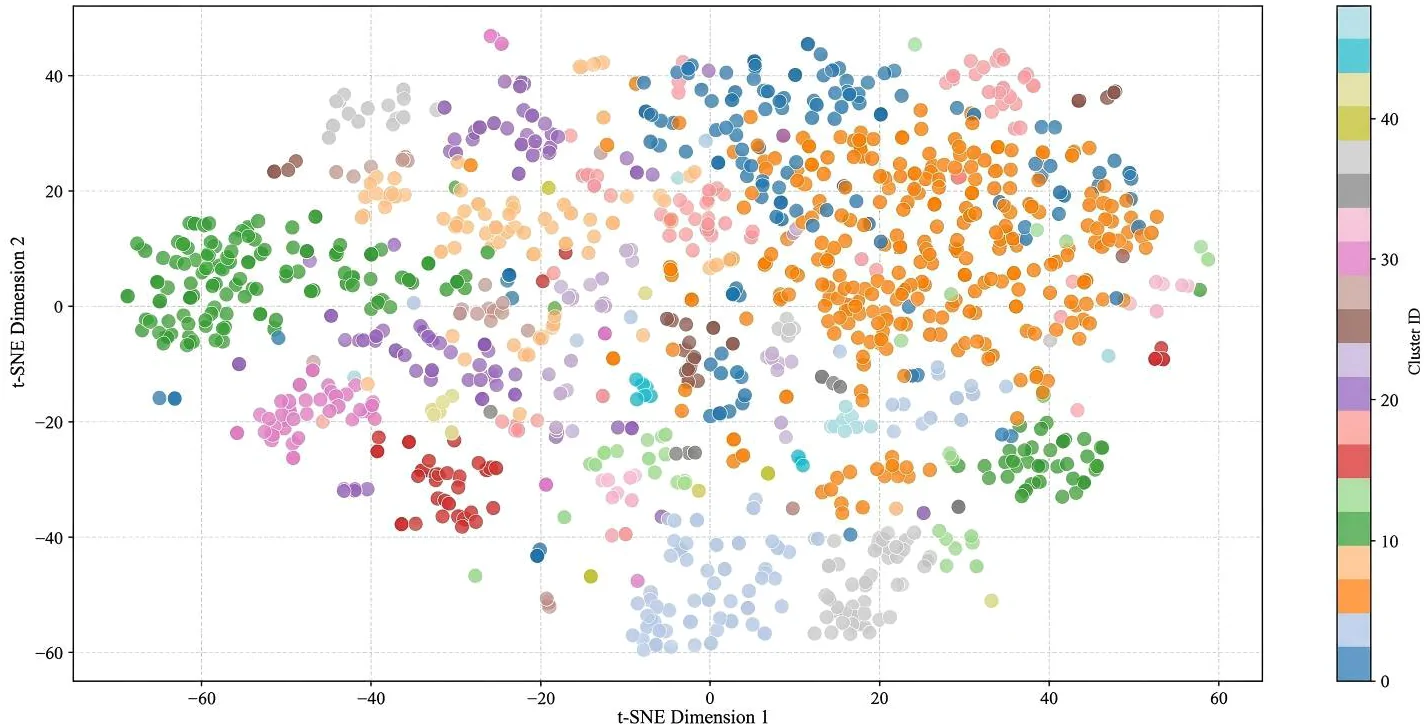
Garlic cultivation knowledge clustering(t-SNE).

A multi-dimensional evaluation system was implemented to ensure the clustering results achieved comprehensive performance in terms of statistical significance, domain relevance, and interpretability: (1) Expert evaluation: two agricultural experts independently annotated 50 randomly selected samples with thematic categories (such as “soil management” and “pests and diseases control”) to assess the algorithm’s accuracy; (2) Visualization: two-dimensional distribution was presented using t-SNE dimensionality reduction. Key parameters were determined through grid search, as shown in **Figure 6**.

### 3.3 Knowledge extraction with few-shot learning and CoT guidance

The knowledge extraction process is formulated as a structured pipeline consisting of three sequential stages: entity recognition, relation inference, and triple generation, guided by CoT prompting.

#### 3.3.1 Few-shot learning

With the rise of artificial intelligence, deep learning has been increasingly applied in agriculture and plant sciences. However, the remarkable performance of deep learning models relies on the availability of large-scale datasets. In plant sciences and biological research, obtaining extensive annotated data remains challenging. The emergence of few-shot learning has addressed this limitation (Yang et al., 2022). Few-shot learning has been successfully applied to crop disease recognition in real field conditions, demonstrating strong generalization across domain gaps between controlled laboratory datasets and real-world field scenarios (Yang et al., 2024). Zero-shot learning enables models to learn new data without any labeled samples, while one-shot learning relies on just a single example. Few-shot learning falls between these two approaches, providing more training samples and enabling LLMs to learn new information from a limited number of instances. By incorporating a few-shot learning mechanism, the GraphRAG prompt engineering process was optimized, leading to a significant improvement in LLM performance within the garlic cultivation domain.

**Figure 7:**
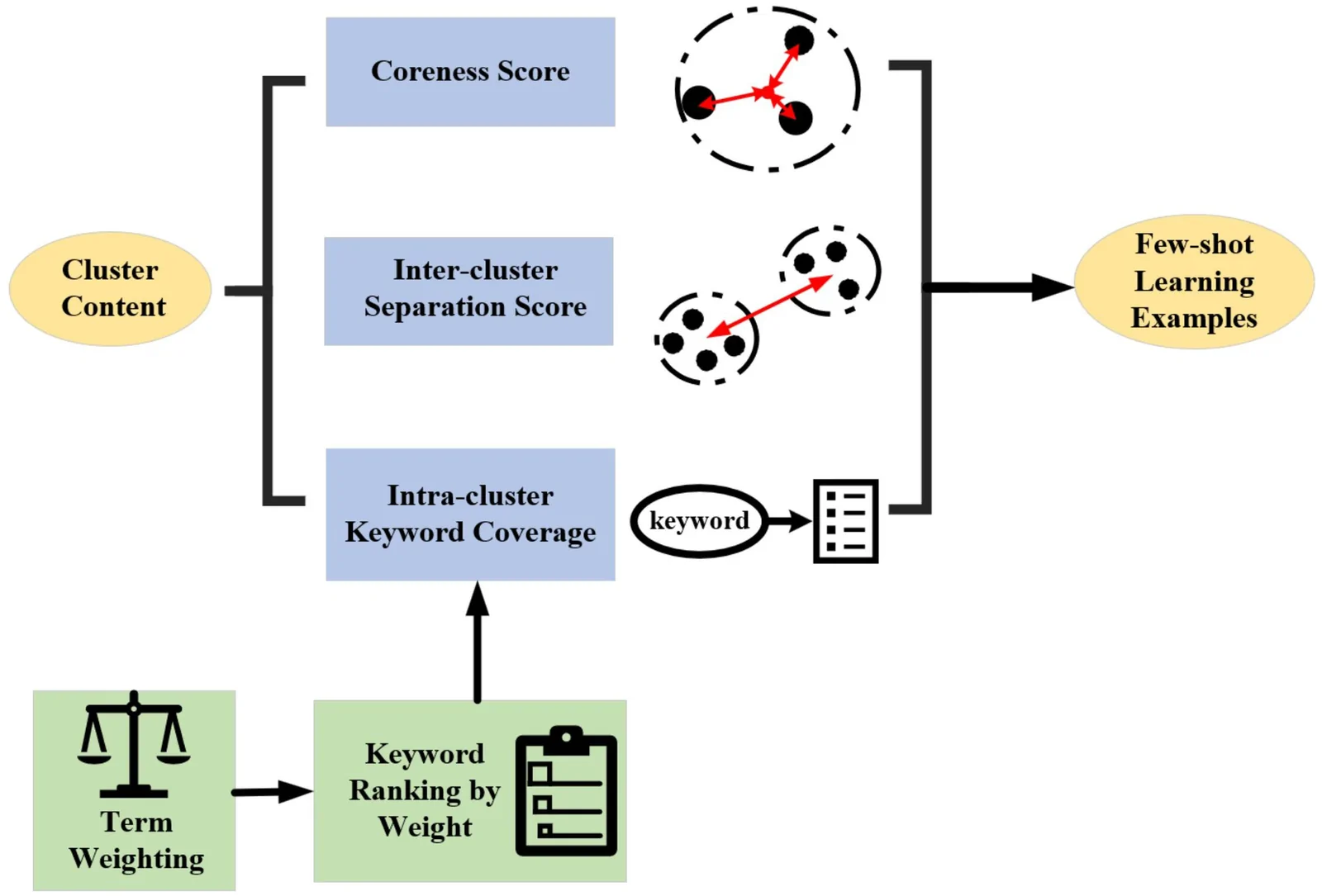
Rank and select few-shot learning cases from clustered textual knowledge through three metrics: coreness score, inter-cluster separation score, and intra-cluster keyword coverage.

This study proposes a domain knowledge representation framework that integrates enhanced TF-IDF feature weighting with hybrid hierarchical clustering, aiming to extract semantically consistent knowledge units from unstructured agricultural texts to generate high-quality samples for few-shot language model training. The framework features dynamic keyword weight computation and a multi-dimensional intra-cluster ranking strategy. The sample extraction workflow is illustrated in **Figure 7**.

For the calculation of domain-adaptive keyword weights, an enhanced TF-IDF algorithm was employed to construct the feature space, with the weight defined according to Equation 6.

(6) $$w_{i,j}=\frac{tf_{i,j}\times \log(\frac{N}{df_{i}+1})}{\sqrt{\sum_{k=1}^{V}{\left(tf_{k,j}\times \log\left(\frac{N}{df_{k}+1}\right)\right)}^{2}}}$$

where *tf_i_*,*_j_* denotes the frequency of term *i* in document *j*; *df_i_* represents the number of documents containing term *i*; *N* is the total number of documents; *V* is the vocabulary set; and *k* denotes the term.

The proposed improvements include the use of a domain-specific lexicon constraint by predefining 86 core garlic cultivation terms (e.g., “sandy loam”, “crop rotation”) and adding specialized vocabulary (e.g., “garlic scape”), thereby ensuring the feature space remains domain-specific and professional. A part-of-speech filtering mechanism was adopted to extract nouns and verbs from document knowledge points, retaining words longer than one character and excluding stop words to reduce noise. An N-gram extension was introduced to capture compound terms (e.g., “pests and diseases control”), addressing the semantic compositionality of agricultural phrases. Finally, the TF-IDF matrix was normalized by rows (||*x*||_2_ = 1) to eliminate the influence of document length differences.

After the hybrid clustering process, each cluster still contains multiple candidate knowledge documents with different representativeness and information quality. Therefore, instead of treating all documents equally, an intra-cluster multidimensional ranking strategy was introduced to identify the most representative knowledge samples within each cluster. Specifically, each document was evaluated from three complementary perspectives, namely cluster relevance, inter-cluster distinctiveness, and keyword coverage. A multi-dimensional intra-cluster ranking strategy was applied: for each document xi in cluster *C_k_*, three metrics were calculated. First, the core relevance score *S_c_*(*x_i_*) was derived by computing the cosine similarity with the cluster centroid. Next, the distinctiveness score *S_d_*(*x_i_*) was measured by assessing separability from other clusters. Subsequently, the proportion of the Top-10 cluster keywords contained in each document was calculated to obtain the keyword coverage score *Cov*(*x_i_*). The final ranking score was then determined according to Equation 7.

(7) $$Rank\left(x_{i}\right)=0.5S_{c}\left(x_{i}\right)+0.3S_{d}\left(x_{i}\right)+0.2Cov(x_{i})$$

After evaluation, documents within each cluster were ranked in descending order based on their final scores, and the top five ranked knowledge samples were selected for few-shot training, forming a 10-way 5-shot sample set. Knowledge selected using this method is characterized by strong representativeness, distinct knowledge features, and high differentiation from knowledge in other clusters. This approach enables LLMs to better understand subtle task distinctions, thereby enhancing contextual comprehension and improving performance in knowledge graph construction tasks.

#### 3.3.2 CoT guided extraction

**Figure 8:**
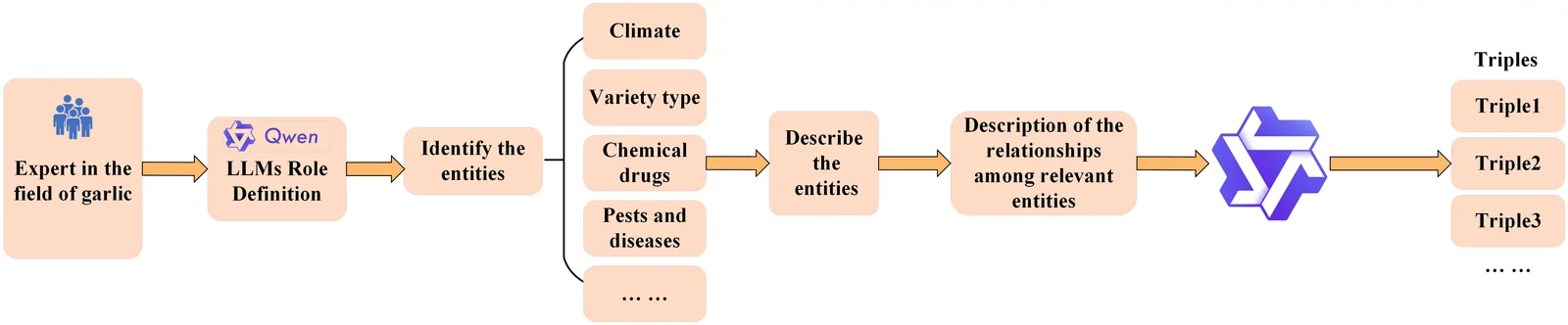
Chain-of-thought prompting: LLM is assigned the role of garlic domain experts to guide the identification of all entities and the description of relationships among these entities, and finally generate a triplet file through their output.

In this study, specific objectives were defined for the LLM by assigning it the role of a garlic cultivation expert. Subsequently, task steps were provided, and the prompt samples constructed were introduced into the prompt design. First, based on the entity types established in Section 3.2, the LLM was guided to identify all entities and provide descriptions of the corresponding entities. Second, the LLM was directed to identify two explicitly related entities and describe the relationship between them, explaining the rationale linking the source entity and the target entity. Finally, the LLM was prompted to output a structured set of triples, comprising the head entity, tail entity, and their relationship. In addition, statements, community reports, and other prompt elements were adjusted to ensure domain relevance. The prompt engineering workflow is illustrated in **Figure 8**.

### 3.4 GraphRAG integration and question answering framework

#### 3.4.1 Prompt engineering optimization

**Figure 9:**
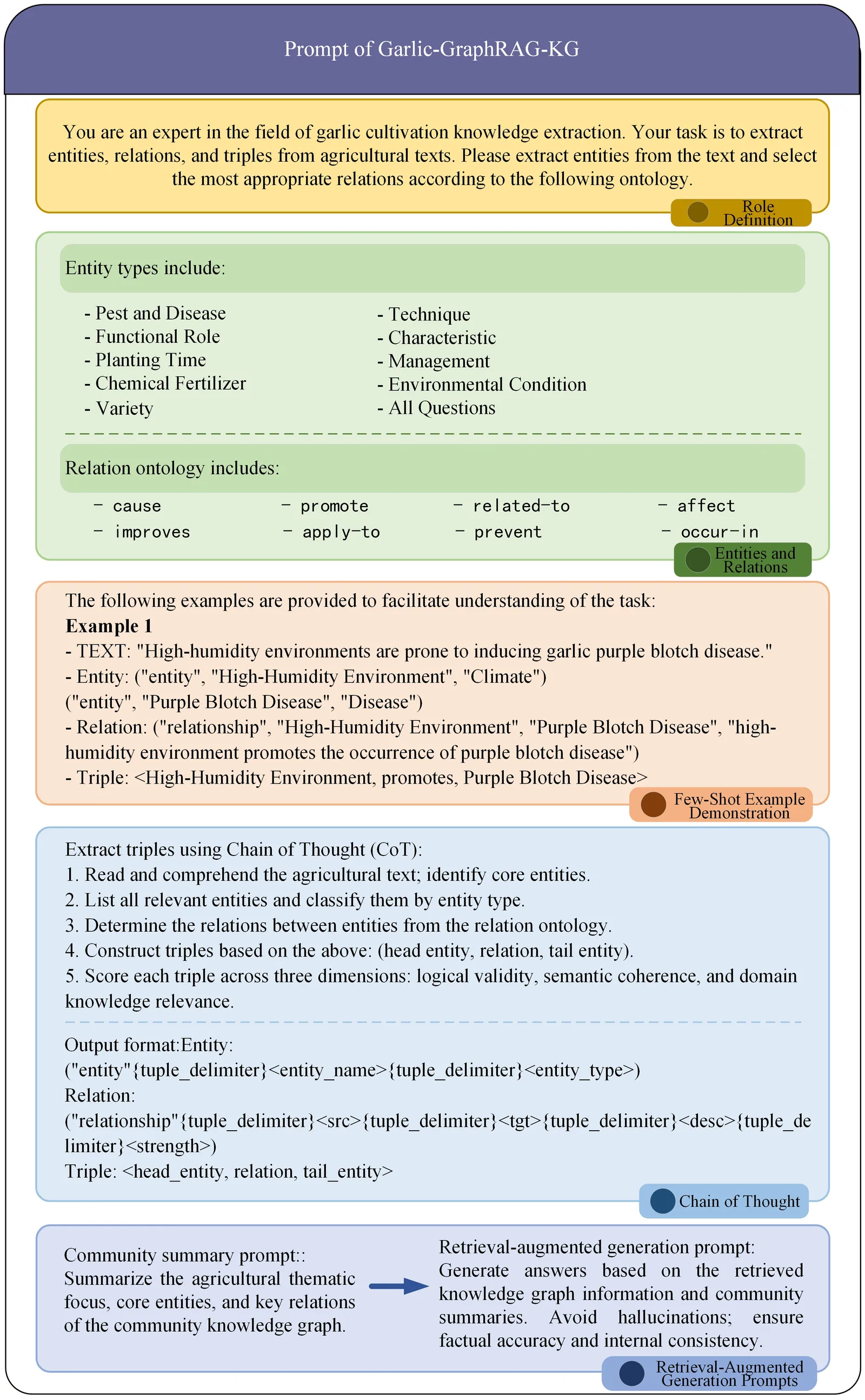
Prompt engineering workflow of Garlic-GraphRAG-KG: role definition, entity and relation ontology, few-shot example demonstration, chain-of-thought extraction, and retrieval-augmented generation.

High-quality prompts are critical for improving the performance of knowledge graph extraction. Prompt engineering optimization aims to enhance the adaptability of LLMs to domain-specific tasks (Sabbatella et al., 2024). In this study, GraphRAG served as the knowledge-enhancement framework responsible for defining entity and relation extraction rules, supplying the schema for subsequent knowledge graph construction, performing community partitioning and semantic summary generation, and offering a structured retrieval mechanism. The locally deployed LLM functioned as the semantic execution unit, conducting entity recognition, relation extraction, and natural language generation under the guidance of GraphRAG-designed prompt templates. However, due to the lack of stable domain-specific knowledge constraints, LLMs are prone to factual errors and domain hallucinations in agricultural scenarios. GraphRAG addresses this limitation by structurally organizing entities and their relationships within the text and integrating the Leiden community detection algorithm for community partitioning and subgraph retrieval, thereby providing more accurate contextual knowledge support for LLMs. Based on this framework, the default GraphRAG prompt templates were domain-optimized, and a CoT reasoning strategy was introduced to improve the accuracy of entity-relation extraction and community retrieval in the garlic cultivation domain. The prompt engineering workflow is illustrated in **Figure 9**.

#### 3.4.2 Knowledge graph construction

Based on the domain-optimized prompt templates designed in Section 3.4.1, GraphRAG leverages the few-shot learning capability of LLMs to extract entities and relations from input documents, thereby constructing a knowledge graph. Under CoT guidance, the pipeline first recognized entities according to the induced schema, then, inferred relations following in-prompt templates, and finally emitted schema-valid triples. This produced a structured, domain-specific graph for downstream retrieval and generation.

**Figure 10:**
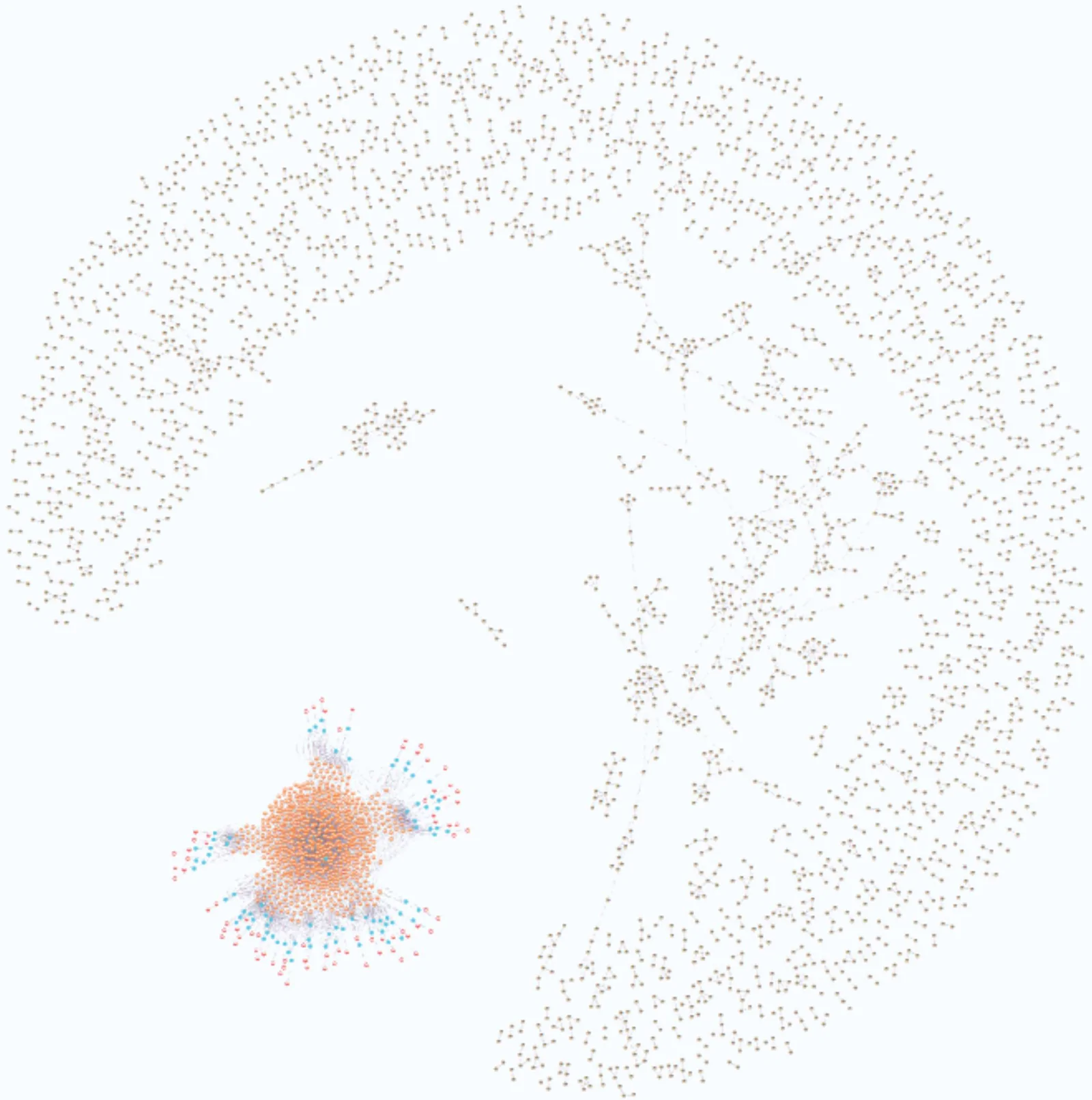
The overall visualization of the garlic planting knowledge graph.

**Figure 11:**
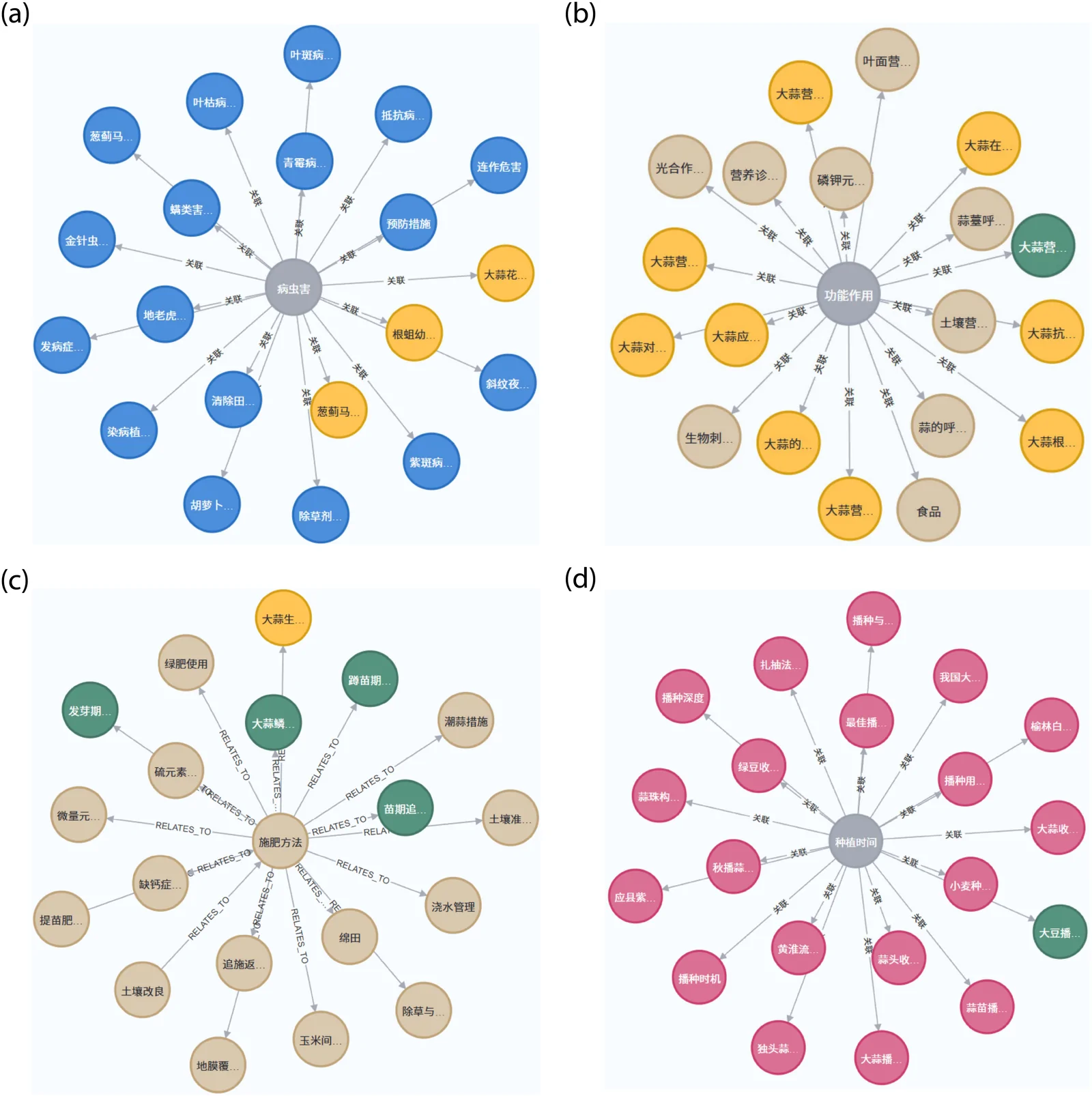
Subgraphs related to four types of entities: pests and diseases, functional roles, planting time, and fertilization methods. **(a)** Pests and diseases subgraph. **(b)** Function and role subgraph. **(c)** Planting time subgraph. **(d)** Fertilization methods subgraph.

In this study, Neo4j was used to visualize the constructed knowledge graph. A total of 8,500 triples and 8,671 entities were extracted, and the resulting knowledge graph was stored and visualized in Neo4j. **Figure 10** shows the global view, **Figures 11a–d** highlights four representative subgraphs (pests and diseases, functional roles, planting periods, fertilization methods). These views illustrate dense local connectivity around key agronomic concepts and provide interpretable evidence trails for QA.

#### 3.4.3 GraphRAG retrieval mechanism

Traditional RAG methods typically perform text retrieval based on vector similarity, relying on embedding models to compute semantic distances between queries and text chunks. However, in agricultural knowledge scenarios, there exist complex interrelationships among crop varieties, growth environments, pest and disease conditions, and management practices. Sole dependence on semantic similarity often fails to capture the structured relationships between entities, resulting in incomplete retrieval results or insufficient logical coherence. Therefore, this study introduces a GraphRAG retrieval mechanism on top of a knowledge graph to achieve structurally enhanced retrieval of agricultural knowledge by integrating vector-based retrieval with graph-structured retrieval.

During the retrieval phase, the system first encodes the user query into a vector representation and employs an embedding model to compute semantic similarity between the query and both entity nodes and textual chunks in the knowledge base, thereby obtaining an initial set of relevant nodes. Subsequently, starting from these retrieved core entities, a k-hop neighborhood expansion is conducted within the knowledge graph to obtain related nodes and edges, forming a subgraph structure relevant to the query semantics. This process overcomes the limitation of traditional vector retrieval, which focuses only on local semantic matching, and enables multi-hop knowledge association based on entity relationship chains. For example, when a user inputs “how to manage garlic under spring drought conditions”, the system not only retrieves content directly related to “drought”, but also further expands along relationship paths such as “climatic conditions—water management—fertilization practices—disease risk”, thereby obtaining more comprehensive contextual information.

Compared with traditional RAG approaches, GraphRAG improves the structural completeness and semantic coherence of retrieval results by explicitly modeling relationships among entities. In addition, to reduce interference from irrelevant nodes during the generation process, the expanded subgraph is filtered for relevance, retaining only entity–relation structures highly pertinent to the current query. The resulting subgraph is then linearized into a textual representation suitable for LLM inputs. Finally, the graph-enhanced structured knowledge context is utilized in the downstream QA generation stage to improve both the accuracy and reasoning capability of the model in complex agricultural scenarios.

#### 3.4.4 QA generation framework

After completing knowledge graph construction and GraphRAG-based retrieval, a knowledge graph–enhanced QA system for the garlic cultivation domain is further developed. The system is built upon locally deployed LLMs, enabling intelligent retrieval and generative question answering over agricultural domain knowledge, thereby improving the accuracy and interpretability of responses to complex agricultural queries.

The overall system follows a processing pipeline consisting of “user query input—GraphRAG retrieval—context construction—LLM generation.” Upon receiving a user query, the system first employs the GraphRAG mechanism to retrieve relevant entity nodes, relational paths, and subgraph structures associated with the query. The retrieved structured knowledge is then linearized to construct a context prompt suitable for LLM comprehension. Compared with traditional RAG approaches that rely solely on raw text passages as input, this method provides explicit entity–relation structures to the model, thereby strengthening its ability to understand logical relationships within agricultural domain knowledge. During the QA generation stage, the user query and the GraphRAG-generated structured context are jointly fed into the LLM, which performs reasoning and answer generation based on the retrieved knowledge. To reduce the risk of hallucinated information during generation, structured prompting is employed to constrain the output space of the model, encouraging responses to be grounded in entity relationships extracted from the knowledge graph. This improves both the factual reliability and consistency of the generated answers.

Compared with traditional vector-based retrieval QA systems, the proposed GraphRAG-enhanced QA system leverages structured relational information in the knowledge graph to perform associative retrieval and context augmentation for complex agricultural questions. As a result, it demonstrates improved adaptability in multi-entity relationship understanding, multi-hop reasoning, and answer accuracy within agricultural domain question answering tasks.

## 4 Results and discussion

In this study, a garlic cultivation knowledge graph was first extracted and visualized, and subsequently integrated with LLMs, enabling the implementation of RAG techniques. Finally, both (i) the QA performance of the graph-augmented system and (ii) the quality of extracted triples was evaluated to verify the scientific validity of the graph and the effectiveness of the pipeline.

### 4.1 Experiment deployment

To prevent information leakage and reduce costs while ensuring data privacy and controllability, a locally deployed GraphRAG system was implemented on the Ollama platform. By default, GraphRAG uses OpenAI’s API; in this study, an RTX 4090D (24 GB) graphics card was rented. The base environment was configured with PyTorch 2.5.1, Python 3.12 (ubuntu 22.04), and CUDA 12.4. Due to hardware constraints, Qwen 2.5:32B and the nomic-embed-text embedding model were selected. Qwen 2.5:32B is a foundational LLM containing 32 billion parameters, supporting a context length of up to 128K tokens and 29 languages, with notable enhancements in specialized domains such as coding and mathematics. Compared to its predecessor Qwen 2, Qwen 2.5 shows significant improvements in instruction following, long-text generation (exceeding 8K tokens), understanding of structured data (e.g., tables), and the generation of structured outputs, particularly in JSON format. The Qwen 2.5 model also demonstrates greater adaptability to various system prompts, enhancing role-playing capabilities and chatbot conditional configurations, which provided substantial support for prompt refinement in this study. Nomic-embed-text is a tool designed for text embedding and natural language processing, typically used to convert textual data into high-dimensional vector representations. These vectors can be effectively applied in machine learning, text analysis, and other natural language processing tasks such as text classification, similarity computation, clustering, and semantic search. By combining advanced embedding techniques, it enables rapid and efficient processing of large-scale text data, making it suitable for diverse language processing tasks. To ensure a fair comparison, Qwen 2.5:32B was adopted as the unified backbone model across all baseline and proposed configurations, including the Basic LLM, Basic LLM+RAG, GraphRAG (Before), and GraphRAG (After) settings evaluated in this study. The only variables across these configurations are the retrieval mechanism and prompt engineering strategy, thereby isolating the contribution of each component.

### 4.2 Quantitative evaluation

The system was evaluated along two axes: (1) graph-model integration, i.e., whether GraphRAG improves QA relative to a text-only LLM; and (2) triple quality, i.e., whether prompt refinements yield more complete and accurate triples, thereby strengthening retrieval and generation.

#### 4.2.1 Effectiveness of graph–model integration

Based on the dataset, a set of test questions was organized, covering ten categories of entity types, including pests and diseases, functional effects, planting periods, and fertilization methods, with 20 retrieval items in each category. The selection ensured that each item was representative. Examples of these test questions are shown in **Table 1**.

**Table 1:** Test question example.

Retrieval type	Example queries
Pest and Disease	Q1: What are the occurrence patterns of aphid damage to garlic?
	Q2: How should garlic white rot disease be controlled?
Functional Role	Q1: What kinds of condiments can be made from garlic?
	Q2: What medicinal value does garlic have?
Planting Time	Q1: In which season is it suitable to plant garlic in the Huang-Huai-Hai region?
	Q2: In which month is it appropriate to plant Zhaosu six-clove garlic?
Fertilization Method	Q1: How should garlic be fertilized during the early growth stage?
	Q2: At which growth stage is phosphate fertilizer suitable for garlic?

To comprehensively evaluate the proposed framework, four representative configurations with progressively increasing levels of knowledge enhancement were selected:

1. Base LLM without external retrieval;
2. Traditional RAG using vector retrieval only;
3. Original GraphRAG using graph-structured retrieval;
4. The proposed enhanced GraphRAG integrating hybrid clustering, Few-shot learning, CoT prompting, and prompt reconstruction.

These four configurations were consistently adopted in both the question-answering evaluation (Section 4.2.1) and the triple extraction evaluation (Section 4.2.2), enabling systematic comparisons under the same experimental settings.

QA accuracy, defined as the proportion of responses containing all required key elements, was evaluated by domain experts across ten categories (**Table 2**). The Base LLM achieved an overall accuracy of 39.5%, while traditional RAG improved it to 56.0%, demonstrating that external retrieval significantly enhances domain knowledge coverage. GraphRAG further increased performance to 68.0%, indicating that graph-structured retrieval effectively improves multi-hop reasoning and entity relationship grounding. After prompt optimization, GraphRAG achieved the highest accuracy of 77.0% (70.6%-82.3%), representing a 9.0 percentage point absolute improvement over the original GraphRAG. Notably, most categories exceeded 70% accuracy under the optimized GraphRAG, suggesting that prompt refinement further strengthens knowledge utilization and reduces hallucination in domain-specific agricultural question answering.

**Table 2:** Question-answering accuracy comparison across different retrieval configurations.

Retrieval type	Samples	Base LLM Acc./%	Traditional RAG Acc./%	Original GraphRAG Acc./%	Enhanced GraphRAG Acc./%
Pest and Disease	20	30.0	65.0	65.0	70.0
Functional Role	20	40.0	60.0	65.0	75.0
Planting Time	20	50.0	55.0	70.0	70.0
Chemical Fertilizer	20	35.0	55.0	75.0	80.0
Variety	20	45.0	60.0	60.0	80.0
Technique	20	40.0	55.0	65.0	85.0
Characteristic	20	35.0	55.0	70.0	85.0
Management	20	35.0	50.0	75.0	80.0
Physiological State	20	40.0	50.0	70.0	75.0
Environmental Condition	20	45.0	55.0	65.0	70.0
All Questions	200	39.5	56.0	68.0	77.0

To further illustrate the practical performance of the proposed framework, representative QA examples are presented below, including one successful QA case and one failed QA case.

Representative QA Examples

Successful QA Case

Question: Where is Zhaosu Six-Clove Garlic a local variety from?

Reference Answer: Zhaosu Six-Clove Garlic is a local variety from Zhaosu County, Xinjiang Uygur Autonomous Region, China.

Enhanced GraphRAG Response: Zhaosu Six-Clove Garlic is a local variety from Zhaosu County, Xinjiang Uygur Autonomous Region, China.

Analysis: The enhanced GraphRAG framework correctly identified the origin of Zhaosu Six-Clove Garlic by retrieving the corresponding knowledge graph entity and incorporating the retrieved evidence into response generation. The generated answer is fully consistent with the reference answer, demonstrating the framework’s ability to provide factually grounded responses for domain-specific questions.

Failed QA Case

Question: Where is Xinhui Fire Garlic a local variety from?
Reference Answer: Xinhui Fire Garlic is a local garlic variety from Xinhui County, Guangdong Province, China.
Enhanced GraphRAG Response: Xinhui Fire Garlic is a local garlic variety from Kaiping, Guangdong Province, China.
Analysis: The enhanced GraphRAG framework incorrectly identified Kaiping as the origin of Xinhui Fire Garlic. This error is likely caused by semantic similarity between cultivar names, as both Xinhui Fire Garlic and Jinshan Fire Garlic belong to Guangdong Province and contain the term “Fire Garlic.” Although GraphRAG substantially reduces hallucinations, incorrect entity retrieval or ambiguity in graph retrieval may still propagate inaccurate evidence to the language model, resulting in an incorrect answer.

#### 4.2.2 Triple quality

Because triples are the atomic units of the KG, we assessed precision, recall, and F1 score using expert-annotated references. The calculation formulas for these three evaluation metrics are presented in Equations 8–10.

(8) $$Precision=\frac{TP}{TP+FP}$$

(9) $$Recall=\frac{\text{TP}}{TP+FN}$$

(10) $$F1=\frac{2\times Precision\times Recall}{Precision+Recall}$$

where *TP* (*True Positives*) represents the number of correctly identified triples, *FP* (*False Positives*) denotes the number of incorrectly identified triples, and *FN* (*False Negatives*) refers to the number of correct triples that were not identified.

(1) Evaluation dataset and ground truth construction

An evaluation dataset was constructed based on the preprocessed agricultural corpus, comprising 2,300 garlic cultivation-related texts covering multiple aspects, including pest and disease control, varietal characteristics, cultivation management, fertilization techniques, and physiological growth. To ensure the objectivity and reliability of the evaluation, a manually annotated Ground Truth triple set was established for all evaluation texts. The annotation content included Head Entity, Relation, and Tail Entity, forming a standardized triple dataset for comparative evaluation against model-generated results. During the annotation process, two researchers with agricultural domain expertise independently annotated the data according to unified entity-type and relation-definition guidelines. Samples with inconsistent annotations were subsequently reconciled through cross-discussion and manual review to ensure consistency and accuracy of the final annotations.

To further assess the reliability of the manually constructed Ground Truth dataset, inter-annotator agreement was quantitatively evaluated based on the independent annotations from the two researchers. Specifically, Cohen’s Kappa coefficient was calculated to measure the consistency between the two annotators. The obtained Kappa value of 0.78 indicates substantial agreement, demonstrating that the annotation guidelines were sufficiently clear and that the constructed Ground Truth dataset provides a reliable basis for subsequent evaluation of triple extraction performance.

(2) Evaluation method and matching rules

During the model evaluation stage, considering that locally deployed LLMs may exhibit expression variability and generation diversity in agricultural knowledge extraction tasks, the use of a strict matching strategy alone—requiring the Head Entity, Relation, and Tail Entity to be completely identical—could easily underestimate model performance. Therefore, in addition to traditional strict matching, this study introduced Semantic Equivalent Matching and rule-constrained auxiliary judgment mechanisms to provide a more reasonable evaluation of triple extraction quality.

Specifically, when the triples generated by the model conveyed the same factual meaning as the manually annotated results, they were regarded as correctly extracted triples (True Positives, TP). For example, within disease-control relations, different expressions such as “prevention method”, “control approach”, and “treatment measure” were considered equivalent if they referred to the same semantic relation. Similarly, entity names with synonymous expressions, abbreviations, or minor lexical variations (e.g., “garlic scape” and alternative spellings referring to the same concept) were treated as successfully matched as long as the semantic reference remained unchanged.

The evaluation was conducted according to the following principles:

Entity Matching Principle: Synonyms, aliases, and minor expression differences were allowed, provided that they referred to the same agricultural concept entity;

Relation Matching Principle: Semantically equivalent relations were mapped into the same relation category, such as “prevent”, “control”, and “suppress” under specific contextual conditions;

Semantic Consistency Principle: The overall semantic structure of the triple had to remain logically valid, ensuring consistency among the head entity, relation, and tail entity.

Based on these principles, semantically equivalent triples between model outputs and the Ground Truth were counted as *TP*; generated triples that could not be semantically aligned with any standard triples were counted as *False Positives* (*FP*); and valid Ground Truth triples not covered or identified by the model were counted as *False Negatives* (*FN*).

This evaluation strategy not only improves evaluation fairness, but also better reflects the semantic understanding capability of knowledge extraction systems in practical application scenarios, thereby providing a more realistic assessment of the performance of different methods in garlic cultivation knowledge graph construction tasks.

To minimize potential bias introduced by semantic equivalence matching, semantic equivalence was only accepted when the generated triple preserved the same factual meaning as the Ground Truth triple. Mere lexical similarity or partial overlap was not sufficient for a correct match. All evaluations followed the predefined matching criteria shown in **Table 3**.

**Table 3:** Criteria for semantic equivalence matching in triple evaluation.

Matching type	Accepted cases	Rejected cases
Entity equivalence	Synonyms, abbreviations, aliases, and minor spelling variations referring to the same agricultural entity	Different entities, varieties, locations, or concepts
Relation equivalence	Different expressions describing the same relationship under the same context	Relations with different meanings or causal directions
Attribute/value equivalence	Different wording describing the same property or value	Missing information, conflicting attributes, or unsupported facts
Overall triple consistency	Head entity, relation, and tail entity maintain the same factual meaning as Ground Truth	Any semantic contradiction or entity mismatch

(3) Experimental Settings

The complete set of 2,300 texts was used as the testing dataset for triple extraction and evaluation to ensure that the evaluation results comprehensively reflected model performance in garlic cultivation knowledge extraction tasks.

(4) Result Description

Using the same four retrieval configurations described in Section 4.2.1, we further evaluated triple extraction quality in terms of Precision, Recall, and F1 score. After prompt refinement, precision increased by 13.0 percentage points, recall by 37.0 percentage points, and F1 by 28.2 percentage points (**Table 4**).

**Table 4:** Triple extraction performance comparison across different system configurations.

Item	Precision/%	Recall/%	F1 Score/%
Basic LLM	66.0	40.0	49.8
Traditional RAG	69.0	56.0	61.8
Original GraphRAG	75.0	68.0	71.3
Enhanced GraphRAG	79.0	77.0	78.0

Experimental results demonstrate significant performance differences among the compared methods in the triple extraction task. Due to the lack of domain-specific knowledge support, the baseline LLM was prone to entity omission, relation confusion, and hallucinated generation when processing complex agricultural semantic relations, resulting in relatively low Precision, Recall, and F1-score values. After introducing relevant contextual information through vector retrieval, the traditional RAG system improved the model’s capability to utilize domain knowledge, leading to better triple extraction performance compared with the baseline LLM. However, because the traditional RAG framework relies primarily on semantic similarity retrieval without modeling entity relationships or structured knowledge, limitations remained in complex relation reasoning and cross-sentence semantic association tasks.

In contrast, the GraphRAG system incorporated entity associations and structured semantic information from the knowledge graph to enhance the retrieval context through graph-structured augmentation, thereby improving the model’s understanding of agricultural domain knowledge. Experimental results indicate that the GraphRAG system without prompt optimization outperformed both the baseline LLM and the traditional RAG system in terms of Precision, Recall, and F1-score, demonstrating that knowledge graph structures can effectively enhance the domain knowledge modeling capability of LLMs and alleviate hallucination issues to a certain extent.

Furthermore, the enhanced GraphRAG system with prompt optimization achieved the best overall experimental performance. Few-shot examples improved the model’s ability to learn domain-specific entity and relation patterns, while the CoT reasoning process explicitly decomposed entity recognition, relation identification, and triple generation into intermediate reasoning steps, thereby improving the accuracy and completeness of complex agricultural semantic relation extraction. Experimental results show that the enhanced GraphRAG system significantly outperformed all other comparison methods in Precision, Recall, and F1-score, indicating that prompt engineering optimization can further unlock the potential of the GraphRAG framework in agricultural knowledge extraction tasks.

Overall, the comparative experiments demonstrate a clear performance progression from parameter-only generation (Base LLM), vector-based retrieval (Traditional RAG), graph-enhanced retrieval (GraphRAG), to the proposed enhanced GraphRAG framework. These results indicate that each additional knowledge enhancement mechanism contributes positively to knowledge extraction quality, with graph-structured retrieval providing greater improvements than vector retrieval alone, while prompt optimization further enhances the utilization of graph knowledge.

#### 4.2.3 Ablation study

To evaluate the contribution of different prompt optimization strategies, an ablation study was conducted. Few-shot learning and CoT-guided reasoning were progressively introduced into the original GraphRAG framework. The results of the ablation study are presented in **Table 5**.

**Table 5:** Ablation study of different components in the proposed GraphRAG framework.

Method	Precision/%	Recall/%	F1/%
GraphRAG	75.0	68.0	71.3
GraphRAG + Few-shot	76.2	72.1	74.1
GraphRAG + CoT	77.0	73.5	75.2
Full Framework	79.0	77.0	78.0

The results show that both Few-shot learning and CoT-guided reasoning contribute positively to triple extraction performance. Compared with the original GraphRAG, Few-shot learning improves the F1-score by providing representative domain examples, while CoT-guided reasoning enhances the model’s ability to identify entities and infer relations through explicit reasoning processes. The combination of both strategies achieves the best performance.

#### 4.2.4 Hallucination reduction analysis

To further validate the hallucination reduction capability of the proposed framework, a human evaluation was conducted on randomly selected QA samples. Agricultural domain experts evaluated the generated responses according to their factual consistency with the reference knowledge. Each response was categorized as Faithful (all factual statements were supported by the reference knowledge), Partial (the response was factually correct but incomplete), or Hallucinated (the response contained unsupported or factually incorrect information). The evaluation results are presented in **Table 6**.

**Table 6:** Hallucination evaluation results across different methods.

Method	Faithful/%	Partial/%	Hallucinated/%
Base LLM	38	27	35
Traditional RAG	54	28	18
Original GraphRAG	67	23	10
Enhanced GraphRAG	76	18	6

As shown in **Table 6**, the Base LLM exhibited the highest hallucination rate, indicating that relying solely on parametric knowledge frequently produced factually incorrect or unsupported responses. Introducing vector-based retrieval in the Traditional RAG framework reduced hallucinations by providing relevant external context. Compared with Traditional RAG, Original GraphRAG further decreased the hallucination rate, suggesting that graph-structured knowledge retrieval improves factual grounding through explicit entity–relation modeling. The proposed Enhanced GraphRAG achieved the lowest hallucination rate among all compared methods, demonstrating that Few-shot learning and CoT prompting further improve the model’s ability to generate factually consistent responses.

Combined with the QA accuracy results presented in **Table 2**, these findings demonstrate that the proposed framework not only improves question-answering performance but also enhances factual consistency by reducing hallucinated responses.

#### 4.2.5 Statistical analysis

To assess the statistical uncertainty of the reported overall question-answering accuracy, 95% confidence intervals (95% CI) were calculated using the Wilson score interval based on the 200-question evaluation dataset. Each question was treated as an independent binary outcome (correct or incorrect), and the overall QA accuracy was estimated as the proportion of correctly answered questions. The corresponding results are presented in **Table 7**.

**Table 7:** 95% confidence intervals for overall QA accuracy.

Method	Overall QA Acc./%	95% CI
Base LLM	39.5	32.9-46.5
Traditional RAG	56.0	49.1-62.7
Original GraphRAG	68.0	61.2-74.2
Enhanced GraphRAG	77.0	70.6-82.3

### 4.3 Computational overhead analysis

Although the proposed GraphRAG framework improves domain-specific question answering performance, the additional computational overhead introduced by knowledge graph construction and graph-based retrieval should also be considered. In this study, the computational costs mainly arise from two stages: knowledge graph construction and QA inference.

During knowledge graph construction, additional operations, including entity extraction, relation identification, embedding generation, and graph organization, are required compared with conventional LLM-based approaches. These procedures introduce an additional preprocessing cost before the QA stage. However, this knowledge construction process only needs to be performed once for a given domain corpus, and the resulting knowledge graph can be reused for subsequent queries. Therefore, the initial construction cost can be amortized over multiple downstream QA tasks. During the QA inference stage, GraphRAG introduces additional operations, including entity matching, graph traversal, and relevant subgraph retrieval, compared with standalone LLM inference. These additional retrieval steps inevitably increase response latency. Nevertheless, the retrieved graph-based knowledge provides structured entity-relation information and improves the grounding of generated responses, which contributes to higher QA accuracy and reduced hallucination risks. When scaling to larger agricultural datasets containing tens of thousands of unstructured farmer logs, the computational burden is expected to mainly increase in the offline knowledge construction stage, including feature extraction, hybrid clustering, entity extraction, embedding generation, and graph updating, while graph-based retrieval remains efficient by focusing on relevant subgraphs rather than the entire knowledge base.

In this study, the knowledge graph was constructed from a garlic cultivation corpus containing 2,300 text entries and integrated with a locally deployed Qwen2.5:32B model. Although the enhanced GraphRAG framework requires additional computational resources compared with a standalone LLM, it achieved a substantial improvement in overall QA accuracy, increasing from 39.5% for the Base LLM to 77.0% for the enhanced GraphRAG framework. These results indicate that the additional computational overhead provides practical benefits for domain-specific question answering scenarios where answer reliability and knowledge consistency are critical.

### 4.4 Discussion

Experimental and evaluation results demonstrate that improvements and refinements in GraphRAG prompt engineering lead to superior performance compared with the original model in terms of accuracy, recall, F1-score, and QA performance. These enhancements effectively improve the performance and domain adaptability of LLMs while also mitigating hallucination phenomena. It is important to note that GraphRAG itself is not responsible for knowledge graph construction; instead, it performs semantic-enhanced retrieval based on a pre-constructed knowledge graph. In this study, an LLM-based entity recognition and relation extraction approach, combined with expert validation, was adopted to construct a structured knowledge graph for the garlic cultivation domain. The resulting graph was subsequently integrated into the GraphRAG retrieval module, providing precise and structured knowledge context for LLMs and thereby improving generation quality. The primary advantages arise from the synergy of two mechanisms: first, the introduction of the knowledge graph provides explicitly structured semantic relationships in retrieved content, effectively mitigating information redundancy and semantic drift commonly observed in traditional vector-based retrieval; second, prompt optimization strategies enhance the model’s understanding of agricultural terminology and user intent, resulting in responses that are more closely aligned with the core of the query.

To further evaluate the interpretability and multi-hop reasoning capability of the proposed enhanced GraphRAG system in complex agricultural scenarios, a case study involving a composite question related to cultivar characteristics, regional environmental conditions, and field management practices was conducted, with a comparative analysis against the baseline LLM.

The test question is as follows:

“How should early-maturing garlic planted in autumn be managed under spring drought conditions?”

For this question, the baseline LLM was able to capture only a superficial association between “spring drought” and “irrigation.” However, due to the absence of structured domain knowledge constraints, the generated response exhibited a certain degree of generalization. The model provided generic agricultural recommendations such as “increase irrigation frequency” and “apply moderate fertilization”, without incorporating specific analyses of cultivar type, growth stage, or environmental conditions. Key agronomic practices such as water management during the greening stage and control strategies during the bulb expansion stage were not addressed, indicating domain hallucination and knowledge insufficiency.

In contrast, the GraphRAG system performed multi-hop reasoning and retrieval based on entity relationships within the knowledge graph. The system first identified the key entities “early-maturing garlic planted in autumn” and “spring drought”, and then executed the following reasoning path within the knowledge graph: early-maturing garlic planted in autumn → belongs to autumn-sown varieties → sensitive to water conditions during the greening stage → spring drought affects bulb expansion → requires enhanced irrigation management during the greening stage → combined with phosphorus and potassium fertilization to promote root recovery. This reasoning path systematically connects cultivar type, growth stage, environmental conditions, and management measures, thereby providing the LLM with semantically structured contextual information. Based on this, the GraphRAG-generated response not only recommended strengthening irrigation during the greening stage under spring drought conditions, but also proposed targeted agronomic measures such as avoiding flood irrigation, increasing phosphorus and potassium application, and promoting root recovery and bulb development. The overall response is more consistent with agricultural production practices.

This case demonstrates that traditional LLMs primarily rely on parametric knowledge for probabilistic generation, which may lead to generalized domain responses and fragmented reasoning chains. In contrast, GraphRAG leverages entity relation paths within a knowledge graph to perform structured semantic reasoning, enabling explicit utilization of relationships among cultivar type, environmental conditions, growth stages, and management strategies. This significantly enhances explainability and reasoning accuracy in complex agricultural QA tasks, further validating the effectiveness of the proposed approach in agricultural intelligent QA scenarios.

Although a structured garlic cultivation knowledge graph was constructed and achieved favorable results, the data source is primarily derived from four agricultural textbooks, representing relatively standardized and static textual data. Such a data source ensures accuracy and consistency of knowledge but limits the system’s ability to cover complex and heterogeneous data in real-world agricultural production scenarios. For instance, practical smart agriculture applications often involve sensor time-series data, farmer experiential records, pest and disease images, and other multimodal environmental information. These dynamic and multimodal data types were not incorporated into the knowledge graph construction process in this study; therefore, further expansion is required to improve adaptability in real agricultural scenarios.

In addition, although the proposed framework was compared with Base LLM, traditional RAG, and the original GraphRAG framework, comparisons with other KG-enhanced LLM approaches and fine-tuned domain-specific LLMs were not included in this study. This is mainly because the objective of this work was to investigate the effect of knowledge graph construction and prompt optimization on domain-specific knowledge grounding within the GraphRAG framework, rather than modifying the parameters of the underlying LLM. Future studies will further evaluate the proposed framework against more diverse KG-augmented approaches and domain-adapted LLMs to assess its generalizability.

## 5 Conclusion

This study addressed domain grounding and factuality in agricultural QA by integrating a garlic cultivation knowledge graph to enhance the accuracy and domain adaptability of LLMs in specialized QA tasks related to garlic cultivation. This study constructed a structured knowledge graph tailored to the garlic cultivation domain, thereby enhancing domain knowledge coverage; additionally, a GraphRAG retrieval framework augmented by knowledge graph paths was designed, and prompt modification techniques were applied to optimize the input context of LLMs, enabling the efficient invocation of specialized knowledge. Experimental results show that the GraphRAG-Prompt framework achieved a precision of 79.0%, a recall of 77.0%, and an F1 score of 78.0%, representing absolute improvements of 13.0, 37.0, and 28.2 percentage points, respectively, compared to the baseline LLM. The accuracy of retrieval-based question answering reached 77.0%, which is 37.5 percentage points higher than that of the baseline LLM (39.5%), validating the effectiveness of incorporating graph structures and prompt engineering into agricultural QA tasks. In particular, the proposed method demonstrates greater robustness and interpretability in the generation quality of complex knowledge nodes such as domain-specific terminology recognition and pests and diseases management. Since it operates without dependence on external APIs, it meets practical needs for localized deployment by agricultural research institutions or ground station models, thereby providing a reference pathway for domestic development of agricultural LLMs.

Future directions include automated KG construction to reduce manual load, incorporation of multimodal data (images, sensor streams) to better reflect field conditions, and multi-hop, multi-turn reasoning.

## Data Availability

The datasets presented in this study can be found in online repositories. The names of the repository/repositories and accession number(s) can be found in the article/**Supplementary Material**.
