# Supplementary material for: Knowledge-enhanced large language model construction for garlic cultivation using GraphRAG
Source: Front Plant Sci. 2026 Aug 18;17:1912355. doi: 10.3389/fpls.2026.1912355 (PMC13529709; doi:10.3389/fpls.2026.1912355)
Supplement: Supplementary file 1 [file Supplementaryfile1.pdf]

大蒜的起源：大蒜又名胡蒜，起源于中亚。最早在古埃及、古罗马和古希腊等地中海沿岸国家栽培，汉代张骞出使西域,通过“丝绸之路”将大蒜引入陕西关中地区,以后遍及全国。
大蒜的营养价值：蒜头中的碳水化合物、蛋白质、磷、多种维生素以及蛋白质等多种人体需要的营养物的含量在大宗蔬菜中是比较高的,并含有人体必需的多种氨基酸，且新鲜蒜头中微量元素硒的含量在蔬菜中是最高的，具有抗氧化功能,被认为有防癌作用。
大蒜的医疗保健作用：大蒜是广谱杀菌素,对危害人或畜、禽的多种病原菌有抑杀作用。大蒜能通过降低血液中胆固醇浓度,延缓血管硬化等途径使动脉粥样硬化程度减轻,控制高血压，预防心血管疾病。大蒜能预防糖尿病，且具有防癌抗癌的作用。
大蒜预防心血管疾病的理：据研究，大蒜能降低血液中胆固醇浓度，延缓血管硬化,增加心肌收缩能力，扩张末梢血管，使动脉粥样硬化程度减轻，控制高血压，预防心血管疾病。
大蒜预防糖尿病的机理：据研究，大蒜可减少血液中糖的含量，保护肝功能,提高血液中胰岛素含量,预防糖尿病。
大蒜防癌抗癌的机理：据研究，大蒜对乳腺癌、结肠癌、膀胱癌有预防作用。大蒜有较强的抗氧化作用，可有效清除活性氧自由基，保护生物膜结构的完整，防止细胞和组织的癌变。且大蒜还有阻断霉菌对亚硝胺合成的促进作用，减少因亚硝胺诱发癌症的可能性。
大蒜素：大蒜素又称蒜辣素，英文译名为阿利辛(Allicin)，是一种含硫化合物。大蒜中至少含有 100 种含硫化合物，其中最重要的起抗菌作用的含硫化合物就是大蒜素。
大蒜所属科目及形态：大蒜属百合科葱属 1~2 年生草本植物。通常不结种子，用蒜瓣进行无性繁殖。一株完整的大蒜植株包括：根、鳞茎、叶鞘、叶身、花茎、总苞及气生鳞茎。
大蒜根的形态与适宜的生长环境：大蒜的根是从蒜瓣基部的茎盘上发生的，为弦线状须根，称为不定根，没有主根。主要根系分布在 5~25 厘米深的土层中，横向分布范围约 30 厘米，属浅根性蔬菜。须根上的根毛少，吸水力弱,所以喜湿、喜肥，不耐旱。
大蒜全生育期的发根情况：大蒜的全生育期有 2 次发根高峰：第一次在发芽期，发根数为 20~30 条；第二次在“退母”后，发根数为 50~80 条。1 棵成龄植株的发根数为 100 条左右。蒜薹采收以后，根系不再增长，并逐渐衰亡。
大蒜的鳞茎：大蒜的鳞茎就是通常所称的蒜头，植物学名词是鳞茎，构成鳞茎的各个蒜瓣，植物学名词叫鳞芽。
大蒜的鳞芽：鳞芽是由大蒜植株叶片叶腋处的侧芽发育而成，所以又称“鳞腋芽”。鳞芽由 2~3 层鳞片和 1 个幼芽构成。外面 1~2 层鳞片起保护作用，保护鳞片或保护叶。最内一层是贮藏养分的部分,称贮藏鳞片或贮藏叶。
蒜薹和气生鳞茎的介绍：大蒜鳞茎盘顶部的生长锥分化为花芽后，逐步发育抽生蒜薹。蒜薹的下部为花茎，顶端的生花苞称总苞。总苞成熟后开裂，可以看到许多小的鳞茎，称气生鳞茎、空中鳞茎，俗称蒜珠或天蒜。
大蒜类型的划分方法：国内外有关大蒜类型的划分方法很不统一，归纳起来主要有以下 6 种分法：按蒜瓣外皮的颜色划分，按蒜瓣大小划分，按叶形及叶的质地划分，按有无蒜薹划分，按生态特性划分，按成熟期早晚划分。
大蒜品种生态型类型：樊治成、陆帼一等(1994)将引自北纬 22°~45°、东经 77°~127°的 73 个大蒜品种划分为 3 个大的生态型，分别是：低温反应敏感型、低温反应迟钝型及低温反应中间型。
金堂早蒜简介：金堂早蒜是低温反应敏感型大蒜，四川省金堂县地方品种。因主要产于云顶山，又名云顶早蒜。蒜薹和蒜头产量较低，但早熟性好，属极早熟品种，生长期约 180 天。蒜薹和蒜头品质俱佳，加之上市早，所以产值高。
软叶蒜简介：软叶蒜是低温反应敏感型大蒜，四川省成都市郊区、新都、彭县为主要产区。又名新都大蒜，是作蒜苗栽培的理想品种。该品种是典型的半抽薹品种，适应性强,不易退

化。
金山火蒜简介：金山火蒜是低温反应敏感型大蒜，广东省开平县一带的地方品种，为广东中部地区大蒜的代表品种。蒜农先将蒜头在田间晾干，然后运至库中用烟熏，待烘干后远销东南亚国家及香港特区，故称火蒜。当地一般于 10 月上旬播种，翌年 3 月上中旬收获蒜头，生育期 140~150 天。
新会火蒜简介：新会火蒜是低温反应敏感型大蒜，广东省新会县地方品种。在当地可抽薹。
普宁大蒜简介：普宁大蒜是低温反应敏感型大蒜，广东省普宁县地方品种，为广东省东部优良品种。在当地可抽薹。
忠信大蒜简介：忠信大蒜是低温反应敏感型大蒜，广东省韶关地区地方品种，为广东北部传统主栽品种。在当地不抽薹。
二水早简介：二水早是低温反应中间型大蒜，四川成都市郊彭县地方品种。当地于 8 月份播种，翌年 3 月上旬收获蒜薹，4 月上旬收获蒜头。属中早熟品种，生长期 210 天左右。耐寒性较金堂早蒜强，耐热，抗病力较强，不早衰，适应性强。
彭县蒜简介：彭县蒜是低温反应中间型大蒜，四川省成都市郊彭县地方品种。有早熟、中熟和晚熟 3 个品种。蒜薹质脆嫩，味香甜，上市早，产量高。是目前比较理想的、主要用作蒜薹栽培的优良品种。
温江红七星简介：温江红七星是低温反应中间型大蒜，四川省成都郊县地方品种。又名硬叶子、刀六瓣。属中熟品种，生育期 230 天左右。当地于 9 月中下旬播种，翌年 4 月上旬收获蒜薹，5 月上旬收蒜头。
嘉定蒜简介：嘉定蒜是低温反应中间型大蒜，上海市嘉定县名优大蒜品种。嘉定县是我国大蒜出口历史长、出口量大的大蒜生产基地。嘉定蒜有嘉定 1 号和嘉定 2 号 2 个品种。
太仓白蒜简介:太仓白蒜是低温反应中间型大蒜,江苏省太仓县地方品种。出口东南亚国家。当地于 9 月下旬播种，每 667 平方米 3 万株，翌年 4 月下旬采收蒜薹，5 月下旬采收蒜头。每 667 平方米产蒜薹 300 千克左右，蒜头 700 千克左右。
来安大蒜简介：来安大蒜是低温反应中间型大蒜，安徽省来安县相官镇地方品种，又名来安薹蒜,是当地蒜薹栽培优良品种。抽薹性很好，抽薹率可 100%。薹生长整齐，可一次采收完毕。当地薹用大蒜的适宜播种期为 9 月下旬至 10 月上旬。蒜头耐贮藏，在室温下可贮藏至翌年 2 月份。
苍山大蒜简介：苍山大蒜是低温反应中间型大蒜，山东省苍山县地方品种，为山东省传统名特蔬菜之一。其特点是蒜头大，色洁白，瓣少而大，味香浓，蒜汁黏稠，蒜薹粗而长，蒜头和蒜薹产量高，适应性强，在国内外颇负盛名。
苍山大蒜的三个品种及简介：苍山大蒜包括蒲棵蒜，糙蒜和高脚子蒜三个品种。蒲棵蒜是目前苍山县蒜区种植面积最大的秋播品种，占苍山县大蒜种植面积的 90%以上。生育期 240 天左右,属中晚熟品种。耐寒性较强；糙蒜比蒲棵蒜早熟,生育期 230~235 天。耐寒性较蒲棵蒜差，后期有早衰现象。适宜作地膜覆盖栽培；高脚子蒜的蒜薹和蒜头产量在 3 个品种中是最高的,适宜作丰产栽培。本品种为晚熟品种，生育期 240 多天，适应性强，较耐寒。
苏联红皮蒜简介：苏联红皮蒜是低温反应中间型大蒜，山东农业大学园艺系已故李家文教授 1957 年从苏联库班蔬菜研究所引进，现已大面积推广，成为目前我国大蒜出口及内销的重要品种之一。生育期 260 天左右。休眠期短，不耐贮藏。该品种的适应性较强，在山东、山西、河南、陕西、江苏等秋播地区种植，普遍表现其丰产特性。
苏联红皮蒜中定向选育而成的新品种：从苏联红皮蒜中定向选育而成的新品种有：山东的“鲁农大蒜”、河南的“宋城大蒜”、江苏的“徐州白蒜”。
鲁农大蒜简介：鲁农大蒜是低温反应中间型大蒜，山东农业大学从苏联红皮大蒜中定向选育而成。植株长势强,株高 80 厘米左右，株形开张。休眠期短，播种后出苗快，苗期生长快，

可利用中、小瓣进行密植作蒜苗栽培。
宋城大蒜简介：宋城大蒜是低温反应中间型大蒜，据原生龙报道，宋城大蒜又称围蒜，系河南农民从“苏联二号”大蒜中挑选优株，经多年选育而成。因主要在河南开封、中牟一带栽培，故称“宋城大蒜”。主要用作蒜头栽培，也可作冬前早熟蒜苗栽培。
徐州白蒜简介：徐州白蒜是低温反应中间型大蒜，江苏省徐州地区的大蒜品种。因蒜头大,内层皮色洁白，商品性好,深受国际市场欢迎，成为该地区出口的主要农副产品之一。生育期 260 天左右。不发生外层型二次生长，只有轻度的内层型二次生长发生。
早薹蒜二号简介：早薹蒜二号是低温反应中间型大蒜，山东农业大学园艺系和西北农业大学园艺系选育的大蒜品种，1997 年通过山东省品种审定委员会认定。该品种的最大特点是抽薹早、抽薹率高，蒜薹产量高。
嘉祥大蒜简介：嘉祥大蒜是低温反应中间型大蒜，山东省嘉祥县地方品种，为当地出口的传统名土特产品种。当地于 9 月下旬至 10 月上旬播种，翌年 5 月中旬采收蒜薹，6 月上旬采收蒜头，生育期 250 天左右。蒜头耐贮藏，在室温下存放时，一般到翌年 3 月才开始发芽。
襄樊红蒜简介：襄樊红蒜是低温反应中间型大蒜，湖北省襄樊市郊区地方品种。经襄樊市蔬菜研究所多年选择，成为以采收蒜薹为主的蒜薹和蒜头兼用优良品种。抽薹率 98%左右，蒜薹长 48 厘米，粗 0.8 厘米，单薹重 13 克左右。
上高大蒜简介：上高大蒜是低温反应中间型大蒜，江西省著名大蒜地方品种。耐涝、耐寒，较早熟，生育期 210 天。当地作蒜苗栽培时,于 8 月中旬至 9 月上旬播种，11 月至翌年 2 月采收，每 667 平方米产 2000~2500 千克。
都昌大蒜简介：都昌大蒜是低温反应中间型大蒜，江西省都昌县地方品种。较耐寒，为当地薹、瓣兼用的优良品种。当地于 9 月中下旬播种，翌年 3 月下旬至 4 月上旬采收蒜薹，每 667 平方米产 400 千克左右；4 月下旬采收蒜头，每 667 平方米产 500 千克左右。
四月蒜简介：四月蒜是低温反应中间型大蒜，湖南省隆回县地方品种。抽薹率达 100%，蒜薹粗实。在当地为晚熟品种，5 月上旬收蒜薹，5 月底至 6 月上旬采收蒜头。
茶陵蒜简介：茶陵蒜是低温反应中间型大蒜，湖南省茶陵县地方品种，是湖南省大蒜栽培面积最大的品种,属紫皮蒜。香辣味浓，品质好，在当地为中熟品种。
广西紫皮简介：广西紫皮是低温反应中间型大蒜，广西壮族自治区南宁市郊地方品种。抽薹性好，抽薹率 90%以上。蒜薹长 32 厘米，粗 0.74 厘米，单薹重 13 克。
舒城大蒜简介：舒城大蒜是低温反应中间型大蒜，安徽省舒城县地方品种。当地于 9 月下旬播种，翌年 4 月下旬至 5 月上旬采收蒜薹，5 月下旬采收蒜头，生育期 240 天。蒜薹行销东北一些大城市，蒜头和脱水蒜片远销日本及印度、泰国、新加坡等东南亚国家。
余姚白蒜简介：余姚白蒜是低温反应中间型大蒜，浙江省余姚县地方品种。当地于 9 月下旬至 10 月上旬播种，每 667 平方米 2.5 万~3 万株。蒜头、速冻蒜薹、脱水蒜薹远销日本及东南亚各国，糖醋蒜头、腌渍蒜头行销国内一些大城市。
陆良蒜简介：陆良蒜是低温反应中间型大蒜，云南省陆良县地方品种。抽薹率 94%以上，蒜薹长 29 厘米，粗 0.8 厘米，平均单薹重 13 克。
毕节蒜简介：毕节蒜是低温反应中间型大蒜，贵州省毕节县地方品种。大蒜产区位于贵州西北部云贵高原东部丘陵地带，主要种植在海拔 1400~1700 米地段。抽薹率 98%~100%，蒜薹长 55 厘米，粗 0.9 厘米，平均单薹重 15 克。当地于 8 月中下旬播种，翌年 5 月下旬采收蒜薹，6 月下旬采收蒜头。
桐梓红蒜简介：桐梓红蒜是低温反应中间型大蒜，贵州省桐梓县地方品种。植株长势强，株形开张。耐寒性强。除适宜作以蒜薹为主的栽培外，因叶片宽大，苗期生长快，还适宜作蒜苗栽培。
蔡家坡红皮蒜简介：蔡家坡红皮蒜是低温反应中间型大蒜，蔡家坡红皮蒜又名火蒜。陕西省

岐山县蔡家坡镇地方品种，是该县驰名省内外的大蒜良种。抽薹性好，蒜薹粗而长，长约 45 厘米，粗约 0.8 厘米，抽薹率 100%,抽薹期较整齐，上市早,品质佳。该品种主要用作早蒜薹栽培，同时因生长快、叶片肥大、假茎粗而长，还适宜作早蒜苗(越冬蒜苗)栽培。

兴平白皮简介：兴平白皮是低温反应中间型大蒜，陕西省兴平市地方品种。植株生长势强。当地于 9 月下旬播种，翌年 5 月下旬采收蒜薹，6 月中下旬采收蒜头。为晚熟品种，多用于加工成糖醋蒜、白玉蒜(咸蒜肉)销往国内部分省、市及出口日本。辣味浓，品质好，晚熟，耐贮藏。

普陀大蒜简介：普陀大蒜是低温反应中间型大蒜，陕西省洋县普陀地方品种。抽薹性好，抽薹率 99%。薹长 46 厘米，粗 0.8 厘米，单薹重 19 克，是以蒜薹栽培为主的优良品种。

耀县红皮简介：耀县红皮是低温反应中间型大蒜，又名耀县火蒜，陕西省耀县地方品种。抽薹性好，抽薹率 100%。薹长 46 厘米，薹粗 0.8 厘米，单薹重 17.3 克。为蒜薹和蒜头俱佳的品种。当地于 9 月中旬播种，翌年 5 月上旬采收蒜薹，6 月上旬采收蒜头。

天津六瓣红简介：天津六瓣红是低温反应中间型大蒜，天津市宝坻县地方品种。当地于 3 月上旬播种，翌年 5 月下旬采收蒜薹，6 月下旬采收蒜头。该品种产区位于北纬 39°以北的平川地带，其特性介于低温反应中间型与低温反应迟钝型之间，在当地系春播品种。

吉木萨尔白皮简介：吉木萨尔白皮是低温反应迟钝型大蒜，新疆维吾尔自治区吉木萨尔县地方品种。因蒜头大、蒜瓣肥、皮色洁白、品质优良、产量高、耐贮藏而享誉国内外，为出口创汇的重要品种。抽薹率 95%以上，但蒜薹短而细。当地于 4 月中旬播种，7 月中旬至 8 月上旬采收蒜薹，9 月上旬采收蒜头。生育期 150 天，为晚熟品种。耐贮藏。

伊宁红皮简介：伊宁红皮是低温反应迟钝型大蒜，新疆维吾尔自治区伊犁州伊宁县地方品种。抽薹率虽然可达 100%，但蒜薹短而细，产量不高。当地于 9 月中旬至 10 月中旬播种，翌年 5 月下旬至 6 月中旬采收蒜薹。生育期 285 天左右。蒜头耐贮藏性不如吉木萨尔白皮蒜，在当地可贮藏至翌年 3~4 月份。

昭苏六瓣蒜简介：昭苏六瓣蒜是低温反应迟钝型大蒜，新疆维吾尔自治区昭苏县地方品种。当地于 10 月 10 日至 10 月 20 日播种，播种过早或过晚，越冬时易受冻害。翌年 7 月中旬采收蒜薹，8 月中旬采收蒜头，生育期 320 天左右。耐寒性强，耐贮藏，可存放至翌年 5 月份。辛辣味浓，蒜泥可存放数天不变质。

格尔木红皮简介：格尔木红皮是低温反应迟钝型大蒜，青海省格尔木市郊地方品种。抽薹性较差，多数不抽薹，蒜头中央形成 1 个蒜瓣，少数为半抽薹。当地为春播品种，9 月份收获蒜头。

江孜红皮蒜简介：江孜红皮蒜是低温反应迟钝型大蒜，西藏自治区江孜县地方品种。当地于 4 月上旬播种，9 月上旬收获蒜头。

拉萨紫皮蒜简介：拉萨紫皮蒜是低温反应迟钝型大蒜，西藏自治区拉萨市郊地方品种。当地一般于 3 月上中旬播种，7 月上中旬采收蒜薹，10 月下旬至 11 月上旬采收蒜头。

下察隅大蒜简介：下察隅大蒜是低温反应迟钝型大蒜，西藏自治区下察隅地区地方品种。蒜头近圆形，横径 7.7 厘米,外皮紫红色。当地于 8~9 月份播种，翌年 6 月下旬至 7 月上旬收获蒜头。

拉萨白皮蒜简介：拉萨白皮蒜是低温反应迟钝型大蒜，西藏自治区拉萨市郊地方品种。蒜头扁圆形，大而整齐，外皮白色。耐寒、耐旱，抽薹率低。当地可实行春、秋两季栽培，3 月上中旬或 10 月上中旬播种，8 月下旬至 9 月上旬收获蒜头。

清涧紫皮蒜简介：清涧紫皮蒜是低温反应迟钝型大蒜，陕西省北部清涧县地方品种。当地于 3 月份播种，6 月上旬采收蒜薹，7 月上旬采收蒜头，为早熟品种。每 667 平方米产蒜薹 90~100 千克，蒜头约 800 千克。

榆林白皮蒜简介：榆林白皮蒜是低温反应迟钝型大蒜，陕西省北部榆林地区地方品种。蒜薹

短小。当地为春播,晚熟,较耐寒,耐瘠薄,多在沟台旱地栽培。
阿城紫皮蒜简介：阿城紫皮蒜是低温反应迟钝型大蒜，黑龙江省阿城传统名优地方品种。抽薹率 90%以上，但蒜薹产量低。当地于 4 月份播种，7 月中旬采收蒜头，生育期 100 天左右，为当地的早熟大蒜品种。每 667 平方米产蒜头约 600 千克。
白皮狗牙蒜简介：白皮狗牙蒜是低温反应迟钝型大蒜，吉林省郑家屯地方品种。抽薹率低,蒜薹细小，无商品价值。当地于 3 月下旬播种，7 月下旬至 8 月下旬采收蒜头，每 667 平方米产 600~750 千克。多做蒜苗生产用或腌渍用。
开原大蒜简介：开原大蒜是低温反应迟钝型大蒜，辽宁省开原县地方品种。株高 89 厘米，株幅 34 厘米。假茎高 34 厘米，粗 1.4 厘米。当地于 3 月下旬播种，6 月中旬采收蒜头。
海城大蒜简介：海城大蒜是低温反应迟钝型大蒜，辽宁省海城市郊耿庄地方品种,又名耿庄大蒜。品种行销黑龙江、吉林、内蒙古等地，还远销加拿大、罗马尼亚及日本等国。当地于 3 月中旬播种，6 月上旬采收蒜薹，7 月上旬采收蒜头。每 667 平方米产蒜薹 100 千克,蒜头 1000 千克。
应县大蒜简介：应县大蒜是低温反应迟钝型大蒜，山西省北部应县地方品种，名产地为小石口村。产品除销往大同市和雁北地区各县外，还销往内蒙古、河北等地，并出口日本。有紫皮和白皮两种，以紫皮为主。当地于 3 月下旬至 4 月上旬播种，6 月下旬至 7 月上旬采收蒜薹，7 月下旬至 8 月上旬采收蒜头。
民乐大蒜简介：民乐大蒜是低温反应迟钝型大蒜，甘肃省民乐县地方品种。株高 78 厘米，株幅 34 厘米。假茎高 12 厘米，粗 1.2 厘米。当地于 4 月上旬播种，7 月中旬采收蒜薹，8 月下旬采收蒜头。
临洮白蒜简介：临洮白蒜是低温反应迟钝型大蒜，甘肃省临洮县地方品种。株高 95 厘米，株幅 24 厘米。假茎高 41 厘米，粗 1.1 厘米。当地于 3 月上中旬播种，7 月中下旬收获蒜头。抽薹性较差，蒜薹短而细。主要用作蒜苗栽培。
临洮红蒜简介：临洮红蒜是低温反应迟钝型大蒜，甘肃省临洮县地方品种。株高 73 厘米，株幅 28 厘米。假茎高 21 厘米，粗 1.5 厘米。在产地可以抽薹。
土城大瓣简介：土城大瓣是低温反应迟钝型大蒜，内蒙古自治区乌兰察布盟和林格尔县土城子乡地方品种。株高 75 厘米，株幅 30 厘米。假茎高 15 厘米，粗 1.1 厘米。当地春播夏收，可抽薹。
银川紫皮简介：银川紫皮是低温反应迟钝型大蒜，宁夏回族自治区银川市郊县地方品种。株高 65 厘米，株幅 25 厘米。假茎高 17 厘米，粗 1.7 厘米。当地春播夏收，抽薹性较差，薹细小，而且有半抽薹现象。
大蒜的生育周期：大蒜从用蒜瓣播种到收获蒜头，重新获得新的蒜瓣，进入休眠状态称为一个生育周期。
大蒜生育周期经历的过程：完成一个生育周期需要顺序经历出苗期、幼苗期、花芽及鳞芽分化期、花茎伸长期、鳞茎肥大期和休眠期。
大蒜生育期的影响因素：大蒜生育期的长短因地区和栽培季节的不同而有很大差异。且品种的熟性不同，生育期也有差异。另外，生育期的长短还和播种期有密切关系。
大蒜的出苗期：从播种至初生叶伸出地面并展开为出苗期。出苗期的长短因播期、品种及土壤湿度的不同而有很大差异，少者 7 天左右，多者近 30 天。
大蒜蒜瓣发芽的适宜温度：蒜瓣发芽的适宜温度为 18℃~20℃。
土壤的湿度对大蒜出苗期的影响：,土壤湿度对出苗期的长短有影响。播种后如土壤湿度低，蒜瓣的茎盘上只发生少数短而细的须根，吸水力弱，发芽叶出土缓慢而且细弱，使出苗期加长，出苗也不整齐，甚至有些蒜瓣在土壤中霉烂，丧失发芽能力，造成缺苗断垄。播种后土壤湿度过大，也会产生同样后果。

播种所用蒜瓣的大小对大蒜生长发育状况的影响：在出苗期中，蒜瓣基部的茎盘上发生弦线状须根，蒜瓣发芽叶中的生长锥继续分化新的幼叶。发根和长叶所需的养分主要来源于蒜瓣中贮藏的养分。
大蒜的幼苗期：从初生叶展开到生长锥停止分化幼叶而分化为花芽、总叶片数不再增加时，为幼苗期。
秋播大蒜中的早熟品种：如金堂早、五凤蒜、二水早、彭县早熟等。花芽于 11 月中旬开始分化，鳞芽于 11 月下旬至 12 月上旬开始分化。
秋播大蒜中的中早熟品种：如彭县中熟、蔡家坡红皮，花芽分别于 12 月下旬和翌年 1 月上旬开始分化，鳞芽分别于 1 月下旬和 3 月上旬开始分化。
秋播大蒜中的晚熟品种：如兴平白皮，花芽于 4 月上旬开始分化，鳞芽于 4 月下旬开始分化。
大蒜在幼苗期的生长过程：幼苗期大蒜生长主要依靠种瓣中贮藏的养分。在此期内，根系继续扩展，生长锥继续分化幼叶，幼叶逐渐长大，露出土面，进行光合作用，制造养分。
退母：在幼苗期，种瓣中贮存的养分被幼苗吸收利用后，逐渐萎缩干瘪，最后只剩下膜状蒜衣，生产上称之为“退母”或“烂母”。
黄尖：在退母过程中，如果土壤缺肥缺水，易产生养分供应不足所造成的叶色变淡、叶尖变黄干枯现象，俗称“黄尖”或“干尖”。这种现象持续的时间愈长，幼苗生长愈缓慢，所以要在烂母前及时追肥灌水。
花芽及鳞芽分化期：从花芽和鳞芽分化开始到分化结束为花芽和鳞芽分化期。
花芽分化状态的分级：花芽分化状态分级如下:0 级——苗端生长锥扁平，陆续分化叶原基；1 级——生长锥呈半圆球形，停止分化叶原基，即将分化花芽；2 级——生长锥伸长，高出叶鞘基部，周缘长出总苞，花芽分化开始；3 级——生长锥继续伸长，明显变肥大并呈凸凹不平状；4 级——生长锥出现许多小突起；5 级——生长锥上的小突起分化为空中鳞茎及小花,花芽分化结束。
鳞芽分化状态的分级：0 级——叶腋中无鳞芽原基；1 级——一般在终止叶下方的叶腋中出现球形鳞芽原基，鳞芽分化开始；2 级——鳞芽原基上分化出 1 片幼叶；3 级——鳞芽原基上分化出 2 片幼叶。
大蒜分化指数的计算：计算分化指数，以确定分化开始期和分化结束期。分化指数计算公式如下:花芽分化指数=Σ(级别×株数)/(5×调查总株数)；鳞芽分化指数=Σ(级别×株数)/(3×调查总株数)。
花茎伸长期：从花芽分化结束到蒜薹采收为花茎伸长期。
鳞茎肥大期：从鳞芽分化结束到鳞茎成熟为鳞茎肥大期。
秋播大蒜不同成熟期品种的花茎伸长期：秋播大蒜品种，一般需要 13~20 天，其中的早熟品种，如金堂早、二水早、彭县早熟等的花芽分化结束期处于早春温度较低的时期，花茎伸长缓慢,所以花茎伸长期较长，一般需要 40 天左右。
春播大蒜品种的花茎伸长期：春播大蒜品种，一般需要 30~35 天。
秋播大蒜不同成熟期品种的鳞茎肥大期：秋播大蒜品种中的早熟品种一般需要 70 天左右，中熟品种需要 40~46 天，晚熟品种需要 35 天左右。早熟品种鳞芽分化结束期多为 3 月初，中熟品种鳞芽分化结束期多为 4 月中下旬，晚熟品种鳞芽分化结束期多为 5 月上旬，但其鳞茎成熟期分别为 5 月中旬、6 月上旬和 6 月下旬，所以秋播大蒜品种鳞茎肥大期所需天数以早熟品种最多，中熟品种次之，晚熟品种最少。
春播大蒜品种的鳞茎肥大期：春播大蒜品种中的早熟品种鳞茎肥大期一般为 35~40 天，晚熟品种为 20~25 天。
大蒜的休眠期：大蒜鳞茎成熟后即进入休眠状态，这时即使给予适宜的温度和水分，鳞芽也

不会萌芽和发根，这段时间称为大蒜的生理休眠。通过生理休眠期以后，在适宜的温、湿度条件下就可以萌芽和发根。
强迫休眠：人为控制其萌发条件，可使大蒜的鳞芽继续休眠，称强迫休眠。
秋播大蒜不同成熟期品种的休眠期：秋播蒜中的早熟品种生理休眠期较长，中熟品种次之，晚熟品种较短的情况，与品种的耐贮性之间并没有必然的联系。
蒜头产量计算公式：蒜头的单位面积产量=株数×蒜头平均重×商品蒜头百分率(%)。
大蒜各个时期生长的适宜温度：大蒜是喜好冷凉气候的蔬菜,通过休眠期的蒜瓣，在 3℃~5℃的低温下便可开始萌发。萌发的适宜温度 16℃~20℃。幼苗期的适宜温度为 12℃~16℃。大蒜花芽和鳞芽的分化都需要低温，花茎伸长和鳞茎膨大的适温为 15℃~20℃。
光照对不同生态型品种大蒜的影响：低温反应敏感型品种,光照时间长短对花茎发育的影响不大；低温反应中间型品种，在 12 小时光照下，花茎发育良好；在 8 小时光照下,花茎发育不良，鳞茎在 13~14 小时光照下发育良好；低温反应迟钝型品种,花茎发育需要 13 小时以上的光照。在 12 小时光照下一般不形成鳞茎，鳞茎发育需要 14 小时以上的光照。
光照过强对大蒜的影响：光照过强时,叶绿体解体，叶组织加速衰老,叶片和叶鞘枯黄,鳞茎提早形成。
光照过弱对大蒜的影响：光照过弱时，叶肉组织不发达，叶片黄化。
大蒜各个时期生长的适宜的土壤湿度：播种后的萌发期要求较高的土壤湿度,促进发根和发芽；幼苗期要适当降低土壤湿度；花茎伸长和鳞茎膨大期是大蒜生长日趋旺盛的时期,要求较高的土壤湿度。
春播地区的大蒜为什么增强土壤湿度：在春播地区常遇春旱，土壤水分蒸发快，地面容易返碱，腐蚀蒜种,这时如土壤湿度低，幼苗生长缓慢，而且叶片易产生黄尖现象，所以要根据当年气候情况灵活掌握。
大蒜生长过程对土壤的要求：大蒜由于根系吸收力较弱，对土壤肥力的要求较高，适宜在富含有机质、透气性好、保水、排水性能好的砂质壤土或壤土中栽培。此外，还需注意选择地势较高、地下水位较低的地段栽培大蒜。大蒜喜微酸性土壤，适宜的土壤氢离子浓度约为 1000 纳摩/升(pH 值为 6)。
大蒜生长过程对肥料的要求：大蒜对富含腐殖质的有机肥反应良好，增产效果显著。全生育期吸收的氮最多，钾次之，磷最少。
大蒜不同生长时期对肥料的要求：大蒜出苗期和幼苗期的生长主要靠种瓣中贮藏的养分，从土壤中吸收的氮、磷、钾量很少，所以在使用基肥的基础上一般不需要再施种肥。进入花茎伸长期后，叶片和蒜薹的生长加快,吸收的氮、磷、钾迅速增加，至鳞茎肥大中期达最高峰，所以花茎伸长期是追肥的关键时期。追肥的种类以氮肥为主。鳞茎膨大后期, 叶片逐渐枯黄，根系老化，吸收力减弱，吸肥量减少，一般不再追肥。
大蒜的栽培季节：大蒜的栽培季节因气候条件和品种特性而异。按气候条件可划分为秋播和春播两大区。
大蒜的秋播及适宜地区：北纬 35°以南的地区，冬季平均最低温一般在-6℃以上，大蒜可以在露地越冬,多为秋季播种，翌年初夏收获。秋播区主要包括华南、华中、河南、山东禹城以南、陕西关中及陕南、山西临汾以南及河北省南部。
大蒜的春播及适宜地区：北纬 35°以北的地区,冬季平均最低气温一般在-10℃以下，大蒜在露地不能安全越冬,多实行春播，当年夏季收获。春播区主要包括陕西省北部、山西省临汾以北、河北省北部、甘肃、宁夏、青海、新疆、吉林、辽宁、黑龙江、内蒙古及西藏。
大蒜秋播的益处：秋播大蒜幼苗的生长期长，有充分的低温期可以满足花芽和鳞芽分化的需要，而且在高温长日照季节来临以前，幼苗可以充分生长，为蒜薹和蒜头的增长积累丰富的营养，所以抽薹率高，蒜薹产量高。

大蒜栽培时的茬口安排：大蒜忌连作。连年在同一块地里种大蒜，或与葱蒜类蔬菜(大葱、洋葱、韭菜等)重茬，则病虫害严重，出苗率低，植株细弱，叶片发黄，蒜薹和蒜头产量降低。
葱蒜类蔬菜不宜重茬的原因：第一重茬时容易引起某种营养元素的缺乏，造成土壤中养分不平衡,使土壤生产力降低。第二重茬时易引起土壤中相同病原菌的大量繁殖和积累，使病害愈来愈严重。第三重茬时虫口密度增加，危害的严重程度也随之增加。第四植株间通风透光良好，为杂草的滋生提供有利环境。
秋播蒜的前茬蔬菜选择：秋播蒜的前茬以小麦、大麦、玉米、高粱、瓜类、豆类、马铃薯、早熟番茄、早熟茄子及甘蓝类蔬菜较好。在陕西关中地区，因前茬作物的不同，有“靠茬蒜”和“回茬蒜”之分。
春播蒜的前茬蔬菜选择：春播蒜的前茬以豆类、瓜类及茄果类蔬菜较好，因为这些蔬菜的施肥量较多，土壤较肥沃，对大蒜生长有利。
大蒜的后茬蔬菜选择：秋播蒜和春播蒜的后茬多为夏秋菜。可以种植夏秋番茄、黄瓜等果菜以及大白菜、萝卜等秋菜。
轮作：轮作是在一定年限内，在同一块土地上，按预定顺序轮换栽种不同性质作物的栽培制度。
大蒜秋播地区的轮作制度：大蒜秋播地区的轮作制度有水旱作物轮作和旱地作物轮作 2 种类型。据彭怀远(1993)报道，安徽省来安县大蒜产区，薹用大蒜的主要栽培制度是实行蒜与水稻或绿肥轮作。
大蒜秋播地区的轮作的具体做法：大蒜秋播地区的轮作制度有水旱作物轮作和旱地作物轮作 2 种类型。具体做法是：第一年头一季栽水稻，水稻收割后种大蒜；第二年大蒜收获后再栽水稻，水稻收割后种绿肥或油菜；第三年绿肥或油菜收获后栽水稻，水稻收割后再种大蒜。
大蒜施轮作制度的益处：第一，水、旱作物轮作，土壤干、湿交替，可以增加土壤的透气性，使土壤微生物活动旺盛，改进土壤理化性质；第二，可以减轻水稻病害和地下害虫(金针虫、蝼蛄等)的危害。第三，蒜稻轮作可以减轻稻田杂草和蒜田杂草的危害。
旱地秋播大蒜的轮作制度：旱地秋播大蒜的轮作制度可以这样安排：第一年春季栽种马铃薯、甘薯、早黄瓜或西葫芦等，收获后种大蒜，也可以在小麦收割后种大蒜；第二年大蒜收获后种大白菜或栽秋冬甘蓝、菜花;第三年春种植黄瓜、番茄、西葫芦、菜豆等夏菜，夏菜收获后再种大蒜;第四年夏，大蒜收获后种玉米。
大蒜春播地区轮作方式：大蒜春播地区轮作方式有：第一年春季种马铃薯、西葫芦、黄瓜或菜豆，收获后种大白菜，大白菜收获后土地休闲；第二年春播大蒜，大蒜收获后栽甘蓝、菜花、芹菜或播种胡萝卜、萝卜等；第三年再种夏菜。
间作：间作是指两种或两种以上作物，同时隔畦、隔行或隔株有规则栽种的种植方式。
套种：套种是指在一种作物的生育后期，于株间或行间栽种另一种作物的种植方式。
大蒜间作的目的：间作时，主作与副作的共生期较长，其主要目的是利用主作与副作对环境条件需求方面的某些差异，达到相互有利、共同发展的目的。
大蒜套种的目的：套种时，主作与副作的共生期较短，其主要目的是高度利用空间和时间，增加复种指数，以提高产量和收益。
大蒜间作和套种的原因：秋播蒜区，生产蒜薹和蒜头的大蒜,生长期长达 7~8 个月，如果单一种植，在同一块地里种了大蒜就不能种越冬蔬菜、棉花或晚(秋)玉米。加之大蒜苗期生长缓慢，生长量小，叶片狭长，直立生长，绿叶面积小，不能充分利用照射到田间的太阳光能，而且根系分布范围小，土壤中的水分和养分也得不到充分利用。所以实施间作和套种。
我国农民创造的间作套种方式和方法：粮、蒜套种，棉、蒜套种，菜、蒜套种，粮、棉蒜套种，棉、粮、蒜、菜套种，粮、菜、蒜套种,棉、蒜、瓜套种及棉、蒜、瓜、菜套种等方式。
夏玉米套冬蒜苗：选用适宜作蒜苗栽培的中早熟品种，播种时在两行玉米之间的空畦内，玉

米于 9 月中下旬收获，这时蒜苗正进入旺盛生长时期,应抓紧灌水追肥。11 月中下旬至 12 月上旬采收蒜苗，也可延长到春节供应。

早蒜苗套种小麦：选用适于作蒜苗栽培的早熟大蒜品种，经催芽处理后于 8 月上旬播种,，10 月上旬将蒜苗行间细锄一遍，然后撒播小麦种，播后锄地将麦种掩埋。12 月份前采收蒜苗。蒜苗收净后将小麦镇压 1 遍，随后灌水。

小麦、大蒜、玉米、大豆套种：10 月上旬播种小麦和大蒜，翌年 6 月上旬大蒜和小麦相继收获后抢时整地，按 354 厘米宽划分种植带，于 6 月中旬同时播种早熟玉米和大豆。玉米和大豆之间的距离为 48 厘米。玉米和大豆同在 9 月下旬收获。

秋播蒜区的棉、蒜套种：山东省棉区棉、蒜套种多采用高低畦及宽窄行套种方法。在低畦中种蒜，在高畦上种棉。大蒜于 9 月中下旬播种，翌年 4 月间在采收蒜薹前灌攻薹水，并为棉花畦造墒。棉花 4 月下旬播种。5 月下旬至 6 月上旬采收蒜头后,按照棉花需要进行田间管理。

棉、蒜套种的优点：大蒜与棉花的共生期为 1 个月左右，相互间无不利影响；棉花生长在高畦上，而且是宽窄行种植，可充分利用边行效应，通风透光良好，植株生长健壮，所以棉花产量不受影响，还增加了一季蒜的收入。

春播蒜区的棉、蒜套种：早春地表化冻 3 厘米左右时筑大蒜畦。春暖后在大蒜畦的畦埂两侧点播棉花。4~5 月份大蒜进入生长盛期时,棉苗尚小，相互间影响不大。6~7 月间大蒜收获后，棉花进入生长盛期。

大蒜套菠菜的套种：早熟大蒜品种于 8 月中旬播种，中、晚熟大蒜品种于 9 月中旬播种。播后耙平畦面，将菠菜种子埋入土中。早熟大蒜地套的菠菜于 11 月份开始间拔上市，小苗可以留在地里越冬，翌年 3 月上中旬全部采收。中、晚熟大蒜品种套种的菠菜于翌年 2 月份开始间拔上市，3 月下旬采收完。

大蒜套生姜的套种：按东西向筑畦，畦宽 1 米。10 月上旬播蒜，实行宽窄行播种。翌年 4 月下旬在宽行中种生姜。

大蒜套生姜套种的优点:这种套种方式对生姜苗期生长有利，因为生姜苗期不耐强光，栽种在大蒜植株的行间，省去了生姜栽培中插姜草遮荫的田间作业，小气候比较阴凉湿润,姜苗生长良好。待生姜进入旺盛生长时期，需要较强的光照时，大蒜已收获，姜与蒜的共生期不长，相互间无不利影响。

早蒜苗(冬蒜苗)畦埂套冬菠菜：蔡家坡红皮蒜于 7 月下旬潮蒜，8 月中旬按行距 13 厘米开沟点播，株距 5 厘米。在畦埂上撒播菠菜籽，然后整理畦埂兼给菠菜覆土。以后的田间管理统一进行。蒜苗于 11 月份至翌年 2 月上旬采收，菠菜于 12 月份至翌年 1 月份采收。

早蒜苗畦埂套冬莴：蔡家坡红皮蒜于 7 月中旬潮蒜，8 月中旬播种。按行距 13 厘米开沟点蒜，株距 5 厘米。莴笋(尖叶白笋)于 8 月上旬播种育苗，9 月中旬定植在蒜苗的畦埂上。莴笋于 11 月中下旬采收，蒜苗于 11 月至翌年 2 月上旬采收。

大蒜畦埂套种春萝卜：蒜按行距 20~23 厘米、株距 10 厘米播种。翌年 3 月下旬至 4 月上旬在畦埂上点播春萝卜(热萝卜)种子。大蒜于 4 月下旬至 5 月上旬采收蒜薹, 5 月下旬挖蒜头。畦埂上的春萝卜于 5 月下旬至 6 月上旬采收。

大蒜套种芋头：这种套种方式适用于南方水田。4 月上旬在大蒜抽薹前，按行距 67~83 厘米、株距 33~50 厘米，在大蒜行间打穴，点播芋头种薯。5 月间收获蒜头后，按照芋头的需要进行田间管理。

大蒜、黄瓜、菜豆套种：施足基肥，精细整地后做高畦，选用苍山大蒜中的糙蒜品种，于 10 月上旬播种。,播种后覆土,平畦面后浇水，然后覆盖宽 90 厘米的地膜。以后按常规方法管理。6 月上旬当大蒜即将收获前，将有机肥施入畦沟内，锄松混匀，搂平。蒜头收获后在畦上播种黄瓜，黄瓜播种后 40 天便可开始采摘。6 月下旬在两架黄瓜之间做两条垄，垄距 30

厘米，按穴距 20 厘米播种菜，9 月上旬开始采收。
蒜、莴笋、豇豆、小白菜套种：莴笋于 10 月下旬至 12 月上旬在阳畦中播种育苗,或者于翌年 1 月中下旬在温室中播种育苗，5 月中下旬至 6 月上旬莴笋收获完毕后，在原垄沟中点播夏豇豆，7 月上旬在豇豆架下撒播小白菜。7 月下旬至 8 月下旬陆续采摘豇豆。小白菜于 8 月份采收完毕后还可以播种根茬菠菜(越冬菠菜)。
夏玉米、棉花、大蒜套种：小麦收割后，按行距 67~73 厘米、株距 23~33 厘米点播夏玉米，玉米在培土后行间按行距 13~17 厘米开沟，沟深约 7 厘米，再按 10~13 厘米株距摆蒜种。播蒜后，根据玉米生育的需要灌水追肥。10 月上旬收获玉米棒后，深挖畦埂并加以修整。翌年 4 月中下旬在原来种玉米的畦埂上播种棉花。4 月下旬至 5 月上旬采收蒜薹，5 月下旬至 6 月上旬收获蒜头，9~10 月份采摘棉花。
夏玉米、棉花、大蒜套种的优点：这种套种方式的优点是：大蒜根系浅,分布范围窄，植株矮小直立，而且其生长期处于玉米生育后期和棉花苗期，所以对玉米和棉花的生育没有不良影响，又多收一茬蒜。
绿豆、棉花、大蒜套种：4 月中旬在畦埂上点播 2 行绿豆。棉花于 4 月上旬播种育苗，5 月中旬定植在预留的棉花畦中。棉花于 4 月上旬播种育苗，5 月中旬定植在预留的棉花畦中。9 月底至 10 月上旬在预留的大蒜畦中播种 4 行大蒜。绿豆于 6 月中旬收获，棉花于 9~10 月份收获。翌年 4 月下旬收获蒜薹，5 月下旬收获蒜头。
棉、粮、蒜、菜套种：秋分(9 月下旬)时,按行距 20 厘米条播 4 行小麦，按行距 17 厘米、株距 10 厘米点播 4 行蒜。小麦行与大蒜行之间的距离为 25 厘米。大蒜行中撒播菠菜。4 行大蒜中的两个边行在采收蒜苗后，于 5 月中旬各移栽 1 行棉花，中间 2 行采收蒜头。6 月上旬小麦和大蒜收获后加强棉田管理。
小麦、南瓜、夏玉米、蒜套种：10 月上旬条播小麦，4 月上旬在日光温室中用营养钵或纸筒育南瓜苗。4 月下旬将南瓜苗定植在预留的瓜沟中，6 月上旬收割小麦后浅犁，给瓜行两侧筑畦埂,然后在原小麦带中播种 2 行玉米。8 月上旬采收老南瓜，10 月上旬玉米收获后又可播种小麦。11 月份至翌年 2 月份采收冬蒜苗。
瓜类、春玉米、蒜套种：瓜类选用笋瓜或南瓜，于 3 月中旬在阳畦(冷床)中育苗，4 月中旬定植到瓜沟中,4 月上旬在延畦中点播玉米。8 月中旬在玉米垄间播种事先经过潮蒜的蒜种。玉米于 9 月中下旬收完,将玉米秆齐地面砍掉，在垄上又可栽青菜。青菜于 8 月下旬育苗，9 月中下旬移栽，11 月下旬至 12 月中下旬冬蒜苗和青菜收获完毕后冬闲。
棉、蒜、瓜套种：瓜类选用西瓜，西瓜选用早熟品种，于翌年 3 月下旬在塑料薄膜拱棚中用营养钵育苗，4 月下旬定植到预留的西瓜畦的中间，5 月上旬收蒜薹，6 月上旬收蒜头。然后在西瓜行间栽 4 行棉花苗。
棉、蒜、瓜套种的优点：这种套种方法的优点是：西瓜定植后不久就采收蒜薹，6 月上旬收获蒜头后，西瓜进入抽蔓期，大蒜和西瓜之间互不影；;西瓜和棉花的共生期约 2 个月，棉花进入生长盛期时西瓜已拔蔓，所以二者之间没有大的矛盾。
棉、蒜、瓜、菜套种：宽 220 厘米划分为一个种植带。其中 120 厘米于 9 月份撒播青菜;另 100 厘米栽 6 行蒜。翌年早春青菜收获完毕后施基肥、整地。4 月下旬在靠近大蒜行的一侧各栽 1 行西瓜，5 月中旬在西瓜行间栽 2 行棉花。6 月间大蒜收获完毕，8 月间西瓜拔秧后,地里只留下棉花。
大蒜的栽培技术：大蒜以露地栽培为主，生产蒜头、蒜薹和蒜苗(青蒜)。冬春低温期也可在保护地中栽培，生产蒜苗和蒜黄,以调节淡季蔬菜供应。
大蒜的营养与药用价值：大蒜富含维生素、氨基酸、大蒜素等，具有抗菌、助消化、防癌等功效。蒜头、蒜薹、蒜苗均可食用，蒜头还可用于医药和食品加工。硒含量高，抗氧化性强，对预防癌症和心血管疾病有益。药用上可治疗感冒、消炎，外用促进血液循环。

大蒜的植物学特性：大蒜为百合科葱属植物，根为弦线状须根，茎为短缩茎盘，叶呈披针形。鳞茎（蒜头）由蒜瓣组成，蒜薹为花茎。生长分为萌芽期、幼苗期、花芽分化期、抽薹期、鳞茎膨大期和休眠期，全生育期因播种季节而异。
大蒜的品种类型：大蒜按皮色分为白皮蒜和紫皮蒜；按蒜瓣大小分为大瓣种和小瓣种；按生态特性分为低温敏感型、中间型和迟钝型。常见品种有苍山大蒜、嘉定蒜、蔡家坡红皮蒜等，各地应根据气候和用途选择适宜品种。
大蒜的栽培条件：大蒜喜冷凉气候，适宜温度为 12～25℃，光照充足，耐旱怕涝。土壤以肥沃、排水良好的沙壤土为佳，pH 值 6 左右。忌连作，需轮作 2～3 年。种瓣大小影响产量，大瓣种更优。
秋播大蒜栽培技术：秋播大蒜在 9～10 月播种，幼苗越冬，次年 5～6 月收获。选择抗寒品种，整地施肥后作畦播种，密度每亩 4 万～5 万株。田间管理包括越冬保护、返青追肥、抽薹期水肥管理，采薹后促进蒜头膨大。
春播大蒜栽培技术：春播大蒜在 3～4 月播种，生长期短，需早播以通过春化阶段。选用红皮或早熟品种，整地后垄作或畦作，密度稍密。苗期加强中耕，退母期追肥，抽薹和鳞茎膨大期保证水肥供应。
青蒜栽培技术：青蒜以幼嫩茎叶为食，露地或设施栽培均可。选用大瓣品种，浸种催芽后密植，保持土壤湿润，追施氮肥。多次间拔采收或一次性收割，亩产可达 2000 千克。
蒜黄栽培技术：蒜黄为无光照软化栽培产品，选用白皮蒜种，密植于畦床或架床，覆盖遮光。温度控制在 12～25℃，保持湿度，20～25 天可收割，亩产 300～500 千克。
独头蒜栽培技术：独头蒜因晚播或小瓣种形成，选用小蒜瓣，延迟播种。整地后密植，管理同常规大蒜，采收期稍晚。独头蒜适合加工，亩产约 300 千克。
大蒜间作套种技术：大蒜可与玉米、小麦、棉花、西瓜等作物套种，提高土地利用率。例如蒜棉套种，大蒜秋播，棉花春播，共生期错峰管理，实现双丰收。套种需注意水肥协调和病虫害防治。
大蒜栽培前整地施基肥：距大蒜播种还有 1 个多月至 3 个月的时间，土壤有一段夏季休闲的机会。前作物收获后立即浅耕(10~13 厘米)灭茬,然后深耕(17~20 厘米)。准备做畦前,施基肥，每 667 平方米施腐熟圈粪 5000 千克左右、过磷酸钙 50 千克及棉籽饼或豆饼 100 千克,三者混匀后撒施。
大蒜栽培时种瓣的选择：种瓣的选择要从蒜头收获后在田间即开始进行。从收获的大蒜植株中选择符合本品种特征、叶片无病斑、蒜头外皮色泽一致、蒜头肥大圆整、外层蒜瓣大小均匀的单株留种,单独贮藏。
大蒜栽培时种瓣的处理：播种以前,种瓣的处理方法包括以下内容：去茎踵，温水浸种，药剂浸种。
大蒜栽培时种瓣去茎踵的目的：蒜瓣基部的干燥茎盘(茎踵)影响吸水,妨碍新根的发生，在掰瓣和选择蒜种的同时最好将茎踵剥掉,以利发根出苗。
大蒜栽培时种瓣温水浸种的目的：经过浸种的蒜瓣，茎踵被泡烂并吸足了水分，播种后可比用干蒜瓣播种的提早 5~7 天出苗，而且蒜头的收获期可提早 8~9 天。但是,经过浸种处理的蒜瓣只宜湿播，不宜干播。
大蒜栽培时种瓣药剂浸种的目的：药剂浸种可促进根系生长，使蒜苗健壮，产量增高，而且可以有效抑制蒜衣内、外部病菌的滋生和蔓延，减少烂瓣,提高出苗率。
秋播地区的大蒜在播种过早的坏处：播种过早，由于温度高，蒜瓣发芽慢，出苗期长，幼苗在冬前生长过旺,抗寒力下降，生长后期下部叶片枯黄，植株早衰，从而减弱大蒜鳞茎肥大期的光合作用和养分的积累，使蒜头变小。
秋播地区的大蒜在播种过晚的坏处：播种过晚，越冬时苗小，积累的养分少，抗寒力下降，

全生育期特别是营养生长期(茎、叶生长期)缩短,叶片数减少，叶面积也较小，但蒜头成熟期与早播的相同或略晚，所以蒜头小，产量低,蒜瓣数减少，甚至于出现独头蒜，而且抽薹率降低，蒜薹产量也低,经济效益不高。
,秋播大蒜的适宜播种期：从气候条件来说，秋播大蒜的适宜播种期一般为日平均温度20℃~22℃，北方地区多在 9 月中下旬，长江流域多在 9 月下旬至 10 月中旬。
确定适宜播期的基本原则：确定适宜播期的基本原则有两条：一是满足种瓣萌发所需的适宜温度(16℃~20℃)；二是越冬期具有 5~7 片展叶，可以安全越冬。
采用地膜覆盖栽培的大蒜的适宜播种期：采用地膜覆盖栽培的大蒜，适宜播期应较不盖地膜的晚 5~10 天。否则因地温高，出苗慢，种瓣易在土中腐烂。
以生产蒜薹为主时大蒜的播种：以生产蒜薹为主时，为了解决蒜薹集中上市价格低的问题，多采用早、中、晚熟品种配套，排开播种、分期上市的办法。
春播大蒜的适宜播种期：当上层土壤化，,日平均气温稳定上升至 3℃~5℃，达到大蒜发芽所需低温界限时，便可以播种。适期早播可以延长生育期,提高产量。
春播地区的大蒜在播种过早的坏处：春播地区的大蒜可以适期早播，但也不可过早，因为蒜瓣在-2℃以下的低温下容易受冻，丧失发芽力，造成大量缺苗，而且容易发生“马尾蒜”。
春播地区的大蒜在播种过晚的坏处：播种过迟，植株生长势弱，生育期缩短，在高温、长日照条件下容易形成独头蒜、少瓣蒜或无薹分瓣蒜。
春播地区的大蒜适宜改变播种期：春播地区的大蒜由于生育期短，抽薹率低，蒜薹发育不良，产量低，甚至形不成产量，而且上市迟，所以要靠从外地贩运蒜薹，以供应市场需求。有的地方采用改变播期、加强配套措施的办法，初步达到提早蒜薹上市期和提高蒜薹产量的目的。
大蒜的种植密度：大蒜的种植密度应根据当地的综合条件，通过田间试验确定。地上部生长旺盛、株形开张、生育期长、蒜头大的品种，需要较大的空间和营养面积，可适当种稀些；株形较直立、生育期较短、蒜头较小的品种，可适当种密些。
不同等级的蒜瓣播种时种植密度不相同的原因：用不同等级的蒜瓣播种时，种植密度不应相同。大蒜瓣所含营养物质较丰富，播种后根系发达，植株生长旺盛，假茎较粗，叶片数较多，单株叶面积较大，蒜头较大，产量也较高，所以需要较大的空间和营养面积，可适当种稀些。小瓣蒜播种则应适当密植。
地膜覆盖栽培的大蒜的种植密度：地膜覆盖栽培的大蒜,植株生长旺盛，应种稀一点，每 667 平方米 3 万株左右。
大蒜种植密度对大蒜的影响：大蒜的种植密度不但影响蒜薹和蒜头产量，而且对品质也有影响。密度太高时，蒜头变小，蒜瓣平均重量下降，小蒜瓣比例增多，单位面积产量虽然有可能提高，但蒜头和蒜瓣品质下降。密度太低时，蒜头增大，蒜瓣平均单重增加。但由于单位面积的株数减少，单位面积产量随之下降。
大蒜的播种方法：目前我国大蒜基本上都采用手工点种，虽然费工，但播种位置和方位比较准确。
大蒜播种时的注意事项：播种时应注意蒜瓣的方位和播种深度，一般都采用趁墒播种。有时采用先干播后浇水的方法，具体播种方法也因做畦方式的不同而不同。
大蒜播种时的种瓣方位：由于大蒜叶生长的方向与蒜瓣背腹线的方向垂直，所以在播种时要求将蒜瓣背腹线的方向与播种行向一致，这样出苗后蒜叶就整齐一致地向行间伸展生长。为了使大蒜生长期间能更好地接受阳光，应尽量采用南北畦向,定方位播种。
大蒜播种时的播种深度：大蒜适于浅播，正如“深葱浅蒜”的农谚所说。一般播种深度以 3~5 厘米为宜。播种过浅易“跳蒜”，出苗时根系将蒜瓣顶出地面；播种过深出苗晚且弱，不利于以后蒜头膨大，产量低。
春播蒜和秋播蒜适宜的播种深度：春季土表温度高，所以春播蒜宜适当浅播，而秋播蒜可适

当深播。
大蒜水肥一体化意义：通过按需供水供肥，提高水肥利用率，减少浪费，提升大蒜产量与品质，同时降低病虫害发生率，实现资源高效利用。
大蒜栽培时膜下滴灌原则：采用膜下滴管带技术，灵活调节水肥供应量，按大蒜生长需求精准分配，减少蒸发与流失。
大蒜栽培时底肥与追肥结合：施足有机底肥，根据生育阶段补充速效化肥，确保养分持续供给，满足大蒜各阶段需求。
大蒜栽培时以有机肥为主：优先施用无公害有机肥，辅以适量速效化肥，增强土壤肥力，减少化学肥料对土壤的负面影响。
大蒜栽培时全元施肥策略：以氮、磷、钾为主，搭配微量元素（如钙、镁等），实现全面营养供给，避免单一元素缺乏，从而使大蒜生长出现问题。
大蒜栽培时土壤肥力提升：逐步增加土壤有机质含量，限制速效化肥用量，改善土壤结构，促进大蒜根系健康生长。
大蒜需氮量：每生产 1000 公斤蒜头需纯氮约 15 公斤，氮肥利用率 25%-30%，主要用于根茎叶生长期。
大蒜栽培时磷肥需求与利用：每产千公斤蒜需五氧化二磷 4.5-5.5 公斤，利用率仅 10%-15%，重点用于鳞芽分化阶段。
大蒜栽培时钾肥需求特点：每产千公斤蒜需氧化钾约 12 公斤，利用率 30%-35%，蒜头膨大期需钾量显著增加。
大蒜栽培时发芽期营养来源：发芽期依赖母体营养，无需额外施肥，可维持至 5 片叶左右生长。
大蒜栽培时苗期需肥占比：越冬前需总肥量的 8%-12%，以氮肥为主，促进幼苗健壮。
大蒜播种时点种方法：因做畦方式不同而不同，有平畦播种、高垄播种、地膜覆盖畦播种等。
大蒜播种时平畦播种：平畦播种有开沟点播和打孔点播 2 种方法，最常用的是开沟点播法。开沟法就是从畦的一侧按计划行距，用角锄或耧子逐条开 5~6 厘米深的浅沟，在沟内按计划株距整齐一致地摆蒜，播后顺手覆土，整畦播完后再用耙子适当镇压，并顺地表搂平。打孔法就是按计划株行距，用“蒜踏”打孔播种。
大蒜播种时高垄播种：高垄播种有先做垄后播种和先播种后做垄 2 种方法。先做垄后播种，即按整地时做的垄，在垄上按行距开 2 条沟，沟内按株距点播后覆土。先播后做垄，即在整地时只将地面整平不做垄，播种时先按宽、窄行开沟，按株距将种瓣摆在沟中，然后在宽行的两侧取土覆盖蒜种，做成高垄，则原来的宽行变成了垄沟，原来的窄行变成了高垄。
大蒜播种时地膜覆盖畦播种：采用地膜覆盖栽培时，无论是高畦还是平畦，一般有 2 种播种方法：一种是先播种、后盖膜，是常用的方法；另一种是先盖膜、后打孔播种。
大蒜需要田间管理的原因：大蒜各生育期有其自身的规律性和对环境条件的不同要求，生产者必须根据不同生长发育时期的特点，进行田间管理工作。
大蒜出苗期的田间管理：在此期间田间管理的中心任务是保证土壤中有充足的水分和氧气，为蒜瓣的萌发出土创造条件，达到早出苗、苗全、苗齐的目的。土壤干燥的，播种后立即灌水使蒜种与土壤密接，并供给萌芽所需水分。
秋播大蒜幼苗期的田间管理：秋播大蒜的幼苗期一般是在冬季度过的，田间管理的中心任务是培育壮苗，确保幼苗安全越冬。
秋播大蒜幼苗期的田间管理的措施：措施是：幼苗长出 2~3 片叶后，施提苗肥，每 667 平方米追施尿素 15 千克或碳酸氢铵 30 千克。碳酸氢铵不稳定，易挥发，应开沟施在沟内，边施边盖土。施肥后随即灌水，然后合墒中耕松土，蹲苗，使根系下扎,并防止过早烂母。土壤封冻前，在灌越冬水后，施“腊肥”，每 667 平方米施畜杂肥 2000 千克左右，然后中耕，并

向根部浅培土。
春播大蒜幼苗期的田间管理：春播大蒜幼苗期的田间管理的中心任务是防止提早烂母。措施是及时锄地，破除土壤板结，疏松土壤，提高地温。春播地区蒜农称此期为蹲苗期，田间管理以锄地为主。
春播地区蒜农锄地的原则：春播地区蒜农称此期为蹲苗期，田间管理以锄地为主。锄地的原则是："头遍深,二遍跟，三遍四遍不伤根"。当幼苗有 2~3 片展叶时锄头遍，这时是根系向下扎，横向分布范围小，深锄不会伤根，同时可疏松土壤，提高地温，有利于根系发展和去除杂草。
秋播大蒜花茎伸长期的田间管理：大蒜花茎伸长期田间管理的重点是抓紧追肥和灌水，以满足花茎生长的需要，并为蒜头的肥大打下基础，防止蒜头肥大期缺肥及植株早衰。
秋播大蒜花茎伸长期田间管理的措施：措施是，当蒜薹"露尾"(总苞尖端伸出叶鞘)时，施"催薹肥"，结合灌水每 667 平方米施尿素 20~25 千克。以后土壤要经常保持湿润状态。采收蒜薹前 3~4 天停止灌水，以免蒜薹太脆，采收时易折断。
春播大蒜花茎伸长期的田间管理：春播大蒜花芽分化结束后，温度呈持续上升趋势，所以花茎伸长较快。另外，由于鳞芽的分化结束期与花芽分化结束期相近，在花茎伸长加快时，鳞芽的增长也加快，当蒜薹露尾时蒜头已开始膨大，需水需肥量大，更要抓紧追肥和灌水。
大蒜鳞茎肥大期的田间管理：大蒜在这一时期的管理目标是，防止早衰，尽量延长叶片寿命和根系寿命，促进蒜头肥大。
大蒜鳞茎肥大期田间管理的措施：具体管理措施是，在蒜薹采收后,及时灌催头水，以后用小水勤灌，保持土壤湿润,降低地温,促进蒜头肥大。
蒜薹收获的适宜时期：在蒜薹(不包括总苞部分)高出最后一片叶的叶鞘口 7 厘米左右、上部尚未弯曲时采收。也可在蒜薹高出最后一片叶的叶鞘口 15 厘米左右、上部向下弯曲("打弯")时采收。
蒜薹采收过早的坏处：蒜薹采收过早不仅蒜薹产量低，而且残留在假茎中的花薹继续生长，消耗养分，对蒜头肥大不利。
蒜薹采收过晚的坏处：蒜薹采收过晚，总苞外皮已变白，蒜薹纤维增多，品质下降，而且蒜薹不易抽出，必须划破假茎，对蒜头的肥大也有不利影响。
采收蒜薹的方法：通常,采收蒜薹的方法有以下 4 种：拉抽法，夹抽法，扎抽法和划抽法。
拉抽法采收蒜薹的益处：拉抽法效率高、省工，对假茎和叶及蒜薹的损伤小，采下的蒜薹适宜贮藏，也有利于蒜头的进一步肥大。
适宜采用拉抽法采收的蒜薹种类：这种采薹法适用于假茎口较松("口松")、蒜薹细的早熟品种，或播期晚、蒜薹细小的大蒜。如苍山大蒜的蒜薹比较容易拉抽。
拉抽法采收蒜薹的具体措施：措施：一手捏住蒜薹上部距假茎口 3~5 厘米处，斜向往上拉直蒜薹，用力要均匀，待蒜薹在假茎中断裂，发出轻微响声时，迅速抽出蒜薹。
夹抽法采收蒜薹的具体措施：措施：采薹时一只手捏住薹的上部，另一只手用夹子将地面 2~3 片叶间的假茎处夹一下，使蒜薹断裂而假茎不断，然后将植株上部两片叶的叶鞘口撕开，徐徐抽出蒜薹。
夹抽法采收蒜薹的益处：夹抽法的断薹率较抽薹法低，蒜薹产量较高，且蒜薹上无损伤，适合贮藏。
适宜采用夹抽法采收的蒜薹种类：这种方法一般多用于晚熟、口松的白皮蒜。
扎抽法采收蒜薹的具体措施：措施：采薹时，一只手握住总苞下部，将蒜薹拉直，另一只手拿筷子尖或针铲，向假茎离地面 5~7 厘米处横向垂直扎入，切断蒜薹，即可将蒜薹抽出。
扎抽法采收蒜薹的益处：扎抽法的优点是：蒜薹无划伤，断面齐，采薹后假茎不倒伏，叶片较完整，蒜头产量高。

适宜采用扎抽法采收的蒜薹种类：此方法适用于稀植、假茎短粗且上下部粗度差异小、采薹期较早的早熟品种。
划抽法采收蒜薹的具体措施：采薹时一只手握住总苞上部，将蒜薹拉直，另一只手将木棍的槽子对准距地面 3~4 叶处假茎无叶身的一侧(俗称"大里面")，使针尖刺入假茎内，并迅速向上划至梢部。叶鞘全部划开后再将蒜薹从基部折断。
划抽法采收蒜薹的益处：划抽法的优点是采薹速度快,不易断薹,蒜薹长,产量高。
划抽法采收蒜薹的坏处：由于采下的蒜薹上多带有一道纵向伤痕，易腐烂,不耐贮运。另外，由于采薹时叶鞘被完全划破后植株倒伏，叶片很快干枯，鳞茎肥大期叶片的光合作用被严重减弱，导致蒜头产量下降。
适宜采用划抽法采收的蒜薹种类：适用于大蒜品种的"秆高"(叶鞘长)，"口紧"(上部叶鞘的孔较细)，或蒜薹下粗上细，难以用其他方法采薹。
蒜头收获的适宜时期：当蒜叶色泽开始变为灰绿色，植株上部尚有 3~4 片绿色叶片，假茎变软，外皮干枯，蒜头茎盘周围的须根已部分萎蔫时便可采收。
蒜头收获过晚的坏处：收获太晚,全部叶鞘都变薄干枯，而且茎盘枯朽，则蒜头开裂，采收时易散瓣。
蒜头收获过早的坏处：收获太早，蒜头外面的叶鞘厚，水分多，遇阴雨天易发霉，同时由于蒜头未充分成熟，晾晒后失水多，蒜头产量降低，且不耐贮藏。
蒜头收获的具体措施：土质黏重的地区，选晴天早晨当土壤较湿润时挖蒜；土质疏松的地块可用手拔出。挖出蒜头后就地将根系剪掉。如果是扎把贮藏，则应同时剪梢，留 10~15 厘米长的叶鞘；如果是编成蒜辫贮藏，则不用剪梢。
大蒜栽培时期返青水关键点：气温回升后及时补水，缓解冬季干旱，激活根系活力，加速大蒜返青时的生长。
大蒜栽培时期抽苔期水肥需求：抽苔期需水肥齐攻，保障茎叶与蒜薹同步生长，避免脱肥早衰。
大蒜栽培时期膨大期通透水：蒜头膨大时浇透水，促进养分吸收，但需避免积水，防止烂根。
大蒜苗期控水原则：返青初期需适当控水，提高地温，促进根系下扎，增强抗逆能力。
大蒜成熟期晾地管理：临近收获时停止浇水，降低土壤湿度，加速蒜皮老化，提升耐贮性。
大蒜栽培时期水溶性肥料优势：追肥选用全水溶、无残渣肥料，提高吸收率，避免滴灌系统堵塞。
大蒜生育期配比调整：前中期以氮为主，中期增磷，后期补钾，动态匹配大蒜营养需求。
大蒜栽培时期水肥浓度控制：浓度过高易烧根，过低则无效，需按生育阶段科学配比，确保安全高效。
大蒜栽培时期均匀灌溉要点：滴灌需水量一致，避免局部积水或干旱，防止肥害或养分分布不均。
大蒜栽培时期采用水肥一体化病害防控效果：水肥一体化促进植株健壮，增强抗病性，减少叶枯病、根腐病发生率。
大蒜栽培时期需肥阶段性：发芽期无需肥，苗期少量，返青后激增，抽苔至膨大期达峰值，后期渐减。
大蒜栽培时期微量元素补充：大蒜在生长时期钙、镁、硼等可预防生理病害（如叶尖干枯），需通过叶面或水肥补充。
大蒜栽培时期有机肥长效性：腐熟有机肥缓慢释放养分，改善土壤团粒结构，为大蒜持续高产提供基础。
大蒜栽培时期速效化肥辅助：在需肥高峰期（如返青期）辅以速效氮肥，快速缓解营养短缺问题，保证大蒜能够持续健康生长。

大蒜栽培时期膜下滴灌节水：覆盖地膜减少蒸发，结合滴灌精准供水，节水率达 30%-50%。
大蒜栽培时期氮肥过量风险：过量施氮易导致徒长、延迟成熟，需按生育期控制用量，平衡营养。
大蒜栽培时期磷肥高效施用：磷易被土壤固定，宜与有机肥混施或采用集中深施，提高利用率。
大蒜栽培时期钾肥抗逆功能：增强大蒜抗寒、抗旱、抗病能力，尤其在鳞茎膨大期不可或缺。
蒜苗的栽培方式：蒜苗的栽培方式可分为露地栽培和保护地栽培两种。
可用于蒜苗栽培的优良品种应具备的条件：用于蒜苗栽培的优良品种，一般应具备以下条件：休眠期短，出苗快，苗期生长快，假茎粗而长，叶片宽大肥厚，黄叶或干尖(叶片上部干枯)现象轻。
秋播地区适宜作蒜苗栽培的品种：目前，秋播地区适宜作蒜苗栽培的品种有：软叶蒜、彭县中熟、普陀蒜、蔡家坡红皮蒜、耀县红皮蒜及陆良蒜等。
春播地区适宜作蒜苗栽培的品种：春播地区适宜作蒜苗栽培的品种有：白皮狗牙蒜、格尔木白皮蒜、阿城紫皮蒜、阿城白皮蒜、土城大蒜及土城小瓣等。
蒜种处理的原因：早蒜苗的播种期处于 7~8 月份高温季节，种瓣的休眠期尚未完全结束，田间的高温也对蒜瓣的发芽不利，如果播种前不经过特殊处理，播种后长期不发芽，待温度下降，种瓣发芽出土后,蒜苗生长也不健康。
陕西大蒜产区农民采用的“潮蒜”(或称“窖蒜”)措施：陕西大蒜产区农民采用“潮蒜”(或称“窖蒜”)措。具体做法是：播种前 20 天左右，选大小整齐、无病虫的蒜瓣，放在清水中淘洗后捞出，喷上杀菌剂，运到可保持冷凉湿润的场所，使种瓣发根(俗称“催芽”)。
蒜苗的播种时间：陕西关中地区早蒜苗从 7 月下旬至 8 月下旬可分期播种，晚蒜苗则于 9 月中下旬播种。
蒜苗的播种方法：在已整好的平畦中按行距 13 厘米开沟，沟深 12 厘米左右。将经过冷凉处理的种瓣装在容器中，上面盖上湿布，以防日晒损伤幼根。按株距 4~5 厘米将种瓣摆在沟中。第一行沟摆完后再开第二行沟，随即将沟中土覆盖在先一行已播种的沟内。如此依次进行。
蒜苗栽培的特点：播种深而密是蒜苗栽培的特点，这样可以增加假茎软化变白部分的长度，提高蒜苗产量和品质。蒜苗栽培一般每 667 平方米用种量 200~350 千克。
早蒜苗的田间管理：早蒜苗播种后，当地面略干时，用耙子碎土保墒，以利于出苗。大部分幼苗出土后轻浇一水，促进全苗。地面略干时中耕，蹲苗。待蒜苗叶片绿色加深时结束蹲苗，再随灌水施第二次追肥。以后视天气情况灌水，使土壤经常保持湿润状态。
晚蒜苗的田间管理：晚蒜苗冬前田间管理同蒜薹和蒜头栽培。为确保安全越冬，应在灌冻水后覆盖圈粪或藁草。不覆盖的则需于地冻前锄地，以防土壤裂缝，使幼苗受冻。
早蒜苗的采收：早蒜苗于 10 月份开始陆续采收至 12 月份。收获时连根挖起，去除根部泥土及下部黄叶后扎成小捆上市。一般每 667 平方米产 3000 千克左右，高产者可达 4000 千克。
晚蒜苗的采收：晚蒜苗于翌年早春便可开始采收，供应期约 2 个月。一般每 667 平方米产 3000 千克左右。
蒜苗的保护地栽培的形式：蒜苗保护地栽培的形式多样，各地也不尽相同。主要形式有地栽和床栽 2 种形式。
蒜苗的地栽：地栽即与露地蒜苗栽培方式一样，在土壤中播种蒜瓣，生长蒜苗并带根采收。
蒜苗的床栽：床栽即将整蒜头直接播入沙子、沙土或混合基质中，生长并割收蒜苗。床栽结合轻基质的使用，可做成多层架栽培。
蒜苗的保护地栽培的益处：保护地蒜苗栽培技术容易掌握，生产周期短，经济效益高，是增加冬、春市场鲜嫩细菜供应的重要方法。

温室中栽培蒜苗不同规模的不同栽培方式：在温室中栽培蒜苗，可根据生产规模的大小，采用不同的栽培方式。小规模栽培时，可利用木箱栽培，将木箱放在温室后墙部分或其他空地上；规模稍大的，可在温室内整地做畦，专门生产蒜苗；大规模生产蒜苗时，可在温室内搭架进行立体栽培。
在温室中栽培蒜苗的技术要点：在温室中栽培蒜苗的技术要点如下：栽培床准备，品种选择，播种期的选择，栽前蒜种处理，摆蒜技术，管理技术，采收技术。
温室栽培蒜苗的品种选择：温室栽培蒜苗，由于要采取高度密植的方法提高单位面积产量，所以应选择蒜头大、休眠期短、生长迅速、假茎长、不易倒伏、蒜瓣多而均匀的白皮蒜品种作种瓣。
温室栽培蒜苗的播种期选择：播种期主要根据计划上市时期确定。地栽的蒜苗一般要生长 2 个月左右，床栽的播种期可根据第一刀蒜苗的上市期向前推 1 个月左右。
温室栽培蒜苗的栽前蒜种处理：床栽的，首先将选好的蒜头表层的碎皮剥去，然后用清水浸泡 1 天，使蒜瓣充分吸水，捞出后堆积 1 天，再挖掉茎盘,抽出残存花茎，准备栽种。地栽的，将种蒜掰成蒜瓣，必要时进行浸种和低温催芽，以打破休眠和促进发芽。
温室栽培蒜苗的摆蒜技术：床栽的，将蒜头掰成两半或用整个蒜头密密地摆在栽培床上，蒜头间的空隙用散瓣填充，每平方米摆种瓣 15 千克左右。摆完后盖 3~5 厘米厚的细沙土并拍实，以防种瓣上升(“跳蒜”)，然后灌 1 次透水。灌水后再盖一层厚约 2 厘米的沙土。地栽的，播种方法与露地蒜苗相同。
保护地蒜苗的管理重点：保护地蒜苗管理的重点是温度和水分。
保护地蒜苗的温度管理：摆蒜后，白天保持 25℃~27℃,夜间 22℃~24℃，使其尽快萌发出土。出苗以后，白天保持 18℃~20℃，夜间 15℃左右，使苗子健壮生长。
保护地蒜苗的水分管理：一般除摆蒜后灌 1 次透水外，只需再灌 2 次水。蒜苗高 10 厘米左右时灌第二次水，收割前 3~5 天灌第三次水。灌水量逐次减少，灌水的水温最好在 20℃左右。
温室栽培蒜苗的采收：床栽蒜苗长到 30~35 厘米,顶部稍倾斜时即可收割。地栽蒜苗生长 2 个月左右，植株有 5~6 片叶时即可连根挖收。收割时间最好放在清晨温度低时，以防蒜苗在堆放和运输中发热腐烂。
适合栽培蒜黄的场所：栽培蒜黄的场所有：阳畦、地窖、窑洞、防空洞及温室等，可因地制宜选用。
大蒜根系需氧特性：土壤含水量过高会抑制大蒜根系呼吸，需保持适度墒情，促进气生根发育。
大蒜生育期水分管理：发芽期保墒，苗期控水，抽苔期足水，膨大期透水，成熟期停水。
大蒜栽培时水肥同步技术：将肥料溶解于灌溉水中，实现精准供应，减少人工成本与资源浪费。
大蒜返青期温度调控：结合浇水提升地温，加速根系恢复，缩短大蒜返青时间，促进早发。
大蒜抽苔期营养分配：此阶段养分优先供应蒜薹，需加强水肥管理，防止因竞争导致蒜头减产。
蒜头膨大关键期：大蒜膨大期缺水缺肥易导致蒜头小而松散，需保证水肥充足且均衡。
大蒜栽培时土壤 pH 值要求：适宜 pH 为 6.0-7.0，过酸或过碱均影响养分吸收，需定期检测调整。
大蒜滴灌系统维护：定期清洗过滤器与管道，防止堵塞，确保水肥均匀输送至每株大蒜。
大蒜栽培时的叶面肥补充：在根系吸收受限时（如低温），喷施叶面肥可快速补充中微量元素。
大蒜轮作减少连作障碍：大蒜在与非葱蒜类作物轮作时，可减少土传病害，维持土壤养分平

衡。
大蒜抽苔期的管理要点：抽苔期通常出现在 4 月上中旬，蒜苔开始分化并迅速生长。此期需及时补充水肥，促进蒜苔伸长，结合抽苔水追施 5 公斤/亩水溶肥。抽苔后需轻提蒜苔，避免损伤植株，同时为蒜头膨大期储备养分。
蒜头膨大期的关键措施：抽苔后 18-20 天进入蒜头膨大期，此时光合产物集中输送至蒜头。需保持土壤湿润，但停止追肥（根系老化无法吸收），避免积水导致腐烂。此期管理以水为主，确保蒜头充分膨大，提升单头重量。
大蒜成熟收获期的判断标准：叶片由青转黄，假茎变软且养分完全回流至蒜头时即可收获。黄淮流域多在 5 月中下旬进行，收获后需及时晾晒，避免暴晒导致蒜皮开裂，影响贮藏品质。
大蒜生育期的重叠特征：大蒜各生育期无明显分界，初期、盛期、末期存在重叠。例如返青期与抽苔期部分重叠，需根据植株生长动态调整管理措施，确保各阶段营养需求均被满足。
底肥的重要性与施用方法：底肥占大蒜总需肥量的 60%-70%，以腐熟有机肥为主，配合氮磷钾复合肥。每亩施用量为腐熟粪肥 3-5 吨+复合肥 50 公斤，深翻入土 20-30 厘米，确保养分长效释放。
返青肥的施用时机与配比：返青盛期（3 月底至 4 月初）追施高氮水溶肥（如尿素）5-8 公斤/亩，促进叶片扩展和鳞芽分化。施肥后需立即浇水，加速养分溶解吸收，避免烧根。
抽苔肥的作用与注意事项：抽苔期追肥以平衡型水溶肥为主，补充 5 公斤/亩，防止后期脱肥导致蒜苔细弱。施肥需与浇水同步，确保肥效及时发挥，但避免过量引发贪青晚熟。
蒙头水的功能与操作：播种后立即浇透蒙头水，确保土壤墒情充足，促进种子吸水萌发。水量以渗透至耕作层为宜，避免积水造成烂种，同时可压实土壤减少出苗不齐现象。
越冬水的浇灌策略：12 月浇越冬水前需确保土壤干燥，浇水量以渗透至根系层（15-20 厘米）为佳。浇水后覆盖秸秆或地膜保温，防止冻害，同时抑制杂草滋生。
返青水的浇灌条件：返青水需在地温稳定 15℃以上时进行，过早浇灌会降低地温延缓生长。结合返青肥施用，采用小水慢灌方式，避免大水漫灌导致土壤板结。
抽苔水的管理技巧：蒜苔伸长至 10-15 厘米时浇抽苔水，水量适中，以保持土壤湿润但不积水为原则。浇水后中耕松土，提高土壤透气性，促进蒜苔快速抽出。
蒜头膨大期的水分调控：蒜头膨大期需保持土壤湿度 60-70%，浇水间隔 7-10 天。收获前 10 天停止浇水，防止蒜头水分过高影响贮藏，同时促进蒜皮干燥增厚。
大蒜根腐病的症状与防治：根尖发黑腐烂，植株萎蔫死亡。病因包括连作、高湿、施肥过量。防治需轮作、增施有机肥，发病初期用恶霉灵（真菌）或铜制剂（细菌）灌根。
叶枯病的类型与综合防治：表现为叶片干枯、白斑或紫斑。防治需清除病残体、轮作，药剂选用代森锰锌或苯醚甲环唑，交替使用以避免病原菌产生抗药性。
大蒜紫斑病的识别与处理：叶片出现紫色梭形病斑，边缘黄晕。高温多湿易发，防治需加强通风，喷施咪鲜胺或醚菌酯，每隔 7-10 天喷施一次，连续 2-3 次。
大蒜锈病的发生规律与防控：叶片出现橙黄色粉状孢子堆，严重时全株枯死。防治需降低田间湿度，发病初期喷施三唑酮或嘧菌酯，及时清除病叶减少传播。
大蒜菌核病的危害与防治措施：茎基部腐烂，产生黑色菌核。连作田块高发，播前可用 50℃温水浸种 10 分钟杀菌，发病后用菌核净或异菌脲喷雾。
大蒜病毒病的传播途径与预防：表现为花叶、畸形，主要由蚜虫传播。防治需选用无毒种蒜，及时灭蚜，田间发现病株立即拔除销毁，避免扩散。
大蒜除草剂的选择与使用：播后苗前用乙氧氟草醚（封闭除草），苗后用辛酰溴苯腈（选择性除草）。施药需均匀，避免重喷或漏喷，注意避开高温时段以防药害。
大蒜封闭除草的操作要点：播种后 3 天内喷施二甲戊乐灵或恶草酮，形成药膜封闭土表。要

求土壤湿润，药液量 30-40 升/亩，确保除草效果且不影响大蒜出苗。
带棵除草剂的应用时机：苗后杂草 3-5 叶期喷施辛酰溴苯腈，定向喷雾避免药液接触大蒜叶片。施药后 6 小时内无雨可保证药效，对阔叶草和禾本科草均有效。
大蒜连作障碍的解决策略：连作导致土传病害加重，需实行 2-3 年轮作（与玉米、小麦轮换）。结合深翻晒垡、增施生物菌肥，改善土壤微生态，降低病原基数。
土壤消毒的常用方法：夏季高温期翻耕后覆盖薄膜，利用太阳能灭菌；或使用棉隆颗粒剂（20-30 公斤/亩）处理土壤，有效杀灭线虫、真菌和杂草种子。
大蒜品种选择的区域适应性：北方寒冷地区宜选耐寒品种（如苍山蒜），南方多雨区选耐湿品种（如金乡蒜）。引种前需试种观察抗逆性，避免盲目推广导致减产。
种蒜处理与消毒技术：播前剔除霉变、损伤蒜瓣，用 50%多菌灵 500 倍液浸种 30 分钟，晾干后播种。可有效预防根腐病、叶枯病等种传病害。
大蒜播种深度与覆土要求：播种深度 3-5 厘米，过浅易倒伏，过深出苗延迟。覆土后轻镇压使种子与土壤紧密接触，提高出苗率，同时减少“跳籽”现象。
地窖栽培蒜黄的技术要点：地窖栽培蒜黄的技术要点如下：建窖，品种选择，播种期选择，蒜种处理，烤窖，摆蒜，遮光软化，管理，收割。
地窖栽培蒜黄的品种选择：选择品种的标准同蒜苗保护地栽培。可以选用蒜瓣多而小的白皮蒜品种或紫皮大蒜品种。
地窖栽培蒜黄的播种期选择：秋季至翌年 2 月份都可以播种。一般可按上市期提前 25~30 天播种。
地窖栽培蒜黄的蒜种处理：同蒜苗保护地栽培。
地窖栽培蒜黄的烤窖技术：摆蒜前 10~15 天，白天在窖顶盖农膜，使窖内温度升高，晚上在农膜上盖玉米秸保温。
地窖栽培蒜黄的摆蒜技术：将经过处理的整个蒜头密摆在地窖内的畦里，空隙处用散蒜瓣填满，蒜头顶部要平齐。然后盖 3~5 厘米厚的细沙，用喷壶洒水，使床土湿润但不积水。洒水后如有蒜头露出，则进行局部覆土，使畦面平整。
地窖栽培蒜黄的遮光软化：摆蒜后在窖顶架木棍，上盖农膜，农膜上盖玉米秸，厚 30 厘米左右，然后盖土，使窖内保持黑暗、温暖、湿润状态。
地窖栽培蒜黄的管理技术：地窖内的管理工作主要是调节温度。蒜黄生长的适温范围为 15℃~25℃。摆蒜后，温度要高些，使其尽快出苗。出苗后，温度逐步降低,使蒜黄粗壮。
地窖栽培蒜黄的收割技术：摆蒜后 25~30 天，当蒜黄长到 30~40 厘米长时收割，刀口与地表平，勿伤蒜种。待新叶出土、老叶伤口愈合后洒水。第二次收获时将蒜黄连同蒜种拔出。
温室栽培蒜黄的准备工作：先在温室中挖栽培池,池深 50~60 厘米，长和宽根据温室面积决定，原则是便于覆盖、遮光和操作。池内填细沙土,厚约 10 厘米。
温室栽培蒜黄的遮光软化：待大部分蒜种的初生叶出土后，在栽培池上覆盖黑色塑料薄膜或双层草帘遮光，四周埋严。如覆盖时间晚或盖不严，则叶鞘变为绿色,品质降低。
温室栽培蒜黄的灌水技术：摆蒜后立即灌水。幼苗期需水量小，可根据室内温度及天气情况，灌少量水或不灌水。蒜黄生长盛期需水量加大，可适当增加灌水量。
温室栽培蒜黄的温度要求：摆蒜后，为促进萌芽，室内温度白天以 25℃、夜间以 18℃~20℃为宜。幼苗期，白天 20℃~22℃,夜间 16℃~18℃。以后随苗高的增长逐渐降低温度收获前 4~5 天,昼夜保持 10℃~15℃。
温室栽培蒜黄需通风换气：燃煤温室内常聚集一些有害气体，如二氧化硫、一氧化碳等,应乘中午外温高时打开天窗，进行短时间的通风换气。
温室栽培蒜黄的收割：温度适宜时，摆蒜后 20 多天、蒜黄高 30 多厘米时，便可割头刀。如果温度偏低，则需 30 多天。割后扎成小捆，放在阳光下照晒片刻，使蒜黄由淡黄色变为金

黄色。
栽培独头蒜的益处：独头蒜可以加工成糖醋蒜，如湖北荆州、沙市生产的甜酸独头蒜，颗粒圆整，质地清脆，甜酸爽口，风味独特，成为畅销国内外及东南亚各国的传统名优商品。
栽培独头蒜时的品种选择：生产独头蒜所用的种瓣必须是小蒜瓣，所以一般多从蒜瓣较多而蒜瓣较小的大蒜品种中选择。
栽培独头蒜时的种瓣的选择：生产独头蒜的种瓣大小，关系到独头蒜的产量和品质。选择大小适宜的蒜瓣作种蒜，才能获得高的独头率和大小适中的独头蒜。
大蒜品种退化的主要原因：大蒜不能正常结种子，生产上长期采用蒜瓣进行无性繁殖，从蒜瓣到蒜瓣，不经过授粉受精产生种子的有性世代，则不能产生生活力强的后代，因而易导致品种退化。
大蒜品种退化的表现：品种退化表现在：植株长势减弱，病毒病严重，蒜头和蒜瓣逐渐变小，产量逐年下降乃至丧失商品价值。
大蒜实现品种提纯复壮的主要途径：目前解决大蒜品种退化，实现品种提纯复壮的主要途径有以下 4 条：异地换种，建立蒜种生产制度，气生鳞茎繁殖和脱毒苗繁殖。
大蒜地膜覆盖栽培的优势：地膜可增温保墒、抑制杂草，促进早出苗。选用黑色地膜效果更佳，覆膜后按株距打孔播种，注意膜边压实防止风吹破损。
大蒜缺氮症状与补救：植株矮小，叶片黄化。发现后立即追施尿素 10 公斤/亩，或叶面喷施 1%尿素溶液，连续 2-3 次，间隔 7 天，快速补充氮素。
缺磷对大蒜生长的影响：根系发育不良，蒜头膨大受阻。基施过磷酸钙 50 公斤/亩，生长期喷施 0.2%磷酸二氢钾，增强光合作用，提高蒜头产量。
大蒜钾肥缺乏的识别与对策：大蒜的叶尖枯焦，蒜瓣松散。膨大期每亩追施硫酸钾 15 公斤，或叶面喷施 3%草木灰浸出液，促进碳水化合物转运，增加蒜头紧实度。
大蒜所需微量元素的补充方法：缺硼导致蒜头裂瓣，可基施硼砂 1 公斤/亩；缺锌叶片出现条纹，喷施 0.1%硫酸锌溶液。结合土壤检测针对性补充，避免盲目施用。
大蒜冻害的预防与补救：寒潮前喷施芸苔素内酯增强抗逆性，冻后及时剪除枯叶，追施速效氮肥促发新叶。严重冻害田块可改种春播大蒜，减少损失。
大蒜高温胁迫的管理措施：超过 25℃时覆盖遮阳网，早晚喷水降温。增施腐殖酸肥提高根系活力，叶面喷施硫酸锌缓解热害，延长功能叶寿命。
大蒜早薹现象的原因与防控：春化不足或氮肥过量导致早抽薹。选择适宜品种，控制苗期氮肥，抽薹初期人工摘除薹尖，促进养分回流至蒜头。
二次生长（复瓣蒜）的成因：种瓣低温处理不当或播期过早引发。解决需严格控温（2-5℃处理 30 天），适期播种，避免秋季高温延长春化时间。
大蒜分蘖的控制技术：分蘖消耗养分，降低商品性。可通过增加种植密度（3.5 万株/亩）、控氮增钾抑制分蘖，及时摘除侧芽集中营养供应主茎。
蒜薹采收的最佳时机：蒜薹弯曲呈“S”形，顶端苞片微开时采收。晴天午后进行，提薹力度均匀，避免拉断。采后及时上市或冷藏保鲜。
蒜头晾晒与贮藏方法：收获后蒜头带秧晾晒 3-5 天，编辫挂藏或去秧堆藏。贮藏环境要求通风干燥，温度 0-2℃，相对湿度 70%-75%，定期检查剔除霉变蒜头。
大蒜轮作的经济效益分析：与小麦轮作可减少病害 30%，提高土地利用率。每亩综合收益增加 20%-25%，同时降低农药使用成本，符合可持续农业要求。
生物防治技术的应用：释放蚜茧蜂防治蚜虫，施用枯草芽孢杆菌抑制根腐病菌。结合太阳能杀虫灯、性诱剂等绿色防控手段，减少化学农药残留。
秋播与春播时间选择：黄淮流域以秋播为主，10 月寒露前后种植，次年 5 月中下旬收获，生长期 210-230 天。春播适用于冬季低温低于-15℃的地区，需根据当地气候调整播期。

大蒜需肥特点：每生产 1000 公斤蒜头需吸收纯氮 28-29 斤、五氧化二磷 9-11 斤、氧化钾 26-27 斤。大蒜需肥量为粮食作物的三倍，需分阶段科学施肥，底肥占总需肥量的 60%-70%。
大蒜苗期生长特征与管理：苗期根系和叶片同步发育，温度低于 10℃时形成六叶一心或七叶一心后停止生长。需控水控肥，防止徒长，增强抗寒能力，为越冬做准备。
大蒜越冬期保护措施：12 月大雪后气温低于 10℃进入越冬期，需浇足越冬水保持土壤湿度，避免干冻损伤根系。农谚“大蒜不怕湿冻怕干冻”强调水分对安全越冬的重要性。
大蒜对土壤与肥力要求：大蒜高产需肥沃土壤，有机质含量 2%-4.5%可减少化肥使用。土壤 pH 值以 6-7.5 为宜，pH 低于 5 或高于 8.5 不宜种植。种植前需培肥地力，每亩施氮磷钾总量 120-140 斤，底肥利用率高，是产量基础。
大蒜的种植密度与产量关系：以蒜头为主的大蒜种植密度为每亩 3.1-3.5 万株，可确保亩产 3000-3500 斤以上。合理密植能优化光合作用效率，减少病虫害，是提高产量的关键因素。
大蒜换种基地的特点：这些换种基地的共同特点是：气候温和，地势较高，土质疏松肥沃含腐殖质较多，排水良好。
大蒜的换种路线：大蒜换种的路线多为平原地区向丘陵低山区换种。从保持优良种性及提高经济效益考虑，在最适生产区培育原种，由次适区繁殖大田用生产种，是大蒜产区比较合理的品种提纯复壮体系。
蒜种生产制度：完整的蒜种生产制度包括：确立选种目标、提纯复壮繁殖原种及制定原种生产田技术措施。
蒜种生产制度中以生产蒜苗为主要目的的确立选种目标：以生产蒜苗为主要目的的品种，其选种目标为：出苗早，苗期生长快，叶鞘粗而长，叶片宽而厚，质地柔嫩，株形直立，叶尖不干枯或轻微干枯。
蒜种生产制度中以生产蒜薹为主要目的的确立选种目标：以生产蒜薹为主要目的的品种，其选种目标为：抽薹早而整齐，蒜薹粗而长，纤维少，质地柔嫩，味香甜，耐贮运。
蒜种生产制度中以生产蒜头为主要目的的确立选种目标：以生产蒜头为主要目的的品种，其选种目标为：蒜头大而圆整，蒜瓣数符合原品种特征，瓣形整齐，无夹瓣，质地致密脆嫩，含水量低，黏稠度大，蒜味浓，耐贮运。
蒜种生产制度中的提纯复壮繁殖原种：最简单的提纯复壮方法是利用一次混合选择法(简称一次混选法)。每年按照既定目标，从种子田中严格选优，去杂去劣，将入植株的蒜头混合在一起。播种前再将入选蒜头中的蒜瓣按大小分级，将一级或二级蒜瓣作为大田生产用种。
蒜种生产制度中的制定大蒜原种生产田技术措施：大蒜原种生产田的栽培管理与一般生产田相比，有以下 6 个方面的特殊要求：第一，选择地势较高、地下水位较低、土质为壤土的地段作为原种生产田；第二，播种期较生产田推迟 10~15 天；第三，选择中等大小的蒜瓣作种瓣；第四，适当稀植；第五，早抽蒜薹，改进采薹技术；第六，选优、去杂、去劣工作应在原种生产田中陆续分期进行。
气生鳞茎繁殖的优点：用气生鳞茎繁殖的优点表现在以下两个方面：提高繁殖系数和后代生长势强。
气生鳞茎繁殖技术中的品种选择：气生鳞茎仅有数粒的品种，繁殖系数难以提高。气生鳞茎数目虽多，但个体太小的品种，培养成大的分瓣蒜头需要的年限较长。一般可选择单株空中鳞茎数 20~30 粒、平均单粒重在 0.3 克以上的品种。
气生鳞茎的培育：根据大田种植面积所需的蒜种数量及所用品种的气生鳞茎数和大小，建立一定面积的气生鳞茎培育圃，施足基肥。头一年在生产田中选择具有原品种典型性状的单株，采用一次混合选择法收获蒜头，从中选择一、二级蒜瓣作为培育气生鳞茎的种瓣。
气生鳞茎的收获期：气生鳞茎的收获期一般比蒜头收获期晚 10~15 天。
气生鳞茎收获过早或过晚的坏处：收获过早，气生鳞茎未充分成熟，播种后出苗率低；收获

过晚，地下的蒜头易散瓣，使蒜头产量和质量降低，而且，气生鳞茎易脱落，不便管理。
气生鳞茎繁殖在生产上未得到广泛应用的原因：气生鳞茎繁殖虽然有它的优点，但目前在生产上尚未得到广泛应用。究其原因有以下 3 个方面：一是不如异地换种简便、快捷；二是留气生鳞茎的植株没有蒜薹产量，当年收入减少；三是留气生鳞茎的植株，蒜头产量也受影响。
大蒜脱毒苗的培育：目前国内外普遍认为，将蒜瓣内幼芽的茎尖分生组织，切取 0.1~0.2 毫米，经植物组织培养获得的脱毒苗，是解决大蒜品种退化、提高大蒜产量和品质的有效途径。
大蒜脱毒苗的起源：在感染病毒病的植株体内，病毒的分布不均匀，一般在生长点部位(茎尖分生组织)不带毒，所以用茎尖分生组织进行组织培养，可以繁殖出脱毒苗(或称无毒苗)。
脱毒苗培养的过程：脱毒苗培养的过程如下：打破休眠，预脱毒，表面消毒，诱导生芽，诱导生根，移栽试管苗，繁育脱毒原原种，网室扩大繁殖脱毒一代及二代原种，种子田扩大繁殖生产种。
脱毒苗培养的过程中打破休眠的方法：如果用刚采收的新鲜鳞茎作为脱毒材料时，应选用打破休眠处理。方法是,将新鲜鳞茎置于 5℃~10℃温度下 25~30 天，以打破休眠，便于及早进行茎尖培养。
脱毒苗培养的过程中预脱毒的方法：将打破休眠后的鳞茎置于 36℃~38℃温度下 30 天左右，使病毒钝化，可提高脱毒率，产生较多的健康苗。
脱毒苗培养的过程中表面消毒的方法：将蒜瓣皮剥掉后，放在 2%次氯酸钠溶液中消毒 15 分钟，用无菌水冲洗数次，再投入 75%酒精中浸泡 20 秒钟，取出后在酒精灯上烧一下，在超净工作台内的解剖镜下，切取长 0.5~1 毫米的茎尖。
应用脱毒蒜种应注意的问题：脱毒大蒜的生产种用于大田生产后，由于蚜虫的传毒而再度感染的情况仍然严重，需要注意以下几点：第一，在脱毒大蒜田周围不能种植洋葱等葱属作物，以免交叉感染；第二，田间采用物理方法诱蚜、避蚜及喷农药灭蚜，消灭传毒媒介。第三，购买脱毒生产种时最好选购增产效果较显著的一代生产种；第四，一般每 3 年更新 1 次，重新购进一代或二代生产种；第五，增施有机肥和磷、钾肥，适当控制速效性氮肥的施用。
大蒜的主要病害：大蒜病害主要有病毒病、灰霉病、叶枯病、紫斑病、白腐病等。
大蒜的病毒病病害：大蒜病毒病是世界性病害，也是对大蒜危害性最大、发病率最高的一种病害。病毒一旦侵入植株体内，不但对当代有影响，而且鳞茎母体带毒后便以垂直传染方式传递给后代，导致种性退化。
大蒜患病毒病的症状：大蒜患病毒病的症状归纳起来有以下几种：①叶片出现黄色条纹；②叶片扭曲、开裂、折叠,叶尖干枯、萎缩；③植株矮小、瘦弱,心叶停止生长,根系发育不良,呈黄褐色；④不抽薹或抽薹后蒜薹上有明显的黄色斑块。
大蒜患病毒病的发病规律：被病毒侵染的蒜头，当年并不表现带毒症状，而是在播种后长出的幼苗上或成年植株上表现出病毒病症状，因此要及早清除田间带病毒植株。高温干旱、植株生长不良时，症状严重；水、肥充足，植株生长健壮时，症状较轻。
大蒜病毒病的防治方法：第一，采用脱毒大蒜生产种。第二，消灭大蒜植株生长期间及蒜头贮藏期间的传毒媒介。第三，大蒜田周围不要种植其他葱属作物。第四，实行 3~4 年轮作。第五，播种前严格选种。第六，从幼苗期开始，对种子田进行严格选择，及时拔除发病植株，以减少病害传播。第七，加强大蒜生产田的水、肥管理，培育健壮植株，增强抗病力。
大蒜病毒病的治疗方法：发病初期喷蓖麻油 100 倍液，或高脂膜 200 倍液，或 10%混合脂肪酸水乳剂 100 倍液，或 20%盐酸吗啉胍·铜可湿性粉剂 500~1000 倍液，或 1.5%植病灵乳油 1000 倍液。
大蒜患灰霉病的症状：大蒜灰霉病是真菌性病害，多发生在植株生长的中后期和蒜薹贮藏期。从下部老叶尖端开始发病。病斑开始为水渍状，以后变为白色至浅灰褐色。病斑扩大后成为沿叶脉扩展的梭形或椭圆形，后期连接成长条形大病斑。

大蒜灰霉病的传播途径及发病条件：大蒜灰霉病病菌主要以菌核在土壤中越过冬、夏，以后借散落在土壤表层或附着在病株残体上的灰霉(分生孢子梗和分生孢子)在田间传播蔓延。风雨、流水、工具及田间操作都可以成为病菌传播的媒介。

大蒜灰霉病的防治方法：第一，选用抗病品种。第二，选择地下水位低、土壤排水性良好的地段种植。第三，施用有机肥作基肥,增施磷、钾肥，避免过量施用氮肥及灌水，合理密植，防止植株徒长。第五，贮藏蒜薹的冷库和其中的货架，在使用前喷 1%~2%福尔马林溶液或0.3%~0.5%漂白粉溶液消毒。

大蒜灰霉病的治疗方法：田间发现中心病株后，及时拔除并喷布 40%嘧霉胺悬浮剂 800 倍液，或 50%腐霉利可湿性粉剂 1500 倍液，或 50%凯泽水分散粒剂 1000 倍液，或 40%多菌灵可湿性粉剂 600 倍液,或 50%多菌灵·乙霉威(灰即定)可湿性粉剂 600 倍液，或 70%甲基硫菌灵可湿性粉剂 800 倍液防治。上述药剂与天达 2116 混合交替使用,隔 7~8 天喷 1 次,连喷 2~3 次，效果更佳。

杂草对大蒜的危害：第一，竞争资源；第二，抑制生长；第三，杂草时病虫害宿主。

杂草与大蒜竞争资源：杂草与大蒜争夺土壤中的养分、水分和光照，导致大蒜生长受限，产量下降。尤其多年生深根杂草吸肥能力强，长期侵占资源，严重时造成绝产。

杂草抑制大蒜的生长：杂草覆盖地表会降低土壤温度，延缓大蒜出苗速度，且部分恶性杂草（如禾本科）分泌化感物质，直接抑制大蒜根系发育。

杂草是病虫害宿主：杂草为地老虎、蚜虫等害虫提供栖息地，同时可能携带病毒病、锈病等病原体，间接威胁大蒜健康。

灭生性除草剂选择：草铵膦铵盐（精草铵膦铵盐）含量 10%可一次性根除所有植物，包括乔木、灌木，药效持久，一年内基本无杂草再生。

草铵膦施用方法：每 100 毫升药液兑水 30 斤定向喷雾，需均匀覆盖杂草叶片，喷后 8-10 小时避雨，避免强光时段（如中午）施药，以免蒸发过快影响药效。

草甘膦与草铵膦区别：草甘膦遇土壤后分解，仅灭杀现有杂草，但土壤中杂草种子仍可能再生，需周期性补喷；草铵膦则通过触杀作用快速灭草，适用于需长期控草的地块。

多年生杂草处理：对深根性杂草（如芦苇），需人工连根清除或多次喷施高浓度灭生性除草剂，确保根系彻底死亡，防止再生。

施药环境要求：施药时需避开早晨露水或雨后湿润条件，以免药液被稀释；最佳时间为半下午，此时杂草气孔开放，吸收效率高。

封闭除草剂组合：二甲戊乐灵+乙氧氟草醚+噁草酮复配使用，可形成药膜层，抑制杂草种子萌发，同时对已出土杂草具有触杀作用。

均匀施药关键：喷雾时需匀速行走，避免重喷或漏喷，确保药液均匀覆盖地表。漏喷区域易成杂草“绿岛”，重喷则可能导致药害。

对大蒜施药剂量控制：按说明书严格配比，过量使用可能抑制大蒜出苗，不足则封闭效果差。例如，二甲戊乐灵推荐亩用量为 100-150 毫升。

施药后土壤湿度管理：施药后 3-5 天内保持土壤湿润，促进药膜形成；若遇干旱需浅层灌溉，但避免大水冲刷破坏药层。

阔叶杂草防治：辛酰溴苯腈可高效防除荠菜、苋菜等阔叶杂草，选择性杀灭，对大蒜安全性高，喷后 24 小时见效。

禾本科杂草防控：氯氟吡氧乙酸+吡氟酰草胺复配剂可针对狗尾草、马唐等禾本科杂草，通过干扰细胞分裂抑制生长，施药后杂草 3-5 天黄化枯萎。

大蒜施药适期：杂草 3-5 叶期为最佳防治窗口，此时杂草抗药性弱且未结籽，可最大限度减少后期再生风险。

天气对大蒜施药的影响：喷药后 8 小时内遇雨需补喷，雨水冲刷会降低药液附着力；避免高

温强光时段施药，以免药液蒸发过快或灼伤大蒜叶片。
除草剂使用注意事项：药械清洗，安全间隔期，防护措施，环境友好性。
对大蒜使用除草剂时的药械清洗：喷施不同除草剂前需彻底清洗喷雾器，防止药剂残留导致交叉污染或药害。
对大蒜使用除草剂时的安全间隔期：最后一次施药距大蒜收获需≥30 天，避免农药残留超标，确保产品符合食品安全标准。
对大蒜使用除草剂时的防护措施：施药人员需穿戴防护服、口罩及手套，避免皮肤接触药液；施药后及时清洗身体和衣物。
环境友好性：避免在邻近水源或生态敏感区使用高残留除草剂，减少对非靶标生物（如蜜蜂、鱼类）的危害。
大蒜患叶枯病的症状：叶枯病主要危害叶片和蒜薹,多从下部老叶尖端开始发病。发病初期病斑呈水渍状，叶色逐渐减退，叶面出现灰白色稍凹陷的圆形斑点。病斑扩大后变为灰黄色至灰褐色，空气湿度大时为紫黑色。病斑表面密生黑褐色霉状物，即分生孢子梗和分生孢子；最后散生小黑点，即子囊壳。病斑形状不整齐，有梭形和椭圆形。
大蒜叶枯病的病原：病原菌属半知菌亚门匐柄霉属真菌。子囊壳球形或椭圆形，黑褐色。每个子囊壳内有子囊 20~30 个。子囊黑色，长筒形或长椭圆形。子囊之间有丝状物相隔。每个子囊内含 8 个子囊孢子。
大蒜叶枯病的传播途径及发病条件：以菌丝体或子囊壳在病株残体或土壤中越冬。借风、雨、流水、种瓣、肥料、人、畜、工具等媒介传播。叶枯病病菌为弱寄生菌，生长健壮的植株不易染病。常与紫斑病同时发生。
大蒜叶枯病的防治方法：第一，加强栽培管理，培育健壮植株，增强抗病力。第二，加强田间排水及松土保墒工作，降低田间湿度，控制发病条件，田间操作时避免损伤叶片，以减少伤口。第三，及时发现病株，收集后烧毁或深埋。第四，播种前种瓣用 40℃~50℃温水浸泡 1 个半小时，预防种瓣带菌。
大蒜叶枯病的治疗方法：发现中心病株后，喷 50%甲基硫菌灵可湿性粉剂 500~600 倍液，或 75%百菌清可湿性粉剂 500 倍液，或 50%敌枯唑(叶枯灵)粉剂 1000 倍液，或 10%叶枯净(杀枯净)可湿性粉剂 1000 倍液，或 60%唑醚·代森联水分散粒剂 800 倍液，或 25%嘧菌脂胶悬剂 1500 倍液，或 10%苯醚甲环唑水分散粒剂 1500 倍液。为增强药液在叶片上的附着力，药液中可加入 0.1%的洗衣粉。
大蒜患紫斑病的症状：病菌主要危害葱蒜类蔬菜的叶部，大葱和洋葱的花梗也易染病。最初在叶或花梗上出现白色、稍凹陷的小斑点，病斑中央呈浅紫色。以后病斑很快扩大为椭圆形或纺锤形，紫色，稍凹陷。2~3 周后，病斑上产生黑色霉状物，即分生孢子，常形成同心轮纹。
大蒜紫斑病的病原：病原菌属半知菌亚门真菌。分生孢子长棒形或圆筒形，褐色或黑褐色，有 7~13 个横隔膜。分生孢子的上部有细长、无色的顶胞。
大蒜紫斑病的传播途径及发病条件：病菌以菌丝体或分生孢子附着在病株残体上及土壤中越冬，或直接以分生孢子越冬。分生孢子萌芽后，借风、雨从植株叶片的气孔或伤口入侵。阴雨多的年份发病较重。
大蒜紫斑病的防治方法：第一，播种前种瓣用 40℃~45℃温水浸泡 1.5 小时；用 50%多菌灵粉剂拌种，药剂用量为种瓣重量的 0.5%；或用 40%多菌灵胶悬剂 50 倍液浸种 4 小时，预防种瓣带菌。第二，实行 3~4 年轮作，避免与葱蒜类蔬菜连作。
大蒜患白腐病的症状：染病植株一般先从外部叶片的叶尖出现黄色或黄褐色条斑并向叶鞘及内叶发展，植株生长缓慢，较正常植株矮小，假茎变软、腐烂，后期整株发黄枯死，受害组织呈灰黑色并出现灰白色菌丝层和黑色菌核。

大蒜患白腐病的病原：病原菌属半知菌亚门小核菌属。菌核多为圆形，表面光滑，黑色，大小为 0.5~1 毫米，单生或相互重叠。菌核萌发时表面破裂，从中伸出菌丝。菌丝上生小梗。小梗上着生分生孢子。孢子圆形,透明。

大蒜白腐病的传播途径及发病条件：白腐病病原菌以菌核在土壤和病株残体内越冬，翌年春季菌核萌发产生菌丝，借雨水或灌水传播，再侵入大蒜组织，造成根系变软腐烂或组织干腐。但田间病株上的新菌核，当条件适宜时可不经休眠，继续萌发产生菌丝，形成再侵染。种瓣带菌是远距离传播的主要途径。

大蒜白腐病的防治方法：第一，避免连作，实行 3~4 年轮作。第二，选择地下水位较低、排水良好的地块种植大蒜，加强松土保墒，降低地表湿度。第三，经常进行田间检查，及时发现中心病株，在未形成菌核时连根带土挖出深埋。第四，播种前，种瓣用 15%三唑酮可湿性粉剂，或 50%甲基硫菌灵可湿性粉剂拌种，药剂用量为种瓣重量的 0.3%。

大蒜白腐病的治疗方法：田间发病初期，喷 50%腐霉利可湿性粉剂 1500~2000 倍液，或 25%多菌灵可湿性粉剂 400~500 倍液，或 50%甲基硫菌灵可湿性粉剂 600 倍液，或 20%甲基立枯磷乳油 1000 倍液，或 75%蒜叶青可湿性粉剂 1500 倍液，每 7~10 天喷 1 次。

大蒜的主要虫害：大蒜的害虫主要有蚜虫、根蛆、葱蓟马、螨类、茎线虫、大蒜粪蚊、豌豆潜叶蝇及轮紫斑跳虫。

为害大蒜的蚜虫种类：为害大蒜的蚜虫有葱蚜、桃蚜、棉蚜、豆蚜和萝卜蚜。

蚜虫危害大蒜的发生规律：蚜虫卵在蔬菜、棉花或桃树枝上越冬，也可以成虫和若蚜在温室、大棚、菜窖比较温暖的场所越冬并继续为害，靠有翅蚜迁飞扩散。温暖、干旱的气候有利于蚜虫的发生，春、秋两季为害严重，夏季高温多雨为害减轻。

蚜虫危害大蒜的防治方法：蚜虫的防治要采取农业防治、化学防治、物理防治与生物防治相结合的综合防治措施。

根蛆的形态特征：种蝇的成虫比家蝇小，体长约 6 毫米，暗褐色，头部银灰色，胸背上有 3 条褐色纵纹，全身有黑色刚毛。翅透明，翅脉黄褐色。卵长椭圆形，稍弯曲，乳白色，表面有网纹。幼虫似粪蛆，乳黄色,体长 7~9 毫米，尾端有 7 对肉质突起。

根蛆危害大蒜的发生规律：种蝇葱蝇在北方 1 年发生 3~4 代(由卵→幼虫→蛹→虫,称为 1 代)，南方 5~6 代。一般以蛹在土中或堆中越冬，成虫和幼虫也以越冬。翌年早春成虫开始大量出现，早、晚躲在土缝中，天气晴时很活跃，田间成虫数量大增。

根蛆危害大蒜的防治方法：第一，选用麦茬地,深耕晒垡。第二，有机肥必须经过高温发酵，充分腐熟后再施用。第三，施用有机肥作基肥时，要深埋入土中并与种瓣隔离。第四，播种前剔除发霉、受伤、受热、受冻的蒜瓣，以免腐烂时招致种蝇和葱蝇产卵。

根蛆幼虫危害大蒜的治疗方法：幼虫发生初期用 2.5%敌百虫粉剂撒在植株基部及周围土壤中；或用蒜蛆一遍净或 48%毒死蜱乳油 1500 倍液，或 50%辛硫磷乳油 800 倍液，装在喷雾器中，将喷头中的旋水片取出，把药液注入根部土壤中，每隔 7~10 天 1 次，连续 2~3 次。可消灭早期幼虫。

根蛆成虫危害大蒜的治疗方法：成虫发生期，喷 50%敌敌畏或 90%敌百虫 1000 倍液防治。成虫发生盛期用糖醋液诱杀成虫。糖醋液的配方为：糖 6 份、醋 3 份、白酒 1 份、水 10 份、90%敌百虫粉 1 份，装在盆中调匀，加盖，分放在田间，待晴天开盖诱杀。

葱蓟马的形态特征：成虫虫体细小，长约 1.3 毫米，宽约 0.3 毫米，翅展 1.8 毫米。体色从淡黄色至深褐色。翅细长，透明，浅褐色，翅脉黑色，翅的周缘密生细长毛。卵极小，椭圆形，一侧向内弯曲，乳白色。若虫如针尖大小，肉眼不易发现。

葱蓟马危害大蒜的发生规律：葱蓟马在华东地区 1 年发生 6~10 代，华北地区 3~4 代，华南地区达 20 多代。主要以成虫和若虫潜藏在葱、洋葱、蒜的叶内及在杂草、枯枝、落叶和土缝中越冬。翌年春季开始活动，继续为害。

葱蓟马危害大蒜的防治方法：第一，不与其他葱蒜类蔬菜连作，实行 3~4 年的轮作。第二，及时清除田间杂草及枯枝落叶，减少虫源。第三，温暖干旱季节勤灌水，抑制葱蓟马的繁殖和活动。第四，勤检查，发现虫情后，及时喷 10%吡虫啉可湿性粉剂 2500 倍液，或 2.5%辉丰菊酯乳油 2000 倍液进行防治。

螨类害虫防治方法:第一，蒜头贮藏期间如发现螨类为害时，可用硫磺粉熏蒸。第二，不与大葱、洋葱、韭菜连作，也不要毗邻种植，实行 3~4 年轮作。第三，播种前严格选种。第四，播种时先在播种沟中均匀撒入乙拌磷颗粒剂。第五，大蒜在田间生长期间用 80%敌敌畏乳剂 300~400 倍液灌根。第六，及时清除田间被害植株，烧毁或深埋，以减少螨源。

为害大蒜的茎线虫种类：为害大蒜的茎线虫有 3 种：圆葱茎线虫、马铃薯茎线虫及甘薯茎线虫。

大蒜茎线虫的防治方法：第一，选择未经大蒜茎线虫污染的土壤种植，或实行 3~4 年的轮作。第二，选择健康种瓣，掰蒜后的蒜皮、蒜根、茎盘及蒜薹残桩集中烧毁。第三，播种前进行种瓣消毒。第四，播种前每 667 平方米用 2.5%敌百虫粉剂 1.5~2 千克，加细土 30 千克，混合均匀后撒入播种沟内，然后播种。

大蒜粪蚊形态特征：雌性成虫体长 2.5~2.8 毫米，体宽 1~1.1 毫米；雄性成虫体长 2.1~2.5 毫米，体宽 0.8~1 毫米，翅展 4.5~5 毫米。全身黑色，有光泽。头小，有 1 对复眼，3 个单眼。触角短而粗，黑色。头下有短而粗的喙。

大蒜粪蚊危害大蒜的发生规律：大蒜粪蚊以蛹或老熟幼虫在土壤或被害蒜头中越冬。成虫在大蒜植株根部土壤表层内产卵，多数产卵成堆，少数散产。孵化后的幼虫聚集在大蒜的假茎基部，由外向内蛀食，破坏假茎组织，使植株萎蔫枯死。当蒜瓣形成时，幼虫则蛀食蒜瓣外的嫩皮部分，使蒜瓣变软、变褐、腐烂，瓣肉裸露，甚至引起整个蒜头腐烂。

大蒜粪蚊的防治方法：第一，避免连作，实行 3~4 年轮作。第二，春播地区于秋季深耕翻地，消灭越冬虫蛹及幼虫。第三，大蒜生长期间加强除草、松土，使植株根际周围的表土干燥，抑制虫卵孵化及幼虫活动。

大蒜粪蚊的治疗方法：成虫期喷洒 80%敌敌畏乳油 1000 倍液，幼虫期用 50%辛硫磷乳油 800 倍液灌根。

辛酰溴苯腈适用场景：针对大蒜 3-4 叶期后阔叶杂草爆发，喷药后 24 小时叶片失绿，3-5 天全株枯死。

氯氟吡氧乙酸混用禁忌：避免与有机硅助剂混用，以免增加渗透性导致大蒜心叶药害。

禾阔双除方案：吡氟酰草胺+精喹禾灵复配，同步防除狗尾草（禾本科）和猪殃殃（阔叶），减少施药次数。

大蒜茎叶处理最佳时段：选择无风晴天上午 9-11 点或下午 4-6 点施药，此时气孔开放，药液吸收效率高。

雾化效果优化：使用扇形喷头，雾滴粒径控制在 150-300 微米，提高药液附着率，减少飘移损失。

杂草抗性监测：定期田间调查，若发现防效下降，需检测杂草抗性基因，及时调整药剂种类。

小气候影响：低洼地块易积水，喷药后需及时排水，避免药液长时间浸泡大蒜根系引发烂根。

生物除草剂补充：在有机种植区可喷施枯草芽孢杆菌制剂，抑制杂草种子萌发，减少化学药剂依赖。

机械中耕辅助：行间中耕深度 5-8 厘米，切断杂草根系，同时培土护苗，增强大蒜抗倒伏能力。

残留杂草处理：对漏防的大株杂草，采用人工拔除后集中堆沤发酵，避免种子散落田间。

大蒜根系特点：大蒜为弦线状须根，无主根，根系浅且分布范围小，主要集中于 30cm 耕作层。根群横向扩展半径约 15cm，吸收能力弱，需勤施薄肥。根系分泌物含杀菌物质，是优

良前茬作物。

鳞茎（蒜头）结构：蒜头由茎盘上侧芽发育的鳞芽（蒜瓣）组成，外层为干膜质保护叶，内层为储藏叶。蒜瓣数 4-20 个不等，排列成 1-3 轮，独头蒜因未分化鳞芽形成。

花茎（蒜薹）发育：花茎从茎盘顶端抽出，总苞内含气生鳞茎（蒜珠）。抽薹需低温春化和长日照，若环境不适会导致半抽薹或不抽薹，影响蒜头产量。

休眠期特性：蒜头成熟后进入 60-70 天生理休眠，休眠期内即使条件适宜也不发芽。3-5℃低温或 28℃以上高温可延长休眠，用于储藏调控。

大蒜贮藏保鲜：（1）低温贮藏：将大蒜存放在 0-4℃的环境中，可延长保鲜期，防止发芽和腐烂。（2）湿度控制：保持相对湿度在 65%-70%，过高易导致霉变，过低则引起干缩。（3）通风：保持库内空气流通，降低二氧化碳浓度，防止大蒜呼吸作用过强。

大蒜加工：（1）蒜蓉加工：将大蒜破碎成蓉，可用于调味品、方便食品等，提高附加值。（2）脱水蒜片：采用真空冷冻干燥技术，保持大蒜营养成分，便于长期储存。（3）发酵蒜制品：如腊八蒜、糖蒜等，具有独特风味，深受消费者喜爱。

大蒜生产中存在的问题及解决途径：（1）品种退化：选育抗病、优质、高产的新品种，提高大蒜产量和品质。（2）病虫害：采用生物防治、物理防治和化学防治相结合的方法，降低病虫害发生率。（3）土壤连作障碍：实行轮作、深耕、增施有机肥等，改善土壤结构。

低温贮藏：大蒜的最佳贮藏温度为 0-4℃，此温度范围可以显著减缓大蒜的呼吸速率，延长其保鲜期，同时防止大蒜发芽和腐烂。

湿度控制：贮藏环境的相对湿度应保持在 65%-70%。湿度过高会导致大蒜霉变，湿度过低则会使大蒜失水皱缩。

通风管理：良好的通风可以降低贮藏环境中的二氧化碳浓度，减少大蒜的呼吸作用，防止热量和湿度的积累。

预冷处理：在大蒜贮藏前进行预冷处理，可以迅速降低大蒜的温度，减少生理病害的发生。

低温贮藏：大蒜的最佳贮藏温度为 0-4℃，此温度范围可以显著减缓大蒜的呼吸速率，延长其保鲜期，同时防止大蒜发芽和腐烂。

湿度控制：贮藏环境的相对湿度应保持在 65%-70%。湿度过高会导致大蒜霉变，湿度过低则会使大蒜失水皱缩。

通风管理：良好的通风可以降低贮藏环境中的二氧化碳浓度，减少大蒜的呼吸作用，防止热量和湿度的积累。

预冷处理：在大蒜贮藏前进行预冷处理，可以迅速降低大蒜的温度，减少生理病害的发生。

蒜蓉加工：将新鲜大蒜破碎成蓉，适用于调味品、速食产品等，加工过程中需保持卫生，避免微生物污染。

蒜片加工：将大蒜切片，可用于烹饪或制作蒜盐等，切片厚度需均匀，以提高产品质量。

蒜粉生产：将干燥的大蒜磨成粉末，适用于调味品和食品添加剂，加工时需控制粉末的粒度。

蒜油提取：通过蒸馏、萃取等方法提取大蒜油，用于食品调味和医药保健品，提取过程需保持低温，以防有效成分破坏。

发酵大蒜：利用微生物发酵技术，生产具有独特风味的发酵大蒜产品，发酵条件需严格控制。

大蒜酱制作：将大蒜与其他食材混合，制成大蒜酱，适用于蘸食或烹饪，注意食材配比和卫生条件。

大蒜调味品：如蒜盐、蒜蓉酱等，需确保调味品的风味和品质稳定。

大蒜保健品：提取大蒜中的有效成分，如大蒜素，用于生产保健品，加工过程中需保持活性成分。

大蒜酒酿造：将大蒜与酒精发酵，制成具有保健功能的大蒜酒，酿造工艺需严格控制。

大蒜醋生产：将大蒜浸泡在醋中，制成大蒜醋，具有调味和保健作用，浸泡时间需充分。

大蒜素提取：通过化学或生物方法提取大蒜素，用于医药和保健品，提取效率是关键。
土壤连作障碍：长期连作导致土壤病虫害累积，土壤肥力下降。解决途径是实行轮作制度，种植其他作物以改善土壤结构。
病虫害问题：大蒜易受叶枯病、根腐病、蒜蛆等病虫害侵害。解决途径是采用生物防治、物理防治和化学防治相结合的方法。
品种退化：长期自交或品种不纯导致大蒜品种退化。解决途径是进行品种提纯复壮或引进新的优良品种。
水资源短缺：大蒜生长需水量大，而水资源不足。解决途径是采用节水灌溉技术，如滴灌、喷灌。
肥料使用不当：过量或不当使用化肥导致土壤盐渍化。解决途径是推广测土配方施肥，增施有机肥。
产量不稳定：受气候、土壤等因素影响，大蒜产量波动大。解决途径是选择适应当地环境的品种，加强田间管理。
大蒜二次生长类型：根据二次生长在大蒜植株上发生的部位,可分为以下 3 种类型：外层型二次生长，内层型二次生长，气生鳞茎型二次生长。
大蒜发生二次生长的原因：据目前所知,与大蒜二次生长有关的影响因素有以下 8 个方面：品种遗传性，蒜种贮藏期间的温度和湿度，播种期，蒜瓣大小，灌水，施肥，覆盖栽培，气候。
防止大蒜二次生长的途径：大蒜产区，特别是商品蒜出口基地，对当地主栽品种的二次生长类型要有所了解。从外地引种时，最好进行以防止二次生长特别是外层型二次生长为主要目的的品种试验。
裂头散瓣产生的原因：品种特性，地下水位高,土质黏重，播期不当，田间管理措施不当，采收时期及方法不当，蒜头收获后遇连阴雨，贮藏方法不当。
大蒜的抽薹性：大蒜的抽薹性主要取决于品种的遗传性，有完全抽薹、不完全抽薹及不抽薹品种之分。
大蒜叶尖枯黄的原因：大蒜叶尖枯黄除了可能在烂母期发生外，在其他时期也可能发生。其原因主要有以下几个方面。第一，退母期养分供应不足。第二，冬季土壤干燥，水分供应不足。第三，土壤排水不良，根系呼吸受阻，植株受湿害。第四，土质黏重而且耕土层浅，根系分布浅，易受土壤过干过湿的影响，春季气温上升时，表现明显。第五，在相同栽培条件下，不同品种间叶尖枯黄的程度有差异。
防止或减轻叶尖枯黄的根本途径：防止或减轻叶尖枯黄的根本途径是：选择地下水位较低，排水良好的砂壤土种蒜；实行高畦栽培，加深耕土层，促进根系发达；维持比较稳定的土壤湿度，避免忽干忽湿以及保证“烂母期”的养分供应。
瘫苗：大蒜未达收获期,植株假茎便变软，叶片枯黄，瘫伏在地上，称为“瘫苗”，也叫“瘫秧”。这是一种早衰现象，严重影响大蒜产量和品质。
产生瘫苗的原因：第一，大蒜是否容易早衰，与品种习性有关。第二，重茬地病虫害严重，地下害虫为害根系，引起植株早衰。第三，肥水管理不当，苗子营养不良或过量施用氮肥使苗徒长，也容易引起瘫苗。
管状叶：大蒜的正常叶片是狭长而扁平的，管状叶则呈中空的管状，形似葱叶。
管状叶的防治措施：秋播地区可采取以下防治措施：蒜种在室温下贮藏，避免长期处于 15℃以下的冷凉环境中；选用中等大小的蒜瓣播种；适期晚播；保持适宜的土壤湿度，避免长期缺水。
开花蒜的形态特征：蒜头外皮破裂,蒜瓣上部向外裂开,似开花状。
大蒜营养丰富，风味独特，用途广泛，具有杀菌、抑菌、抗病毒等医疗和保健功能。

大蒜最早开始只是用于预防瘟疫和治病，后来逐渐作为食用。
大蒜的营养价值十分丰富。有水分，蛋白质，脂肪，碳水化合物，钙，磷，铁，维生素 C。此外,还含有维生素 B1、核黄素、烟酸、蒜素、柠檬醛及硒和锗等微量元素。大蒜中含有 17 种氨基酸，其中 8 种为人体必需氨基酸，尤以精氨酸含量最高，占氨基酸总量的 20.4%，其次是谷氨酸，占氨基酸总量的 19.75%。
蒜头中微量元素硒的含量在蔬菜中是最高的,硒是人体必需的微量元素，并具有抗氧化功能,被认为有防癌作用。
大蒜自古就被当作天然杀菌剂，有“天然抗生素”之称。它没有任何副作用，是人体循环及神经系统的天然强健剂。
大蒜含有含硫化合物、氨基酸、糖类、甙类、维生素、肽类、蛋白质、酶类、脂肪及无机盐等多种成分，具有防癌抗癌、消炎、杀菌、抗病毒、抗凝血、降血脂、降胆固醇、预防动脉硬化等多重功效.
大蒜素被誉为天然广谱抗生素药物，能抑制多种细菌，在降低心血管疾病的风险上，大蒜一直扮演着非常重要的角色。
大蒜对防治心脑血管疾病有较好疗效，其药理与大蒜能够使血脂水平正常化、增进血内纤维蛋白的活性、抑制血小板聚合、降低血压与血糖、防血栓形成、清除氧自由基、抗缺血再灌注损伤、细胞保护、钙拮抗、扩管降压等作用有关。
大蒜有降低血糖的作用，其机理是大蒜能促进胰岛素的合成，增加组织细胞对葡萄糖的吸收利用。
大蒜中富含的硒可以降低某些有毒元素及物质的毒性，如抵抗和减低汞、镉、铊、砷等。大蒜能治疗人体铅中毒, 对镉染毒大鼠有解毒作用, 并比某些传统解毒剂更有效。大蒜还对铅、汞染毒大鼠有解毒作用，对甲基汞染毒小鼠具有一定的预防作用。
大蒜能激活人体巨噬细胞功能，提高机体免疫力,同时，大蒜所含的有效成分可增加物质代谢、能量转换,并能促进血液循环，改善体质。
将大蒜用于畜牧和水产养殖饲料添加剂可以部分替代抗生素从而减少微生物产生的抗药性,对畜禽及鱼类的健康产生有益的影响, 在饲料中添加大蒜素可作为解决抗生素问题的一条途径。
大蒜素可以改善饲料的适口性,通过添加大蒜素可明显改善饲料的适口性、提高动物采食量。大蒜素通过气味吸引动物对饲料的食用，使之产生食欲，从而提高采食量。
大蒜素不仅能增加动物的采食量，而且能防治多种疾病，提高免疫机能，改善动物体内各系统组织的功能，促进胃肠的蠕动和各种消化酶的分泌，提高畜禽及鱼类对饲料的消化利用,从而使生产性能提高，降低饲料成本。
大蒜营养丰富，药用保健价值较高，开发利用价值极高。
大蒜是喜冷凉气候条件的作物，特别是发芽期和幼苗期适宜较低的温度,发芽的始温为 3～5℃，发芽及幼苗期最适温度为 12～16℃。此期温度过高，植株呼吸作用增强，养分消耗较多，生长受抑制.
大蒜的叶片呈带状，叶面积小，表面有蜡质，可防止水分快速蒸发，使大蒜具有一定的耐旱性,但由于根系小，根毛少，吸收能力弱，所以要求的土壤湿度很严格。
大蒜喜好富含有机质、疏松肥沃、通气良好、保水、排水和保肥性能良好的微酸性沙壤土或壤土，土壤 pH 为 5.5～6.0 最适合大蒜种植。
大蒜的根系弱，吸收力差，而需肥又多，根据这一特点，施肥时应本着多次、少量的原则,施肥后注意立即浇水，以利吸收。
土壤中的多种有益微生物是好气性的，微生物活动旺盛，有机物质被微生物迅速而彻底地分解，土壤空气中氧气充足时形成大量速效氮养分。缺氧时，有机质分解缓慢且不彻底，常积

累有害的物质，对大蒜生长不利。减少土壤积水、中耕松土可以改善土壤通气条件，对大蒜根系生长有利。
大蒜叶形直立，呈带状披针形，各叶层间相互遮光面较小，是适宜密植的典型形态特征。
大蒜鳞茎膨大和抽薹都对生态条件有严格的要求，而且引种优良实践证明，一般大蒜品种的生态适应性不强。因此，生产中品种的选择首先应该因地制宜，优先考虑就地选用的原则，充分发掘地方名优大蒜品种资源。
大蒜产品多样，每茬生产要收获的主要产品不同，品种选择应注意生产的主要目的。
大蒜的栽培季节既取决于当地的气候和环境条件，也与品种的特性有关。另外，不同大蒜的生育期对温度有不同的要求，因此栽培季节的确定，要根据大蒜不同的品种，在不同的生育阶段对生长环境的要求以及各地区的气候条件来确定。
大蒜的栽培季节随南北各地气候的不同而有所差别。在北纬 35°以南的地区，冬季不太寒冷，大蒜幼苗可以安全露地越冬，以秋播为主；北纬 38°以北的地区，冬季严寒，幼苗不能自然露地越冬，秋播容易遭受冻害，以早春播种为宜；北纬 35°～38°之间的地区，春播、秋播均可。长江流域一般进行秋播，由于秋播生育期较长，产量明显高于春播，所以如果温度环境允许，最好进行秋播。
春播大蒜的幼苗生长期明显缩短，所以在适宜的播种时期内，应尽可能早播，以延长生长期。
大蒜忌连作，与葱蒜类蔬菜重茬，植株细弱，叶片变黄，产量降低，还容易遭受病虫害。
大蒜体内普遍存在病毒，并且随着种植年限的增加，病毒不断累加，在生长过程中大蒜还会分泌一些对自身产生毒害作用的化感自毒物质，并通过地上淋溶、根系分泌物和残体分解物遗留在土壤中。随着大蒜连作年限的增加，这些自毒物质在土壤中的积累越来越多，从而抑制大蒜自身的生长。
大蒜对土壤中矿质营养元素的需求种类及吸收比例有一定的规律，即大蒜对土壤养分的吸收是有选择的。
大蒜播种前需要对种瓣进行一些处理，以促进萌芽发根，减少病虫为害。
大蒜虽然对土壤的适应性较强，除了盐碱沙荒地外，均能生长。但是由于大蒜根系浅，吸收能力弱，对土壤有一定的要求才能达到优质高产的目的。
大蒜的基肥施用量应根据大蒜的目标产量和形成单位产量的吸肥量等多种因素综合考虑。
大蒜播种方法因做畦方式不同而不同，有平畦播种、高垄播种、地膜覆盖畦播种等。
大蒜播种时应尽量避免种瓣受损伤,土壤板结坚硬或开沟深度不够时，应重新开沟或挖穴播种，切勿捏住种瓣顶部用力往土里按，以免挤压损伤种蒜而影响出苗。
合理的栽培密度是达到大蒜优质、高产、高效的关键措施之一，在确定栽培密度时，应考虑到品种特点、种瓣大小、播种期、土壤肥力程度和栽培方式等因素。
大蒜的生长发育过程，分为 6 个时期，其中 5 个时期是在田间度过的。各生育期有其自身的规律，对环境条件的要求也不同,生产者必须根据不同生长发育时期的特点来确定其田间管理方案和具体栽培措施.
秋播大蒜的幼苗期一般是在冬季度过的，田间管理的中心任务是培育壮苗，确保幼苗安全越冬。
大蒜地膜覆盖栽培也为秋播大蒜解决了晚茬大蒜成熟晚、产量低、质量差以及北方干旱地区用水量大等问题。
青蒜（蒜苗）是大蒜的青苗，是以鲜嫩翠绿的蒜叶和洁白嫩脆或白嫩透红的假茎作为食用器官的重要蔬菜，也可作为调味品，一年四季均可生产和应市。
蒜黄的产值较高，应该选择大瓣、休眠期短的品种，以求发芽快、生长健壮、产量高。
大蒜产区要想达到持续高产、高效、优质的目标，不但要注意茬口安排，而且应建立合理的多年轮作制度，最好每隔 2～3 年种 1 茬大蒜。

大蒜的间作套种方式主要有粮、蒜套种，棉、蒜套种，菜、蒜套种，粮、棉、蒜套种，棉、粮、蒜、菜套种，粮、菜、蒜套种，棉、蒜、瓜套种及棉、蒜、瓜、菜套种等方式。
为了提高种植效益，可采用地膜覆盖大蒜套种花生栽培模式。该栽培模式为大蒜优质高产提供了良好的生长环境，花生的播种期比麦垄套种提前 15～20 天，比麦后直播提前 30～40 天，收获期提前 10～15 天。
大蒜栽种前一定要施足底肥。最佳播种期以在 10 月 5～15 日之间为宜，采取开沟播种的方法，沟深 3～4cm，地温不低于 16℃时播种，14～23℃出苗。
大蒜一般在 9 月下旬～10 月上旬播种。菠菜的播期与大蒜的播期相同，但菠菜应撒播在畦埂上,大蒜出苗慢，苗期占空间小，菠菜有充分的生长空间。
大蒜属耐寒性作物，可尽量提早播种，这样墒情好，有利于苗全、苗壮，最佳播种期应在 4 月 5 日左右。大豆播种时期也很重要，播种过早影响大蒜生长.
大蒜、菠菜、棉花间套种模式的优点在于棉花的前茬抢种一茬菠菜，在棉花种植密度不减的前提下又套种一茬大蒜，经济效益十分可观。
大蒜覆盖栽培有两种方式，一种是地膜覆盖栽培，另一种是塑料拱棚覆盖栽培，目前应用较普遍的是前一种方式。
大蒜地膜覆盖栽培有增产增收的效果，但有时会出现二次生长增多，蒜头形状不整齐，蒜瓣数增多，蒜薹短缩、发育不正常等现象，究其原因是与地膜覆盖后土壤温、湿度及养分的变化有关。
大蒜二次生长发生的程度，在不同年份往往有很大的差异:气候包括温度、降水、空气湿度、日照等因素，有关气候变化与二次生长关系的研究还很少，就现有研究资料来看，以花芽和鳞芽分化为中心的气候状况，对二次生长的发生有较大的影响。
大蒜二次生长的发生与种瓣大小密切相关。种瓣过大二次生长率高，种瓣过小产量低，又易产生独头蒜。生产中应选用适中的种瓣为宜。
大蒜的适宜生长温度为 12～26℃，蒜瓣在 12℃以上发芽生长整齐。秋播大蒜播种过早，当年会感受低温而分瓣，在持续低温下，幼小鳞芽还可以再次感受低温而通过春化，第二年这些大蒜就会形成二次生长的复瓣蒜.
大蒜的适应性较强，但在生长过程中对环境条件、水分都十分敏感，管理过程中的大肥大水，土壤含水量过大，都会造成面包蒜的产生。
大蒜播种过早或过晚，均会诱发异常生长现象,大蒜播种过早，由于温度高，蒜瓣发芽慢，出苗期长，出苗率低，幼苗在冬前生长过旺，发育进程加快，抗寒力下降，植株早衰，减弱了蒜鳞茎肥大期的光合作用和养分的积累，不能正常接受低温长日照的春化。
大蒜的抽薹性主要取决于品种的遗传性，有完全抽薹、不完全抽薹及不抽薹品种之分。但有时原来是完全抽薹的品种，却出现大量不抽薹或不完全抽薹的植株，这种现象称为抽薹不良。
大蒜的根系是喜湿根系，须根分布范围小，对水肥要求较高。土壤过干或过湿，肥力不足，极易出现黄叶干尖。
大蒜正常叶片呈狭长的扁平带状，横切呈“V”字形，管状叶则呈中空的管状，形似葱叶，横切呈环状，只在顶端有很小的出叶口，这是大蒜分化中的一种异常现象。
大蒜生长期间，遇低温引起的寒流，导致大蒜叶片受冻失绿，产生严重的叶尖干枯。如果在大蒜生长期间缺肥缺水，或施肥过剩也能引起生理性危害，如大蒜生长受抑制，蒜头膨大受阻。
大蒜缺钙，会影响根系和生长点的发育，降低组织内部碳水化合物的含量，使蒜头膨大受阻，产量降低，品质下降，同时也诱发心腐病。
一般来讲，大蒜长到一定高度，要控制氮肥，增施钾肥,如果在蒜头膨大期间缺钾，易感染心腐病、白腐病。

大蒜播后、苗前用除草剂过量，特别是乙草胺用量过大，易引起叶片生长受抑制、失绿发黄、个别心叶扭曲、根系减少、蒜头变小等症状。

大蒜病毒病又名花叶病，是世界性病害，也是为害大蒜最大、发病率最高的一种病害。

大蒜病毒病是由多种病毒侵染引起的，病毒主要有大蒜花叶病毒和大蒜潜隐病毒。

大蒜主要虫害有蒜蛆、蓟马、蚜虫、螨类、潜叶蝇、粪蚊和跳虫等。

大蒜蚜虫有桃蚜（又称烟蚜、桃赤蚜)、葱蚜两种，属同翅目蚜科。

大蒜粪蚊属双翅目粪蚊科的害虫，在大蒜整个生育期都可为害，是大蒜生产上的危险性害虫。

大蒜的适应性较强，但在生长过程中对环境条件、水分都十分敏感，管理过程中的大肥大水，土壤含水量过大，都会造成面包蒜的产生。

大蒜的抽薹性主要取决于品种的遗传性，有完全抽薹、不完全抽薹及不抽薹品种之分。

大蒜的根系是喜湿根系，须根分布范围小，对水肥要求较高。土壤过干或过湿，肥力不足，极易出现黄叶干尖。

大蒜正常叶片呈狭长的扁平带状，横切呈“V”字形，管状叶则呈中空的管状，形似葱叶，横切呈环状，只在顶端有很小的出叶口，这是大蒜分化中的一种异常现象。

大蒜收获期尚未达到，植株假茎便变软，叶片枯黄，瘫伏在地上，称为瘫苗，也叫“瘫秧”。这是一种早衰现象，会严重影响大蒜的产量和品质。

大蒜在储藏期间，有些蒜头外观完好，但内部蒜瓣干缩变黑，整个蒜头成为空包，俗称棉花蒜，蒜农称为黑粉蒜。

大蒜干腐病在生育期和储藏运输期可发生，尤其是在储运期发生严重。

大蒜叶枯病不仅是大蒜的一种重要病害，而且危害洋葱、大葱、韭菜等葱属类蔬菜。此病由真菌子囊菌亚门枯叶格孢腔菌侵染所致。

大蒜病毒病又名花叶病，是世界性病害，也是为害大蒜最大、发病率最高的一种病害。

大蒜病毒病是由多种病毒侵染引起的，病毒主要有大蒜花叶病毒和大蒜潜隐病毒。

大蒜粪蚊属双翅目粪蚊科的害虫，在大蒜整个生育期都可为害，是大蒜生产上的危险性害虫。

大蒜的简易鲜藏，既不需要特殊复杂的工艺设备，更不需要固定的储存场所。因地制宜，就地取材，是在自然温度状态下的储藏方式，其优点是简便易行，成本低，是常用的储藏方式。

大蒜的窖址一定要选在干燥、地势高、不积水、通风好的地方。窖内温度由窖的深浅决定，要经常清理窖，及时剔除病变烂蒜。

大蒜营养价值虽高，但大蒜及其制品在食用后口腔中会产生一种难闻的臭味，让人难以接受。

大蒜油是深加工产品中附加值较高的产品之一。大蒜特有的香气和香味，也是加工其他高附加值产品的重要原料。

大蒜素是黄色液体，具有强烈刺激味和蒜所特有的辛辣味。不耐热，遇碱不稳定但遇酸较稳定，难溶于水，可与乙醇、乙醚和苯等混合。

大蒜播种后 7～10 天即可出土。在蒜芽快要出土时，清晨用新扫把轻拍地膜，使蒜芽顶破地膜，少数未能顶出地膜的，应用小铁钩及时破膜引苗，以使蒜苗顺利顶出地膜。

大蒜覆膜栽培的，膜下温度高，利于多种病原物和病虫越冬，加重了病虫害的发生。

大蒜需肥较多，掌握以有机肥料为主、化肥为辅，基肥为主、追肥为辅的施肥原则。

大蒜幼苗生长期虽有种瓣营养，但为促进幼苗生长，增大植株的营养面积，仍应适期追肥。

大蒜又名胡蒜。起源于中亚(包括印度的西北部、阿富汗、塔吉克斯坦和乌兹别克斯坦以及天山西部)。最早在古埃及、古罗马和古希腊等地中海沿岸国家栽培,开始只是用于预防瘟疫和治病。

大蒜的独特辛辣气味可以解除鱼、肉的腥味,增进食欲,是膳食烹调中不可缺少的调味品。

大蒜是广谱杀菌素,对危害人或畜、禽的多种病原菌(如葡萄球菌、化脓性球菌、痢疾杆菌、大肠杆菌、伤寒杆菌、结核杆菌、白喉杆菌、炭疽杆菌、霍乱弧菌、脑膜炎及肺炎双球菌、

链球菌等)有抑杀作用。
大蒜在兽医临床和饲料添加剂方面的应用,显示出广阔的开发前景。
大蒜对危害植物的真菌性病害,如瓜类白粉病、猝倒病、枯萎病,番茄早疫病、灰霉病,芹菜斑枯病,棉花炭疽病、立枯病,小麦锈病等的病原菌,有抑制其孢子萌发和菌丝生长的作用。
大蒜有较强的抗氧化作用,可有效清除活性氧自由基,保护生物膜结构的完整,防止细胞和组织的癌变。
大蒜鳞茎盘顶部的生长锥分化为花芽后,逐步发育抽生蒜薹。蒜薹的下部为花茎,顶端着生花苞称总苞。
大蒜的叶片包括叶鞘和叶身两部分。叶鞘呈圆筒形,着生在茎盘上。每一片叶均由先发生的前一片叶的出叶口伸出,许多层叶鞘套在一起,形成直立的圆柱形茎秆,由于它不是真正的茎,故称"假茎"。
大蒜的叶片互生,对称排列。叶片的排列方向与蒜瓣的背腹连线垂直。
大蒜在我国栽培的历史悠久,栽培地区遍及全国,各大蒜产区一般都有适应本地区生态条件的优良品种,因而了解大蒜品种的生态型对大蒜引种和栽培都有重要指导意义。
大蒜生态型的形成不但和纬度有关,而且在很大程度上受海拔高度的影响。
大蒜从用蒜瓣播种到收获蒜头,重新获得新的蒜瓣,进入休眠状态称为一个生育周期。
大蒜鳞茎成熟后即进入休眠状态,这时即使给予适宜的温度和水分,鳞芽也不会萌芽和发根,这段时间称为大蒜的生理休眠期。
大蒜的直立叶形是适宜密植的形态特征。但是,和其他蔬菜一样,在一定的密度范围内,总产量随单位面积株数的增加而增加。
大蒜的根系浅,根毛少,吸水范围较小,所以不耐旱,但不同生育期对土壤湿度的要求有差异。
大蒜叶片呈带状,较厚,表面积小,尤其是叶表面有蜡粉等保护组织,地上部具有耐旱的特征。
大蒜由于根系吸收力较弱,对土壤肥力的要求较高,适宜在富含有机质、透气性好、保水、排水性能好的砂质壤土或壤土中栽培。
大蒜喜微酸性土壤,适宜的土壤氢离子浓度约为 1000 纳摩/升(pH 值为 6)。在碱性大的土壤中种蒜,蒜种容易腐烂,植株生长不良,独头蒜增多,蒜头变小。
大蒜对富含腐殖质的有机肥反应良好,增产效果显著。全生育期吸收的氮最多,钾次之,磷最少。
大蒜出苗期和幼苗期的生长主要靠种瓣中贮藏的养分,从土壤中吸收的氮、磷、钾量很少,所以在使用基肥的基础上一般不需要再施种肥。
大蒜的栽培季节因气候条件和品种特性而异。按气候条件可划分为秋播和春播两大区。
秋播大蒜幼苗的生长期长,有充分的低温期可以满足花芽和鳞芽分化的需要,而且在高温长日照季节来临以前,幼苗可以充分生长,为蒜薹和蒜头的增长积累丰富的营养,所以抽薹率高,蒜薹产量高。
大蒜忌连作。连年在同一块地里种大蒜,或与葱蒜类蔬菜(大葱、洋葱、韭菜等)重茬,则病虫害严重,出苗率低,植株细弱,叶片发黄,蒜薹和蒜头产量降低。
大蒜的根系分泌物有杀菌作用,同时蒜的吸肥量较少,土壤中残留的养分较多,所以是其他非葱蒜类蔬菜和农作物的良好前茬。
大蒜秋播地区的轮作制度有水旱作物轮作和旱地作物轮作 2 种类型。
,大蒜根系分泌物对病菌的繁殖有抑制作用,加上蒜、稻轮作田改变了病、虫生活的环境条件,可以减轻水稻病害和地下害虫(金针虫、蝼蛄等)的危害。
大蒜春播地区轮作方式有:第一年春季种马铃薯、西葫芦、黄瓜或菜豆,收获后种大白菜,大白菜收获后土地休闲;第二年春播大蒜,大蒜收获后栽甘蓝、菜花、芹菜或播种胡萝卜、萝卜等;第三年再种夏菜。如此轮换种植。
大蒜根系浅,分布范围窄,植株矮小直立,而且其生长期处于玉米生育后期和棉花苗期,所以对

玉米和棉花的生育没有不良影响,又多收一茬蒜。
大蒜以露地栽培为主,生产蒜头、蒜薹和蒜苗(青蒜)。冬春低温期也可在保护地中栽培,生产蒜苗和蒜黄,以调节淡季蔬菜供应。
春播大蒜一般采用平畦,也有采用高垄栽培的,如北京、新疆、黑龙江等大蒜产区。
大蒜播种期是否适当,对蒜薹和蒜头产量与品质都有很大影响。播种过早,由于温度高,蒜瓣发芽慢,出苗期长,幼苗在冬前生长过旺,抗寒力下降,生长后期下部叶片枯黄,植株早衰,从而减弱大蒜鳞茎肥大期的光合作用和养分的积累,使蒜头变小。
大蒜的种植密度应根据当地的综合条件,如品种特性、蒜种大小、土壤肥力及栽培方式等,通过田间试验确定。
大蒜瓣所含营养物质较丰富,播种后根系发达,植株生长旺盛,假茎较粗,叶片数较多,单株叶面积较大,蒜头较大,产量也较高,所以需要较大的空间和营养面积,可适当种稀些。小瓣蒜播种则应适当密植。
秋播大蒜和春播大蒜幼苗期所处的环境条件不同,幼苗期的田间管理也应有所区别。
秋播大蒜的幼苗期一般是在冬季度过的,田间管理的中心任务是培育壮苗,确保幼苗安全越冬。
大蒜花芽和鳞芽的开始分化期与品种习性有密切关系,但同一品种的花芽和鳞芽分化结束期却很相近,所以不同品种间这一生育期的长短仍有较大的差异。
春播大蒜的花芽和鳞芽分化发育期较短,烂母期也较短,追肥、灌水等田间管理要相应提前,如延误时机或水、肥供应不足,则会影响花芽和鳞芽的分化发育,不但抽薹率降低,还会增加独头蒜的比例。
秋播大蒜花芽分化结束期一般在早春,温度低,所以花茎的伸长开始很缓慢。
秋播大蒜种的早熟品种为 80 天左右,中、晚熟品种为 40~50 天;春播大蒜的早熟品种为 55 天左右,晚熟品种为 45 天左右。
春播大蒜地区为了在冬季和早春供应蒜苗,多采用加温温室或日光温室栽培。如果没有温室,还可以利用以马粪酿热物或地热线加温的温床栽培蒜苗。保护地蒜苗栽培技术容易掌握,生产周期短,经济效益高,是增加冬、春市场鲜嫩细菜供应的重要方法。
大蒜不能正常结种子,生产上长期采用蒜瓣进行无性繁殖,从蒜瓣到蒜瓣,不经过授粉受精产生种子的有性世代,则不能产生生活力强的后代,因而易导致品种退化。
生产大蒜的名产区必定是具备某些品种生育最佳的气候、土壤、地形、地势条件及栽培技术的地区。许多大蒜产区都有每隔 1~2 年到名产区换种的习惯。
大蒜用蒜瓣作繁殖材料时,1 个蒜头中经挑选后可用于播种的蒜瓣不过 6~8 瓣(蒜瓣多的品种多一些),也就是说,繁殖系数一般为 6~8,所以单位面积的用种量很大,使生产成本增大。
大蒜感染病毒病是引起种性退化的主要原因。国内主要大蒜产区,病毒病严重时,发病株率几乎达到 100%。由于大蒜是无性繁殖植物,连年利用蒜瓣繁殖,使病毒病逐年积累并代代相传,致使种性退化,产量和品质逐年下降。
大蒜病害主要有病毒病、灰霉病、叶枯病、紫斑病、白腐病等。
大蒜病毒病是世界性病害,也是对大蒜危害性最大、发病率最高的一种病害。病毒一旦侵入植株体内,不但对当代有影响,而且鳞茎母体带毒后便以垂直传染方式传递给后代,导致种性退化。
大蒜的害虫主要有蚜虫、根蛆、葱蓟马、螨类、茎线虫、大蒜粪蚊、豌豆潜叶蝇及轮紫斑跳虫。
大蒜播后出苗慢,苗期长,加上叶片窄长,地面长期不能形成荫蔽状态,从而给杂草的滋生创造了有利条件。
大蒜的加工产品多种多样,包括食品、医疗保健品及畜禽饲料添加剂等。大蒜食品有干制、盐渍、糖渍、糖醋渍、饮料、酱、酒、油及调味品等。

大蒜中的蒜氨酸本来是没有臭味的,当切碎或捣烂时,蒜的细胞被破坏,细胞中的蒜酶会活化,将蒜氨酸分解,产生大蒜素。
大蒜的脱臭方法包括化学脱臭和物理脱臭两大类,大多需要购置化学药品和仪器设备,而且手续比较多。用食盐水灭酶不失为目前简便易行的大蒜脱臭方法。
大蒜辣椒酱是利用大蒜和辣椒自身含有的杀菌成分,抑制微生物的活动,延长产品的保质期。
大蒜选蒜瓣肥大、未发芽、无霉变、无病斑、无虫伤、肉质脆嫩、色泽洁白的蒜头。辣椒选色泽鲜红、个大、整齐、无霉烂、无病斑、无虫伤的尖辣椒品种。
大蒜辣椒酱的配方中除蒜和辣椒外,还有豆豉、食盐、酱油、白酒、味精等调料。
大蒜生产中存在的问题很多,有的属于生理异常现象,有的属于栽培技术不当;有的带普遍性,有的则是在少数地区、少数年份发生。
大蒜二次生长是蒜头收获前蒜瓣就萌发生长的异常现象。国内外对这种现象采用的名词很多,除二次生长外还有次生蒜、马尾蒜、胡子蒜、分株蒜、分杈蒜、背娃蒜、复瓣蒜、再生叶薹、收获前萌发、母子蒜、分球、带侧枝蒜等。
大蒜二次生长类型虽然主要取决于品种的遗传性,但不同品种间的遗传稳定性有差异。
大蒜植株基部不膨大形成蒜头,或膨大形成结构类似洋葱鳞茎的松软蒜头,故名"葱头蒜"。
大蒜未达收获期,植株假茎便变软,叶片枯黄,瘫伏在地上,称为"瘫苗",也叫"瘫秧"。这是一种早衰现象,严重影响大蒜产量和品质。
大蒜是我国重要的蔬菜之一，具有很高的营养价值和药用价值。然而大蒜种植需要大量的水肥资源。而传统的施肥灌溉方式会导致大量水资源和肥料的浪费，利用率低流失多。
大蒜的需肥规律：前期和中期以氮素化肥为主，开花坐果(鳞芽、鳞茎分化)开始承步转向以磷肥为主，蒜头膨大期逐步转向以钾元素为主的生长发育。
大蒜需水最多的时期，往往需肥量也大，特别是中后期，一般浇水要配比一定含量的肥料，随水带肥。
大蒜水肥一体化, 必须施用水溶性肥料, 全水溶无残渣, 利用率显著提高, 速溶性肥料最好,效果快。
大蒜水肥一体化, 要计算好水肥比例, 浓度过大作物不能吸收。浓度过小达不到应有的效果,适量配比。
大蒜是食品的一种调料，广泛用于吃食当中，具有消毒、杀菌、健肠胃等多种功能，称为四季良药大蒜生姜。
大蒜的植物学分类，属于植物界，被子植物门，单子叶植物纲，百合目，百合科，葱属，蒜种，又分为多个亚种和小种。
大蒜喜凉爽怕炎热，10-12℃可以发芽，15-22℃生长发育最快最好。5℃左右停止发育，-8—-10℃可以忍耐，超过-10℃开始死亡。高温 25℃以上根系不能吸水肥，自然死亡。
大蒜适宜的酸碱度 PH 值，应该在 6-7.5 最好。过酸或过碱，PH 低于 5 高于 8.5 不宜种植大蒜。
大蒜的种植密度是高产的关健，一般以大蒜蒜头为主的每亩应该在 3.1-3.5 万株，可以确保亩产在 3000-3500 斤以上。
大蒜种植|时间，在黄淮流域以秋播为主。气温降至 20℃左右，10 日份在寒露节前后种植,翌年 5 月中下、旬收获，生长期 210-230 天左右，其它地区根据气候而定，也可以春播。
大蒜的需肥，一般每生产 1000 公斤大蒜头(1 吨)，需要吸收纯氮 28-29 斤，五氧化磷 9-11 斤，氧化甲 26-27 斤，大蒜种植应该以施足底肥为主，大蒜用肥是粮食作物的三倍左右，每亩施氮磷钾的总含量 120-140 斤为宜。
大蒜种子的选择, 一般要选择适合当地种植的品种, 外地引种要先试种, 成功后再大量引进。
大蒜的播种及出苗期，一般大蒜从播种到齐苗应该是 7-8 天，胚芽鞘出土保护第一片叶伸出

地面。
大蒜的生育期没自明显的分界线，从一个生育期到下一个生育期前后重叠，只有初期、盛期和末期。
大蒜是越冬作物，病虫害都比较轻。再者大蒜抗蚜虫飞虱，很少生虫子，只有生长后期有蛆和根螨危害，一般新地没种过大蒜的地块很少生。如果用药剂拌种基本不生虫。
蒜根腐病，由两种菌类引起，一是细菌类病害，二是疫霉菌属，真菌类病害。发病症状都是变黄、发黑，根尖表皮先腐烂，再整体腐烂，植株枯萎死亡。造成的原因，土壤湿度大，温度高不透气，或者化肥过多、集中烧苗，或音连年重茬，土壤带菌量大。我们要对症用药。
大蒜播种前除草,特别是没有种过庄稼的滩途地、落荒地，必须先清除杂草,特别是多年生的乔木、灌木。
大蒜是食品的一种调料，广泛应用于人们的吃食当中，葱姜蒜是大家一日三餐中不可缺少的调味品。
大蒜具有杀毒、赤菌、消炎、治疗肠胃病、缓解高血压、高血脂、高血糖等多种疾病，称为四季良药，大蒜、生姜是广大人民生适中不可缺少的物质。
大蒜具有防治瘟疫，杀死各种病毒有奇效，在历史上有不小瘟疫流行时，用大蒜煮汤预防瘟疫的实例。
大蒜可用于医药，如硒蒜胶囊、大蒜油胶囊、大蒜素等，可以有效提高维生素 B1 与葡萄糖的转化，提供人体大脑所需的能量，起到补脑、健脑、健身的作用。
大蒜素合成乙蒜素可用于农药，并且是生物型农药，可以杀虫杀菌,没有副作用，不产生残留，已推广应用。
大蒜油是一种挥发性油,能帮助脱发患者促进皮脂腺和毛囊的生长，起到治疗脱发的作用。
大蒜可用于养殖业，饲料中加一定比例的大蒜粉可以使家畜、家禽、鱼类等不生病、少生病、健康生长。
大蒜属植物界，被子植物门，单子叶植物纲，百合目，百台科，葱属，蒜种，又有很多亚种和各地的生理小种。
大蒜喜凉爽，怕炎热，可以耐-10℃的低温不死，冬季温度-15℃以下全部冻死，不能越冬。
大蒜的正常生长温度是 12 一 25℃，低于 12℃能发芽但是不能长叶，25℃以上不能吸肥吸水而死亡。
大蒜存放应该在 25℃从上进入休眠期，-1℃—-5℃也可以存放，最好在-1℃—-3℃冷库储存，5℃以上芽萌动。
大蒜在黄准海地区属二年生草本植物,生育期在七个月左右 210-220，在高寒地区或北纬 38°以上冬季-15℃—-20℃以上，冬前不能种植，只能春播，一般生育期 120-130 天左右。
大蒜是需要春化的作物,需要在 5-6 片叶以前终过 0 一 12℃的低温两个月左右，渡过春化，如果春化不好或不能顺利的通过春化，指种过早或过晚，容易出现独头或形成面包蒜和毛蒜，失去商品价值。
春化阶段：大蒜发芽以后在五、六片以前，经过 0 至 12℃的低温 2 个月以上，分化成新的蒜头，形成蒜瓣，这就叫春化(同小麦一样)。没有通过春化的大蒜形成独头，春化不好的大蒜形成气包蒜或马尾蒜。主要是特定的温度、时间、蒜苗的生长期所决定的。
光照阶段:大蒜完成春化以后，随着气温的升高进入光照阶段，这个时期大量生长根、茎、叶，积累营养，准备结实。这个时期，需要充足的适宜的光照，大蒜返青以后，叶片达到 18-20 片，单株根系达到 180 一 200 条，茎粗大到直径 1.5-1.8 厘米，叶片厚度可达 2 一 3 毫米，长度达到 40-60 厘米，叶面积指数达到 4 一 5 个，叶色浓绿，也是大蒜需肥零水最多的时期。3 月中下旬-4 月上中旬。
结实阶段:也叫生殖生长阶段，这个时期营养生长完成，根、茎、叶不在增加，体内积累了大

量的碳水化合物，抽完蒜苔以后，全部转移到蒜头，迅速膨大，形以蒜头等茎叶全部枯黄、萎缩发黄转移完成收获。
大蒜的播种及出苗期,大蒜播种一般最高气温降到 18-22℃（即可播种本地，大体时间在寒露节前后，也就 10 月 5-15 日最适，腾种后一般出苗 5-7 天。
大蒜的蒜母（蒜瓣）可以供大蒜生长五片叶，不需要从土壤中吸收营养，有水和温度即可，年前生长 6 叶一心越冬，吸肥量很少。
大蒜是越冬作物，病虫害比较轻。主要虫害集中到根部。在气温回暖以后，有根蛆和根螨，以拌种防治为最好。用噻虫嗪、高巧一类的药剂防治。
大蒜不同年份、不同气候环境条件，发病的情况不同。有些年份紫斑病、锈病、菌核病、疫病、病毒病、叶斑病等也很严重，要对症用药，防治在发病初期，不能造成危害。
大蒜除草封闭地面，乙草胺、乙氧氟草醚、施田补、扑草等。春季带跟除草，辛酰溴苯腈。
大蒜的成熟标准:蒜叶全部发黄，假茎用脚踩棉软不断，证明体内储存的碳水化合物已全部回收到蒜头。每一个蒜瓣都能鼓起,农民说有膀头，最合适产量最高。
大蒜是高含水的作物，一般含量 80%以上。晾晒非常重要，稍微不注意，容易霉变腐烂。收获后最好就地晾晒 3-5 天后再装袋，放在通风的地方，不能暴晒，一般不能少于 10-15 天,基部表皮都干了以后再入库诸存。商品大蒜要根系全部切除。
大蒜的晾晒有多种形式：全部用网袋包装，支架晾晒；竹薄晾晒等。要勤翻动，多通风。
大蒜是人民生话中不可缺少的重要调味品之一，四季良药大蒜、生姜具有杀菌、消毒、消炎、防病、防瘟疫等作用。
大蒜是重要的饲料添加剂，广泛应用于各种饲养业，同样具有防病、防瘟疫、保生长、保健康的功效。
大蒜的药品开发越来越广泛，人用的硒蒜胶囊，大蒜的食品开发也越来越多，应用也越来越广，蒜面、蒜饼等。
大蒜是一个高投入、高产出、高附加值、高效益的产业。一般相当于粮食作物的 5-7 倍，是发展高效农业的方向。各省、市、县、地区都在考查、引进、发展，做为重点开发项目，发展前景广阔。
大蒜产业由零星分散向成方连片发展，由个体的小户向种植大户发展，由手工种植向半机械化、全机械化发展，由小型机械化向大型机械化、规模化、集约化、智能化发展，先进农业机械必须代替落后的人工。
大蒜产业逐步走向集团化种植、管理、收获、晾晒、储存、加工、深加工、运输、销售等一条龙的服务，逐步走向大蒜生产的现代化，前途是光明的、美好的。
大蒜是用工特别大的作物，一般从种到收获、储存用工到整个成本的一半以上，正常情况下每亩 2000 元以上，如果没有机械协助，费时费工又耽误时间，很难大面称种植。
大蒜产业的发展壮大，靠零星分散的手工操作是不可能的，必须实现机械化，这也是产业的需求，发展的需求，社会进步的需求，必须依靠现代化的智能机械武装起来。
为了解决大蒜产业的大发展，在提供机械的通时，玛丽亚又组建了农业技术推广部，良种选择、配方施肥、科学管理、病虫防治、高产栽培等服务。
大蒜产区进行统计、排查，多大面积需要服务，什么时间进行服务，保证作业队及时到达，双方签订意向书、协议书，包括面积、质量、价格、期限等，保证种植户的利益。
大蒜田除草首先要选择适宜的除草剂，确保对大蒜安全，同时对杂草有效。
在大蒜播种前进行土壤处理，可以有效预防杂草的发生。
合理安排播种时间，尽量避开杂草生长高峰期。
采用覆膜种植，减少杂草生长空间，提高大蒜生长环境。
注意土壤湿度，适时进行中耕，破坏杂草生长环境。选用抗草性强的大蒜品种，降低除草压

力。
使用生物除草剂，减少化学农药对环境的污染。采用人工除草与机械除草相结合的方式，提高除草效果。
大蒜具有特殊的营养价值和医疗保健作用而受到高度重视。
在国内,由于广泛食用和利用大蒜,并将大蒜作为重要的出口农产品,大蒜规模化生产和产业化发展很快,许多地方也建立了大蒜专业网站,介绍大蒜生产、流通等信息和技术。
国内外用大蒜为原料制成的调味品、保健食品、医疗制品、化妆品和工业品随着市场需求的不断增长日益丰富,从而促进了大蒜生产的发展。
大蒜在食品工业、医药工业、化妆品工业、饲料工业以及农用杀虫、杀菌剂制造业等方面的应用前景愈来愈广阔。
大蒜的高产、高效及优质栽培技术在实现农业产业化,帮助农民脱贫致富,发展农村经济等方面都具有重要意义。
蒜头中微量元素硒的含量在蔬菜中是最高的,达到 0.276 微克/克,一般蔬菜的含硒量仅为 0.01 微克/克。
大蒜含有 0.2%的挥发油,内含蒜氨酸。蒜氨酸没有挥发性,也没有臭味,只有在切蒜时蒜氨酸在蒜酶的作用下才分解成有臭味的蒜辣素(大蒜素)。
大蒜的独特辛辣气味可以解除鱼、肉的腥味,增进食欲,是膳食烹调中不可缺少的调味品。
大蒜在兽医临床和饲料添加剂方面的应用,显示出广阔的开发前景。
将蒜头捣成泥或榨出汁,涂在牲畜的伤口上,可明显增加白细胞数,促进伤口愈合。
将大蒜剥皮后加等量的凉开水磨成浆,过滤后将蒜汁按 3%的比例加入饲料中,均匀搅拌,可防止饲料发霉,能预防畜禽胃肠道疾病的发生。
研制成大蒜素饲料添加剂,即在大蒜素饲料添加剂中加入微量元素碘,具有活血化淤、清瘟解毒、杀菌抑菌、促进生长等作用,饲喂效果明显。
大蒜对黄瓜和西瓜枯萎病及辣椒疫病的菌丝生长和孢子萌发都有极显著的抑制作用,大蒜鳞茎的粗提取液可有效防治这 3 种病害。
大蒜可减少血液中糖的含量,保护肝功能,提高血液中胰岛素含量,预防糖尿病。
大蒜还有阻断霉菌对亚硝胺合成的促进作用,减少因亚硝胺诱发癌症的可能性。
大蒜素又称蒜辣素,英文译名为阿利辛(Allicin),是一种含硫化合物。
蒜头的新鲜程度也会影响大蒜素的含量。采收后贮藏时间长,大蒜素含量降低。
大蒜不仅是民间广泛用以防疫治病的重要食品和药品,而且被提炼制成抗菌消炎的成药及保健品。
我国合成的大蒜新素(Allitridi)具有很强的抗菌消炎作用,已应用于临床。
一株完整的大蒜植株包括:根、鳞茎、叶鞘、叶身、花茎、总苞及气生鳞茎
大蒜的根是从蒜瓣基部的茎盘上发生的,为弦线状须根,称为不定根,没有主根。
蒜头和蒜瓣的基部都有一个扁平的盘状致密组织,称茎盘，它与植物正常的茎不同,属于茎的变态,又称变态茎。蒜头成熟以后,茎盘木质化,有保护蒜瓣、减少水分散失的作用,所以大蒜贮藏时要用完整的蒜头。
大蒜鳞茎盘顶部的生长锥分化为花芽后,逐步发育抽生蒜薹。蒜薹的下部为花茎,顶端着生花苞称总苞。总苞成熟后开裂,可以看到许多小的鳞茎,称气生鳞茎、空中鳞茎,俗称蒜珠或天蒜。
大蒜的叶片包括叶鞘和叶身两部分。叶鞘呈圆筒形,着生在茎盘上。每一片叶均由先发生的前一片叶的出叶口伸出,许多层叶鞘套在一起,形成直立的圆柱形茎秆,由于它不是真正的茎,故称“假茎”。
大蒜的叶片互生,对称排列。叶片的排列方向与蒜瓣的背腹连线垂直。所以,播种时应将蒜瓣的背腹连线与播种行的方向相平行,则出苗后叶片的排列方向就和播种行的方向垂直,这样一

来,叶片与叶片之间的遮荫减少,可以接受更多的阳光,增强叶片的光合作用。
大蒜在我国栽培的历史悠久,栽培地区遍及全国,各大蒜产区一般都有适应本地区生态条件的优良品种,因而了解大蒜品种的生态型对大蒜引种和栽培都有重要指导意义。
大蒜生态型的形成不但和纬度有关,而且在很大程度上受海拔高度的影响。
薹用大蒜的适宜播种期为 9 月下旬至 10 月上旬。行距 27~33 厘米,株距 6.5~7 厘米,或行距 17 厘米,株距 10 厘米,每 667 平方米 3 万~3.5 万株。
耀县火蒜,陕西省耀县地方品种。株高 85 厘米,株幅 44 厘米。假茎高 36 厘米,粗 1.4 厘米。薹长 46 厘米,薹粗 0.8 厘米,单薹重 17.3 克。为蒜薹和蒜头俱佳的品种。
吉木萨尔白皮新疆维吾尔自治区吉木萨尔县地方品种。因蒜头大、蒜瓣肥、皮色洁白、品质优良、产量高、耐贮藏而享誉国内外,为出口创汇的重要品种。
伊宁红皮新疆维吾尔自治区伊犁州伊宁县地方品种。株高约 90 厘米,株幅 43 厘米。假茎高 26 厘米,粗 1.6 厘米。单株叶片数 11~12 片,最大叶长 69 厘米,最大叶宽 3.7 厘米。抽薹率虽然可达 100%,但蒜薹短而细,产量不高。
昭苏六瓣蒜新疆维吾尔自治区昭苏县地方品种。单株叶片数 9 片,叶色深绿,叶片蜡粉较厚。蒜头近圆形,横径 5~6 厘米,外皮淡紫色。瓣型肥大而整齐,蒜瓣背宽达 2.2 厘米,蒜衣一层,紫褐色。
格尔木红皮蒜头近圆形,横径 6~7 厘米,外皮褐色带紫色条纹。平均单头重 76 克,大者可达 92 克。抽薹性较差,多数不抽薹,蒜头中央形成 1 个蒜瓣,少数为半抽薹。
江孜红皮蒜株高 79 厘米,株幅 23 厘米。假茎高 35 厘米,粗 1.2 厘米。单株叶片数 13 片,最大叶长 51 厘米,最大叶宽 2.1 厘米。蒜头扁圆形,横径 6~7 厘米,形状整齐,外皮灰白色带紫色条纹。蒜衣两层,紫红色,容易剥离。
拉萨紫皮蒜西藏自治区拉萨市郊地方品种。蒜头扁圆形,横径 7.5 厘米,外皮紫色,易破裂。平均单头重 108 克,大者达 127 克。有 8~20 个蒜瓣,一般为 11 瓣。平均单瓣重 10 克左右,大者达 14 克。蒜衣紫褐色。
下察隅大蒜西藏自治区下察隅地区地方品种。蒜头近圆形,横径 7.7 厘米,外皮紫红色。平均单头重 66 克,大者达 100 克。每头蒜有蒜瓣 9~10 个,蒜衣紫色。
拉萨白皮蒜蒜头扁圆形,大而整齐,外皮白色。平均单头重 150 克,大者达 250 克。每头蒜有蒜瓣 20 多个,蒜衣白色。耐寒、耐旱,抽薹率低。
清涧紫皮蒜蒜头扁圆形,横径 5 厘米左右,外皮灰白色带紫色条纹,平均单头重 30 克。每头蒜有蒜瓣 5~6 个,分两层排列,内、外层蒜瓣数及单瓣重差异不大,瓣形整齐,平均单瓣重 5.4 克。蒜衣一层,紫红色,不易剥离。
榆林白皮蒜株高 51 厘米,株幅 42 厘米。单株叶片数 7 枚,叶面蜡粉多。蒜头近圆形,横径 4.5 厘米左右,外皮白色,平均单头重 70 克。每个蒜头有蒜瓣 14~17 个,分 3~4 层排列,蒜瓣小而细长。蒜薹短小。
阿城紫皮蒜株高 84 厘米,株幅 32 厘米。假茎高 33 厘米,粗 1.3 厘米。单株叶片数 8 枚,叶色深绿,叶面有蜡粉。蒜衣一层,紫红色,不易剥离。抽薹率 90%以上,但蒜薹产量低。
白皮狗牙蒜，株高 83 厘米,株幅 18 厘米,株形较直立。假茎高 36 厘米,粗 1.2 厘米。蒜衣一层,淡黄色,不易剥离。抽薹率低,蒜薹细小,无商品价值。
开原大蒜辽宁省开原县地方品种。株高 89 厘米,株幅 34 厘米。假茎高 34 厘米,粗 1.4 厘米。单株叶片数 10~11 枚,最大叶长 60.5 厘米,最大叶宽 2.7 厘米，蒜衣一层,暗紫色,易剥离。
海城大蒜，品种行销黑龙江、吉林、内蒙古等地,还远销加拿大、罗马尼亚及日本等国。株高 75 厘米,株幅 47 厘米,株形较开张。叶片淡绿色,叶面有蜡粉。蒜头近圆形,外皮灰白色带紫色条纹。
应县大蒜有紫皮和白皮两种,以紫皮为主。植株长势旺盛,叶片深绿,有蜡粉，蒜头扁圆形,横径

5 厘米左右,外皮紫色，蒜瓣肥大而匀整,肉质致密,辛辣味浓,品质好。蒜衣紫红色。
民乐大蒜，假茎高 12 厘米,粗 1.2 厘米。单株叶片数 16 片,最大叶长 66.5 厘米,最大叶宽 2.4 厘米。蒜头近圆形,横径 5.2 厘米,形状整齐,外皮灰白色带紫色条纹,平均单头重 50 克。
临洮白蒜，8 月下旬采收蒜头，株高 95 厘米,株幅 24 厘米。假茎高 41 厘米,粗 1.1 厘米。单株叶片数 18 片,最大叶长 52 厘米,最大叶宽 2 厘米。
临洮红蒜，甘肃省临洮县地方品种。株高 73 厘米,株幅 28 厘米。假茎高 21 厘米,粗 1.5 厘米。单株叶片数 15~16 片,最大叶长 57 厘米,最大叶宽 2.8 厘米。
土城大瓣，株高 75 厘米,株幅 30 厘米。假茎高 15 厘米,粗 1.1 厘米。单株叶片数 8~9 枚,最大叶长 57 厘米,最大叶宽 2.6 厘米。蒜头近圆形,横径 4.6 厘米,外皮灰白色带紫色条纹，蒜衣一层,紫红色，当地春播夏收可抽薹。
银川紫皮，株高 65 厘米,株幅 25 厘米。假茎高 17 厘米,粗 1.7 厘米。单株叶片数 13~14 枚,最大叶长 49 厘米,最大叶宽 3 厘米。蒜衣两层,紫红色。当地春播夏收,抽薹性较差,薹细小,而且有半抽薹现象。
幼苗期大蒜生长主要依靠种瓣中贮藏的养分。在此期内,根系继续扩展,生长锥继续分化幼叶,幼叶逐渐长大,露出土面,进行光合作用,制造养分。
春播大蒜品种的花芽和鳞芽开始分化期处于温度逐渐升高、日照逐渐加长的春季,分化进程加快,花芽和鳞芽从分化开始到分化结束需要的天数较少,需 15~35 天。
大蒜鳞茎成熟后即进入休眠状态,这时即使给予适宜的温度和水分,鳞芽也不会萌芽和发根,这段时间称为大蒜的生理休眠期。
蒜头的单位面积产量=株数×蒜头平均重×商品蒜头百分率(%)
超过一定的密度范围后,蒜头的平均重量减轻,当蒜头重量的减轻不能用株数的增加而加以弥补时,单位面积产量则下降。而且栽植过密时,叶鞘细长紧实,不易抽出蒜薹,断薹率增多,影响蒜薹产量;栽植过稀时,大蒜头的比例增多,但形状不整齐的畸形蒜头也增多。
大蒜是要求中等强度光照的作物。光照过强时,叶绿体解体,叶组织加速衰老,叶片和叶鞘枯黄,鳞茎提早形成;光照过弱时,叶肉组织不发达,叶片黄化。
幼苗期要适当降低土壤湿度,防止苗子徒长,促进根系向纵深发展,避免蒜种因土壤过湿而提早腐烂,对幼苗生长造成不利影响。
退母结束以后,大蒜叶片生长加快,水分的消耗增多,需要保持较高的土壤湿度,促进植株生长,为花芽、鳞芽的分化和发育打基础。
大蒜叶片呈带状,较厚,表面积小,尤其是叶表面有蜡粉等保护组织,地上部具有耐旱的特征。因此,大蒜能适应干燥的空气条件,适宜的空气相对湿度为 45%~55%。在设施栽培中,因空气湿度大,很易诱发叶部病害。
如果在地下水位高而且排水不良的土壤上种蒜,抽蒜薹后要在 20 天之内就挖蒜头,否则易发生散瓣、烂瓣现象。
大蒜喜微酸性土壤,适宜的土壤氢离子浓度约为 1000 纳摩/升(pH 值为 6)。在碱性大的土壤中种蒜,蒜种容易腐烂,植株生长不良,独头蒜增多,蒜头变小。
葱蒜类有相同的地下害虫,主要是根蛆。重茬时虫口密度增加,危害的严重程度也随之增加。
葱蒜类蔬菜叶片狭长,叶面积小而且是直立生长,植株间通风透光良好,为杂草的滋生提供有利环境,所以是属于对杂草抑制能力弱的一类蔬菜。
春播蒜的前茬以豆类、瓜类及茄果类蔬菜较好,因为这些蔬菜的施肥量较多,土壤较肥沃,对大蒜生长有利。
秋播蒜和春播蒜的后茬多为夏秋菜。可以种植夏秋番茄、黄瓜等果菜以及大白菜、萝卜等秋菜。大蒜的根系分泌物有杀菌作用,同时蒜的吸肥量较少,土壤中残留的养分较多,所以是其他非葱蒜类蔬菜和农作物的良好前茬。

大蒜根系分泌物对病菌的繁殖有抑制作用,加上蒜、稻轮作田改变了病、虫生活的环境条件,可以减轻水稻病害和地下害虫(金针虫、蝼蛄等)的危害。
间作是指两种或两种以上作物,同时隔畦、隔行或隔株有规则栽种的种植方式。
春播地区大蒜的前作物多为豆类、瓜类及茄果类蔬菜。前作收获后深耕,耕后不耙耱,使土壤经过冬季冻结(称“冻垡”)与春季融化,土质变疏松。
大蒜播种期是否适当,对蒜薹和蒜头产量与品质都有很大影响。播种过早,由于温度高,蒜瓣发芽慢,出苗期长,幼苗在冬前生长过旺,抗寒力下降,生长后期下部叶片枯黄,植株早衰,从而减弱大蒜鳞茎肥大期的光合作用和养分的积累,使蒜头变小。
大蒜的种植密度应根据当地的综合条件,如品种特性、蒜种大小、土壤肥力及栽培方式等,通过田间试验确定。
大蒜瓣所含营养物质较丰富,播种后根系发达,植株生长旺盛,假茎较粗,叶片数较多,单株叶面积较大,蒜头较大,产量也较高,所以需要较大的空间和营养面积,可适当种稀些。小瓣蒜播种则应适当密植。
秋播大蒜和春播大蒜幼苗期所处的环境条件不同,幼苗期的田间管理也应有所区别，秋播大蒜不同品种间幼苗期的长短有很大差异。
秋播大蒜在有条件的地区可在灌越冬水后覆盖草粪或豆叶,保墒保温,以利于幼苗安全越冬。
大蒜花芽和鳞芽的开始分化期与品种习性有密切关系,但同一品种的花芽和鳞芽分化结束期却很相近,所以不同品种间这一生育期的长短仍有较大的差异。
秋播大蒜于翌年春暖返青后,结合灌水施返青肥,为幼苗返青后的旺盛生长提供充足的水分和营养，秋播大蒜种的极早熟和早熟品种以及南方冬季比较温暖的地区,可提早追肥和灌水。
花茎伸长期田间管理的重点是抓紧追肥和灌水,以满足花茎生长的需要,并为蒜头的肥大打下基础,防止蒜头肥大期缺肥及植株早衰，采收蒜薹前 3~4 天停止灌水,以免蒜薹太脆,采收时易折断。
南方在鳞茎肥大期常遇多雨天气,土壤湿度大,容易引起散瓣,影响蒜头质量,应注意开沟排水,降低土壤含水量。
拉抽法一手捏住蒜薹上部距假茎口 3~5 厘米处,斜向往上拉直蒜薹,用力要均匀,待蒜薹在假茎中断裂,发出轻微响声时,迅速抽出蒜薹，但如果遇到阴雨天气,空气及土壤湿度大时,断薹较多,蒜薹产量降低。
夹抽法用一根直径为 1~1.5 厘米、长 40~50 厘米的新鲜竹竿,削平节部突起,用火将竹竿中部烤软,对折,固定 2 天,做成有弹性的夹子。采薹时一只手捏住薹的上部,另一只手用夹子将地面 2~3 片叶间的假茎处夹一下,使蒜薹断裂而假茎不断,然后将植株上部两片叶的叶鞘口撕开,徐徐抽出蒜薹。
夹抽法的断薹率较抽薹法低,蒜薹产量较高,且蒜薹上无损伤,适合贮藏。但蒜薹粗长而且下粗上细的品种,断薹率仍偏高,而且采薹后植株易从夹薹部位倒伏。
扎抽法将竹筷子的细头或木条的先端用刀削成尖锥形,也可以将竹筷或木条先端一侧削一凹口,在凹口中心处钉入一个半截大针,针的断面一端烧红砸扁成铲状,用原工具凹口处的针尖,从有叶身的一侧(俗称“小里面”)将假茎上部两三个叶鞘顶端划开,便可抽出。
扎抽法的优点是:蒜薹无划伤,断面齐,采薹后假茎不倒伏,叶片较完整,蒜头产量高。此法适用于稀植、假茎短粗且上下部粗度差异小、采薹期较早的早熟品种。
划抽法采薹工具用一根直径约 1.5 厘米、长约 15 厘米的木棍,距木棍一端约 3 厘米处横划一个半圆形的槽,在槽的中央钉一个短而尖的钉子或一个半截针,使针尖刺入假茎内,并迅速向上划至梢部。叶鞘全部划开后再将蒜薹从基部折断。
划抽法的优点是采薹速度快,不易断薹,蒜薹长,产量高。但由于采下的蒜薹上多带有一道纵向伤痕,易腐烂,不耐贮运。另外,由于采薹时叶鞘被完全划破后植株倒伏,叶片很快干枯,鳞茎肥

大期叶片的光合作用被严重减弱,导致蒜头产量下降。
无论采用何种采薹方法,采薹均应选在晴天下午进行,这时植株体内水分较少,质地较软,弹性增加,薹不易被折断。
蒜种处理早蒜苗的播种期处于 7~8 月份高温季节,种瓣的休眠期尚未完全结束,田间的高温也对蒜瓣的发芽不利,如果播种前不经过特殊处理,播种后长期不发芽,待温度下降,种瓣发芽出土后,蒜苗生长也不健康。
晚蒜苗的播期较晚,种瓣已度过休眠期,田间温度已下降,不需要进行潮蒜处理。
田间管理早蒜苗播种后,当地面略干时,用耙子碎土保墒,以利于出苗。大部分幼苗出土后轻浇一水,促进全苗。
晚蒜苗冬前田间管理同蒜薹和蒜头栽培。为确保安全越冬,应在灌冻水后覆盖圈粪或藁草。
蒜苗保护地栽培的形式多样,各地也不尽相同。主要形式有地栽和床栽 2 种形式:地栽即与露地蒜苗栽培方式一样,在土壤中播种蒜瓣,生长蒜苗并带根采收;床栽即将整蒜头直接播入沙子、沙土或混合基质中,生长并割收蒜苗。床栽结合轻基质的使用,可做成多层架栽培。
春播大蒜地区为了在冬季和早春供应蒜苗,多采用加温温室或日光温室栽培。
品种选择温室栽培蒜苗,由于要采取高度密植的方法提高单位面积产量,所以应选择蒜头大、休眠期短、生长迅速、假茎长、不易倒伏、蒜瓣多而均匀的白皮蒜品种作种瓣。
栽蒜苗的生长主要靠蒜瓣中贮藏的营养,一般不需要施肥,只要供给适宜的温度和水分,就可以生产出鲜嫩的蒜苗。地栽蒜苗,根据生长期的长短,一般需追肥 1~2 次。
遮光软化待大部分蒜种的初生叶出土后,在栽培池上覆盖黑色塑料薄膜或双层草帘遮光,四周埋严。如覆盖时间晚或盖不严,则叶鞘变为绿色,品质降低。
灌水摆蒜后立即灌水。幼苗期需水量小,可根据室内温度及天气情况,灌少量水或不灌水。蒜黄生长盛期需水量加大,可适当增加灌水量。
温度摆蒜后,如温度过高,蒜黄生长虽快,但组织太柔嫩,易倒伏、腐烂,使产量和品质下降。
生产独头蒜所用的种瓣必须是小蒜瓣,所以一般多从蒜瓣较多而蒜瓣较小的大蒜品种中选择。
生产独头蒜的种瓣大小,关系到独头蒜的产量和品质。选择大小适宜的蒜瓣作种蒜,才能获得高的独头率和大小适中的独头蒜。
独头蒜的适宜播期必须在当地做分期播种试验才能确定。播早了,蒜苗的营养生长期长,积累的养分较多,易产生有 2~3 个蒜瓣的小蒜头。
大蒜不能正常结种子,生产上长期采用蒜瓣进行无性繁殖,从蒜瓣到蒜瓣,不经过授粉受精产生种子的有性世代,则不能产生生活力强的后代,因而易导致品种退化。
植株长势减弱,病毒病严重,蒜头和蒜瓣逐渐变小,产量逐年下降乃至丧失商品价值。很多大蒜产区的主栽品种,不同程度存在着品种退化问题,从而限制了大蒜生产的进一步发展。
生产大蒜的名产区必定是具备某些品种生育最佳的气候、土壤、地形、地势条件及栽培技术的地区。
生产蒜头为主要目的的品种,其选种目标为:蒜头大而圆整,蒜瓣数符合原品种特征,瓣形整齐,无夹瓣,质地致密脆嫩,含水量低,黏稠度大,蒜味浓,耐贮运。
生产蒜薹为主要目的的品种,其选种目标为:抽薹早而整齐,蒜薹粗而长,纤维少,质地柔嫩,味香甜,耐贮运。
以生产蒜苗为主要目的的品种,其选种目标为:出苗早,苗期生长快,叶鞘粗而长,叶片宽而厚,质地柔嫩,株形直立,叶尖不干枯或轻微干枯。
播种期较生产田推迟 10~15 天。迟播的蒜头虽较早播者稍小,但蒜瓣数适中,瓣形较整齐,可用作种瓣的比例较高。
过大的种瓣容易发生外层型二次生长;过小的种瓣生产的蒜头小,蒜瓣少,有时还会发生内层型二次生长。二者都导致种瓣数量减少,质量下降。

早抽蒜薹,改进采薹技术。当蒜薹伸出叶鞘口,上部微现弯曲时,采取抽薹法抽出蒜薹,尽量不破坏叶片,使抽薹后叶片能比较长时期地保持绿色,继续为蒜头的肥大提供营养。

一般在幼苗期、抽薹期、蒜头收获期、贮藏期及播种前各进行 1 次。根据生产目的,各时期的选优性状标准要明确、稳定。

大蒜是无性繁殖植物,连年利用蒜瓣繁殖,使病毒病逐年积累并代代相传,致使种性退化,产量和品质逐年下降。

我国从 20 世纪 80 年代初即开始研究大蒜的脱毒技术,取得了一些进展。,已基本解决了实验室大蒜茎尖培养脱毒苗、温室繁育脱毒母种、网室扩大繁育脱毒原种及种子田繁育生产用种的一系列技术措施

由于大蒜花叶病毒病是由多种病毒复合侵染所致,而用茎尖分生组织脱毒的效果因病毒种类的不同而异,所以在检测脱毒效果时,应当制备大蒜复合病毒抗血清,以常规酶联夹心法检测脱毒效果比较准确可靠。

幼苗生长到一定程度后,基部膨大形成小鳞茎,小鳞茎成熟后,地上部干枯,可收获贮藏,这就是脱毒原原种,又称当代或零代鳞茎。

茎尖脱毒、病毒的检测、脱毒原原种的室内快速繁殖、脱毒原种的扩大繁殖乃至种子田生产种的扩大繁殖,需要一定的仪器设备和成熟的配套技术,目前在农村尚难以推广,因此一些主要大蒜产区如能建立脱毒蒜种产销机构,蒜农便可直接购买生产种用于大田生产。

侵染我国大蒜的病毒有很多种,其中最主要的是大蒜花叶病毒(GMV)和洋葱黄矮病毒(OYDV),因此在脱毒大蒜田周围不能种植洋葱等葱属作物,以免交叉感染。

田间采用物理方法诱蚜、避蚜及喷农药灭蚜,消灭传毒媒介。具体方法参见本书病虫害防治部分，购买脱毒生产种时最好选购增产效果较显著的一代生产种。

脱毒生产种用于大田生产后,由于再度感染病毒病,增产效果会逐年下降,所以需要更新。一般每 3 年更新 1 次,重新购进一代或二代生产种。

增施有机肥和磷、钾肥,适当控制速效性氮肥的施用。因为脱毒生产种的生长势强,植株高大,如果速效性氮肥施用过多,施用期偏晚,植株生长过旺,容易发生“二次生长”,降低商品蒜头和蒜薹的产量和品质。

田间有蚜虫、蓟马、线虫及瘿螨等许多传毒媒介,又可将病株中的病毒传给健康的植株。因此,病毒传染率不断扩大,导致大蒜严重减产。

只有识别病毒病在田间植株上的种种带毒症状,才能淘汰病株,减少毒源,降低大蒜带毒率。这是做好大蒜病毒病防治工作必备的知识。

被病毒侵染的蒜头,当年并不表现带毒症状,而是在播种后长出的幼苗上或成年植株上表现出病毒病症状,因此要及早清除田间带病毒植株。高温干旱、植株生长不良时,症状严重，水、肥充足,植株生长健壮时,症状较轻。

防治方法: 第一,采用脱毒大蒜生产种, 消灭大蒜植株生长期间及蒜头贮藏期间的传毒媒介。

大蒜田周围不要种植其他葱属作物,如大葱、洋葱、韭菜等，实行 3~4 年轮作,避免与大蒜及其他葱属作物连作,播种前严格选种,淘汰有病、虫的蒜头,再将选出的种瓣用 80%敌敌畏乳剂 1000 倍液浸泡 24 小时,以消灭螨类。

从幼苗期开始,对种子田进行严格选择,及时拔除发病植株,以减少病害传播,加强大蒜生产田的水、肥管理,培育健壮植株,增强抗病力。

病原病原菌属半知菌亚门葡萄孢属葱鳞葡萄孢菌真菌。孢子梗顶端多分枝,枝梗顶端膨大,其上着生分生孢子，分生孢子卵形至椭圆形,透明，田间病叶上大量形成不规则形、黑色、坚硬的菌核。

传播途径及发病条件：大蒜灰霉病病菌主要以菌核在土壤中越过冬、夏,以后借散落在土壤表层或附着在病株残体上的灰霉(分生孢子梗和分生孢子)在田间传播蔓延。风雨、流水、工

具及田间操作都可以成为病菌传播的媒介。
大蒜灰霉病的发生除了与品种抗性有关外,还受气候、土质及栽培技术的影响。春季降雨多,田间湿度大;土质黏重,透水性差;种植密度过大,田间通风透光不良;播期过晚,苗子长势差;偏施氮素化肥,植株抗病力减弱等有利于病菌繁殖和传播。
在冷库中贮藏的蒜薹,当库温变化大、塑料袋中因湿度大结成水滴时,也易发生灰霉病。
苍山大蒜中的高脚子品种抗性较强,其次是蒲棵和鲁山白、二水早、嘉定白蒜、安丘大蒜。感病品种有糙蒜和苏联红皮蒜二号。
选择地下水位低、土壤排水性良好的地段种植。整平畦面,设排水沟,防治田间积水, 施用有机肥作基肥,增施磷、钾肥,避免过量施用氮肥及灌水,合理密植,防止植株徒长。
贮藏蒜薹的冷库和其中的货架,在使用前喷 1%~2%福尔马林溶液或 0.3%~0.5%漂白粉溶液消毒。
叶枯病不但危害大蒜,而且危害大葱、洋葱、韭菜等葱蒜类蔬菜。在国内分布广,危害重。
叶枯病主要危害叶片和蒜薹,多从下部老叶尖端开始发病。发病初期病斑呈水渍状,叶色逐渐减退,叶面出现灰白色稍凹陷的圆形斑点。
以菌丝体或子囊壳在病株残体或土壤中越冬。借风、雨、流水、种瓣、肥料、人、畜、工具等媒介传播。叶枯病病菌为弱寄生菌,生长健壮的植株不易染病。常与紫斑病同时发生。雨量多、湿度大、气温高、缺肥、植株早衰的田块发病较严重。
病菌主要危害葱蒜类蔬菜的叶部,大葱和洋葱的花梗也易染病。最初在叶或花梗上出现白色、稍凹陷的小斑点,病斑中央呈浅紫色。以后病斑很快扩大为椭圆形或纺锤形,紫色,稍凹陷。
病菌以菌丝体或分生孢子附着在病株残体上及土壤中越冬,或直接以分生孢子越冬。分生孢子萌芽后,借风、雨从植株叶片的气孔或伤口入侵。阴雨多的年份发病较重。
大蒜白腐病是甘肃省天水市及陇南地区蒜田的主要真菌性病害之一,自 1992 年以来危害日趋严重,秋播蒜区也有发生。在陕西关中蒜区,近年大蒜白腐病已成为主要病害之一,连作田块出现成片死苗,损失较大。
白腐病病原菌以菌核在土壤和病株残体内越冬,翌年春季菌核萌发产生菌丝,借雨水或灌水传播,再侵入大蒜组织,造成根系变软腐烂或组织干腐。但田间病株上的新菌核,当条件适宜时可不经休眠,继续萌发产生菌丝,形成再侵染。
防治方法第一,避免连作,实行 3~4 年轮作; 选择地下水位较低、排水良好的地块种植大蒜,加强松土保墒,降低地表湿度。
大蒜在烂母时期发出特殊臭味,招致种蝇和葱蝇在表土中产卵,所以大蒜在烂母期受害最严重。
根螨分布广,繁殖快,为害重,是大蒜田间及贮藏期间的危险害虫。成螨体长 0.58~0.81 毫米,宽卵圆形,似洋梨状,表面白色,光滑发亮,有 4 对短而粗的足。
以成螨及若螨为害贮藏的蒜头,有时田间蒜头也可受到侵害。多从蒜头的茎盘边缘缝隙处入侵,在蒜瓣基部的肉质部刺吸汁液,以后逐渐转移到蒜瓣的尖端部为害,使蒜瓣逐渐萎蔫、变褐、干枯,蒜头成为空壳。
瘿螨还是病毒病的传毒媒介,带毒量大,传毒快,毒期长,危害重,对大蒜的传毒率达 100%。凡被瘿螨为害过的大蒜,播种后长出的幼苗呈现多种病毒病症状,生长缓慢,不抽薹,不形成蒜头,或形成独头蒜。
蒜头贮藏期间如发现螨类为害时,可用硫磺粉熏蒸。每立方米空间用硫磺粉 100 克,加入少量锯木屑,拌匀后装在容器中,放在蒜头贮藏室内,点燃后将门窗封闭,熏蒸 24 小时,杀螨效果达 100%。但对卵无效,可待卵孵化后再熏蒸 1 次。
播种前严格选种,淘汰有病、虫的蒜瓣,再将入选种瓣用 80%敌敌畏乳剂 1000 倍液浸泡 24 小时,杀螨效果可达 100%, 掰蒜后剩下的蒜皮、蒜根、蒜薹残桩及茎盘要全部集中烧毁,以减少侵染源。
线虫主要在蒜头中越冬,或在贮藏的蒜头中继续繁殖后代。其传播方式可通过受害种瓣传播

繁殖后代;也可以土壤为传播源,借助水流传播繁殖后代。
马铃薯茎线虫分布范围广,为世界性害虫,除为害葱蒜类蔬菜外,还为害马铃薯、胡萝卜、萝卜、甜菜及甘蓝等。
马铃薯茎线虫以成虫及幼虫从大蒜植株茎盘的生根部位侵入,在鳞茎盘周围繁殖为害。被害大蒜植株靠近根部组织带有稍凹陷的灰褐色或黄褐色病斑,然后植株下部叶片黄化,假茎变软腐烂,最后植株变黄枯死,造成田间缺苗断垄。
大蒜粪蚊以蛹或老熟幼虫在土壤或被害蒜头中越冬。成虫在大蒜植株根部土壤表层内产卵,多数产卵成堆,少数散产。
大蒜生长期间应加强除草、松土,使植株根际周围的表土干燥,抑制虫卵孵化及幼虫活动。
大蒜播后出苗慢,苗期长,加上叶片窄长,地面长期不能形成荫蔽状态,从而给杂草的滋生创造了有利条件。
使用化学除草剂是防除大蒜田间杂草的有效途径，理想的蒜田化学除草剂应具备的条件是:低毒,无残留,安全,成本较低;能兼除阔叶草、禾草和莎草;播种后发芽前至全生长期,只要避开大蒜 1 叶至 2 叶期,均可使用;对与大蒜间作套种的作物无毒害作用。
蒜薹和蒜头生产的季节性强,收获期集中,如不采取有效的贮藏保鲜措施,不但满足不了消费者的需求,而且会给生产者和经营者造成重大的经济损失。
研究、推广蒜薹和蒜头贮藏保鲜技术,对调节市场供应,促进大蒜生产发展,有重要作用。
蒜薹的收获期多集中在 4 月份,春播地区蒜薹的收获期多集中在 6 月份。收获后,除少部分在当地及时销售外,大部分需要贮存起来以调节当地和外地蔬菜市场供应。
蒜薹入窖后,每隔 6~7 天要进窖检查 1 次,如发现冰块间有空隙,要扒开隔热材料用碎冰填好。同时,要经常检查冰窖外面的排水井,如果排水井的渗水口经常滴水,表示窖内冰块没有融化,滴水系土壤中水分渗出造成的。
采收后的蒜薹要仔细挑选,淘汰有病斑、虫伤、划伤、霉烂及总苞膨大变白的蒜薹。
收获后的蒜薹仍然是有生命的活体,继续进行呼吸,体内积累的养分通过呼吸作用被消耗掉。所以,采收后如果呼吸作用强,养分消耗快而多,就会使蒜薹的品质迅速变坏,乃至腐烂。
收获后的蒜头已进入休眠期,蒜瓣中的发芽叶停止生长。秋播地区在适温下贮藏的蒜头,一般在 9 月份以内基本上可以保持原有品质。
我国是大蒜出口大国。外商除了对蒜头规格有严格标准外,还要求延长供货时间,从蒜头采收后到年底,甚至再长一些时间都有新鲜蒜头出口。
不同大蒜品种间,耐贮性有差异。以各品种芽瓣比的大小,表示其耐贮藏性的强弱,可划分为 4 类：不耐藏，较不耐藏，较耐藏，耐藏。
蒜头收获期不但影响蒜头产量和品质,而且关系到贮藏期的长短和蒜种品质的好坏。收获太早,蒜瓣中的水分容易散失,另一方面,收获太早时,蒜瓣中的发芽叶发育不充分,播种后出苗率低。
蒜头必须选晴天收获,而且在收获前后最好各有 3 天的晴天,则挖蒜时蒜头外皮完整,而且晾晒时可迅速干燥,以提高贮藏质量。
蒜头收获后,切断假茎后干燥或连带假茎一同干燥对贮藏期间蒜头重量的变化也有影响。带假茎干燥的蒜头比切去假茎干燥的蒜头重量和直径都比较大。
蒜头收获后在田间干燥期间如遇雨,则外皮颜色发乌、长霉,如果移至通风不良的室内阴干,还会引起蒜头腐烂。
,大规模生产时应有通风干燥设备,在 50℃温度下干燥 12 小时,可提高蒜头贮藏质量。
秋播地区,在室温贮藏条件下,一般当进入 8 月份后休眠期结束,蒜瓣中的发芽叶和茎盘上的根开始生长。以后随室内温度的下降,生长加快。因此,要想延长贮藏期,必须抑制发芽叶和控制根生长的条件。

蒜头贮藏场所的空气相对湿度以 70%~75%为宜。湿度过高,蒜头易发霉;湿度过低,蒜头易失水。
常温贮藏是在自然温度状态下的贮藏方式,其优点是简便易行,成本低,是我国蒜农常用的贮藏方式。但贮藏期较短,而且易发生虫蛀和霉变。
冷藏是指利用人工降低贮藏环境的温度或利用自然低温条件,抑制蒜头的呼吸作用,减少养分和水分损失,防止病虫繁殖蔓延,以延长贮藏保鲜期。
蒜头下窖的时期应选在田间地面刚结冻时。下窖太早,蒜头易受热;下窖太晚,蒜头易受冻。下窖的时间应在下午 4 时后,以免窖内温度升高。
大蒜的加工产品多种多样,包括食品、医疗保健品及畜禽饲料添加剂等。大蒜食品有干制、盐渍、糖渍、糖醋渍、饮料、酱、酒、油及调味品等。
糖醋蒜多选用蒜瓣数少、瓣形整齐的白皮蒜品种,于抽蒜薹后半个月左右采收蒜头。采收过早,蒜头未充分成熟,腌制时容易失水干缩;采收过晚,蒜头易散瓣。
蒜粉加工可以与脱水蒜片加工相结合。烘干后的蒜片,精选出符合质量标准的蒜片后,可将剩下的碎片用粉碎机磨成蒜粉,然后再用 80~100 目筛网过筛,即得蒜粉。
大蒜中的蒜氨酸本来是没有臭味的,当切碎或捣烂时,蒜的细胞被破坏,细胞中的蒜酶会活化,将蒜氨酸分解,产生大蒜素。大蒜素很不稳定,易被分解产生多种烯丙基硫化物,有强烈的蒜臭味。
大蒜辣椒酱是利用大蒜和辣椒自身含有的杀菌成分,抑制微生物的活动,延长产品的保质期。由于不加防腐剂,不经高温灭菌,原料中的各种营养成分能够较好地保存下来,所以是一种纯天然的调味食品。
大蒜生产中存在的问题很多,有的属于生理异常现象,有的属于栽培技术不当;有的带普遍性,有的则是在少数地区、少数年份发生。其中发生最普遍、对大蒜生产影响最大的问题是二次生长,其他还有裂球散瓣、抽薹不良、葱头蒜、叶尖枯黄、瘫苗、管状叶、开花蒜、变色蒜及棉花蒜等。
大蒜二次生长是蒜头收获前蒜瓣就萌发生长的异常现象。国内外对这种现象采用的名词很多,笔者认为,二次生长的植物学概念比较明确,为避免名词太多造成的混乱,统一采用二次生长一词比较好。
大蒜植株外层叶片的叶腋中萌生一至数个鳞芽,鳞芽延迟进入休眠而继续分化和生长,形成独瓣蒜,或没有花薹的分瓣蒜,或有花薹的分瓣蒜,结果在蒜头的外围着生一些排列错乱的蒜瓣或小蒜头,使整个蒜头成为畸形。
大蒜植株内层叶片的叶腋中,正常分化的鳞芽延迟进入休眠,鳞芽外围的保护叶继续生长,从植株的叶鞘口伸出,形成多个分杈。
,大蒜产区对当地的大蒜品种或引进外地品种时,在了解其丰产性和商品性的同时,还应了解其二次生长情况,尽量选择不易发生二次生长,特别是不易发生外层型二次生长的品种。
研究蒜瓣大小与二次生长的关系时,首先应了解品种的二次生长类型,并综合考虑蒜种贮藏条件、种植密度等因素,根据生产目的选用适当大小的蒜瓣播种,以达到产量和质量的统一。
大蒜二次生长发生的程度,在不同年份往往有很大的差异。气候包括温度、降水、空气湿度、日照等因素。
秋播大蒜的播种期应比不覆盖栽培的推迟 5~10 天,使苗期生长不过旺,使花芽和鳞芽分化后处于温度持续升高和日照时间逐渐加长的环境中,以利于花薹和鳞芽的正常发育。
春播大蒜的播种期应比不覆盖栽培的适当提早,使生育的各个时期提前,使分化后的花芽和鳞芽在高温、长日照条件来临以前有较充分的生长时间,以后在高温、长日照条件下顺利抽薹和形成鳞茎。
秋播大蒜早春返青后,要浅中耕;蒜头肥大期应停止中耕,以免损伤蒜头外皮。蒜头收获前半个月左右浇水过多或降雨过多或排水不良时,由于土壤湿度大,地温又高,蒜头外皮容易腐烂,造

成裂头散瓣。所以,收获前应根据土壤墒情和天气情况,适当控制灌水,并做好开沟排水工作,降低土壤湿度。

过早抽取蒜薹或抽蒜薹时蒜薹从基部断裂,造成蒜头中间空虚,也容易散瓣。

蒜头采收过迟,蒜头外皮少而薄,特别是当土壤湿度大时,外皮易腐烂,茎盘易枯朽,可造成裂头散瓣。

要掌握蒜头成熟期标准外,蒜头收获后应及时将根剪去,则残留在茎盘上的根段在干燥过程中呈米黄色,而且坚实紧密,对茎盘起保护作用,不易散瓣。

蒜头收获后遇连阴雨无法晒干时,如果堆放在室内,茎盘易霉烂,造成散瓣。量少时可将大蒜植株移至室内,蒜头朝上摆放在地上晾。量多时可将蒜头朝下摆在秝秸架上,上面用苫席和防雨布遮盖,周围挖排水沟,待雨停后立即揭席通风。

蒜头经晾晒后移至室内挂藏时,如果过于拥挤,而且离地面又近,在多雨季节蒜头会返潮,茎盘发霉腐烂,引起裂头散瓣。

大蒜的抽薹性主要取决于品种的遗传性,有完全抽薹、不完全抽薹及不抽薹品种之分。但有时原来是完全抽薹的品种,却出现大量不抽薹或不完全抽薹的植株,这是由于环境条件不适或栽培措施不当造成的。

大蒜植株基部不膨大形成蒜头,或膨大形成结构类似洋葱鳞茎的松软蒜头,故名“葱头蒜”。

蒜种贮藏期间或播种后没有经历足够天数的低温以及栽培管理粗放,苗生长瘦弱,鳞芽不能分化,不形成蒜瓣,从而产生“葱头蒜”。

防止或减轻叶尖枯黄的根本途径是:选择地下水位较低,排水良好的砂壤土种蒜;实行高畦栽培,加深耕土层,促进根系发达;维持比较稳定的土壤湿度,避免忽干忽湿以及保证“烂母期”的养分供应。

大蒜的正常叶片是狭长而扁平的,管状叶则呈中空的管状,形似葱叶，管状叶发生的叶位愈向下,被套在管状叶中的叶片数愈多,使正处在鳞茎肥大期的植株,减少了制造养分的器官,导致蒜薹、蒜头产量和质量显著下降。

蒜种在室温下贮藏,避免长期处于 15℃以下的冷凉环境中;选用中等大小的蒜瓣播种;适期晚播;保持适宜的土壤湿度,避免长期缺水。一旦发现管状叶,可及时划开,以消除或减轻对蒜薹和蒜头的不利影响。

蒜头外皮破裂,蒜瓣上部向外裂开,似开花状。在鳞茎肥大期,锄地时如将假茎的地下部分或蒜头的外皮损伤,则蒜瓣肥大时产生的压力使蒜头上部的外皮破裂,蒜瓣间产生空隙,然后上部向外裂开。

新鲜蒜头,如果假茎基部受伤破裂,以后在贮藏期间也会发生“开花”现象。所以,在鳞茎肥大期锄草时,要特别注意,避免损伤蒜头;在收获、晾晒及整理过程中也要避免假茎基部受损伤。

大蒜在贮藏期间,有些蒜头外观完好但内部蒜瓣干缩变黑,整个蒜头成为空包,俗称“棉花蒜”。

选择适合大蒜田的除草剂，注意剂型和使用浓度，避免对大蒜产生药害。

在大蒜播种前进行土壤处理，使用封闭性除草剂，减少杂草萌发。

播种后及时进行覆膜，抑制杂草生长，保持土壤湿度，定期进行中耕松土，破坏杂草根系，促进大蒜生长。

早期发现杂草，及时进行人工拔除，防止杂草扩散，在大蒜生长期间，合理使用除草剂，避免对大蒜产生不良影响。

采用轮作制度，减少田间杂草种类和数量，选择抗杂草的大蒜品种，降低除草难度。

合理密植，增加大蒜对土壤的竞争力，抑制杂草生长。注意天气预报，避免在雨后或大风天气进行除草剂喷施。

使用生物除草剂，如草甘膦，减少化学农药对环境的污染。结合机械除草，提高除草效率，减轻劳动力负担。

在杂草种子成熟前进行除草，防止杂草种子散布，对于顽固杂草，可多次施用除草剂，确保除草效果。
清除田边杂草，减少杂草向田内扩散的机会，根据田块实际情况，调整除草剂种类和用量。
使用选择性除草剂，减少对大蒜的伤害，在大蒜田周边种植覆盖作物，抑制杂草生长。
合理安排施肥时间，避免刺激杂草生长，对于恶性杂草，可采取点对点涂抹除草剂的方法。
选择排水良好、土壤肥沃的田块进行大蒜种植，确保根系发达，播种前进行土壤消毒，减少病虫害的发生。
选择优质、抗病的大蒜品种，提高产量和品质，播种前进行种子处理，如浸泡、晾晒，提高发芽率。
播种时保持适当的深度和行距，利于植株生长和通风，播种后及时浇水，促进种子发芽和生根。
生长期间保持土壤湿润，避免干旱或积水。合理施肥，以氮、磷、钾为主，注意微量元素的补充。
生长初期进行间苗，确保合理密度，利于光合作用，防治地下害虫，如地老虎、蛴螬，保护大蒜根系。
定期检查大蒜叶片，发现病虫害及时防治，采用生物防治方法，如释放天敌昆虫，减少化学农药使用。
避免连作，实行轮作制度，降低土传病害风险，适时中耕松土，提高土壤透气性，促进根系生长。
遇到极端天气，及时采取防护措施，如遮阳、防寒，合理安排种植时间，确保大蒜在适宜季节生长。
生长后期控制浇水，促进蒜头成熟。适时收获，避免蒜头过老或过嫩，影响品质。
收获后及时晾晒，防止蒜头发霉，储存大蒜时保持干燥、通风，避免受潮。
选择适应当地气候的优质品种，如紫皮蒜（辛辣味浓）或白皮蒜（耐寒性强）。检查蒜种无病虫害、鳞茎饱满，推荐使用脱毒种蒜，增产效果显著。
选择前茬未种葱蒜类的沙壤土，pH 值 6.0-7.0 最佳。深耕 25-30 厘米，清除杂草碎石，确保土层疏松透气，避免积水。
每亩施腐熟有机肥 3000-4000kg，配施三元复合肥（15-15-15）30kg。肥料均匀撒施后翻耕，避免烧根。
播种前掰瓣，剔除霉变、损伤蒜瓣。用 50%多菌灵 500 倍液浸种 30 分钟，晾干后播种，预防真菌病害。
北方秋播一般在 9 月下旬至 10 月上旬（地温 15-20℃），春播 3 月上中旬；南方可延至 11 月，确保越冬前长出 3-5 片真叶。
行距 20cm，株距 10-12cm，亩植 2.5-3 万株。大瓣蒜可适当稀植，小瓣蒜加密，播种深度 3-5 厘米，尖头朝上。
推荐黑色地膜覆盖，保温抑草。膜要拉紧贴地，播种后立即覆膜，幼苗出土时及时人工破膜引苗。
播种后浇透水，幼苗期保持土壤湿润。抽薹期需水量大，采收前 7 天停水，整个生育期浇水 4-6 次，忌大水漫灌。
返青期亩追尿素 15kg，抽薹期施高钾复合肥 20kg。叶面喷施 0.2%磷酸二氢钾 2-3 次，增强抗逆性。
幼苗期浅中耕 2-3 次，配合人工除草。覆膜田可减少除草次数，露地栽培可在播后苗前喷施二甲戊灵封闭除草。
实行 3 年以上轮作，避免与葱、韭菜等百合科作物连作。前茬优选豆科、禾本科作物，减少

土传病害积累，提高土壤肥力。
连作田建议播前用石灰氮（每亩 50kg）或棉隆（30kg/亩）处理土壤，覆膜熏蒸 15-20 天，有效杀灭线虫和病原菌。
按蒜瓣大小分级播种：一级瓣（＞5g）、二级瓣（3-5g）、三级瓣（＜3g）。分级种植确保出苗整齐，便于管理。
沙质土播种深度 5-6cm，黏土 3-4cm。过深影响出苗，过浅易受冻或干旱。播后轻镇压保墒。
北方秋播后覆盖秸秆或腐熟粪肥 2-3cm，保温防冻。越冬前浇封冻水，避免干冻伤苗。
春播地区遇倒春寒，可搭小拱棚或临时覆盖无纺布，气温稳定 5℃以上时撤除。
若幼苗徒长，可喷施 5%烯效唑 1000 倍液控旺，或减少氮肥用量，防止后期倒伏。
大风天气检查地膜压实情况，破损处及时用土封堵。生长中期可揭膜中耕，改善土壤透气性。
推荐采用滴灌系统，节水 30%以上。滴灌带间距 40cm，滴头流量 1-1.5L/h，生育期灌水 8-10 次。
低洼田块开挖排水沟，沟深 30cm。雨后及时排涝，避免积水超 2 小时导致烂根。
选用抗逆性强、产量高的脱毒大蒜品种，如鲁蒜、豫蒜等，确保种瓣饱满无病害，适应本地气候条件。
选择土层深厚、排水良好的沙壤土，pH 值 6.0-7.5，深耕 25-30cm，清除前茬残株以减少病虫害。
播种前用 50%多菌灵 500 倍液浸种 2 小时，晾干后掰瓣，去除盘茎并分级，确保种瓣大小均匀。
北方 9 月下旬至 10 月上旬，南方 10 月下旬至 11 月上旬，地温稳定在 18-20℃时播种最佳。
行距 20cm，株距 10-12cm，亩植 2.5-3 万株，大瓣种略稀，小瓣种略密，保证通风透光。
覆土厚度 2-3cm，过深出苗迟，过浅易冻害，播后轻镇压使种瓣与土壤紧密接触。
每亩铺设滴灌带 2-3 条，滴头间距 20-30cm，流量 1.5-2L/h，确保水分均匀分布。
亩施腐熟有机肥 3000kg+复合肥（15-15-15）40kg，深翻入土，避免肥料直接接触种瓣。
播后立即滴灌 15-20m³/亩，促进出苗整齐，土壤湿度保持 60-70%田间持水量。
3 叶期滴灌尿素 5kg/亩+硫酸钾 3kg/亩，间隔 10 天 1 次，共 2 次，促进幼苗健壮生长。
封冻前滴灌防冻水 20m³/亩，覆盖秸秆保温，防止冻害，确保安全越冬。
蒜头膨大初期滴灌高钾肥（12-8-30）10kg/亩，间隔 7 天，共 2-3 次，提升产量。
生长中期保持土壤湿度 70-80%，后期降至 60%以防烂根，采收前 7 天停止灌溉。
播后苗前喷施 33%二甲戊灵乳油 150mL/亩，覆膜田可减少用药量 50%。叶枯病用 50%腐霉利 1000 倍液滴灌，根腐病用恶霉灵 3000 倍液灌根，轮换用药。
蒜蛆用 5%高效氯氟氰菊酯 1000 倍液滴灌，蓟马喷施吡虫啉 2000 倍液，早防早治。越冬期地膜+秸秆双层覆盖，春季气温超过 25℃时揭膜，防止高温灼伤叶片。
现蕾期滴灌螯合钙 1kg/亩+硼砂 0.5kg/亩，预防干尖和畸形蒜。发现早抽薹及时摘薹，并滴灌磷酸二氢钾 2kg/亩，减少养分消耗。
每月用 0.2%次氯酸钠冲洗滴灌系统，防止藻类及杂质堵塞滴头，定期用土壤墒情仪检测 0-40cm 土层含水量，确保精准灌溉。
抽薹期喷施矮壮素 500ppm，降低株高，增强抗风能力，与禾本科作物轮作 2 年以上，减少土传病害积累。黑色地膜覆盖可提高地温 2-3℃，抑草效果达 90%，注意膜孔对位。
干旱地区采用保水剂（5kg/亩）与基肥混施，提高土壤持水力。多雨地区起垄栽培，垄高 15-20cm，配套排水沟，防止积水烂根。
定期检测灌溉水 pH 值，超过 7.5 时加磷酸调节至 6.5，提高肥效。连作田播前施生物菌肥 200kg/亩，抑制病原菌繁殖。采用大蒜播种机，效率提高 10 倍，播种深度误差±0.5cm。

田间操作避免机械损伤滴灌带，破损处及时用专用接头修复。设置反光带或驱鸟器，减少鸟类啄食幼苗。冻害后立即滴灌氨基酸肥 2kg/亩+芸苔素内酯，促进恢复。
根据叶片营养诊断结果调整氮磷钾比例，实现动态调控后期喷施尿素 1%+磷酸二氢钾 0.3%溶液，延长功能叶寿命。按蒜头横径分级：>5.5cm 为特级，4.5-5.5cm 为一级，提高商品率。
肥料溶解度应>500g/L，避免沉淀堵塞系统，优先选用水溶肥。黄叶时滴灌硫酸亚铁 2kg/亩，紫叶时补施磷酸二氢钾 3kg/亩，沙土地播种深度增加 1cm，黏土地播后覆盖地膜防雨冲刷。
安装土壤温湿度传感器，实现自动化灌溉，节水 15%以上，采收前 10 天停止氮肥，促进蒜皮老化，提高耐贮性，选择阴天或傍晚施肥，避免高温蒸发损失肥效。
抽薹前 7 天滴灌高钾肥（12-8-30）5kg/亩+尿素 3kg/亩，保持土壤湿度 70%，促进蒜薹粗壮生长。
当蒜薹弯曲呈“秤钩”状，顶端花苞膨大但未开裂时采收，晴天上午 10 点前进行，避免薹条易断。
左手捏住假茎基部，右手用采薹针在离地 15cm 处划开叶鞘，顺势上提，不断裂、不伤叶。采收时保持力度均匀，避免猛拽导致薹管撕裂，影响蒜头膨大，破损处喷杀菌剂防病。
分 2-3 次采收，先收健壮薹，弱薹延后 2-3 天，提高总产量和商品率。采收后立即滴灌平衡肥（20-20-20）5kg/亩，促进蒜头膨大，同时叶面喷施磷酸二氢钾防早衰。
采薹后喷施代森锰锌 800 倍液+芸苔素内酯，预防病菌从伤口侵入，加速植株恢复，若薹长超过 40cm，需提前采收，避免养分过度消耗，影响蒜头产量。
抽薹期遇风雨天气，可喷施矮壮素 500ppm 降低株高，增强抗倒伏能力，漏采的残薹需人工补摘，防止继续消耗养分，摘除后及时补充水肥。
采收后按薹长（≥30cm）、粗度（≥0.6cm）分级捆扎，剔除病薹、畸形薹，提高售价。短期储存需直立放置于阴凉处，基部浸水 1-2cm；长期储存宜冷藏（0-2℃），湿度 85%-90%。
选种处理选择饱满无病蒜种，播前用多菌灵浸种 2 小时，晾干后掰瓣，去除底盘促进发芽。深耕 25-30cm，亩施腐熟有机肥 3000kg+复合肥 40kg 作基肥，确保土壤疏松肥沃。
播种时间北方 9-10 月，南方 10-11 月，地温 18-20℃时播种，覆土 2-3cm，镇压保墒。行距 20cm，株距 10-12cm，亩植 2.5-3 万株，大瓣稀植，小瓣密植，保证通风透光。
铺设滴灌带 2-3 条/亩，滴头间距 20-30cm，流量 1.5-2L/h，确保水肥均匀。苗期管理 3 叶期追尿素 5kg/亩，保持土壤湿润，促进幼苗健壮生长。封冻前浇透水，覆盖秸秆或地膜保温，防止冻害死苗。
气温回升至 5℃时滴灌高氮肥 8kg/亩，促早发快长，蒜头膨大初期滴灌高钾肥 10kg/亩，间隔 7 天，共 2-3 次，提高产量，生长中期保持土壤湿度 70-80%，后期降至 60%，采收前 7 天停水防裂头。
叶枯病：发病初期喷施腐霉利 1000 倍液，7 天 1 次，连喷 2-3 次。用恶霉灵 3000 倍液灌根，病株及时拔除，避免蔓延，播前沟施辛硫磷颗粒剂 3kg/亩，生长期用高效氯氟氰菊酯滴灌。
悬挂蓝色粘板 20-30 块/亩，或喷施吡虫啉 2000 倍液，播后苗前喷二甲戊灵 150mL/亩，覆膜田减半，抑草 90%以上，抽薹前 7 天滴灌高钾肥 5kg/亩，保持土壤湿润，促薹粗壮。
蒜薹弯曲呈“秤钩”状，花苞未开裂时采收，避免过老影响品质。左手扶假茎，右手用采薹针划开叶鞘，顺势上提，不断裂、不伤叶。先收健壮薹，弱薹延后 2-3 天，提高总产量和商品率。
摘薹后立即滴灌平衡肥 5kg/亩，促进蒜头膨大，叶片 2/3 枯黄、假茎变软时采收，过早减产，过迟易散瓣，晴天上午采收，深挖轻放，避免机械损伤，晾晒 2-3 天。
按蒜头横径分级，>5.5cm 为特级，4.5-5.5cm 为一级，提高商品率，采收后摊放通风处晾晒 3-5 天，待外皮干燥后剪茎去根，温度 0-2℃，湿度 65%-70%，通风良好，避免发芽和霉变。

与禾本科作物轮作2年以上，减少土传病害积累，后期喷施0.3%磷酸二氢钾+1%尿素，延长叶片功能期。膨大期保持水分均衡，避免忽干忽湿导致蒜头开裂。
每月用0.2%次氯酸钠冲洗系统，防止堵塞滴头。详细记录播种、水肥、病虫害等数据，优化来年管理，选择地势平坦、排灌方便的地块，土壤以疏松肥沃的砂壤土为宜，pH值保持在6.0-7.5之间。
播种前深耕25-30厘米，结合整地每亩施入腐熟有机肥3000-4000公斤，提高土壤保水保肥能力，选用脱毒蒜种，播种前用50℃温水浸泡15分钟或用50%多菌灵500倍液浸种2小时，预防病害。
北方地区9月下旬至1月上旬播种，南方地区10月下旬至11月上旬播种，确保冬前形成5-6片叶。采用宽窄行种植，宽行25厘米，窄行15厘米，株距10厘米，每亩保苗3万株左右。
安装滴灌系统，主管道采用PE管，支管间距60-80厘米，滴头间距30厘米，流量1.5-2L/h。播种后立即滴灌出苗水，灌水量15-20立方米/亩，确保土壤含水量达到田间持水量的70%。越冬前浇足封冻水，灌水量25-30立方米/亩，结合覆盖地膜或秸秆保温防冻。返青期追施高氮水溶肥（30-10-10）5-8公斤/亩，促进植株快速恢复生长。
蒜薹伸长期增施高钾水溶肥（15-5-30）8-10公斤/亩，间隔7-10天一次，共2-3次。鳞茎膨大期控制灌水量，保持土壤含水量60-65%，防止水分过多导致蒜头开裂。
采收前7-10天停止灌水，降低土壤湿度，提高蒜头耐贮性。定期检测灌溉水pH值和EC值，pH值高于7.5时用磷酸调节，EC值控制在1.5mS/cm以内。
肥料选择全水溶性复合肥，溶解度高，无沉淀，避免堵塞滴灌系统。每次施肥量控制在5公斤/亩以内，采用"少量多次"原则，提高肥料利用率。施肥后继续滴灌15-20分钟，冲洗管道内残留肥料，防止滴头堵塞。
每月用0.2%次氯酸钠溶液冲洗滴灌系统，清除藻类和杂质，保持系统畅通。定期检查滴头出水情况，发现堵塞及时更换或清洗，确保灌溉均匀。冬季来临前排空管道存水，防止冻裂，延长系统使用寿命。
采用土壤墒情监测仪，实时监测0-40cm土层含水量，实现精准灌溉，结合气象数据调整灌溉计划，雨天减少灌水量，晴天适当增加，苗期保持土壤含水量65-70%，促进根系发育，培育壮苗。
抽薹期保持土壤含水量70-75%，确保蒜薹生长健壮，提高产量。鳞茎膨大期控制灌水量，保持土壤含水量60-65%，防止裂头。采用交替灌溉方式，每次灌溉湿润不同区域的根系，促进根系发育。
配合滴灌施用生物菌肥，改善土壤微生态，提高肥料利用率，出现缺素症状时，通过滴灌系统补充相应中微量元素，快速矫正。高温季节选择早晚灌溉，减少水分蒸发损失，提高灌溉效率。
沙质土壤增加灌溉频次，减少单次灌水量；粘重土壤反之，盐碱地加大灌水量，定期淋洗盐分，控制土壤EC值在安全范围，连作地块滴灌时添加枯草芽孢杆菌等生物制剂，减轻连作障碍。抽薹前喷施0.3%硼砂溶液，提高蒜薹品质，防止花而不实。
滴灌系统压力控制在0.05-0.1MPa，确保各滴头出水均匀。安装过滤器，定期清洗，防止杂质进入滴灌带，造成堵塞，采用压力补偿式滴灌带，适应地形起伏，保证灌溉均匀度。结合水肥一体化系统，实现自动化控制，提高管理效率。
建立灌溉档案，记录每次灌水量、施肥量及作物长势，优化管理。苗期喷施0.2%磷酸二氢钾+0.1%尿素溶液，促进幼苗健壮生长。返青期叶面喷施微量元素肥，补充根系吸收不足的营养元素。
大蒜水肥一体化的意义：推广水肥一体化，按照大蒜生长发育的需求，供水供肥。不但节约

水肥资源,提高利用率,同时也提高大蒜的产量和品质，生长健壮，减少病虫害危害，一举多得。
大蒜水肥一体化的原则：最好是膜下滴管带，可以随意调节水肥供应量，按需分配；在施足底肥的基础上，根据大蒜生长发育进行补充施肥；从施用无公害的有机肥为主，提高肥力，辅助速效性化肥；以氮磷钾三要素为主，适当搭配各种微量元素，全元施肥；逐步提高土壤肥沃度，尽量限制速效性化肥的施用量。
大蒜的需肥规律：前期和中期以氮素化肥为主，开花坐果(鳞芽、鳞茎分化)开始承步转向以磷肥为主，蒜头膨大期逐步转向以钾元素为主的生长发育。
大蒜不同部位需肥种类：根茎叶的生长是以氮肥为主的生长，磷钾肥辅助作用。鳞芽、鳞茎的生长是以磷肥为主的生长，氮钾肥辅助生长。蒜头膨大期是以钾为主的生长，氮磷辅助生长。
”水调肥，气生根。”的含义:各种肥料、营养物质必须溶解到水里，作物的根吸收水分时带入体内，这叫以水调肥，水多气少，根系不好，甚至沤根,含水量适中，土壤中有一定的空气，根系才能发达，健壮成长，这叫气生根。
大蒜的苗期，返春初期需要的是温度，要适当控制含水。
大蒜的成熟期，要适当晾地，一般不能浇水。
大蒜的五个需水高峰期：①发芽水（也叫大蒜蒙头水），大蒜播种后一段都要浇一次水。②越冬期，因为温度低，大蒜不能生长。应该浇一次水保护安全越冬；③返青水，大蒜返青后，气温升高消耗量大必须浇一次返青水；④大蒜开始抽苔，水肥需要量大，应该水肥齐攻，保证生长；⑤大蒜抽苔后，蒜头急剧膨大，生长迅速，必须浇一通透水。
大蒜的追肥，也就是水肥一体化：大蒜需水最多的时期，往往需肥量也大，特别是中后期，一般浇水要配比一定含量的肥料，随水带肥；大蒜水肥一体化，必须施用水溶性肥料，全水溶无残渣，利用率显著提高，速溶性肥料最好，效果快；水肥一体化必须按大蒜生育期的需求配比，前中期以氮肥为主，中偏后期以磷肥为主，后期膨大以钾肥为主；大蒜水肥一体化，要计算好水肥比例，浓度过大作物不能吸收。浓度过小达不到应有的效果，适量配比；水肥一体化要浇水均匀一致，存水多的地方，肥料也多，容易伤苗，避免水量过大；水肥一体化，可以使大蒜生长健壮，发育良好，有效提高产量，抵抗各种疾病，减少发病率。
培肥地力是高产的其础，土壤肥沃才能使大蒜高产优质。土壤的肥沃度是指有机质的含量多少，有机质的含量越高肥沃度越高，有机质含量达到 2-4.5%时可从少用或不用化肥。
大蒜适宜的酸碱度 PH 值，应该在 6-7.5 最好。过酸或过碱，PH 低于 5 高于 8.5 不宜种植大蒜。
大蒜的需肥，一般每生产 1000 公斤大蒜头(1 吨)，需要吸收纯氮 28-29 斤，五氧化磷 9-11 斤，氧化甲 26-27 斤，大蒜种植应该以施足底肥为主，大蒜用肥是粮食作物的三倍左右，每亩施氮磷钾的总含量 120-140 斤为宜。
三肥：一肥是底肥，施足底肥，利用率高，肥效长，从种到收都可以利用，就是高产的基础。二肥是大蒜返青盛期，植株需要大量生长，内部开始分化鳞茎鳞芽，急需要大量的水肥，单靠土壤分解的肥料不能满足作物生长的需要，需要人工补充，水肥齐攻，大约在四月上旬，清明节前后，水溶肥从高氮为主，5-8 公斤。三肥是抽苔期，防止后期脱肥，也就是在上次浇水施肥后 8-12 天，再施一次肥，每亩 5 公斤左右水溶肥。
五水：五水中的第一水是大蒜蒙头水，在大蒜种植完后立即浇水，确保大蒜苗齐苗壮。第二水是越冬水，进入 12 月份气温降至 10℃以下停止生长，需要足墒越冬，浇一次水。常言说，大蒜不怕湿冻怕干冻，充足的水份可以保护大蒜安全越冬。第三水是返青盛期的返青水，这一水不能早浇，必须在地温稳定在 15℃左右，结合施肥浇一次水。第四水是抽苔水，也叫摇尾水。大蒜蒜苔开始伸长上次浇水后 8-12 天，促施蒜苔快速生长，结合追肥。第五水是

蒜头彭大水，抽完蒜苔以从后，大蒜失去生长点，光合作用产生的有机物质大量向蒜头集中，促使蒜头快速彭大，必须有水配合，但是不用追肥，此时根系已老化失去生长功能。所谓后期管理水当先，有水就是管好了。一般抽苔后十八天就成熟了，可以收获,进入收获期。
大蒜喜凉爽，怕炎热，可以耐-10℃的低温不死，冬季温度-15℃以下全部冻死，不能越冬。
大蒜的正常生长温度是 12 一 25℃，低于 12℃能发芽但是不能长叶，25℃以上不能吸肥吸水而死亡。
大蒜是需要春化的作物,需要在 5-6 片叶以前终过 0 一 12℃的低温两个月左右，渡过春化，如果春化不好或不能顺利的通过春化，指种过早或过晚，容易出现独头或形成面包蒜和毛蒜，失去商品价值。
春化阶段：大蒜发芽以后在五、六片以前，经过 0 至 12℃的低温 2 个月以上，分化成新的蒜头，形成蒜瓣，这就叫春化(同小麦一样)。没有通过春化的大蒜形成独头，春化不好的大蒜形成气包蒜或马尾蒜。主要是特定的温度、时间、蒜苗的生长期所决定的。
光照阶段:大蒜完成春化以后，随着气温的升高进入光照阶段，这个时期大量生长根、茎、叶，积累营养，准备结实。这个时期，需要充足的适宜的光照，大蒜返青以后，叶片达到 18-20 片，单株根系达到 180 一 200 条，茎粗大到直径 1.5-1.8 厘米，叶片厚度可达 2 一 3 毫米，长度达到 40-60 厘米，叶面积指数达到 4 一 5 个，叶色浓绿，也是大蒜需肥零水最多的时期。3 月中下旬-4 月上中旬。
结实阶段:也叫生殖生长阶段，这个时期营养生长完成，根、茎、叶不在增加，体内积累了大量的碳水化合物，抽完蒜苔以后，全部转移到蒜头，迅速膨大，形以蒜头等茎叶全部枯黄、萎缩发黄转移完成收获。
培肥地力是基础，土壤肥沃才能使作物高产忧质。土壤的肥沃度是指有机质的含量越高越肥沃。有机质含有各种农作物生长发育的所需营养，土壤中的水、气、温适宜。土壤肥沃度有四个标准①有机质含量高 15‰以上；②PH 值中性 6.5-7.5；③土壤疏松海绵田；④病虫害少，大量增施有机肥，改良土壤结构，努力提高肥沃度。
适期播种很重要，过早或过晚播种，春化不好容易奇型（独头、面包蒜、毛蒜、开裂等），一般最高温 18-22℃最适，大体时间每年 10 月 5 日一 15 日，寒露节前后。
大蒜的生长主要是：两大阶段，前期营养生长，后期生殖生长。
①营养生长是指根、茎、叶的生长，这个时期以氮肥为主，磷钾肥为辅，主要指抽苔前。返青从后需要大量的肥水，特别是温度在 15℃以上清明前后，必须保证水,肥充足。增加氮肥可显著旺盛，提高产量。
②生殖生长:为繁衍后代的生长、开花、结果。大蒜从开始抽苔、蒜头膨大为生殖生长,根、茎、叶生长停止。这个时期以水和磷钾肥为主，很少用氮、钾肥对蒜头膨大起着很重要的作用。氮肥主要促进叶绿体合成。
大蒜的蒜母（蒜瓣）可以供大蒜生长五片叶，不需要从土壤中吸收营养，有水和温度即可，年前生长 6 叶一心越冬，吸肥量很少。
肥料是作物的粮食，庄稼吃足喝饱了才能获得高产。庄稼一枝花，全靠肥当家。合理施肥是高产丰收重要一环。
大蒜喜凉爽怕炎热，10-12℃可以发芽，15-22℃生长发育最快最好。5℃左右停止发育，-8—-10℃可以忍耐，超过-10℃开始死亡。高温 25℃以上根系不能吸水肥，自然死亡。
春播地区的大蒜,从播种到收获蒜头,在 1 年以内完成,需 100~120 天;秋播地区的大蒜,播种后要经过一段较长的越冬期,到翌年才收获蒜头,生育期长达 210~270 天。
花茎伸长期,叶片全部展开,叶面积迅速增长,花茎生长加快,鳞芽逐渐增大,是大蒜植株生长量最大的时期,也是生产上水、肥管理的关键时期。
用大的种瓣播种,植株长势较强,叶数较多,单株叶面积较大,单位面积株数比较小的种瓣可适

当减少。
大蒜的根系浅,根毛少,吸水范围较小,所以不耐旱,但不同生育期对土壤湿度的要求有差异。
大蒜对富含腐殖质的有机肥反应良好,增产效果显著。全生育期吸收的氮最多,钾次之,磷最少。。缺氮对产量的影响最大,缺磷影响次之,缺钾的影响相对较小。
大蒜出苗期和幼苗期的生长主要靠种瓣中贮藏的养分,从土壤中吸收的氮、磷、钾量很少,所以在使用基肥的基础上一般不需要再施种肥。特别是用碳酸氢铵、硝酸铵、尿素作种肥时,对根系有腐蚀作用。
大蒜进入花茎伸长期后,叶片和蒜薹的生长加快,吸收的氮、磷、钾迅速增加,至鳞茎肥大中期达最高峰,所以花茎伸长期是追肥的关键时期。追肥的种类以氮肥为主。鳞茎膨大后期,叶片逐渐枯黄,根系老化,吸收力减弱,吸肥量减少,一般不再追肥,特别要控制氮肥的施用,否则易导致鳞茎开裂和散瓣。
硫是大蒜风味品质成分的构成元素,增施硫肥不仅可以增进风味品质,还可以提高蒜薹和蒜头产量。
大蒜幼苗长势的强弱关系到花芽和鳞芽能否正常分化,所以幼苗期长短不同的品种,在追肥和灌水时期的掌握上也应有所区别。幼苗期短的极早熟品种和早熟品种在苗出齐后便可开始追肥和灌水,中、晚熟品种可在 3 叶期和越冬前各施 1 次追肥。
秋播大蒜于翌年春暖返青后,结合灌水施返青肥,每 667 平方米施尿素 10 千克或碳酸氢铵 20 千克或氮磷钾复合肥 30 千克,为幼苗返青后的旺盛生长提供充足的水分和营养。秋播大蒜种的极早熟和早熟品种以及南方冬季比较温暖的地区,可提早追肥和灌水。
花茎伸长旺盛时期,也是植株营养生长的旺盛时期,对氮、磷、钾的吸收量继续迅速上升。采收蒜薹时,平均日吸收量达最高峰。所以,花茎伸长期田间管理的重点是抓紧追肥和灌水,以满足花茎生长的需要,并为蒜头的肥大打下基础,防止蒜头肥大期缺肥及植株早衰。具体措施是,当蒜薹“露尾”(总苞尖端伸出叶鞘)时,施“催薹肥”,结合灌水每 667 平方米施尿素 20~25 千克。鳞茎肥大期, 结合灌催头水,可根据土壤肥力和前期施肥情况,在肥力不足时可追施催头肥,每 667 平方米施速效化肥尿素 10~15 千克或碳铵 20~40 千克、钾肥 5 千克。同时,可叶面喷施 2%磷酸二氢钾
陕西大蒜产区农民采用“潮蒜”(或称“窨蒜”)措施,具体做法是:播种前 20 天左右,选大小整齐、无病虫的蒜瓣,放在清水中淘洗后捞出,喷上杀菌剂,运到可保持冷凉湿润的场所,使种瓣发根(俗称“催芽”)。潮蒜的场所可因地制宜,因陋就简。
与玉米间作的晚蒜苗,当玉米棒收获后,将玉米秆在蒜苗行间顺着堆放,也可以起防寒保苗及促进幼苗生长的作用。翌年早春地开始解冻后锄地松土,结合浇返青水,追施返青肥,每 667 平方米施尿素 15 千克或碳酸氢铵 37 千克,加速蒜苗生长。
床栽蒜苗的生长主要靠蒜瓣中贮藏的营养,一般不需要施肥,只要供给适宜的温度和水分,就可以生产出鲜嫩的蒜苗。地栽蒜苗,根据生长期的长短,一般需追肥 1~2 次。保护地蒜苗管理的重点是温度和水分。
独头蒜的适宜播期必须在当地做分期播种试验才能确定。播早了,蒜苗的营养生长期长,积累的养分较多,易产生有 2~3 个蒜瓣的小蒜头;播晚了,独头蒜太小。一般秋播地区较蒜头栽培推迟 50 天左右播种为宜。每 667 平方米用种量一般为 100 千克左右。
蒜头贮藏方式(1)常温贮藏是在自然温度状态下的贮藏方式,其优点是简便易行,成本低,是我国蒜农常用的贮藏方式。但贮藏期较短,而且易发生虫蛀和霉变。(2)冷藏是指利用人工降低贮藏环境的温度或利用自然低温条件,抑制蒜头的呼吸作用,减少养分和水分损失,防止病虫繁殖蔓延,以延长贮藏保鲜期。
大蒜在施用有机肥作基肥的基础上,氮肥的使用量和使用次数对二次生长也有影响。氮肥施用量大,二次生长株率增高。同样数量的氮肥,施用次数不同时,二次生长的发生情况也不同。

大蒜喜好富含有机质、疏松肥沃、通气良好、保水、排水和保肥性能良好的微酸性沙壤土或壤土，土壤 pH 为 5.5～6.0 最适合大蒜种植。
大蒜需肥多而且耐肥，增施有机肥有显著的增产效果。大蒜施肥以氮肥为主，增施磷、钾肥可显著增产。
大蒜对硫、铜、硼、锌等微量元素敏感，增施这些微量元素有增产和改善品质的作用。
大蒜苗期需肥较少，所需的营养多由母瓣供应。在叶片旺盛生长期和鳞茎迅速膨大期，需要的营养较多。
大蒜的根系弱，吸收力差，而需肥又多，根据这一特点，施肥时应本着多次、少量的原则，施肥后注意立即浇水，以利吸收。
大蒜在整个生长发育过程中，对氮、磷、钾的总需要量是有一定比例的，大蒜需氮最多，需钾次之，需磷较少。一般在蒜头每亩（1 亩=667m2）产量 1000kg 以上的肥力水平地块，每形成 100kg 蒜头产量，约需氮肥 1.42kg，磷肥 0.44kg，钾肥 0.99kg。但是大蒜的产量不同，对养分的吸收量也有一定的差异。
在亩产量为 1570kg 时，对肥料氮、磷、钾的吸收比例为 1：0.36：0.72。
生产上按比例适量增施肥料，可明显增加大蒜对养分的吸收，充分发挥肥效，降低成本，提高产量和效益。大蒜生长中、后期对氮素的需要量较大，分化大蒜生长中、后期对氮素的需要量较大，分化至抽薹期占 30%左右，鳞茎膨大期最多，吸收量占总吸收量的 40%，所以生产上应注意后期追施氮肥。
大蒜鳞芽分化、抽薹期是对磷素营养元素吸收的强度营养期，吸收量较大，生产上在此期应注意追施速效磷肥。返青期和鳞茎膨大期是两个钾素的吸收高峰期，生产上应注意追施钾肥。
自然空气中，氧气含量约 21%，可满足大蒜生长的需要。二氧化碳含量为 0.03%左右，而光合作用其含量可高达 0.12%，故大蒜设施栽培中可增施二氧化碳气肥。
露地栽培，只在大蒜密度过大和植株封垄后株行间空气流通缓慢时，才会出现二氧化碳亏缺。
土壤中的多种有益微生物是好气性的，微生物活动旺盛，有机物质被微生物迅速而彻底地分解，土壤空气中氧气充足时形成大量速效氮养分。缺氧时，有机质分解缓慢且不彻底，常积累有害的物质，对大蒜生长不利。
减少土壤积水、中耕松土可以改善土壤通气条件，对大蒜根系生长有利
通过合理密植，及时除草、高矮秆作物合理间套作等措施来改善通风条件，有利于提高作物的产量和品质
大蒜的栽培季节既取决于当地的气候和环境条件，也与品种的特性有关。另外，不同大蒜的生育期对温度有不同的要求，因此栽培季节的确定，要根据大蒜不同的品种，在不同的生育阶段对生长环境的要求以及各地区的气候条件来确定。
过量施用化肥，造成土壤板结，导致土壤中拮抗菌减少，给病原菌滋生提供了空间。长期施用化学农药使有益菌减少、病原菌产生抗药性，导致病原菌猖獗，数量猛增。
大蒜体内普遍存在病毒，并且随着种植年限的增加，病毒不断累加，在生长过程中大蒜还会分泌一些对自身产生毒害作用的化感自毒物质，并通过地上淋溶、根系分泌物和残体分解物遗留在土壤中。随着大蒜连作年限的增加，这些自毒物质在土壤中的积累越来越多，从而抑制大蒜自身的生长。表现为生长势衰弱、抗逆性降低。
大蒜对土壤中矿质营养元素的需求种类及吸收比例有一定的规律，即大蒜对土壤养分的吸收是有选择的。大蒜长期连作必然造成土壤中某一种或几种营养元素的亏缺。在得不到及时补充的情况下引发缺素症。
过量施用大量元素化肥，不注重中、微量元素 54 肥料的施用，必然导致大蒜被动吸收，使大蒜体内各种养分比例失调，抗逆能力下降，抗病能力减弱，甚至造成生理病害。
浸种属于可选用技术，其方法有温水浸种、液肥浸种和药剂浸种三类。

温水浸种是用 40℃左右温水浸泡 24h，期间换水 2～3 次，保持水温，捞出后晾 4h 左右即可播种。
液肥浸种可增强种瓣活力，促进早萌发，植株生长健壮，具有显著的早熟增产作用。可在播前用 0.3%的磷酸二氢钾溶液浸种 6h，56 随浸随种。
药剂浸种可在种瓣传播和土传性病害严重的地区选用。药剂浸种可以结合液肥浸种一起完成，浸种液为 1000 份水+3 份 77%硫酸铜钙（多宁）或多菌灵或代森锰锌或甲基托布津等杀菌剂+3 份磷酸二氢钾配制搅匀，将剥好的蒜种装入网袋中，浸入浸种液，将放入的种子袋上部压实，确保种子浸入药液中。
一般浸种 4～6h，不要超过 12h，捞出沥干。不能马上播种时或未播完时，应摊开晾放。此种浸种方法不仅出苗率高，生长健壮，生产量明显增大，而且能有效地抑制母瓣表皮内外多种病菌的滋生和蔓延，减少烂瓣，促进根系发育，从而有利于增产。
浸过的蒜种只宜湿播，不宜干播。所以干旱且灌溉条件差的地区，播种前不宜浸种。
若在干旱土壤中播种浸过种的蒜瓣，种瓣内外水势逆差，蒜瓣内水分将被土壤吸收而致失水，反而阻碍发芽生根，达不到催芽早发的目的
大蒜虽然对土壤的适应性较强，除了盐碱沙荒地外，均能生长。但是由于大蒜根系浅，吸收能力弱，对土壤有一定的要求才能达到优质高产的目的。
适宜种植大蒜的土壤应该符合无公害产地环境条件的要求，尽管大蒜的适应性较强，但还是以土壤疏松、排水良好、有机质丰富的沙壤土为好
沙壤土疏松，适宜根系发育，抽薹早，蒜头大且辛辣味浓，起蒜容易。沙土保肥保水能力较弱，生产的蒜头小，但辣味强。黏重土壤生长出的蒜头小且呈尖形。
大蒜对土壤水分要求较严，既怕干旱，又怕水涝，因此，选择的地块应该具有良好的灌溉和排水条件。
秋播的大蒜在前茬作物收获以后，如果距离播种时间较长，土壤处于板结的状态，要耕翻晒垡，翻耕深度一般在 10～15cm。翻耕大蒜高效栽培管理技术第四章 57 后晒垡，时间一般在 15 天以上。
大蒜的基肥施用量应根据大蒜的目标产量和形成单位产量的吸肥量等多种因素综合考虑。配合有机肥作基肥施用的化肥通常有过磷酸钙、氮磷复合肥、氮钾复合肥或三元复合肥等。
在产量水平和施肥的基础上，一般要求每亩大蒜施标准氮肥 75kg 左右，氮肥的施用，要求 2/3 作基肥、1/3 作追肥，磷、钾肥绝大部分作基肥施用。
一般每亩大蒜施过磷酸钙 30kg 左右，缺磷的新蒜种植区可施到 45kg，老蒜种植区土壤速效磷含量比较高，有机肥料的施用量又多时，可施用过磷酸钙 15kg 左右。
大蒜因生长期长，群体密度高，需肥量大，一般亩施充分腐熟的优质有机肥如粪肥、厩肥等 5000～8000kg 或精制有机肥 400～500kg。并配合施用 50kg 氮、磷、钾三元复合肥
使用生肥，发酵时会烧伤大蒜根系，还会引起地下虫害，尤其是地蛆的严重发生。
基肥的施用方法：一是撒施，在土地翻耕前将肥料均匀撒于地表，然后翻入土中。撒施是施用基肥的一种常见方法，凡是植物密度较大（如水稻等），植物根系遍布于整个耕层，且施肥量又相对较多的地块，都可采用这种方法施肥。
撒施的肥料必须均匀，防止肥料集结，以免植物生长不平衡。
地块经过耕翻、耙、耢之后，还要整地作畦。其目的主要是便于灌溉、排水、密植及管理。此外对土壤温度、湿度和空气条件也有一定的调节作用。
大蒜在播种 58 前需要精细整地作畦，地膜覆盖栽培的，为充分利用土地和便于田间管理，一般畦宽 1.5～2m，畦长以能均匀灌水为宜。畦面要求平整，土壤上松下实，无明暗土块，无杂草，东西向为好。因为冬季日光入射角小，东西向畦能接受较多的阳光，有利于大蒜的越冬生长。

基肥施用的原则：一般以有机肥为主，无机肥为辅；长效肥为主，速效肥为辅；氮、磷、钾（或多元素）肥配合施为主，根据土壤的缺素情况，个别补充为辅。
基肥的施用量：基肥的施用量应根据植物的需肥特点与土壤的供肥特征而定，一般基肥使用量应占该植物总施肥量的 50%左右为宜。质地偏黏的土壤应适当多施，相反，质地偏沙的土壤适当少施。
秋播的大蒜，以畦作为主，便于灌冻水和越冬管理。畦宽 1.5～2m，东西向延长。春播的大蒜，既可垄作，也可畦作，畦作一般可做 1.5～2m 宽的畦，畦作可适当提高种植密度，提高单位面积产量，但地温低，幼苗出土慢，鳞茎发育膨大时受到的土壤压力大。
大蒜播种方法因做畦方式不同而不同，有平畦播种、高垄播种、地膜覆盖畦播种等。
秋播大蒜于第二年返青后，结合灌水施返青肥，每亩施尿素 20kg 或氮、磷、钾复合肥 15kg，为幼苗返青后的旺盛生长提供充足的水分和营养，促进鳞芽和花芽分化。
秋播大蒜中的极早熟和早熟品种及南方冬季比较温暖的地区，可提早追肥和灌水。
春播大蒜的鳞芽和花芽分化发育期较短，烂母期也较短。追肥、灌水等田间管理要相应提前，如果延误时机或水、肥供应不足，则会影响鳞芽和花芽的分化发育，不但抽薹率降低，还会增加独头蒜的比例
当蒜薹“露尾”（总苞尖端伸出叶鞘）时，施“催薹肥”，结合灌水每亩施氮、磷、钾复合肥 20～30kg。以后的时期土壤要经常保持湿润状态。采收蒜薹前 3～4 天停止灌水，以免蒜薹太脆采收时折断。
春播大蒜花芽分化结束后，温度呈持续上升趋势，所以花茎伸长较快。另外，由于鳞芽的分化结束期与花芽的分化结束期相近，在花茎伸长加快时，鳞芽的增长也加快，当蒜薹露尾时蒜头已开始膨大，需水需肥量大，更要抓紧追肥和灌水。
采收蒜薹后，叶片的生长基本停止，鳞茎膨大进入旺盛时期，2 周后开始枯黄脱落，根、茎、叶的生长逐渐衰退，植株生长减慢，日平均吸收的氮、磷、钾量明显减少。
南方在鳞茎膨大期常遇多雨天气，土壤湿度大，容易引起散瓣，影响蒜头质量，应注意开沟排水，降低土壤含水量。
由于环境条件的改善，大蒜地膜覆盖条件下，植株生长健壮，根系发达，叶面积大，抗病性增强。大蒜进行地膜覆盖后，地膜阻挡了种蝇向蒜根周围产卵，减少了根蛆为害。地膜覆盖也抑制了杂草的发生和为害
地膜覆盖一般有三种方法，一是播种后浇水及时盖膜法，即大蒜播种后浇水，待水渗下后，喷洒除草剂后覆盖地膜；二是播后浇水、苗长 3～5 叶后盖膜法，即提前播种、浇水、喷洒除草剂，待温度降到 16℃左右时盖膜；三是先盖膜后播种法，即将地膜盖好后，根据株行距用尖头木棍或铁棍等打孔，深 2～3cm，随后点种覆土，此方法适于播种晚的蒜田，是提高地温的一项措施。
（1）青蒜苗喜肥，尤其是农家肥、有机肥，兼施化肥，肥水充足时，产量更高。2）适当培土可增加青蒜假茎长度，提高产品质量。3）青蒜主要依靠种瓣积累的养分生长，如果积肥充足，生长期可不必追肥。
经催芽发根的种瓣，播种时应避免在烈日下暴晒。天旱地干，可先灌水造墒，合墒整地后再进行播种。
大蒜、花生套种　为了提高种植效益，可采用地膜覆盖大蒜套种花生栽培模式。该栽培模式为大蒜优质高产提供了良好的生长环境，花生的播种期比麦垄套种提前 15～20 天，比麦后直播提前 30～40 天，收获期提前 10～15 天。该模式株行距配置比较合理，通风透光好，个体发育好，分枝多、花量大、结果多、产量高。
早蒜苗套种小麦　选用适于作蒜苗栽培的早熟大蒜品种，经打破休眠催芽处理后于 8 月上旬播种，行距为 15cm，株距为 3～4cm。10 月上旬将蒜苗行间细锄一遍，然后撒播小麦种，

播后锄地将小麦种掩埋压实；12 月前采收蒜苗。蒜苗收获后将小麦镇压一遍，随后灌水，使因采收蒜苗而受损伤的小麦根系与土壤紧密接触，以减少冬季冻死苗现象。

蒜棉间作两熟栽培是一种高效栽培方式，在山东省大蒜主产区金乡县及周边地区普遍采用大蒜、棉花套种模式。这种栽培模式下，大蒜在生长过程中产生的根系分泌物对棉花苗期病虫害有一定的抑制作用，减轻了棉蚜危害。棉花产量、质量与纯春棉基本相当，多收一季大蒜，棉田综合效益高，深受农民欢迎。

大蒜、菠菜套种　以生产蒜头为主的大蒜与菠菜套种技术如下。大蒜一般在 9 月下旬～10 月上旬播种。菠菜的播期与大蒜的播期相同，但菠菜应撒播在畦埂上。大蒜出苗慢，苗期占空间小，菠菜有充分的生长空间。初冬至春天均可以随时进去采挖菠菜，先挖离蒜近的菠菜。小苗可留在地里越冬，第二年 3 月上中旬全部采收完毕。

在大蒜生长发育过程中，正常情况下生长到一定时期，鳞芽开始分化，叶片退化，鳞芽逐渐形成蒜瓣，退化的鳞芽外的鳞片形成了蒜瓣的外皮。但是不利的环境条件下，大蒜在蒜薹露出之前，蒜轴周围长出 5～6 片小蒜叶，围着蒜轴生长。

防止大蒜二次生长的途径 1.选择适宜品种不同品种抗二次生长的能力不同。因此，可以通过多年种植经验了解不同品种对二次生长是属于抗型还是易发型的。尤其是对外层型二次生长的抗性更应了解清楚，因为该类型对蒜头的质量影响最大。如果目前所种的主栽品种属于二次生长易发型，可以通过引种确定后进行换种，这样可以争取时间、加快速度。当然，在确定品种时不仅要选择对二次生长抗性强，而且要考虑适合国内外市场要求的优良品种。

冷库中储藏的保鲜大蒜只能作为食用商品蒜，不能作为以收获蒜头为主的种用蒜。不论哪种熟性的品种，都会随着低温储藏时间的延长，使长势减弱，二次生长加重，尤其是早熟品种更甚之，不但外层型所含比例大，收获的蒜头几乎没有商品价值。

选择大小适中的蒜瓣播种，合理密植大蒜二次生长的发生与种瓣大小密切相关。种瓣过大二次生长率高，种瓣过小产量低，又易产生独头蒜。生产中应选用适中的种瓣为宜。适当密植可减少二次生长发生。

掌握适宜的播种期盲目提早播期是二次生长发生的原因之一。对易发生二次生长的早熟品种，更不能盲目提早，尤其在暖冬年情况下，二次生长更为严重。大蒜的适宜生长温度为 12～26℃，蒜瓣在 12℃以上发芽生长整齐。

合理灌水，控制氮肥施用氮肥用量过大，返青水浇得早，二次生长率均高。要多施有机肥，配施磷钾肥，防止偏施氮肥。返青水要控制适当晚浇。基肥采用以有机肥为主，适当配合氮磷钾三元复合肥。用化肥作追肥时忌多量氮肥单独施用，特别是返青期少施或不施速效性氮肥。尤其在水多氮肥足的情况下，植株生长过旺，二次生长的发生率也会高。

地膜覆盖不要过早地膜覆盖栽培在大蒜集中产区已经成为提高产量和质量的重要手段。但使用不当也是诱发二次生长的一个原因。在同样冬暖倒春寒年份，地膜覆盖的大蒜二次生长明显多于不覆盖的。

为了防止由于覆膜而引起的二次生长，覆膜时应注意以下几个方面：①秋播区覆膜的要比不覆膜的在适宜播期内向后推迟 5～6 天，以防止苗期生长过旺，鳞芽、花芽分化过早，第二年春遇低温产生二次生长；②播期不推迟，播种后先不覆盖薄膜，待蒜苗已经全部出土齐苗后，天气已经开始转凉，于 10 月中、下旬采取一次性集中盖膜掏苗。这种方法省工，也降低了二次生长的发生率。③地膜覆盖的返青后要控制氮素化肥用量。

“面包蒜”的产生原因：1.追施氮肥过多 2.施用未腐熟的有机肥 3.基肥中氮、磷、钾的配比不合理 4.土壤含水量过大 5.气候因素 6.播种时期 7.蒜种不适宜 8.蒜种储藏的条件不当

春季追肥随着追氮肥量的增加、时间的提前，“面包蒜”发生率随之提高。早春一次性追施氮肥，偏施氮肥，比追施复合肥料、分期延迟追施氮肥，“面包蒜”发生量较高。

在大蒜主产区，由于人力、物力的制约，蒜农往往施用未腐熟的有机肥，易导致地蛆的发生，

增加生产成本，致使许多蒜农少施或不施有机肥，这是“面包蒜”产生的重要因素。
基肥中氮、磷、钾配比不合理，氮肥含量较高，或重施、偏施氮肥，磷钾肥相对缺乏，施肥比例失调是导致“面包蒜”发生的主 110 要原因之一。“面包蒜”的发生程度与氮、磷、钾肥的配比关系密切，随着氮肥比例的增加（即磷钾肥比例的下降）而不断增加。大蒜是需钾较多的作物，钾和磷促进植物对氮素营养的吸收和运转，磷、钾肥的不足会加剧氮肥结构性过剩，加强蒜株的顶端优势，抑制养分向鳞茎的转移和运输，减缓鳞茎的膨大速度，鳞芽发育受阻，使鳞茎膨大不充实而产生“面包蒜”。
大蒜的适应性较强，但在生长过程中对环境条件、水分都十分敏感，管理过程中的大肥大水，土壤含水量过大，都会造成面包蒜的产生。田间调查发现，地势低洼的大蒜地块，“面包蒜”发生较普遍。据试验，春季随着浇水量的增加，不仅大蒜产量降低，而且“面包蒜”发生率直线上升。
防止“面包蒜”的途径：1.选择优良品种 2.适期播种 3.合理施肥 4.水分管理得当 5.科学储存蒜种
防止抽薹不良的途径：1.正确选择适宜的品种 2.适期播种
要适时播种，加强水肥管理，防止缺水、少肥、受冻。注意创造适宜大蒜花芽分化和抽薹的环境条件，以满足品种花芽发育的需要。
肥水管理不当，苗期营养不良或过量施用氮肥使植株徒长，都容易引起瘫苗。
防止瘫苗的途径及时进行病虫害防治，加强肥水管理，使大蒜苗生长健壮，以避免植株早衰而引起瘫苗的产生。低温多雨时，应防止田间积水，土壤湿度不宜过大，合理采薹，避免伤害假茎。
氮素供应不足，大蒜的生长受到抑制，叶先从外部失绿发黄，重则枯死。在蒜头膨大前，大蒜进行营养生长，为蒜头的膨大打基础，因此，对氮素的要求较高，也是供氮的关键时期；否则蒜头膨大以后，氮供应不足，会使蒜头膨大受阻。所以应在蒜头膨大前施足氮肥。
幼苗期缺磷，株高降低，叶数增加受抑，根系发育不良。蒜头膨大期缺磷会减产。出现缺磷症状以后，再向土壤中追施磷肥已于事无补。必须在基肥中配加磷肥，也可用磷酸二氢钾液进行叶面喷肥来急救。
苗期缺钾，当时并无明显的症状，但对以后蒜头膨大产生很大的影响。如果在蒜头膨大期间缺钾，易感染心腐病、白腐病。一般来讲，大蒜长到一定高度，要控制氮肥，增施钾肥。
大蒜缺钙，会影响根系和生长点的发育，降低组织内部碳水化合物的含量，使蒜头膨大受阻，产量降低，品质下降，同时也诱发心腐病。
营养生长不良，叶片弯曲，嫩叶黄绿相间，质地也变脆，蒜头疏松。发病后可在叶面喷 0.1%～0.2%的硼酸溶液。在土壤中施 1kg/亩硼砂可缓解。但不要过量，否则烧根。
嫩叶顶部变黄，继而向基部扩展，严重时全株枯死。发现缺镁，即在叶面喷 1%的硫酸镁溶液，每隔 5～7 天连喷 2～3 次可以急救。
氮素吸收过剩，叶色深绿，发育进程迟缓，地上部分“贪青”生长，大蒜成熟晚。氮素供应过多，蒜头内的氮积累过多，易诱发心腐病。
磷吸收过剩时，可表现缺钙、缺钾、缺镁等症状，易诱发心腐病。
大蒜播后、苗前用除草剂过量，特别是乙草胺用量过大，易引起叶片生长受抑制、失绿发黄、个别心叶扭曲、根系减少、蒜头变小等症状。
当土壤的 pH 低于 5 时，就会发生大蒜酸害（彩图 6），导致土壤呈酸性的根本原因是大量使用化学肥料。大蒜受酸害后，植株发育不良，蒜瓣软化腐烂。防治技术：大量使用腐熟的有机肥，减少化学肥料的用量，实行配方施肥技术，氮、磷、钾肥配合施用，避免偏施氮肥。
预防大蒜生理性病害一定要注意采用配方施肥技术，合理安排氮、磷、钾肥的施用量和施用比例；用速效氮肥作追肥时忌多次多量施用，特别是在大蒜返青期，要少施或者不施速效氮

肥。使用除草剂时一定要注意不要过量。

对大蒜虫害防治应以应用黑光灯、色板、性诱剂等诱杀和趋避为主，化学防治为辅。

整地在符合无公害蔬菜生产条件的基地选择肥沃、疏松的壤土地块，选用玉米、西瓜、棉花等前茬，避免与葱、蒜类植物连作。整地时田间土壤宜干不宜湿，采取机耕、机耙、机打畦。畦面宽度一般为 1.8m（与薄膜宽度相配套）。

发芽期管理大蒜播种后 7～10 天即可出土。在蒜芽快要出土时，清晨用新扫把轻拍地膜，使蒜芽顶破地膜；少数未能顶出地膜的，应用小铁钩及时破膜引苗，以使蒜苗顺利顶出地膜。

幼苗期管理秋播大蒜苗期较长，管理的主要工作是使幼苗生长健壮，防止徒长和提早退母，保护幼苗安全越冬。第二年春幼苗返青后，可于清明前后，视植株长势及天气和土壤情况浇水、追肥 1 次，每亩追施尿素 20kg，并用辛硫磷等结合浇水灌根防治蒜蛆。

鳞芽、花芽分化和蒜薹生长期管理幼苗期后，植株迅速生长，花芽和鳞芽开始分化，植株进入旺盛生长期，对水肥的需要明显增多。当蒜薹形成以后，需要追施钾肥。蒜薹采收前 3～4 天停止浇水，以免蒜薹折断。

鳞茎膨大期管理蒜薹采收后，植株中的营养逐渐向鳞茎中转运，鳞茎进入膨大大蒜高效栽培实例第十章 163 期后，需水量增加。在蒜薹全部收完后，需增加灌水次数，保持土壤湿润；大蒜采收前 5～7 天停止浇水，以防土壤湿度过大，引起蒜皮腐烂，鳞茎松散，不耐储存。

病虫害防治大蒜覆膜栽培的，膜下温度高，利于多种病原物和病虫越冬，加重了病虫害的发生。大蒜常发生的虫害有蒜蛆，病害有大蒜叶枯病、紫斑病、灰霉病、锈病和病毒病。蒜蛆防治方法为土壤处理，用 20%辛硫磷乳剂每亩 750～1000mL 冲施防治效果最好。

整地施肥 1.精耕细作前茬作物收获后，要抢茬耕翻，耕后纵横耙细、耙平，使耕层疏松，保好墒情，以利栽种 2.科学施肥大蒜需肥较多，掌握以有机肥料为主、化肥为辅，基肥为主、追肥为辅的施肥原则 3.土壤处理用辛硫磷或阿维菌素防治蒜蛆等地下害虫，同时可施入敌克松、多菌灵、百菌清、地菌净进行土壤消毒，以防治土传病害。

合理播种 1.异地换种 2.精选良种 3.适时播种 4.合理密植 5.科学播种 6.化学除草，科学覆膜。

肥水管理大蒜幼苗生长期虽有种瓣营养，但为促进幼苗生长，增大植株的营养面积，仍应适期追肥。由于大蒜根系吸收水肥的能力弱，耐肥，故可以经常追肥，以满足其生长发育的需要。大蒜肥水管理一般要进行 3～4 次。

催苗肥大蒜出齐苗后，追一次催苗肥，以提高大蒜的安全越冬性能。对幼苗前期生长较快的品种，可适当晚施。如果土壤肥沃，基肥充足，尤其是易发生外层型二次生长的品种，催苗肥应少施或不施。

盛长肥播种 60～80 天后，重施一次，每亩硫酸铵 10kg，硫酸钾 10kg。做到早熟品种早追，中晚熟品种晚追，以促进幼苗长势旺，茎叶粗壮，到烂母时少黄尖或不黄尖

孕薹肥种蒜烂母后，花芽和鳞芽陆续分化进入花茎伸长期。此期旧根衰老，新根大量发生，同时茎叶和蒜薹也迅速伸长，蒜头也开始缓慢膨大，因而需要养分多，应重施速效钾、氮肥 10～15kg。于现尾前半月左右施入，以满足需要，促使蒜薹抽生快、旺盛生长。

大蒜生长对氮、磷、钾等养分需求较大。在幼苗期，氮素能促进叶片生长，使蒜苗健壮。一般每亩可施入高氮复合肥 15-20 公斤，施肥时要注意均匀，避免烧苗，可结合浇水进行，让肥料更好地溶解被吸收。

大蒜进入花芽和鳞芽分化期，对养分需求增加。此时磷元素至关重要，它能促进花芽分化和蒜头膨大。建议每亩追施过磷酸钙 20-25 公斤，可开沟条施，施后覆土，保证磷元素能被大蒜根系有效吸收，助力其生长发育。

钾元素对于大蒜提高抗逆性和蒜头品质意义重大。在大蒜膨大期，钾素能促进光合产物向蒜头运输。可每亩追施硫酸钾 10-15 公斤，施肥后适当浇水，让土壤中的钾元素以离子态被大蒜吸收，使蒜头饱满。

除了大量元素，大蒜生长也离不开中微量元素。钙能增强细胞壁强度，预防大蒜生理性病害。可在大蒜生长中期，通过叶面喷施氯化钙溶液补充钙元素，浓度控制在 0.3%-0.5%，选择无风晴天的上午或傍晚喷施，利于叶片吸收。
镁元素参与大蒜光合作用，缺镁会导致叶片发黄。在大蒜生长过程中，若发现叶片有缺镁症状，可每亩用硫酸镁 1-2 公斤，溶解后随水冲施，改善叶片光合性能，促进大蒜生长，提高产量。
硼元素能促进大蒜花粉萌发和花粉管伸长，提高坐果率。在大蒜抽薹期，可叶面喷施硼砂溶液，浓度为 0.1%-0.2%，隔 7-10 天喷一次，连喷 2-3 次，有助于大蒜生殖生长，提高蒜头产量和品质。
锌元素对大蒜生长发育也有重要作用，能增强植株抗逆性。在基肥中可每亩添加硫酸锌 1-1.5 公斤，与有机肥充分混合后施入土壤，为大蒜整个生育期提供锌元素，促进其根系和地上部分生长。
大蒜基肥很关键，应重施有机肥。每亩可施入充分腐熟的农家肥 3000-5000 公斤，配合过磷酸钙 50-75 公斤、硫酸钾 20-30 公斤。有机肥能改善土壤结构，增加土壤肥力，为大蒜生长提供长效养分。
大蒜追肥要根据不同生长阶段进行。在返青期，可每亩追施尿素 10-15 公斤，促进蒜苗快速返青生长。施肥时可在行间开浅沟施入，然后覆土，避免肥料挥发损失，提高肥料利用率。
大蒜在蒜薹伸长期，生长迅速，需肥量大。此时应每亩追施高氮高钾复合肥 20-25 公斤，满足蒜薹生长和蒜头膨大对养分的需求，保证蒜薹粗壮、蒜头紧实。
大蒜施肥要注意方法和用量。如果氮肥过量，会导致大蒜叶片徒长，抗逆性下降。因此，要严格按照不同生长阶段的需求施肥，平衡各种养分比例，确保大蒜健康生长。
在大蒜种植前，进行土壤检测十分必要。通过检测了解土壤中各种养分含量，根据检测结果制定科学施肥方案，缺什么补什么，精准施肥，避免盲目施肥造成资源浪费和环境污染。
大蒜根外追肥是补充养分的有效方式。在大蒜生长后期，根系吸收能力减弱，可通过叶面喷施磷酸二氢钾溶液，浓度为 0.2%-0.3%，补充磷钾元素，延长叶片功能期，增加蒜头产量。
微生物菌肥对大蒜生长有良好促进作用。它能增加土壤有益微生物数量，改善土壤微生态环境。在基肥中可添加适量微生物菌肥，每亩用量 1-2 公斤，与有机肥混合使用，提高土壤肥力和肥料利用率。
大蒜不同品种对养分需求略有差异。一些早熟品种生长周期短，前期需肥相对集中；晚熟品种生长周期长，后期需肥量较大。种植时要根据品种特性合理调整施肥方案。
施肥时间对大蒜吸收养分影响较大。一般来说，早上或傍晚施肥效果较好，此时土壤温度和湿度适宜，肥料能较快溶解被吸收，避免中午高温时施肥，防止肥料挥发和烧根。
在大蒜生长过程中，要及时观察植株生长状况。如果发现叶片发黄、生长缓慢等缺素症状，要及时判断缺乏哪种养分，并采取相应的施肥措施进行补充，确保大蒜正常生长。
合理轮作对大蒜营养管理很重要。与非葱蒜类作物轮作，能减少土壤中病虫害积累，改善土壤养分状况。例如，大蒜与玉米轮作，玉米收获后残留的秸秆还田可增加土壤有机质。
大蒜施肥要结合灌溉。干旱时施肥，肥料难以溶解被吸收，还可能造成肥害。在施肥前后要保证土壤有适宜的湿度，可根据天气和土壤墒情及时浇水，促进肥料吸收。
大蒜在越冬前要适当控制施肥量，避免植株徒长，降低抗寒能力。可在越冬前追施一次稀薄的有机肥，增强大蒜抗寒能力，确保其安全越冬。
微量元素肥料虽然用量少，但对大蒜生长作用不可忽视。铁元素参与叶绿素合成，缺铁会导致大蒜叶片失绿发黄。可在出现缺铁症状时，叶面喷施硫酸亚铁溶液，浓度为 0.2%-0.3%。
锰元素对大蒜的光合作用和呼吸作用有影响。当大蒜缺锰时，叶片会出现失绿斑点。可通过叶面喷施硫酸锰溶液补充锰元素，浓度控制在 0.1%-0.2%，隔 7-10 天喷一次。

硫元素是大蒜生长必需的营养元素之一，它能提高大蒜的风味和品质。在基肥中可适当添加含硫肥料，如硫酸钾，既能补充钾元素，又能补充硫元素，使大蒜更具风味。
大蒜施肥要注意肥料的搭配。例如，氮肥与有机肥配合使用，可提高氮肥利用率，减少氮素流失。有机肥中的有机质能吸附氮肥，缓慢释放，为大蒜持续提供养分。
在大蒜种植过程中，可采用分层施肥的方法。将基肥深施，中层施缓效肥料，上层施速效肥料，这样能满足大蒜不同生长阶段根系对养分的需求，促进根系生长和植株发育。
大蒜生长过程中，要避免偏施某一种肥料。单一施肥会导致土壤养分失衡，影响大蒜生长。要均衡供应氮、磷、钾及中微量元素，保证大蒜全面吸收养分，提高产量和品质。
大蒜在幼苗期根系较弱，吸收养分能力有限。此时施肥应以薄肥勤施为原则，避免浓肥烧根。可每隔 7-10 天追施一次稀薄的氮肥溶液，促进幼苗生长。
大蒜在生长后期，根系逐渐衰老，对养分的吸收能力下降。除了叶面喷施肥料外，还可适当减少土壤施肥量，避免肥料浪费和土壤污染，同时防止因施肥过多造成大蒜品质下降。
土壤酸碱度对大蒜养分吸收有影响。大蒜适宜在中性至微酸性的土壤中生长，当土壤过酸或过碱时，会影响某些养分的有效性。若土壤偏酸，可适量施用石灰调节土壤酸碱度。
大蒜种植密度也会影响养分需求。种植密度大，单位面积内大蒜植株多，对养分需求总量增加。要根据种植密度合理调整施肥量，保证每株大蒜都能获得足够的养分。
大蒜在不同生长阶段对养分的吸收速率不同。在生长旺盛期，吸收速率快，需肥量大；在生长缓慢期，吸收速率慢，需肥量相对较少。要根据这一特点合理安排施肥时间和施肥量。
大蒜施肥时，要注意肥料的质量。选择正规厂家生产的肥料，避免使用劣质肥料，以免肥料中养分含量不足或含有有害物质，影响大蒜生长和品质。
大蒜生长需要充足的光照，光照不足会影响光合作用，降低大蒜对养分的利用效率。合理密植和及时清理田间杂草，保证大蒜有充足的光照，促进养分转化和吸收。
大蒜在抽薹期，蒜薹和蒜头同时生长，对养分竞争激烈。此时要保证养分均衡供应，既要满足蒜薹生长对氮素的需求，又要保证蒜头膨大对磷钾元素的需求，通过合理施肥促进二者协调生长。
大蒜施肥后，要注意观察肥料的效果。如果施肥后植株生长状况没有明显改善，要分析原因，可能是施肥方法不当、肥料选择错误或土壤条件不适等，及时调整施肥措施。
大蒜生长过程中，水分管理与施肥密切相关。水分过多或过少都会影响肥料的吸收和利用。保持土壤适度湿润，避免积水和干旱，为大蒜生长创造良好的水分条件，利于肥料发挥作用。
在大蒜种植前，对种子进行处理也能提高其对养分的吸收能力。例如，用微量元素溶液浸种，可增强种子活力，促进种子萌发和幼苗生长，使其在生长初期更好地吸收养分。
大蒜生长过程中，病虫害会影响植株对养分的吸收和利用。及时防治病虫害，减少病虫害对大蒜植株的伤害，保证植株健康生长，提高其对养分的吸收能力。
大蒜在膨大期，除了追施化肥外，还可适量追施饼肥。饼肥含有丰富的有机质和多种养分，能缓慢释放，为蒜头膨大提供长效养分，改善蒜头品质。
大蒜施肥要考虑土壤肥力状况。肥沃的土壤可适当减少施肥量，贫瘠的土壤则要增加施肥量。通过测土配方施肥，根据土壤肥力调整施肥方案，实现科学施肥。
大蒜生长过程中，适时中耕松土能改善土壤通气性，促进根系生长和养分吸收。在施肥后进行中耕松土，可使肥料与土壤充分混合，提高肥料利用率。
大蒜在生长后期，可通过滴灌或喷灌的方式施肥。这种施肥方式能精确控制施肥量和施肥时间，减少肥料浪费，提高肥料利用率，同时节约用水，满足大蒜对水分和养分的需求。
大蒜对硅元素也有一定需求，硅元素能增强大蒜的抗倒伏能力和抗病能力。可在基肥中添加硅肥，每亩用量 10-15 公斤，提高大蒜的抗逆性，促进其生长。
大蒜施肥时，要注意肥料的存放。避免肥料受潮、淋雨，防止肥料变质和养分流失。将肥料

存放在干燥、通风的地方，保证肥料质量，以便在施肥时能发挥其应有的作用。
大蒜生长过程中，可根据植株生长情况进行根外追肥。例如，在大蒜遭受病虫害或自然灾害后，通过叶面喷施肥料，能快速补充养分，促进植株恢复生长。
大蒜在生长初期，根系分布较浅，施肥时应浅施。随着植株生长，根系逐渐向下生长，后期施肥可适当加深，保证肥料能被根系有效吸收。
大蒜不同生长阶段对肥料的形态也有一定要求。在幼苗期，以水溶性肥料为宜，便于根系吸收；在生长后期，可适当施用颗粒状肥料，延长肥料释放时间。
大蒜生长过程中，可结合除草进行施肥。将肥料与除草工作相结合，既能减少劳动力投入，又能使肥料在除草过程中更好地混入土壤，提高肥料利用率。
大蒜施肥要注意与农业措施配合。例如，合理整枝打杈能改善植株通风透光条件，促进光合作用，提高大蒜对养分的利用效率，与施肥措施相互配合，促进大蒜生长。
大蒜在生长过程中，要定期对土壤进行养分监测。根据监测结果及时调整施肥方案，保证土壤养分始终满足大蒜生长需求，实现大蒜持续高产优质。
大蒜在生长旺盛期，对氮肥的需求较大，但也要注意控制氮肥用量。过量施用氮肥会导致大蒜叶片柔软，易倒伏，还会延迟蒜头成熟。应根据大蒜生长状况，合理控制氮肥施用量。
大蒜生长需要多种养分协同作用。例如，磷元素能促进氮元素的吸收和利用，钾元素能增强大蒜对氮、磷的吸收效果。在施肥时要注意各种养分的合理搭配，发挥养分的协同效应。
大蒜在种植过程中，可采用有机无机肥配合的施肥方式。有机肥能改善土壤结构，提高土壤肥力，无机肥能快速补充大蒜生长所需的养分。二者配合使用，既能保证大蒜高产，又能提高土壤质量。
大蒜生长过程中，要注意防止肥料的拮抗作用。例如，钙、镁元素之间存在拮抗关系，过量施用钙肥可能会影响镁元素的吸收。在施肥时要注意控制各种元素的比例，避免出现拮抗现象。
大蒜在生长后期，可适当补充氨基酸类肥料。氨基酸能增强大蒜的抗逆性，提高大蒜品质。通过叶面喷施氨基酸叶面肥，可延长叶片功能期，增加蒜头产量。
大蒜施肥时，要根据当地的气候条件进行调整。在干旱地区，施肥后要及时浇水，促进肥料溶解和吸收；在多雨地区，要注意防止肥料流失，可适当增加施肥次数，减少每次施肥量。
大蒜在生长过程中，可利用生物固氮技术补充氮素。例如，与豆科植物间作，豆科植物的根瘤菌能固定空气中的氮素，为大蒜提供部分氮源，减少氮肥施用量。
大蒜不同生长阶段对养分的敏感度不同。在花芽分化期，对磷、硼等元素敏感度较高，此时保证这些元素的充足供应，能促进花芽分化，提高大蒜产量和品质。
大蒜施肥要注意肥料的施用深度。基肥一般深施 15-20 厘米，追肥根据不同生长阶段和肥料种类，施肥深度在 5-15 厘米不等，保证肥料能被根系充分吸收。
大蒜生长过程中，可通过土壤调理剂改善土壤环境，促进养分吸收。例如，施用土壤调理剂能调节土壤酸碱度，增加土壤阳离子交换量，提高土壤保肥保水能力，为大蒜生长创造良好的土壤条件。
大蒜在生长初期，可适当施用腐植酸肥料。腐植酸能刺激大蒜根系生长，提高根系活力，增强大蒜对养分的吸收能力，促进幼苗健壮生长。
大蒜不同生长阶段的养分需求特点：大蒜在不同生长阶段对养分需求有明显差异。萌芽期主要依靠种蒜自身营养，对外部养分吸收少。幼苗期生长缓慢，需氮较多，适量磷钾可促进根系发育，此时氮磷钾比例约为 3:1:2。鳞茎膨大期是需肥高峰期，对钾需求剧增，氮磷钾比例调整为 2:1:3，充足钾素能使蒜头饱满、产量提高。
基肥对大蒜生长的重要性及施用方法：基肥是大蒜生长的基础养分来源。应以有机肥为主，如充分腐熟的农家肥、堆肥等，配合适量化肥。一般每亩施农家肥 3000-5000 千克，过磷酸

钙 30-50 千克，硫酸钾 15-20 千克。将肥料均匀撒施后深耕翻入土中，可改善土壤结构，为大蒜整个生育期提供长效养分支持，促进根系和植株早期健壮生长。

大蒜苗期追肥技巧与养分补给：大蒜苗期追肥要早，以促进幼苗快速生长。在苗高 10-15 厘米时，每亩追施高氮复合肥 10-15 千克，可结合浇水冲施。这次追肥能满足幼苗对氮素的需求，加快叶片生长，增强光合作用。同时，可适当补充微量元素肥料，如硼肥和锌肥，每亩用硼砂 0.5-1 千克、硫酸锌 1-1.5 千克，对提高蒜苗品质和抗病能力有积极作用。

大蒜鳞茎膨大期施肥要点与产量关系：鳞茎膨大期是大蒜产量形成的关键时期。此时应重施追肥，以高钾肥料为主。每亩追施硫酸钾复合肥 20-30 千克，分 2-3 次施用，每次间隔 10-15 天。充足的钾肥能促进鳞茎细胞分裂和膨大，提高大蒜的重量和品质。同时，保持土壤湿润，有利于肥料的溶解和吸收，对提高大蒜产量至关重要。

大蒜叶面施肥的优势与操作方法：叶面施肥是大蒜营养补充的有效辅助手段。在大蒜生长中后期，根系吸收能力减弱，通过叶面喷施肥料能快速补充养分。可选用磷酸二氢钾溶液，浓度为 0.2%-0.3%，每隔 7-10 天喷施一次，共喷 2-3 次。还可根据大蒜生长状况添加微量元素叶面肥，如缺铁时喷施硫酸亚铁溶液。叶面施肥能提高肥料利用率，增强大蒜抗逆性，改善大蒜品质。

合理轮作对大蒜营养吸收和土壤肥力的影响：合理轮作能改善大蒜营养吸收环境和维持土壤肥力。大蒜不宜连作，与小麦、玉米等禾本科作物轮作效果较好。轮作可减少土壤中大蒜病虫害残留，避免土壤养分单一消耗。不同作物对养分需求不同，轮作后土壤中各种养分能得到均衡利用，为大蒜生长创造良好的土壤条件，有利于大蒜吸收全面的养分，提高产量和品质。

土壤酸碱度对大蒜养分有效性及施肥的影响：大蒜适宜在中性至微酸性土壤中生长，土壤酸碱度影响养分的有效性。在酸性土壤中，铁、铝等元素溶解度增加，可能对大蒜产生毒害；在碱性土壤中，磷、铁、锌等元素易被固定，难以被大蒜吸收。当土壤偏酸时，可适量施用石灰调节；偏碱时，增施有机肥和酸性肥料改良。根据土壤酸碱度合理施肥，能提高肥料利用率，满足大蒜对各种养分的需求。

大蒜施肥量与产量品质的量化关系研究：研究表明，大蒜施肥量与产量品质密切相关。在一定范围内，随着施肥量增加，大蒜产量呈上升趋势，但过量施肥会导致产量下降，品质变差。例如，当每亩纯氮施用量在 18-22 千克、五氧化二磷 8-12 千克、氧化钾 15-20 千克时，大蒜产量较高且品质优良。合理控制施肥量，能实现大蒜产量和品质的最佳平衡。

有机肥料在大蒜种植中的独特作用与使用注意：有机肥料在大蒜种植中有独特作用。它不仅能提供全面养分，还能改善土壤结构，增加土壤保水保肥能力。如施用腐熟的鸡粪，能为大蒜提供丰富的氮、磷、钾及微量元素。但使用时要注意充分腐熟，否则易烧根，还可能携带病菌和虫卵。同时，有机肥料与化肥配合使用，可取长补短，提高肥料整体效果。

大蒜微量元素肥料的精准施用策略：大蒜生长除需要大量元素外，微量元素也不可或缺。硼能促进大蒜生殖生长，提高结实率；锌能增强大蒜抗逆性和光合作用。在大蒜苗期和抽薹期，可分别喷施含硼、锌的微量元素叶面肥。根据土壤检测结果精准施用，缺硼土壤每亩基施硼砂 0.5-1 千克，锌缺乏时每亩基施硫酸锌 1-1.5 千克，避免盲目施用造成浪费或肥害。

不同灌溉条件下大蒜施肥的调整方法：灌溉条件影响大蒜对肥料的吸收利用。在干旱地区，肥料不易溶解和被根系吸收，施肥应结合灌溉，采用少量多次的方式。如滴灌时，可将肥料配制成适宜浓度的溶液随水滴灌，提高肥料利用率。在湿润地区，要注意防止肥料淋失，适当减少施肥量，避免养分流失污染环境，同时保证大蒜获得充足养分。

大蒜生长后期根系老化后的营养管理措施：大蒜生长后期根系老化，吸收能力减弱。此时除叶面施肥外，可通过养根护根措施改善根系功能。如冲施含腐植酸、氨基酸的水溶肥，能刺激根系生长，增强根系活力。同时，保持土壤疏松透气，避免土壤板结，为根系创造良好的

生长环境，维持大蒜对养分的吸收，保证蒜头膨大所需营养。
大蒜基肥中化肥与有机肥配合比例的优化：基肥中化肥与有机肥配合比例对大蒜生长影响显著。经试验，有机肥与化肥以 3:1 的比例配合效果较好。过多施用化肥会导致土壤板结，影响大蒜根系生长；有机肥过多，前期养分供应不足。按此比例配合，既能保证前期养分供应，又能长期改善土壤环境，为大蒜生长提供稳定的养分来源，促进大蒜植株健壮生长。
大蒜在连作障碍土壤中的施肥改良方案：在连作障碍的大蒜田，土壤中病菌积累、养分失衡。施肥时应增施生物菌肥，如含有枯草芽孢杆菌、放线菌等有益菌的菌肥，每亩施用 2-3 千克，可抑制有害病菌生长。同时，补充土壤中缺乏的微量元素，调节氮磷钾比例，适当减少氮肥用量，增加磷钾肥。通过这些施肥改良措施，缓解连作障碍，促进大蒜生长。
大蒜施肥与病虫害防治的协同关系：合理施肥能增强大蒜的抗病虫害能力。充足的氮磷钾供应使大蒜植株生长健壮，抵抗病虫害的能力提高。例如，适量钾素能使大蒜细胞壁增厚，增强对病害的抵抗力。同时，施肥不当也可能诱发病虫害，如偏施氮肥会使大蒜叶片嫩绿，易吸引蚜虫等害虫。因此，要平衡施肥，并结合病虫害防治措施，保障大蒜健康生长。
大蒜种植中新型肥料的应用效果与前景：新型肥料如缓控释肥、水溶肥在大蒜种植中有良好应用效果。缓控释肥能根据大蒜生长需求缓慢释放养分，减少施肥次数，提高肥料利用率，一次基施可满足大蒜大部分生育期需求。水溶肥溶解性好，能通过滴灌、喷灌等方式精准施肥，尤其在干旱地区节水节肥效果明显。随着农业技术发展，新型肥料在大蒜种植中前景广阔。
大蒜不同品种对养分需求的差异及施肥对策：不同大蒜品种对养分需求存在差异。早熟品种生育期短，前期需肥集中，应重施基肥和早期追肥，促进早发快长。晚熟品种生育期长，需肥总量较大，除基肥外，在生长中后期要多次追肥，保证后期养分供应。如紫皮蒜对钾素需求相对较高，白皮蒜对氮素需求稍多，根据品种特性施肥，能提高肥料针对性和大蒜产量品质。
大蒜种植中基肥深施与浅施的效果对比：基肥深施和浅施对大蒜生长效果不同。深施（20-30 厘米）能使肥料与深层土壤充分混合，有利于根系向下生长，提高根系对养分的吸收范围，肥料长效性好。浅施（10-15 厘米）肥料释放快，前期养分供应充足，但后期易脱肥。在实际种植中，可根据土壤质地和大蒜生长需求选择，一般粘质土壤宜深施，砂质土壤可适当浅施。
大蒜追肥时机与大蒜生长发育进程的关联：大蒜追肥时机紧密关联其生长发育进程。在大蒜花芽分化期追肥，能促进花芽分化和蒜薹生长；蒜薹伸长期追肥，可满足蒜薹快速生长对养分的大量需求，使蒜薹粗壮。鳞茎膨大初期追肥，为蒜头膨大奠定营养基础。错过关键追肥时机，会影响大蒜生长，如蒜薹伸长期缺肥，蒜薹细弱、产量低。
大蒜种植中土壤肥力监测与精准施肥的实施：定期进行土壤肥力监测是大蒜精准施肥的基础。通过检测土壤中的有机质、氮、磷、钾及微量元素含量，了解土壤养分状况。根据检测结果制定施肥方案，缺什么补什么，缺多少补多少。例如土壤中速效磷含量低，可适当增加磷肥施用量。精准施肥避免了肥料浪费和过量施用造成的环境污染，同时保障大蒜获得充足且合理的养分。
当大蒜处于幼苗期，此时根系较脆弱，对养分的吸收能力有限。在保水性较好的黏土种植，可每亩追施 8 公斤尿素，补充氮素，促进叶片生长。施肥时在距离蒜苗根部 6 厘米处挖浅穴施入，然后覆土，避免肥料直接接触根系，施肥后适量浇水，使肥料缓慢溶解，便于幼苗吸收。
蒜薹伸长期是大蒜需肥的关键时期之一。在沙壤土种植，因沙壤土保肥性差，除了追施 12 公斤硫酸钾促进蒜薹粗壮生长外，还需每隔 7 天喷施一次 0.3%的磷酸二氢钾溶液，增强叶片光合作用，为蒜薹生长提供更多能量，提升蒜薹的品质与产量。

大蒜鳞茎膨大期，对钾元素需求旺盛。在酸性土壤中种植，为调节土壤酸碱度并补充养分，可每亩施入 15 公斤草木灰，同时追施 10 公斤硫酸钾和 5 公斤尿素。草木灰不仅提供钾元素，还能改善土壤酸性环境。将肥料混合均匀后，在大蒜行间开沟施入，沟深 8-10 厘米，随后覆土并浇透水，促进蒜头膨大。
微量元素对于大蒜生长至关重要。在石灰性土壤种植大蒜，易出现缺铁现象，可在大蒜生长中期，每隔 10 天叶面喷施一次 0.2%的硫酸亚铁溶液，连喷 3 次。选择阴天或晴天下午 4 点后喷施，重点喷施叶片背面，提高铁元素的吸收效率，防止叶片黄化，保障光合作用正常进行。
大蒜返青期，在北方寒冷地区，土壤刚刚解冻。此时每亩追施 10 公斤高氮复合肥，快速补充养分，促进蒜苗快速返青生长，增强其抗寒能力。施肥后及时浇水，提高地温，使肥料尽快发挥作用，让大蒜顺利度过返青阶段，进入快速生长时期。
大蒜花芽分化期，在南方温暖湿润地区种植，土壤湿度较大。可每亩追施 10 公斤过磷酸钙，促进花芽分化，保证蒜薹和蒜头的正常发育。采用条施的方式，在大蒜植株旁开浅沟施入，然后覆土，避免肥料在高湿度环境下流失过快，确保磷元素有效供给。
在盐碱地种植大蒜，为减轻土壤盐分对大蒜生长的影响，基肥可增加有机肥用量至每亩 4000 公斤，同时添加 50 公斤硫酸钾型复合肥。有机肥能改善土壤结构，降低土壤盐分浓度。硫酸钾型复合肥避免氯离子对大蒜的伤害。将肥料均匀撒施后深翻 25 厘米，使肥料与土壤充分融合，为大蒜生长创造良好的土壤环境。
大蒜生长后期，根系吸收能力减弱。在地势低洼易积水的地块种植，为补充养分，可进行叶面施肥。用 0.5%的尿素溶液和 0.3%的磷酸二氢钾溶液混合喷施，每隔 5 天喷一次，共喷 3 次。选择无风晴天上午 10 点前喷施，提高叶片的光合效率，延长叶片功能期，促进蒜头充实饱满。
大蒜抽薹前，在中等肥力的土壤种植，为保证蒜薹顺利抽出并生长，每亩追施 10 公斤尿素和 8 公斤硫酸钾。尿素提供氮素促进植株生长，硫酸钾提供钾元素增强植株抗逆性，促进蒜薹生长。将肥料混合后在大蒜植株周围挖穴施入，穴深 5-7 厘米，然后覆土浇水，满足大蒜抽薹期的养分需求。
大蒜播种前，先将土壤深耕 30 厘米，每亩均匀撒施 4000 公斤充分腐熟的鸡粪，再搭配 50 公斤硫酸钾复合肥作为基肥。鸡粪能有效改善土壤结构，增加土壤保肥保水能力，复合肥提供全面养分，为大蒜整个生长周期奠定良好基础。
大蒜处于幼苗期，当苗高 10-15 厘米时，在距离植株根部 8-10 厘米处挖穴，每亩追施 10 公斤尿素。施后及时覆土并浇水，让尿素快速溶解，被根系吸收，助力蒜苗叶片生长，提高其抗寒能力。
进入蒜薹伸长期，大蒜对钾元素需求激增。此时每亩追施 15 公斤硫酸钾，采用条施的方式，在大蒜行间开 5-7 厘米深的浅沟，将硫酸钾施入后覆土，促进蒜薹伸长、增粗，提升蒜薹品质和产量。
大蒜鳞茎膨大初期，每亩施 12 公斤硫酸钾与 8 公斤尿素的混合肥。施肥后立即浇水，使肥料迅速渗透到根系周围，满足蒜头膨大对养分的需求，促进碳水化合物积累，让蒜头饱满。
大蒜生长后期，根系活力下降，采用叶面施肥补充养分。将 0.3%的磷酸二氢钾溶液与 0.2%的尿素溶液混合，每隔 7 天喷施一次，共喷 3 次，重点喷施叶片背面，增强叶片光合作用，防止植株早衰。
为改善土壤微生态环境，播种前每亩撒施 200 公斤生物菌肥，然后翻耕入土。生物菌肥能增加土壤有益微生物数量，活化土壤养分，提高肥料利用率，为大蒜生长创造良好土壤环境。
大蒜苗期若发现叶片发黄、生长缓慢，可能是缺乏微量元素。每亩追施 5 公斤硫酸亚铁和 3 公斤硼砂的混合微肥，在植株周围开浅沟施入，覆土后浇水，促进叶片转绿，保证植株正常

生长。
当大蒜进入花芽分化期，为促进花芽分化和后续蒜薹、蒜头发育，每亩追施 10 公斤高磷复合肥。采用穴施，每穴施肥量均匀，然后覆土踏实，确保磷元素有效供给。
大蒜生长中期，为增强植株抗逆性，提高抗病能力，每隔 10 天喷施一次氨基酸叶面肥，每次每亩用量为 500 克，稀释成 500 倍液进行喷雾，均匀覆盖叶片，使植株健壮生长。
为预防大蒜在生长过程中出现缺素症，在基肥中加入 5 公斤硫酸镁和 3 公斤硫酸锌。将这些中微量元素肥与有机肥、复合肥充分混合后施入土壤，保证大蒜对镁、锌等元素的需求。
大蒜抽薹前，每亩追施 10 公斤尿素和 10 公斤硫酸钾，采用撒施的方式，撒施后及时浇水，使肥料快速溶解，为蒜薹生长提供充足养分，促进蒜薹快速抽出。
大蒜鳞茎膨大中期，再次追施 10 公斤硫酸钾，补充钾元素，促进蒜头进一步膨大。此次施肥可采用冲施的方式，随水冲入田间，确保肥料均匀分布。
播种前，将土壤整细耙平，每亩施入 3000 公斤绿肥，然后翻耕 25 厘米，使绿肥与土壤充分混合。绿肥在土壤中分解，能增加土壤有机质含量，改善土壤结构，提高土壤肥力。
大蒜生长早期，若土壤肥力较低，可在浇水时每亩冲施 3-5 公斤高氮水溶肥，每隔 5-7 天冲施一次，共冲施 2-3 次，快速补充养分，促进幼苗生长。
为防止大蒜出现徒长现象，在叶片生长过旺时，可喷施矮壮素进行调控。将矮壮素稀释成 1000-1500 倍液，均匀喷施在叶片上，抑制茎叶生长，促进养分向蒜头转移。
大蒜生长过程中，若遭遇连续阴雨天气，土壤湿度大，养分易流失。雨停后，每亩撒施 5-8 公斤尿素进行追肥，补充流失的养分，保证植株正常生长。
大蒜进入膨大后期，为提高蒜头的耐储存性，叶面喷施 0.5%的氯化钙溶液，每隔 5 天喷施一次，共喷 2 次，增加蒜头细胞壁的韧性，延长储存时间。
为提高大蒜产量和品质，在基肥中加入 100 公斤花生饼肥。花生饼肥营养丰富，肥效持久，可在土壤中缓慢释放养分，为大蒜生长提供长效营养支持。
大蒜苗期，结合中耕除草，每亩追施 8 公斤碳酸氢铵。将碳酸氢铵均匀撒在植株周围，然后中耕，使肥料与土壤混合，为蒜苗生长提供养分，同时减少肥料挥发。
大蒜生长中期，若发现土壤板结，可每亩施入 150 公斤土壤调理剂。将土壤调理剂撒施后翻耕入土，改善土壤结构，增加土壤透气性，促进根系生长。
为促进大蒜根系生长，在播种前用生根剂溶液浸泡蒜种 3-5 小时。生根剂能刺激蒜种萌发新根，提高根系活力，增强植株对养分和水分的吸收能力。
大蒜蒜薹伸长期，为防止蒜薹弯曲、折断，每亩追施 5 公斤磷酸二氢钾。采用叶面喷施的方式，稀释成 0.3%-0.5%的溶液，均匀喷施在叶片和蒜薹上，增强蒜薹的韧性。
大蒜生长后期，为促进蒜头成熟，可喷施乙烯利。将乙烯利稀释成 800-1000 倍液，在收获前 10-15 天进行叶面喷施，加快蒜头成熟，提高收获效率。
播种前，每亩施入 2000 公斤蚯蚓粪和 30 公斤三元复合肥。蚯蚓粪富含多种有益微生物和微量元素，能改善土壤环境，与复合肥配合，为大蒜生长提供全面养分。
大蒜生长过程中，定期检测土壤养分含量。当土壤中氮含量低于 100mg/kg 时，及时追施氮肥；磷含量低于 30mg/kg 时，追施磷肥；钾含量低于 150mg/kg 时，追施钾肥，确保土壤养分平衡。
大蒜幼苗期，为预防病虫害，可在施肥时加入生物防治剂。如每亩加入 1 公斤枯草芽孢杆菌，与肥料混合后施入土壤，抑制土壤中的有害病菌，降低病虫害发生几率。
大蒜鳞茎膨大期，若发现蒜头大小不均，可对较小的蒜头进行单独施肥。在其周围挖穴，施入 20-30 克高钾复合肥，然后覆土浇水，促进小蒜头快速生长，提高整体产量。
为提高大蒜的商品性，在收获前 20 天停止追施氮肥，增加钾肥施用量。每亩追施 10 公斤硫酸钾，促进蒜头淀粉积累，使蒜头表皮光滑，色泽鲜亮。

大蒜生长早期，若气温较低，可在施肥时加入适量的腐植酸。每亩施入 50 公斤腐植酸肥，与基肥混合施入，提高地温，促进大蒜根系生长，增强植株抗寒能力。

大蒜生长过程中，可采用滴灌施肥的方式。根据大蒜不同生长阶段的养分需求，将肥料溶解在水中，通过滴灌系统精准施入，提高肥料利用率，节约水资源。

大蒜的氮肥需求：大蒜生长前期（苗期至鳞茎膨大前）对氮肥需求较高，氮元素能促进叶片生长，增强光合作用。建议每亩基施尿素 15-20 公斤或腐熟有机肥 2000-3000 公斤，追肥分 2-3 次进行，避免一次性过量导致徒长。

磷肥促进根系发育：磷肥有助于大蒜根系生长和鳞茎膨大，缺磷会导致植株矮小、产量降低。基肥中可加入过磷酸钙 30-40 公斤/亩，或磷酸二铵 15-20 公斤，尤其在土壤贫瘠地块需重点补充。

钾肥提升抗逆性：　　钾元素能增强大蒜抗病、抗寒能力，并促进鳞茎饱满。建议在鳞茎膨大期追施硫酸钾 10-15 公斤/亩，或草木灰 100-150 公斤，避免使用氯化钾以防氯害。

中微量元素不可忽视：钙、镁、硫、硼等中微量元素对大蒜品质至关重要。缺钙易引发叶尖干枯，缺硼会导致鳞茎畸形。可通过叶面喷施钙镁肥或 0.2%硼砂溶液补充。

有机肥改良土壤：腐熟农家肥（如羊粪、鸡粪）能改善土壤结构，提供长效养分。每亩施 2000-3000 公斤作基肥，需充分腐熟以避免烧根和病虫害传播。

基肥深施提高利用率：基肥应深翻入土 20-30 厘米，与土壤混合均匀，避免肥料集中导致烧苗。氮肥可浅施，磷钾肥深施以利于根系吸收。

追肥分阶段进行：大蒜追肥分 3 次：返青期（氮为主）、蒜薹伸长期（氮钾结合）、鳞茎膨大期（钾为主）。每次间隔 15-20 天，结合浇水提高肥效。

避免过量施氮：氮肥过量会导致叶片过旺、鳞茎膨大延迟，且易倒伏。控制尿素用量不超过 25 公斤/亩，后期减少氮肥比例。

叶面肥快速补肥：生长中后期可通过叶面喷施 0.3%磷酸二氢钾+0.1%尿素溶液，每 10 天一次，连喷 2-3 次，缓解根系吸收不足的问题。

酸性土壤调酸补钙:pH 值低于 5.5 的土壤需施石灰 50-100 公斤/亩调节酸碱度，同时补充钙肥，预防大蒜干尖病和鳞茎软腐。

蒜薹期加强钾肥:蒜薹抽生期对钾需求激增，及时追施硫酸钾或高钾复合肥 10-15 公斤/亩，促进蒜薹粗壮和鳞茎积累干物质。

滴灌施肥精准高效:采用滴灌系统可结合水溶性肥料（如 20-20-20 平衡肥），按需分次施入，减少浪费，尤其适合规模化种植。

忌氯作物慎用含氯肥:大蒜对氯敏感，长期使用氯化钾或含氯复合肥易导致叶片发黄、鳞茎变小，优先选择硫酸钾型肥料。

覆盖秸秆保肥保湿:播种后覆盖稻草或麦秸，可减少水分蒸发，抑制杂草，秸秆腐烂后还能补充有机质，提高土壤肥力。

轮作减少养分耗竭:避免连作，与豆科、禾本科作物轮作，可减少土传病害并平衡土壤养分，尤其缓解大蒜对钾的过度消耗。

测土配方科学施肥:种植前检测土壤养分含量，根据结果调整氮磷钾比例。例如，富磷土壤可减少磷肥用量，缺钾地块增施钾肥。

鳞茎膨大期控水控氮:鳞茎形成后减少灌水和氮肥，避免贪青晚熟，同时增施钾肥促进干物质转运，提高大蒜耐储性。

生物菌肥增效提质:配合枯草芽孢杆菌、解磷菌等生物菌肥使用，可活化土壤养分，减少化肥用量 20%-30%，提升大蒜风味。

防早衰后期补肥：生长后期根系老化，可通过叶面喷施氨基酸肥或腐殖酸肥延缓叶片衰老，延长光合作用时间，增加产量。

.收获前停肥控水：收获前 10-15 天停止施肥和浇水，降低鳞茎含水量，促进蒜皮干燥，提高商品性和贮藏稳定性。
硫元素对风味的影响：大蒜是喜硫作物，硫能增强辛辣味和香气。缺硫时叶片变黄、鳞茎变小。可通过基施硫酸钾或硫磺粉（10-15 公斤/亩）补充，避免使用无硫复合肥。
镁肥预防叶片早衰：镁是叶绿素核心成分，缺镁时老叶脉间黄化。可在抽薹期喷施 0.2%硫酸镁溶液，或基施镁石灰（含氧化镁）50 公斤/亩，尤其适用于沙质土壤。
锌肥促生长抗病毒：缺锌会导致大蒜植株矮小、叶片扭曲。播种前用 0.1%硫酸锌溶液浸种 12 小时，或生长期叶面喷施，可提高抗花叶病毒能力。
.铁肥矫正黄叶：碱性土壤易缺铁，新叶发黄（叶脉仍绿）。可通过滴灌螯合铁（EDTA-Fe）或叶面喷施 0.05%硫酸亚铁+0.1%柠檬酸，每周 1 次连喷 2 次。
钼肥提升氮利用率：钼能促进硝酸盐转化，缺钼时叶片出现鞭状卷曲。建议用 0.01%钼酸铵溶液拌种，或苗期叶面喷施，尤其在酸性土壤中效果显著。
硅肥增强抗逆性：硅元素使细胞壁加厚，减少病虫害侵染。可基施硅钙肥（50 公斤/亩）或生长期喷施硅酸钾，显著降低蓟马和叶枯病发生率。
铜肥抑制真菌病害：缺铜时叶片失绿萎蔫，抗病力下降。播种前用 0.1%硫酸铜溶液浸种，或结合杀菌剂喷施，可预防霜霉病和紫斑病。
锰肥调节酶活性：缺锰时新叶出现网状黄斑。在 pH>6.5 的土壤中，通过叶面喷施 0.1%硫酸锰（加 0.1%熟石灰防药害），每 10 天 1 次。
生物炭改良连作土壤：每亩施 500-1000 公斤生物炭，可吸附重金属、调节土壤微生态，缓解连作障碍，尤其适合设施大蒜栽培。
控释肥减少追肥次数：选用包膜控释氮肥（如脲甲醛）作基肥，养分释放周期 3-4 个月，可减少 30%追肥量，避免淋溶损失。
氨基酸肥缓解胁迫：遭遇冻害或干旱时，喷施 5%氨基酸水溶液（含脯氨酸、甘氨酸），能增强细胞渗透调节能力，加速恢复生长。
腐殖酸提高肥效：将腐殖酸（3-5 公斤/亩）与化肥混施，可螯合养分、刺激根系发育，磷肥利用率提高 20%以上。
种肥隔离防烧苗：播种时若使用复合肥作种肥，需确保种子与肥料间隔 5 厘米以上，防止高浓度盐分抑制发芽。
分蘖期平衡施肥：大蒜分蘖期（4-5 叶）需均衡养分，每亩追施氮磷钾 15-15-15 复合肥 10 公斤，促进有效分蘖形成。
防早抽薹的氮调控：春大蒜易早抽薹，可通过降低苗期氮肥用量（基氮减 20%）、增施磷钾肥延迟花芽分化。
微量元素套餐施肥：采用"锌+硼+钼"组合方案：播种前拌种用锌，返青期喷硼，抽薹期补钼，全面提升产量和品质。
水肥一体化精准调控：滴灌系统配置 A/B 肥罐，前期用 20-10-10 高氮肥，后期切换 10-5-30 高钾肥，实现按需供给。
忌用未腐熟粪肥：生粪发酵产生高温和有害气体，会导致大蒜烂根、黄叶。必须堆沤 60 天以上，以杀灭病原菌和草籽。
叶面肥喷施时间：选择无风晴天上午 9 点前或下午 4 点后喷施，避开高温强光时段，添加有机硅助剂提高附着力。
营养诊断指导施肥：定期取样叶片进行营养分析（临界值：N3.5-4.5%、P0.3-0.5%、K2.5-3.5%），动态调整施肥方案。
苗期低温期施肥管理：大蒜苗期遇低温（低于 10℃）时，根系吸收能力下降。建议叶面喷施 0.3%磷酸二氢钾+0.1%尿素溶液，每 7-10 天一次，连续 2-3 次，可增强抗寒性。同时可配合

施用腐殖酸类肥料，提高地温 1-2℃。
鳞茎膨大期钙肥补充：鳞茎膨大期对钙需求增加，缺钙易导致鳞茎开裂。可在膨大初期土壤追施硝酸钙 10-15 公斤/亩，或叶面喷施 0.3%糖醇钙，间隔 7 天喷 2 次。注意避免与磷酸盐肥料混用，以防沉淀。
有机无机肥配施技术：建议采用有机肥与化肥 7:3 比例配施。每亩用腐熟有机肥 2000 公斤配施 45%复合肥（15-15-15）30 公斤作基肥，既保证速效养分供应，又能改善土壤结构。
微量元素螯合肥应用：使用 EDTA 螯合态的微量元素肥料（如铁、锌、锰等），其吸收利用率比无机盐提高 30%以上。建议每亩基施 1-2 公斤螯合微肥，或在关键生长期叶面喷施。
土壤调理剂配合施肥：在板结或酸化土壤中，配合施用土壤调理剂（如硅钙镁肥）50-100 公斤/亩，可调节 pH 值至 6.0-7.0 的适宜范围，同时补充中量元素，提高肥料利用率 15%以上。
不同品种的施肥差异：白皮蒜需肥量比紫皮蒜高 20%左右，尤其对钾肥更敏感。建议白皮蒜在鳞茎膨大期增施钾肥 5 公斤/亩，而紫皮蒜则需注重硫肥补充。
滴灌追肥浓度控制：采用滴灌系统追肥时，肥料溶液 EC 值控制在 1.5-2.0mS/cm 为宜。建议使用全水溶性肥料，每次每亩用肥量不超过 5 公斤，防止烧根。
生物刺激素应用技术：在逆境（干旱、盐碱等）条件下，配合施用海藻酸、氨基酸类生物刺激素，可激活植物抗逆机制。建议每亩用海藻精 50ml 兑水喷施，每 15 天一次。
精准变量施肥技术：利用土壤养分检测仪和变量施肥机，实现田块内不同区域的精准施肥。高产区域适当减氮增钾，低产区域增施有机肥改良。
肥料深施机械应用：采用深松施肥一体机，将基肥施入 15-20cm 土层，比表施提高氮肥利用率 10%，尤其适合砂质土壤。
缓释肥与速效肥配比：建议基肥中缓释肥占比 60%（如树脂包衣尿素），速效肥 40%（如硫酸铵）。这种配比可保证养分持续供应，减少追肥次数。
叶面肥 pH 值调节：配制叶面肥时，将溶液 pH 调至 5.5-6.5（用柠檬酸调节），可提高叶片对养分的吸收率。避免在碱性条件下喷施微量元素肥料。
有机肥替代部分化肥：通过施用高品质有机肥（如豆粕、菜籽饼等），可替代 30%的化学氮肥。建议每亩用 200 公斤饼肥+20 公斤复合肥作基肥。
追肥时期诊断指标：根据植株形态确定追肥时机：当最外层叶片开始褪绿时追氮肥；假茎基部增粗时追钾肥；蒜薹露出时补微肥。
肥料与农药协同施用：在防治病虫害时，可加入尿素（0.5%）或磷酸二氢钾（0.3%）进行药肥混施，既能补充养分，又能提高农药展着性。
不同土质的施肥策略：黏质土壤采用"少量多次"施肥原则；砂质土壤增加有机肥用量；盐碱地选用生理酸性肥料（如硫酸铵）。
营养元素协同增效：注意养分平衡：氮磷比控制在 1:0.4-0.6，氮钾比 1:0.8-1.2。失衡会导致元素拮抗，如过量钾会抑制镁的吸收。
功能性肥料选择：选用添加聚谷氨酸、腐殖酸等功能性成分的复合肥，可提高肥料利用率 20%以上，尤其在逆境条件下效果显著。
施肥与水分管理配合：施肥后及时灌溉，保持土壤含水量在 60-70%田间持水量。但鳞茎成熟期要控水，以防裂球和病害发生。
营养诊断施肥法：采用 SPAD 叶绿素仪监测叶片氮素状况，当读数低于 45 时立即补氮；用火焰光度计测定植株钾含量，低于 2%时需追钾肥。
返青期"促控结合"施肥技术：大蒜返青期需采取"促根控叶"策略。每亩追施硝酸铵钙 10kg（提供速效氮和钙）配合磷酸二氢钾 3kg，促进根系发育的同时避免叶片徒长。此时土壤温度仍较低，建议采用硝态氮肥更易吸收。
鳞茎分化期关键营养调控：鳞茎开始分化时（约 8 叶期）是品质形成关键期。需提高钾、硫

供应，每亩追施硫酸钾 15kg+硫磺粉 5kg。此时叶面喷施 0.2%钼酸铵可促进同化物转运，增加鳞茎比重。
有机无机复合微生物肥应用：选用含有固氮菌、解磷菌的复合微生物肥料（有效菌≥2 亿/g）作基肥，每亩 80-100kg。与常规化肥相比，可减少 20%化肥用量，且能显著降低土传病害发生率。
膜下滴灌施肥技术参数：地膜覆盖配合滴灌时，肥料溶液浓度控制在 0.3%-0.5%，EC 值 1.2-1.8mS/cm。采用"少量多次"原则，全生育期滴施 8-10 次，每次每亩用肥 3-5kg。特别注意膨大期增加钾肥比例。
镁硫协同施肥方案：在缺镁、缺硫土壤（南方红壤区多见）采用镁硫配施：基施硫镁肥（含 MgO15%，S20%）50kg/亩，追肥时用硫酸钾镁 10kg/亩。可同时矫正两种元素缺乏，提高肥料协同效应。
不同茬口施肥量调整：春大蒜比秋大蒜减少基肥用量 20%，但增加追肥次数。因春蒜生长期短，需采用"前轻后重"施肥法，将 60%氮肥放在返青后施用，促进快速生长。
功能性氨基酸肥应用：选用含γ-氨基丁酸、脯氨酸等功能性氨基酸的叶面肥（浓度 0.3%），在逆境（低温、干旱）时喷施，可激活抗逆基因表达，提高植株抗性。与常规肥料相比，增产 8-12%。
中微量元素套餐施肥：设计"钙镁硼锌"组合方案：基施钙镁磷肥 50kg，返青期喷 0.2%硼砂+0.1%硫酸锌，抽薹期补 0.3%糖醇钙。该方案可全面预防生理性病害，使商品率提高 15%以上。
精准叶面营养诊断：采用便携式 X 射线荧光仪（PXRF）快速检测叶片养分，当检测值低于临界值（硫 0.3%、镁 0.2%、锌 25mg/kg）时立即补充。比传统化验节省 3-5 天时间，实现实时调控。
缓释肥与生物刺激素组合：将聚合物包膜尿素（释放期 90 天）与海藻提取物（含甜菜碱、甘露醇）配合使用。基施时按 3:1 比例混合，可延长肥效期，同时增强根系活力，特别适合规模化种植
土壤酶活性调控施肥：根据土壤酶检测结果调整施肥：脲酶活性高时减少尿素用量；磷酸酶活性低时增施有机磷肥。维持土壤中性磷酸酶在 80-120μg/g·h 最利于磷素活化。
不同密度下的施肥调整：高密度种植（株距 8cm）时增加 20%钾肥用量，因群体内光照不足易导致徒长。可采用"立体施肥"法：表层 5cm 施钾肥，深层 15cm 施磷肥，满足不同层次根系需求。
水肥气热协同管理：将灌溉（保持土壤含水量 60-70%）、施肥（EC 值 1.5-2.0）、中耕（收获前 20 天断根）和地温（覆盖黑色地膜提高 2-3℃）四项措施协同实施，可提升肥料利用率至 65%以上。
有机替代梯度施肥技术：按照有机肥替代化肥比例梯度（0%、30%、50%、70%）制定方案。建议普通地块采用 30%替代（亩施腐熟粪肥 1500kg+化肥减量 30%），既保证产量又改善品质。
抗重茬专用肥配方：针对连作地块，选用含枯草芽孢杆菌（≥5 亿/g）、腐殖酸（≥30%）的抗重茬肥。基施 100-120kg/亩，可有效缓解自毒物质危害，降低根腐病发生率 40%以上。
品质调控施肥技术：为提高大蒜素含量，在鳞茎膨大期增施硫肥（元素硫 10kg/亩）并控制氮肥。收获前 30 天喷施 0.1%硒肥，可同步提升功能成分含量和贮藏性。
机械化深施追肥技术：使用带有开沟器的追肥机，将肥料施入 10-15cm 土层，比表施减少氨挥发损失 30%。追肥后立即覆土镇压，尤其适合砂质土壤保肥。
智能施肥决策系统：应用基于物联网的智能施肥系统，通过土壤传感器实时监测 N、P、K 动态，结合大蒜生长模型，每 7 天生成施肥处方，实现精准变量施肥。

碳氮比调控技术：通过添加秸秆（C/N=60-80）或豆粕（C/N=10-15）调节土壤 C/N 比。大蒜适宜 C/N 比为 20-25，过高时补氮，过低时增施碳源，维持微生物群落平衡。

全周期营养诊断图谱：建立从苗期到收获期的大蒜营养诊断图谱：包括叶片 SPAD 值（苗期 35-45，旺盛期 45-55）、茎粗（抽薹期 6-8mm）、鳞茎比重（成熟期≥1.05）等指标，实现可视化精准管理。

光肥耦合增效技术：在大蒜鳞茎膨大期，结合叶面喷施 0.1%螯合铁+0.05%硫酸锰，同时延长光照时间至 14 小时/天（补光灯 200μmol·m^{-2}·s^{-1}），可显著提高光合效率，促进干物质积累，增产 15-20%。

纳米肥料应用技术：采用纳米级氧化锌（粒径 20-50nm）和纳米碳（粒径 10nm）配制的专用肥，通过叶面喷施或滴灌施用，养分利用率比常规肥料提高 40%，每亩减施 30%仍能保证产量。

根系靶向施肥方法：在距植株基部 5-8cm 处开 10cm 深沟，将腐殖酸包裹的控释肥（N-P_2O_5-K_2O=18-12-20）按 15kg/亩施入，覆土后滴灌。该方法使肥料集中在活跃根区，利用率达 75%以上。

逆境恢复营养方案：遭遇冻害后，立即喷施 0.3%磷酸二氢钾+0.1%芸苔素内酯（0.01mg/L），配合地温提升（覆盖透明地膜），3 天后追施硝态氮肥 5kg/亩，可促进新叶萌发，恢复生长势。

生育期动态营养模型：基于积温（≥5℃有效积温 1400-1600℃）和叶龄指数（收获时达 12-14 片叶），建立氮磷钾动态需求模型。如出苗至越冬期需 N30%、P20%、K10%；返青至抽薹期需 N40%、P30%、K30%。

功能性微生物组合：将固氮菌（Azotobacter）、解磷菌（Pseudomonas）和根际促生菌（PGPR）按 3:2:1 比例复合，每亩基施 2kg（含活菌数≥10 亿/g），可替代 25%氮肥和 40%磷肥，连作障碍减轻 50%。

元素拮抗规避技术：针对钙-硼拮抗，采用分次施用：基施钙肥（硝酸钙 15kg），返青期喷硼（0.2%四水八硼酸钠）；对钾-镁拮抗，保持土壤 K/Mg 比在 3-5:1，超出时补施硫酸镁。

智能缓释肥包膜技术：采用温敏型聚合物包膜尿素，膜厚 50-80μm，在 10-25℃区间释放速率与大蒜需求同步。全生育期基施一次（40kg/亩），可满足 80%氮素需求，减少追肥 2 次。

同位素示踪施肥法：用^{15}N 标记尿素研究氮素去向，发现大蒜对基肥氮利用率仅 35%，改进为"20%基施+50%返青期追施+30%膨大期追施"后，利用率提升至 55%，氨挥发减少 40%。

光谱诊断精准追肥：利用多光谱无人机（波段 530nm、670nm、720nm）获取 NDVI 值，当指数低于 0.65 时启动变量追肥，通过处方图指导施肥机按需施氮，误差控制在 5%以内。

生物炭基肥改良技术：将稻壳生物炭（pH8.5，比表面积 300m^2/g）与有机肥按 1:3 混合，每亩施用 500kg，可使砂质土壤保水率提高 25%，铵态氮流失减少 60%，特别适合干旱区大蒜栽培。

硫素形态调控策略：在冷凉地区选用硫代硫酸铵（含 S26%），比硫酸盐更易吸收；在温暖地区用元素硫（S90%），通过微生物氧化缓慢释放。根据土壤温度选择硫源，利用率差异达 30%。

根冠协调施肥法：苗期注重根系发育，每亩用腐殖酸 5kg+磷酸一铵 10kg 冲施；旺盛期促进冠层扩展，追施硝酸钾 15kg；成熟期控制地上生长，叶面喷施 3%草木灰浸提液控旺。

等离子体处理种肥技术：播种前用低温等离子体（功率 300W，处理 5min）激活种子，配合带电晕处理的复合肥（N-P_2O_5-K_2O=15-30-15），发芽率提高 20%，苗期干重增加 35%。

微生物-矿物复合技术：将蒙脱石（吸附载体）、磷矿粉（P_2O_5 28%）与解磷菌（Bacillusmegaterium）复合，制成缓释磷肥。比过磷酸钙利用率提高 50%，且持续释放期达 120 天。

生理障碍矫正方案：针对"黄叶早衰"现象（多因缺钾和早衰素积累），喷施 0.5%硫酸钾+0.1%水杨酸；对"紫斑叶"（缺磷和花青素累积），土施磷酸二铵 10kg 并提升地温 2-3℃。
空间分层施肥系统：0-10cm 土层施速效肥（占 30%），10-20cm 施缓释肥（50%），20-30cm 施有机肥（20%），形成立体供肥网络，满足不同生育期根系的空间需求。
量子点标记追踪技术;采用 CdSe/ZnS 量子点标记肥料颗粒，通过荧光显微镜观察养分在根系的运输路径，优化出最佳施肥位点为距主根 5cm、深 10-15cm 处，吸收效率最高。
仿生叶片营养技术:模拟健康大蒜叶片元素组成（N3.8%、K2.9%、Ca1.2%等），配制专用平衡肥，使植株体内元素比例趋近理想值，品质指标（大蒜素、可溶性糖）提升 25%。
区块链施肥追溯系统:从施肥记录、检测数据到品质认证全程上链，通过智能合约自动触发追肥决策。如当区块链接收到的土壤传感器数据显示钾含量<120mg/kg 时，自动启动钾肥追施程序。
大蒜在生长过程中对氮、磷、钾的需求较为均衡，其中氮肥对促进叶片生长尤为重要，磷肥有助于根系发展和鳞茎的形成，钾肥则能增强植株的抗病能力。
基肥施用时，应以充分腐熟的有机肥为主，如农家肥，每亩施用 3000-4000 公斤，为大蒜生长提供充足的养分基础。
大蒜追肥一般分两次进行，第一次在幼苗期，以氮肥为主，促进植株早发快长；第二次在鳞茎膨大期，以磷钾肥为主，促进鳞茎发育。
大蒜对硼、锌等微量元素较为敏感，适量施用硼锌肥可以改善鳞茎品质，提高产量，一般在播种前或生长初期进行施用。
施肥时应注意深施，将肥料施于土壤深处，有利于根系向下生长，同时减少肥料流失。
施肥后要及时浇水，帮助肥料溶解，提高肥料利用率，同时保持土壤湿润，有利于大蒜吸收养分。
大蒜生长期间，土壤湿度应保持在 60%-70%，过高会导致根系病害，过低则会影响植株生长。
土壤酸碱度对大蒜生长影响较大，适宜的 pH 值为 6.0-7.0，过高或过低均不利于大蒜的生长和鳞茎品质。
大蒜施肥应遵循“少量多次”的原则，避免一次性施肥过多导致烧根，影响植株生长。
在大蒜生长中后期，可适量喷施叶面肥，如磷酸二氢钾，以补充植株所需的磷钾元素，促进鳞茎发育。
大蒜收获前 20 天应停止施肥，以免肥料残留影响鳞茎品质，同时让植株有时间将吸收的养分转化为鳞茎产量。
施肥时应注意天气变化，避免在高温或阴雨天气进行，以免造成肥料损失和环境污染。
土壤缺钙时，可通过施用石灰来调节土壤 pH 值，同时补充钙元素，有助于提高大蒜的抗病能力。
大蒜生长初期，应注重氮肥的施用，以促进叶片的快速生长，为后续鳞茎的发育打下良好的营养基础。
磷肥在大蒜生长中的作用是促进根系发育和增强植株的抗寒能力，尤其在寒冷地区更为重要。
钾肥能提高大蒜的抗病能力和逆境适应力，同时有助于提高鳞茎的干物质含量。
大蒜对镁元素的需求不可忽视，缺镁会导致叶片黄化，影响光合作用，适当施用镁肥可以改善这一状况。
大蒜施肥时应注意肥料与土壤的混合均匀，以免造成局部浓度过高，损伤根系。
大蒜生长期间，应定期进行土壤测试，了解土壤养分状况，以便及时调整施肥计划。
大蒜在鳞茎膨大期对水肥需求量大，应保证充足的水分供应，以促进鳞茎的充分发育。
大蒜施肥时，可以采用条施或穴施的方法，使肥料更接近根系，提高肥料利用率。

有机肥的施用不仅能提供养分，还能改善土壤结构，增强土壤的保水和供肥能力。
大蒜在生长过程中，若出现叶片发黄、生长缓慢等症状，可能是缺肥的表现，应及时补充相应肥料。
大蒜营养管理的关键在于平衡施肥，既要满足植株生长需求，又要避免过量施肥造成资源浪费和环境污染。
大蒜在生长初期对氮肥的需求尤为突出，因为氮是构成蛋白质和叶绿素的重要成分，充足的氮肥能促进叶片的快速生长，增加光合作用面积，为后续鳞茎的发育积累养分，此时应以施用尿素或硫酸铵等氮肥为主，每亩施用量控制在 15-20 公斤。
磷肥在大蒜生长中起到关键作用，它不仅促进根系的发展，还能增强植株的抗寒和抗旱能力，特别是在低温季节，磷肥的施用尤为关键，可以选择过磷酸钙或磷酸二铵，基肥时每亩施用 40-50 公斤。
钾肥对提高大蒜的抗病能力和逆境适应力至关重要，同时能够促进鳞茎中干物质的积累，提高鳞茎的品质，通常在基肥和追肥时施用硫酸钾，每亩施用量约为 20-30 公斤。
大蒜对硼、锌等微量元素的需求虽不高，但缺乏时会导致生长发育受阻，因此，适时施用微量元素肥料，如硼砂或硫酸锌，可以喷施或随水冲施，用量一般为每亩 0.5-1 公斤。
大蒜施肥时应注意肥料的均匀分布，避免局部施肥过浓造成烧根现象，可以通过机械施肥或人工撒施后翻耕的方式，确保肥料与土壤充分混合。
大蒜在鳞茎膨大期对磷钾肥的需求增加，此时应减少氮肥的施用，增加磷钾肥的比例，以促进鳞茎的充实和成熟，可以追施磷酸二铵和硫酸钾，每亩追施 15-20 公斤。
大蒜施肥时，应选择在晴朗天气的早晨或傍晚进行，此时气温较低，施肥后土壤中的肥料不易挥发，有利于植株的吸收。
大蒜对钙的需求较高，钙能增强鳞茎的耐储性和减少病害的发生，可以通过施用石灰或钙镁磷肥来补充土壤中的钙含量，每亩施用 50-100 公斤。
大蒜生长过程中，保持土壤疏松透气是关键，定期进行中耕松土，不仅有利于根系生长，还能促进土壤中有益微生物的活动，增强土壤的供肥能力。
大蒜施肥后，应及时浇水，以促进肥料的溶解和根系对养分的吸收，但浇水不宜过多，以免造成土壤湿度过大，影响鳞茎的正常生长。
大蒜在生长初期，可以施用充分腐熟的有机肥，如牛粪、羊粪等，这些有机肥不仅能提供全面的营养，还能改善土壤结构，每亩施用 3000-4000 公斤。.
大蒜施肥时要考虑到土壤的肥力水平和肥料利用率，避免过量施用化肥造成资源浪费和环境污染，应实行测土配方施肥。
大蒜在生长后期，应严格控制氮肥的施用，以防植株生长过旺，导致鳞茎成熟延迟，影响产量和品质。
大蒜对硫肥的需求也不可忽视，硫是合成氨基酸和蛋白质的必需元素，适量施用硫磺或硫酸盐肥料，可以促进大蒜的生长。
大蒜生长期间，可以通过叶面喷施的方式补充微量元素，如硼、锌、铁等，这种施肥方式快速直接，易于植株吸收。
大蒜施肥时，应根据不同地块的肥力状况和植株生长情况，实行差异化施肥，以达到最佳的经济效益。
大蒜在生长过程中，要密切关注土壤湿度，通过合理的灌溉管理，保持土壤湿润但不积水，以利于根系的健康生长。
大蒜施肥后，要定期观察植株的生长状况，如叶色、长势等，以便及时调整施肥策略，确保植株营养均衡。
大蒜在鳞茎膨大期，可以适量施用磷酸二铵，这种肥料既能提供磷肥又能提供氮肥，有助于

鳞茎的发育和产量的提高。
大蒜施肥时，要注意避免肥料直接撒在叶片上，以免造成肥害，影响植株的正常生长。正确的施肥方法是将肥料施于植株根部附近，然后覆土。
大蒜在播种前，应进行土壤检测，了解土壤的 pH 值、有机质含量、氮磷钾等养分的状况，以便制定合理的施肥计划。土壤 pH 值以 6.0-7.0 为宜，过于酸性或碱性都需要通过施用石灰或硫磺进行调整。
大蒜基肥的施用应以有机肥为主，如充分腐熟的鸡粪、牛粪等，每亩施用 3000-5000 公斤，不仅可以提供丰富的养分，还能改善土壤结构，增强土壤的保水和供肥能力。
大蒜追肥一般分为两次，第一次在植株长出 3-4 片叶时进行，以氮肥为主，可施用尿素或硫酸铵，每亩施用 10-15 公斤；第二次追肥在鳞茎开始膨大时进行，以磷钾肥为主。
大蒜对钙的需求较高，钙肥能促进大蒜根系的发展，提高鳞茎的品质，可以通过施用石灰或钙镁磷肥来补充钙元素。
大蒜在生长过程中，若出现叶片黄化、生长缓慢等症状，可能是缺铁或缺锰的表现，应及时叶面喷施硫酸亚铁或硫酸锰等微量元素肥料。
大蒜施肥时应注意施肥时间，一般选择在晴天的早晨或傍晚进行，避免高温时段施肥，以免造成肥害。
大蒜在鳞茎膨大期，对水分和养分的需求达到高峰，此时应保证充足的水分供应，并结合追肥，以满足植株的生长需求。
大蒜施肥后，要及时进行灌溉，以促进肥料溶解和根系吸收，提高肥料的利用率。
大蒜在生长后期，应减少氮肥的施用，防止植株徒长，影响鳞茎的成熟和品质。
大蒜对硼的需求量虽不大，但硼元素的缺乏会导致花芽分化受阻，影响产量，可在生长初期叶面喷施硼砂溶液。
大蒜施肥时，应避免肥料直接接触蒜种，以免造成烧种现象，影响出苗率。
大蒜在收获前 20 天左右，应停止施肥，以免肥料残留影响鳞茎的储藏品质。
大蒜对锌的需求虽不高，但锌元素的充足能提高植株的抗病能力，可通过叶面喷施硫酸锌来补充。
大蒜在施肥过程中，要密切关注植株的生长状况，及时调整施肥策略，确保植株生长健康。
大蒜在鳞茎膨大期，可以适量施用生物有机肥，如腐殖酸肥料，以改善土壤环境，促进鳞茎的发育。
大蒜施肥时，要考虑到不同肥料之间的相互作用，如氮肥过多会抑制钾的吸收，因此要平衡施肥。
大蒜营养管理的总体原则是“前期促生长，中期促鳞茎，后期控旺长”，通过科学施肥和田间管理，实现大蒜的高产优质。
大蒜在生长初期，除了注重氮肥的施用外，还应适当补充一些生物菌肥，如根瘤菌剂，这些菌剂能够固定空气中的氮素，为大蒜提供额外的氮源，同时改善土壤微生物环境。
大蒜对硼肥的需求虽然不大，但硼元素的充足能显著提高大蒜的结蒜率和鳞茎品质，因此，在生长前期和中期各喷施一次硼酸溶液是很有必要的。
大蒜施肥时应采用深施的方法，将肥料施于土壤深处，这样既可以减少肥料挥发损失，又能促进根系向下生长，增强吸水吸肥能力。
大蒜在生长过程中，若遇到连续阴雨天气，应及时排涝，避免土壤湿度过大导致根系缺氧和病害的发生。
大蒜在鳞茎膨大期，可以结合叶面喷施磷酸二氢钾溶液，这种做法既能补充磷钾营养，又能增强植株的抗逆性。
大蒜在生长期间，要注意观察植株的叶色变化，叶色偏黄可能是缺氮，叶尖枯黄可能是缺钾，

及时调整施肥策略。
大蒜施肥后，要及时浇水，以促进肥料溶解和根系吸收，但浇水不宜过多，以免造成土壤湿度过大。
大蒜对硫元素的需求不可忽视，硫能促进大蒜对氮磷钾的吸收利用，可通过施用硫酸钾或硫磺粉来补充硫元素。
大蒜在生长过程中，要定期进行土壤测试，根据土壤养分状况和植株生长需求，制定个性化的施肥方案。
大蒜施肥时，可以采用穴施或条施的方法，将肥料集中施在植株根部附近，提高肥料利用率。
大蒜在生长后期，若出现旺长现象，可以适当控制水分和氮肥，以防止植株徒长，影响鳞茎的形成。
大蒜在收获前，应避免使用氮肥，以免造成鳞茎含水量过高，不利于储藏和运输。
大蒜在施肥过程中，要注意不同肥料之间的搭配，如氮肥与磷钾肥的配比，以确保养分的均衡供应。
大蒜施肥时，可以结合使用有机肥和化肥，有机肥提供长效养分，化肥提供速效养分，两者结合使用效果更佳。
大蒜营养管理的目标是实现植株生长与鳞茎发育的平衡，通过合理的施肥和田间管理，达到优质、高产、高效的生产目的。
光合碳流导向施肥技术:在大蒜鳞茎膨大关键期，通过喷施 0.1%蔗糖+0.2%磷酸二氢钾混合液，引导光合产物向鳞茎运输。配合午后摘除底层老叶，可使同化物分配效率提高 25%，鳞茎饱满度显著改善。
根际 pH 微域调控方法:针对碱性土壤（pH>8.0），在根际 5cm 范围内施用硫磺粉（3kg/亩）与腐殖酸（5kg/亩）混合物，通过微生物酸化作用，创造 pH6.5-7.0 的微环境，提高磷铁锌等元素有效性达 40%。
气孔导度响应型叶面肥:利用热成像仪监测叶片温度，当温差（叶-气）>3℃时，喷施添加表面活性剂（0.05%Tween20）的钾钙肥。可降低气孔阻力，使养分吸收窗口期延长 2-3 小时
生物膜阻控施肥技术:在砂质土壤中，施用含胶质芽孢杆菌（Bacillusmucilaginosus）的生物膜制剂，其在根表形成选择性屏障，减少氮钾淋失 50%以上，同时促进难溶性磷的活化。
纳米硅钾复合肥应用:将纳米二氧化硅（10-50nm）与硫酸钾复合，制成含 Si5%、K_2O30%的专用肥。基施 20kg/亩可增强细胞壁强度，使叶斑病发生率降低 60%，抗倒伏能力提升。
代谢抑制剂协同施肥：在追施尿素时，配合使用硝化抑制剂 DCD（双氰胺）1kg/亩，将铵态氮向硝态氮转化时间延长至 20-25 天，使氮肥利用率从 35%提升至 55%，特别适合多雨地区。
根系分泌物引导施肥：分析大蒜根系分泌的有机酸（以柠檬酸为主），在分泌高峰前 3 天追施磷肥。利用其天然螯合作用，使难溶性磷活化效率提高 70%，减少磷肥用量 30%。
电位调控营养液系统：通过调节营养液氧化还原电位（ORP+150~+200mV），维持铁锰等元素的可溶性。配合自动 pH 调控（5.8-6.2），使水培大蒜微量元素吸收稳定性提高 40%。
叶面微环境优化技术：在露水未干时喷施添加甘油（2%）的叶面肥，利用其保湿性延长叶片湿润时间，使钙硼等移动性差的元素吸收量增加 50%，尤其预防干尖病效果显著。
生物炭基缓释肥配方;将稻壳生物炭（孔隙率 80%）与尿素按 1:1 混合热压成型，制成缓释氮肥。在土壤中尿素释放周期延长至 60 天，氨挥发损失减少 70%，适合一次性基施。
光谱诊断变量追肥:利用多光谱相机获取冠层 NDRE（红边归一化指数），当指数<0.55 时，通过变量施肥机按 0.5kg/亩梯度增施氮肥，实现单株精准补给，均匀度达 90%以上。
磁化水肥耦合技术:将灌溉水以 0.3T 磁场强度处理，溶解肥料后滴灌。水分子团簇变小，携肥能力增强，使土壤深层（20-30cm）养分分布均匀度提高 35%，促进深层根系发育。

内生菌接种增效技术:接种大蒜专用内生枯草芽孢杆菌（BacillussubtilisGB03），其分泌的 ACC 脱氨酶可降低逆境乙烯伤害，使化肥利用率提升 20%，连作障碍减轻 40%。
冠层温光调控施肥:在设施栽培中，当冠层温度持续>25℃时，改用硝态氮肥替代铵态氮肥，配合增加 10%钾肥用量。可缓解高温胁迫，维持正常代谢，产量波动减少 30%。
土壤生物电活性提升:埋设石墨烯电极（间距 50cm，电压 0.5V），刺激土壤微生物活性，使有机质分解速率提高 40%。配合秸秆还田，可替代 15%化肥用量，同时改善土壤结构。
叶片角质层穿透技术:在叶面肥中添加木质素降解酶（0.001%），短暂打开角质层通道 2-3 小时。使大分子物质（如腐殖酸）吸收率从 5%提升至 25%，显著增强抗逆性。
根系分区交替灌溉施肥:将根区分为湿润区和干燥区交替灌溉，湿润区施氮肥，干燥区施磷钾肥。利用根系趋水性诱导养分高效吸收，节水 30%的同时产量保持稳定。
纳米氧化还原调节剂:施用 CeO_2纳米颗粒（10mg/kg 土），其可变价特性缓冲土壤氧化还原电位，使铁锰有效性提高 50%，尤其解决石灰性土壤缺素问题效果突出。
大蒜生长初期对氮肥需求较高，可在播种后 15-20 天，每亩追施尿素 10-15 公斤，促进蒜苗快速生长，长出健壮叶片，为后期生长奠定基础，实际操作时要注意尿素均匀撒施，避免烧苗。
大蒜在鳞芽分化期，对磷元素需求增大，此时可每亩施用过磷酸钙 20-25 公斤，以促进大蒜花芽和鳞芽分化，保证蒜薹和蒜头的正常发育，过磷酸钙最好开沟条施，提高肥料利用率。
蒜薹伸长期是大蒜生长的关键时期，对氮、钾的需求量大幅增加。一般每亩追施高氮高钾复合肥 20-25 公斤，同时配合浇水，促进蒜薹快速伸长，提高蒜薹的产量和品质。
蒜头膨大期，钾元素对大蒜至关重要，可每亩追施硫酸钾 10-15 公斤，促进蒜头膨大，增加蒜头重量。此阶段施肥不能偏施氮肥，否则易导致蒜头松散、不耐储存。
基肥是大蒜生长的重要养分来源，在播种前，每亩应施入充分腐熟的农家肥 3000-5000 公斤，配施三元复合肥 30-50 公斤，改善土壤结构，持续为大蒜生长提供养分，农家肥一定要腐熟彻底，防止烧根。
大蒜苗期若基肥不足，可在 2-3 叶期进行一次追肥，以氮肥为主，配合少量磷肥，每亩施用尿素 5-8 公斤、过磷酸钙 5-10 公斤，促使幼苗茁壮成长，增强抗寒能力，安全越冬。
微量元素对大蒜生长也不可或缺。例如，大蒜对锌、硼元素敏感，在大蒜生长中期，可叶面喷施 0.2%-0.3%的硫酸锌和硼砂溶液 2-3 次，间隔 7-10 天，促进大蒜生长，提高蒜头品质。
地膜覆盖栽培的大蒜，施肥方式与露地有所不同。基肥要适当增加，因为地膜覆盖后追肥不便。在施足基肥基础上，可在苗期和蒜薹伸长期随水冲施速效肥，满足大蒜生长需求。
不同土壤肥力条件下大蒜施肥有差异。土壤肥力高的地块，基肥用量可适当减少，追肥次数和用量也相应降低;而土壤肥力低的地块,则要增加施肥量和施肥次数,保证大蒜养分供应。
大蒜根系浅，吸收能力较弱，施肥时应遵循“少量多次”原则。每次施肥量不宜过多，以免肥料浪费和造成土壤污染，同时要结合浇水，促进肥料溶解吸收。
大蒜返青期是生长的重要转折点，此时应及时追肥，每亩追施尿素 10-15 公斤，促进蒜苗快速返青生长，增强光合作用，为后续生长积累养分，追肥时间要把握准确，过早过晚都不利于大蒜生长。
蒜薹采收后，大蒜进入蒜头快速膨大阶段，此时根系吸收能力减弱，可通过叶面喷施磷酸二氢钾溶液，浓度为 0.2%-0.3%，每隔 5-7 天喷一次，共喷 2-3 次，补充养分，防止叶片早衰。
.种植大蒜时，可将有机肥和化肥配合施用。有机肥能改善土壤理化性质，提高土壤保肥保水能力，化肥则能快速补充大蒜所需养分，二者结合可提高大蒜产量和品质。
在酸性土壤中种植大蒜，容易出现铁、铝等元素过量而影响大蒜生长，可适当施用石灰调节土壤酸碱度，同时补充钙元素，一般每亩施用石灰 50-100 公斤，改善土壤环境，促进大蒜健康生长。

大蒜生长后期，根系老化，吸收养分能力下降，除了叶面追肥外，还可通过滴灌或冲施氨基酸水溶肥，补充氨基酸、腐殖酸等营养物质，增强大蒜抗逆性，提高蒜头产量。
连作的大蒜田，土壤中病原菌和有害微生物增多，养分失衡。施肥时应增施生物菌肥，每亩施用 2-3 公斤，改善土壤微生物群落，抑制有害菌生长，同时补充土壤中缺乏的养分。
大蒜施肥还要考虑气候因素。在干旱年份，施肥应结合浇水进行，或者采用水肥一体化技术，保证肥料能被大蒜有效吸收；在雨水较多的年份，要注意及时排水，避免肥料流失。
为提高大蒜的商品性，在施肥过程中要控制重金属和有害物质的含量。避免使用含有重金属的肥料，选择正规厂家生产的合格肥料产品，确保大蒜质量安全。
大蒜在不同生长阶段对养分的吸收比例不同。从幼苗期到蒜头膨大期，氮、磷、钾的吸收比例大致为 3:1:3，根据这个比例合理调配肥料，满足大蒜生长需求。
精准施肥是提高大蒜产量和效益的关键。可通过土壤检测，了解土壤养分状况，根据大蒜的需肥规律制定施肥方案，做到缺什么补什么，既节省肥料成本，又能提高大蒜产量和品质。
秋播大蒜越冬前，需施一次越冬肥，以有机肥为主，比如每亩施入 2000 公斤左右的腐熟厩肥。不仅能为大蒜缓慢提供养分，还能起到保温护根作用，帮助大蒜安全度过寒冬，为开春返青生长储备能量。
大蒜抽薹前，可追施一次速效氮肥，搭配适量钾肥，比如每亩用 10 公斤尿素和 5 公斤硫酸钾。有助于蒜薹快速抽出，提高蒜薹的粗细和长度，从而提升蒜薹产量，满足市场对优质蒜薹的需求。
大蒜对硫元素有特殊需求，硫元素可提高大蒜的风味和品质。在基肥中可加入含硫的肥料，如硫酸钾型复合肥，或者每亩施用 15-20 公斤的石膏粉，改善大蒜品质，提升市场竞争力。
基肥中的农家肥一定要充分腐熟，未腐熟的农家肥在土壤中发酵会产生热量，容易烧根，影响大蒜出苗和幼苗生长。可提前 2-3 个月进行堆沤，保证腐熟度，为大蒜生长提供安全有效的养分。
大蒜苗期若发现叶片发黄、生长缓慢，可能是缺乏养分，可进行叶面喷施肥料。如喷施 0.5%的尿素溶液和 0.2%的磷酸二氢钾溶液混合液，每隔 5-7 天喷一次，连续 2-3 次，快速补充养分，使蒜苗恢复生机。
蒜薹收获后，蒜头进入迅速膨大期，此时要保证养分充足供应。可随水冲施高钾水溶肥，每亩用量 5-8 公斤，促进蒜头快速膨大，增加单头重量，提高大蒜整体产量。
在大蒜种植过程中，可采用分层施肥的方法。播种时将基肥的三分之二均匀撒施在土壤表面，然后翻耕入土；剩余三分之一基肥在播种沟内施入，使大蒜在不同土层都能吸收到养分。
大蒜生长过程中，可定期进行土壤养分检测。一般每隔 1-2 年检测一次，根据检测结果调整施肥方案，保证土壤养分平衡，满足大蒜生长对各种养分的需求。
大蒜种植密度不同，施肥量也应有所调整。密度大的地块，大蒜个体生长空间有限，需适当增加施肥量，以保证每株大蒜都能获得足够养分；密度小的地块则可适当减少施肥量。
大蒜的根际施肥可结合中耕进行，中耕能疏松土壤，增强土壤透气性，同时将肥料翻入土中，使肥料与根系更好地接触，促进根系对养分的吸收，一般在每次追肥后进行中耕。
对于砂质土壤，由于保肥保水能力差，大蒜施肥应少量多次，避免肥料随水流失。每次施肥量可适当减少，但增加施肥次数，确保大蒜生长期间养分不断档。
大蒜生长后期，根系活力下降，除了叶面追肥外，还可进行灌根施肥。用氨基酸类或腐植酸类水溶肥稀释后进行灌根，促进根系吸收，增强大蒜抗逆性，延长叶片功能期。
大蒜种植前，可将肥料与土壤充分混合。比如将复合肥和土壤按一定比例搅拌均匀后，再进行播种，使肥料在土壤中分布均匀，有利于大蒜根系均匀吸收养分。
春季气温回升后，大蒜进入快速生长阶段，此时应及时追施返青肥。以氮肥为主，搭配适量磷肥，每亩施用尿素 15 公斤左右、过磷酸钙 10 公斤左右，促进蒜苗快速生长，增加叶片数

量和面积。
大蒜在生长过程中，如果出现缺钙症状，如叶片边缘发黄、干枯，可叶面喷施 0.3%-0.5%的氯化钙溶液，每隔 7-10 天喷一次，连续 2-3 次，补充钙元素，保证大蒜正常生长。
种植大蒜时，可利用微生物菌剂来提高肥料利用率。在基肥或追肥中添加微生物菌剂，如枯草芽孢杆菌、解磷解钾菌等，促进土壤中养分的转化和释放，减少肥料用量。
大蒜生长中期，可适当追施一些有机肥液，如腐熟的饼肥水。将饼肥浸泡腐熟后，稀释 10-15 倍，每亩浇施 500-800 公斤，既能补充养分，又能改善土壤环境，促进根系生长。
夏季高温多雨，大蒜种植后容易出现肥料流失的情况。在施肥后要及时进行覆土，减少肥料暴露，同时注意排水，避免积水导致肥料淋失，降低肥效。
大蒜对镁元素也有一定需求，镁元素参与光合作用。当大蒜出现缺镁症状，如叶片失绿发黄时，可叶面喷施 0.2%-0.3%的硫酸镁溶液，2-3 次后可有效缓解缺镁症状，提高大蒜光合效率。
大蒜收获前 20 天左右，应停止土壤追肥，避免肥料残留影响大蒜品质。但可进行叶面喷施磷酸二氢钾，增强大蒜的抗逆性，提高大蒜的耐储存性。
我国大蒜分布地域广阔，种植区从北到南跨越了寒温带、中温带、暖温带、亚热带和热带五个气候带，在多年的栽培过程中形成了许多各具特色的大蒜品种。
我国大蒜分布地域广阔，种植区从北到南跨越了寒温带、中温带、暖温带、亚热带和热带五个气候带，在多年的栽培过程中形成了许多各具特色的大蒜品种。
大蒜是百合科葱属大蒜种植物，根据大蒜的鳞茎（蒜头)、蒜瓣、植株、生育期、蒜薹、生态和生理特性等性状进行分析，将大蒜分为双层蒜衣和单层蒜衣两个变种，每个变种又各分成三个品种群。
双层蒜衣变种包括抽薹大蒜品种群、不完全抽薹大蒜品种群和春蒜品种群。
单层蒜衣变种包括长叶大蒜品种群、短叶大蒜品种群和多层蒜瓣大蒜品种群。
生态分类法是根据大蒜生长发育对生态环境条件的要求及其生态适应性进行品种分类的。根据生态分类可将大蒜分为低温反应敏感型、低温反应中间型和低温反应迟钝型三个生态型。
根据大蒜鳞茎外皮色泽分类可将大蒜分为白皮蒜和紫（红）皮蒜两种类型。
白皮蒜类型有苍山大蒜、大马牙蒜、狗牙蒜、无薹大蒜、杭州白皮蒜等。
紫（红）皮蒜类型代表品种有蔡家坡红皮蒜、阿城大蒜、定县紫皮蒜、嘉祥大蒜等。
根据构成鳞茎的蒜瓣大小和蒜瓣数可将大蒜分为大瓣蒜和小瓣蒜两种类型。
大瓣蒜蒜瓣数较少，每头为 4～8 瓣，蒜瓣个体肥大，而且比较均匀。味香辛辣，外皮容易剥落，蒜头产量较高，蒜薹粗而长，以生产蒜薹和蒜头为主。其代表品种有苍山大蒜、开原大蒜、阿城大蒜等。
小瓣蒜又叫狗牙蒜。蒜瓣狭长，大小不均匀，瓣数较多，一般 10 个以上，多者可达 20 多瓣。蒜皮薄，不易剥落，辣味较淡，产量偏低，适于蒜黄和青蒜栽培。其代表品种有白皮马牙蒜、拉萨白皮蒜等。
根据蒜薹的有无或发达程度可将大蒜分为有薹蒜、无薹蒜和半抽薹蒜三种类型
有薹蒜是可以正常抽生蒜薹的大蒜品种，既可收获蒜薹，又可收获蒜头，是我国各地普遍栽培的大蒜品种。
无薹蒜有两种情况，一种是大蒜生长点不分化花芽，因而不能形成蒜薹；另一种是大蒜生长点分化花芽，但花芽发育不良形成弱小的花薹，包藏在基部叶鞘中，不能生长成蒜薹。
半抽薹蒜是由于花芽分化形成后，在发育过程中延伸生长缓慢，形成粗短的蒜薹，而蒜薹顶部的气生鳞茎膨大，将叶鞘膨胀，从裂口处伸出，形成“腰蒜”。
根据叶片的质地和空间姿态可将大蒜分为软叶蒜和硬叶蒜两种类型。
软叶蒜一般叶片质地较软，叶片较宽而平展，生长期叶片下垂。如四川新都软叶子

硬叶蒜叶片质地较硬，叶片较窄而呈槽形，生长期叶片挺直向上扬。如成都硬叶子。这种分类有助于了解大蒜品种的生长习性，制定合理的密植措施。
根据蒜秸质地可将大蒜分为硬秸、软秸两大类型。
春性蒜　一般耐寒性较差，春播或近冬播种，一般不抽薹蒜瓣较小。
冬性蒜　一般耐寒性较强，秋播，可抽薹，蒜瓣较大而少。
根据生育期长短和熟性可将大蒜分为极早熟、早熟、中熟和晚熟四大类型。
秋播蒜的中熟类型又分为中早熟、中熟和中晚熟三类。
根据栽培用途可将大蒜分为蒜头用型、薹头兼用型、早薹型、苗用型和加工用型五种类型。
苍山大蒜：山东省苍山县地方品种,为山东省传统名特蔬菜之一。其特点是蒜头大,色洁白,瓣少而大,味香浓,蒜汁黏稠,蒜薹粗而长,蒜头和蒜薹产量高,适应性强,在国内外颇负盛名。
鲁农大蒜：山东农业大学从苏联红皮大蒜中定向选育而成。植株长势强,株高 80 厘米左右,株形开张。全株叶片数 13 片,最大叶长 73 厘米,最大叶宽 4 厘米。蒜头扁圆形,横径 5 厘米左右,形状整齐,外皮灰白色带紫色条斑,单头重 50 克左右。每个蒜头有蒜瓣 10~13 个,分两层排列,外层 6~7 瓣,内层 4~6 瓣,外层蒜瓣肥大,内层为中小蒜瓣。蒜衣基部淡红色,平均单瓣重 4.5 克。抽薹率 80%以上,蒜薹长 60 厘米左右,粗 0.7 厘米左右。休眠期短,播种后出苗快,苗期生长快,可利用中、小瓣进行密植作蒜苗栽培。
大蒜属百合科葱属 1~2 年生草本植物。通常不结种子,用蒜瓣进行无性繁殖。一株完整的大蒜植株包括:根、鳞茎、叶鞘、叶身、花茎、总苞及气生鳞茎。
大蒜的根是从蒜瓣基部的茎盘上发生的,为弦线状须根,称为不定根,没有主根。蒜瓣背部(外侧)的茎盘边缘发根较多,腹部(内侧)发根较少。主要根系分布在 5~25 厘米深的土层中,横向分布范围约 30 厘米,属浅根性蔬菜。须根上的根毛少,吸水力弱,所以喜湿、喜肥,不耐旱。
通常所称的蒜头,植物学名词是鳞茎,构成鳞茎的各个蒜瓣,植物学名词叫鳞芽。蒜头和蒜瓣的基部都有一个扁平的盘状致密组织,称茎盘。它与植物正常的茎不同,属于茎的变态,又称变态茎。蒜头成熟以后,茎盘木质化,有保护蒜瓣、减少水分散失的作用,所以大蒜贮藏时要用完整的蒜头。
鳞芽是由大蒜植株叶片叶腋处的侧芽发育而成,所以又称“鳞腋芽”。鳞芽由 2~3 层鳞片和 1 个幼芽构成。外面 1~2 层鳞片起保护作用,称保护鳞片或保护叶。最内一层是贮藏养分的部分,称贮藏鳞片或贮藏叶。
大蒜通常通过无性繁殖方式进行，即通过鳞茎的分株繁殖。将鳞茎分成多个带有部分鳞片的蒜瓣，每个蒜瓣都能长成新的植株。
大蒜的地下部分为鳞茎，由多个肥厚的鳞片组成，外皮白色或紫色，是主要的食用部分。
大蒜的叶片包括叶鞘和叶身两部分。叶鞘呈圆筒形,着生在茎盘上。每一片叶均由先发生的前一片叶的出叶口伸出,许多层叶鞘套在一起,形成直立的圆柱形茎秆,由于它不是真正的茎,故称“假茎”。叶与叶之间的叶鞘长度随叶位的升高而增加。一般在花茎伸出最后一片叶的叶鞘口以后,叶鞘停止生长。
大蒜鳞茎盘顶部的生长锥分化为花芽后,逐步发育抽生蒜薹。蒜薹的下部为花茎,顶端着生花苞称总苞。总苞成熟后开裂,可以看到许多小的鳞茎,称气生鳞茎、空中鳞茎,俗称蒜珠或天蒜。
大蒜的叶片互生,对称排列。叶片的排列方向与蒜瓣的背腹连线垂直。所以,播种时应将蒜瓣的背腹连线与播种行的方向相平行,则出苗后叶片的排列方向就和播种行的方向垂直,这样一来,叶片与叶片之间的遮荫减少,可以接受更多的阳光,增强叶片的光合作用。
大蒜叶片的颜色多为绿色至深绿色。叶面一般有白粉。叶片绿色的深浅,叶片的长度和宽度,叶片质地的软硬与白色蜡粉的多少,叶鞘的长短和粗细,叶片数目的多少以及叶片的开张程度等,都与品种有关。
前苏联红皮蒜：山东农业大学于 1957 年从前苏联库班蔬菜研究所引进该种红皮蒜。现已大

面积推广，它是中国目前大蒜出口及内销的重要品种之一。有的地区称之为“改良蒜”，有的地区称之为“杂交蒜”。显然，称“杂交蒜”是不正确的，因为大蒜花薹退化，一般不结种子。

前苏联红皮蒜：植株高 85～90cm，株型开张，株幅 40cm 左右。叶片为黄绿色，有蜡粉。蒜头扁圆形，外皮浅紫色至紫色，干燥后外皮呈灰白色带紫色条斑。单头重 55g 左右，蒜头横径 5.5cm，良好栽培条件下有 80%～90%的蒜头直径大于 5cm，瓣形整齐。蒜瓣形状较独特，腹面的上部隆起，下部凹入，容易与其他品种区别。肉质较疏松，辛辣味较淡。每个蒜头有 12～13 瓣，分两层排列，外层蒜瓣肥大而瓣形整齐，内层瓣小且不整齐。蒜衣 1 层，浅红黄色，基部带紫色条斑，较薄，有光泽易剥离。蒜瓣肉质较疏松，辛辣味较淡。休眠期短，不耐储藏

前苏联红皮蒜：该品种适应性较强，适宜在山东、山西、河南、陕西、江苏等秋播地区种植，一般 9 月下旬～10 月上旬播种，第二年 5 月上中旬收获蒜薹，5 月下旬收获蒜头

阿城紫皮蒜：黑龙江省阿城市地方名优品种，已有 800 多年的栽培历史。它具有头大，瓣齐，口味纯正浓郁、辛辣醇香，早熟，耐寒，高产的特点，是东北各省大蒜主栽品种。属低温反应迟钝型品种。

阿城紫皮蒜：株高 85cm 左右，开展度约 32cm，假茎长 33cm、粗 0.8～1.5cm。须根多，每株有须根 40～90 条不等，根长 13～18cm，粗 0.2～1.2mm，根系不分叉。单株叶片数 8～9 片，叶片长 10～40cm、宽 0.7～1.5cm，叶色深绿，叶面有蜡粉。抽薹率 90%以上，粗。蒜头近圆形，外皮灰白色带紫色条纹，横径 5cm 左右，高 4～5.5cm。单头干重 25～50g，蒜衣 1 层，紫红色，不易剥离。每头蒜 6～10 瓣，分两层排列，外层蒜瓣数略多于内层，但蒜瓣大小差异不大，平均单瓣重 4g 左右，蒜瓣间排列紧实。蒜瓣脆嫩、汁黏、味辛辣、芳香、鲜美。它适宜作蒜头和蒜薹生产栽培。该品种在当地 4 月播种，7 月中旬收获蒜头，生育期 100 天左右，为当地的早熟大蒜品种。每亩生产蒜头 600kg。

吉林白马牙：吉林省农安县、和龙市等地农家品种，栽培历史悠久，全省普遍种植

吉林白马牙：植株直立，株高 50～60cm，叶狭长，披针形，长 35～40cm，宽 1.5cm，浅绿色。不易抽薹。蒜头外皮白色，横径 4～6cm，纵径 3～4cm，单头重 30～40g。每个蒜头约有 20 瓣，大小不整齐，瓣狭长呈三角形，辣味较淡。该品种具有中晚熟、长势旺盛、优质高产等特点，属低温反应迟钝型品种。

吉林白马牙：该品种生育期较长，宜作青蒜和春播蒜头栽培。在吉林省各地春季栽培，3 月下旬～4 月上旬播种，7 月下旬～8 月下旬收获蒜头。

天津六瓣红：天津市宝坻区地方品种。株高 65～70cm，假茎高约 26.5cm，粗约 1.5cm。单株叶片数 9 片，叶色浅绿，叶面蜡粉较厚，最大叶长 50.7cm，最大叶宽 2.4cm。抽薹早，蒜薹粗壮，产量高。蒜头扁圆形，横径 5cm 左右，外皮浅紫色，平均单头重 30g 左右。每个蒜头的蒜瓣数一般为 6 瓣，少者 5 瓣，多者 7 瓣，分内、外两层排列，蒜瓣大小相近，瓣形整齐，排列紧实，蒜衣 1 层，暗紫色。

天津六瓣红：该品种适宜作青蒜、蒜薹和蒜头栽培，当地于 3 月上旬播种，第二年 5 月下旬采收蒜薹，6 月下旬采收蒜头。

延吉紫皮蒜（大红袍）：吉林省地方品种，吉林省延吉、吉林地区普遍种植。

延吉紫皮蒜（大红袍）：株高 50cm，叶长披针形，长 40cm、宽 1.8cm，深绿色，成株有 8 片叶左右。蒜头皮色紫色，纵径 3～4cm，横径 4cm，每个蒜头有 6～8 瓣，瓣肥大，呈斜三角形，整齐，有蒜薹。蒜头平均重 20g。生 30 长期 100 天左右，耐寒性强，储藏性中等，辛辣味浓，品质好。

延吉紫皮蒜（大红袍）：该品种在吉林省各地春季栽培，延边地区 4 月上旬播种，畦作或垄作，株距 8～10cm，6 月末收获蒜薹，7 月下旬收获蒜头。

宁夏紫皮大蒜：宁夏回族自治区银川市郊区农家品种。该品种栽培历史悠久，宁夏黄河灌区

等地普遍栽培。
宁夏紫皮大蒜：株高 47cm，开展度 36cm，叶灰绿色，披针形，叶长 45cm、宽 3cm，表面蜡粉多，叶鞘高 18cm，粗 1.3cm，浅紫色，全株有叶 6 片。蒜头高 4cm，横径 5cm，扁圆形，蒜皮紫红色。每个蒜头 4～6 瓣，蒜头重 34g。生长期 130 天。抗寒、耐旱性强，不耐热，蒜苗、蒜薹与蒜头均可食用，辣味较浓，品质优良。
宁夏紫皮大蒜：该品种在银川地区 3 月上旬播种，行距 22cm，株距 6cm。一般在 4 月上旬后出土，5 月中旬开始分瓣，6 月中下旬花薹伸出叶外，采收蒜薹不宜过迟，否则易老化。形成蒜头时浇水，应根据天气、土壤和植株生长状况而定，土壤过湿，容易引起蒜头腐烂和蒜蛆的发生。采收期一般在 7 月中旬
成县红蒜：甘肃省东南部成县地方农家品种，除成县多栽外，天水地区各县也有栽培。
成县红蒜：植株直立，株高 40～45cm，叶呈条带披针形，具白色蜡粉，深绿色，叶长 30～35cm，叶鞘长 10～12cm，蒜薹长 60～70cm，横径 0.4cm。蒜头筒子状，外皮紫红色，纵径 3.8～4.0cm，横径 4.8～5.0cm，每个蒜头 6～10 瓣。
成县红蒜：该品种抗寒性、抗病性强，辣味浓郁，品质好，水分足，蒜瓣脆嫩，生长期 120 天。
成县红蒜：甘肃省东南部秦岭一带，在施肥深翻、整平、耙细后，于 9 月下旬～10 月上旬按行距 33cm，株距 13～17cm，挖 6cm 深的穴，点播蒜瓣 1 个。冬季防寒，于第二年 5 月上旬、中旬可收蒜苗食用，也可待抽薹后，抽取蒜薹，可采薹 300kg/亩，到 6 月上旬，采收蒜头
泾川红蒜：甘肃省泾川县农家品种，平凉地区栽培。株高 60cm，假茎高大蒜的品种选择与特点第三章 3120cm，蒜薹长 50cm 左右，横径 0.6cm。蒜头中等，皮浅红色，纵径 3.5cm，横径 5cm，每个蒜头有 6～8 瓣，蒜头平均重 40g。生长期 120 天，耐寒性强，蒜头瓣大，辛辣味浓，品质好。
泾川红蒜：泾川县 3 月上旬、中旬播种，播前选择蒜瓣肥大、每头 5～6 瓣的作种蒜。株距 17cm，行距 20cm，表面盖土粪。旱地不浇水，水浇地仅在 6～7 片叶时浇水 1～2 次。于 7 月上、中旬采收蒜头。
吉木萨尔红皮蒜：新疆伊犁农家品种，栽培历史悠久。目前，在伊宁市郊、伊宁县广泛栽培
吉木萨尔红皮蒜：株高 75cm，叶长披针形，叶长 55cm、宽 2.6cm，叶色深绿。蒜头皮紫红色，蒜头纵径 4.1cm，横径 5.8cm，每个蒜头有 5～6 瓣。有蒜薹、复瓣。蒜头平均重 81g。生育期 260 天左右。耐寒性强，耐热性中等，耐储藏，抽薹较早，辛辣味浓，品质较好，鲜食、熟食、加工均可
吉木萨尔红皮蒜：该品种在秋季栽培。10 月上旬播种，行距 20～25cm，株距 10cm 左右，第二年 5 月下旬收获蒜薹，6 月底收获蒜头。
喀什紫皮蒜：新疆喀什市地方品种，喀什市郊均有栽培。
喀什紫皮蒜：株高 51cm，叶长披针形，叶长 42cm、宽 2cm，叶色深绿。蒜头外皮紫红色。蒜头纵径 2.5cm，横径 5.5cm，每个蒜头有 7～10 瓣，有蒜薹，有复瓣。蒜头平均重 50g。耐寒性强，耐热性弱，耐储性差。抽薹晚，辛辣味浓，品质较好。宜熟食或加工。
喀什紫皮蒜：该品种在当地一般秋季栽培。10 月中、下旬播种，行距 20cm，株距 10cm。第二年 6 月上、中旬抽薹，6 月下旬～7 月上旬收获蒜头。
蔡家坡红皮蒜：陕西省岐山县蔡家坡地方品种，主要分布在岐山县蔡家坡一带。
蔡家坡红皮蒜：株高 70～85cm，叶长披针形，长 40cm、宽 1.5～1.8cm，叶色深绿，蒜头皮为紫色，纵径 4cm，横径 4～6cm，每个蒜头有 7～8 瓣，有蒜薹，有复瓣。蒜头平均重 60g。早熟种，生育期 250～260 天，耐寒性强，耐热性中等，耐储性强，抽薹早，辛辣味浓，品质好，鲜、熟食及加工均可。

蔡家坡红皮蒜：该品种在当地适宜播期为 8 月下旬，行距 20～25cm，株距 7～10cm，第二年 5 月下旬～6 月上旬收获。蒜薹单产 400kg/亩，蒜头单产 1000kg/亩。

耀县红皮蒜：陕西省耀县地方品种，主要分布在本县境内

耀县红皮蒜：株高 50～60cm，叶为长披针形，长 42cm、宽 2.4cm，绿色。蒜头外皮为紫色，纵径为 3～3.5cm，横径 4～4.5cm，每个蒜头有 5～14 瓣，有蒜薹，有复瓣。蒜头平均重 40g。中早熟，生育期 250～260 天，耐寒性强，耐热性、耐储性均为中等，抽薹早，辛辣味浓，品质中等，鲜、熟食及加工均可。

耀县红皮蒜：该品种在耀县地区的栽培季节为 9 月上旬，当地适宜播期为 8 月下旬～9 月上旬；行距 20～25cm，株距 8～10cm，第二年 5 月下旬～6 月上旬收获。蒜薹单产 400kg/亩，蒜头 1000kg/亩。

商南笨黑皮蒜：陕西省商南县农家品种，主要分布在商南县境内。

商南笨黑皮蒜：株高 44cm，叶为短披针形，长 25～30cm、宽 1.2～1.5cm，绿色。蒜头皮为紫色，纵径为 3.5～4cm，横径 3.5～5.0cm，每个蒜头有 8～9 瓣，无蒜薹，有复瓣。蒜头平均重 30～40g。早熟，生育期 180 天，耐寒性、耐热性均为中等，耐储性强，不抽薹，辛辣味中等，品质较差，鲜、熟食及加工均可。

商南笨黑皮蒜：该品种在当地适宜播期为 12 月中旬，行距 20～25cm，株距 10cm，第二年 6 月上旬收获。

平利火蒜：陕西省平利县地方品种，主要分布在平利县境内。

平利火蒜：株高 36cm，叶形为短披针形，长 20cm、宽 0.8～1.2cm，绿色。蒜头外皮紫色，纵径 3.5cm，横径 4cm，每个蒜头有 6～8 瓣，有蒜薹，有复瓣。蒜头平均重 20～25g。早熟，生育期 230 天，耐寒性、耐热性、储藏性均为中等，抽薹早，辛辣味居中，品质较好，鲜、熟食及加工均可。

平利火蒜：该品种在当地适宜播期为 9 月，行距 20～25cm，株距 6～8cm，第二年 6 月收获。蒜薹单产 200kg/亩，蒜头单产 1500kg/亩。

宁强山蒜：陕西省宁强县农家品种，主要分布在本县境内。

宁强山蒜：株高 50～60cm，叶形为长披针形，长 40～45cm、宽 1.5cm，绿色。蒜头外皮为紫色，纵径 3.5～4cm，横径 4～5cm，每个蒜头有 6～7 瓣，有蒜薹，有复瓣。蒜头平均重 30～40g。早熟，生育期 240～250 天，耐寒性强、耐热性中等、储藏性强、抽薹早，辛辣味浓，品质好，鲜、熟食及加工均可。

宁强山蒜：该品种在当地适宜播期为 9 月上中旬，行距 20～25cm，株距 8～10cm，第二年 6 月上旬收获。蒜薹单产 300kg/亩，蒜头单产 800kg/亩。

三月黄：江苏省大丰市地方品种，该品种抽薹前有明显落黄特性，且时值农历三月，故此得名。

三月黄：株高 70cm，全株有叶 10 片，长 50cm，色深绿。蒜薹长 45～50cm，粗 0.5～0.7cm。蒜头外皮浅紫色，略呈现扁球形，横径 4cm 左右，纵径高 3.3～3.5cm，每个蒜头 8～10 瓣，单头重 30～40g。辛辣味浓，品质好。

三月黄：该品种具有中熟、生长势较旺盛、抗寒性较强、春季落黄明显、高产优质等特点，主要用于青蒜栽培，也可蒜薹和蒜头兼用。在当地作青蒜栽培时 8 月中下旬播种，作蒜薹和蒜头栽培时于 9 月下旬～10 月上旬播种，全生育期 250～255 天。

丹凤火蒜：陕西省丹凤县地方品种，主要分布在本县境内。

丹凤火蒜：株高 50cm 左右，叶为长披针形，长 30cm、宽 1.5～1.8cm，绿色。蒜头外皮为紫色，纵径 3～3.5cm，横径 4cm 左右，每个蒜头有 6～8 瓣，有蒜薹，有复瓣。蒜头平均重 25g。生育期 230～240 天，耐寒性、耐热性、耐储性均为中等，抽薹早，辛辣味浓，品质较好，鲜食、加工均可。

丹凤火蒜：该品种在当地适宜播期为 9 月上旬，行距 20～25cm，株距 6～8cm，第二年 5 月上旬收获。蒜薹单产 200kg/亩，蒜头单产 1000kg/亩

耀县竹叶青：陕西省耀县地方品种，主要分布在本县境内

耀县竹叶青：株高 50～60cm，叶为长披针形，长 30～40cm、宽 1.5cm，绿色。蒜头外皮为浅紫色，纵径 3～3.5cm，横径 4～4.5cm，每个蒜头有 6～8 瓣，有蒜薹，有复瓣。蒜头平均重 20～25g。中晚熟，生育期 250 天左右，耐寒性强，耐热性、耐储性中等，抽薹早，辛辣味浓，品质好，鲜、熟食及加工均可。

耀县竹叶青：该品种在当地适宜播期为 8 月下旬～9 月中旬，行距 20～25cm，株距 8～10cm，第二年 6 月中旬收获，蒜头单产 1000kg/亩。

应县紫皮蒜：山西省应县小石口地方品种，栽培历史悠久，在应县及邻近县有栽培

应县紫皮蒜：株高 50～60cm。叶片长披针形，深绿色。蒜头外皮浅紫红色，蒜头纵径约 4cm，横径约 5cm。每个蒜头有 4～6 瓣，无复瓣，单个蒜头重 25～30g。中熟，生长势强，病虫害较轻。抽薹率为 50%～80%。蒜瓣肉质致密，香辛味浓，蒜泥黏稠隔夜不变味，品质好。

应县紫皮蒜：该品种在应县 3 月下旬～4 月初播种，7 月下旬～8 月上旬收获蒜头。

云顶早：四川省成都市地方品种，在市郊青白江区和金堂县分布较多。

云顶早：株高 60cm，开展度 28cm。叶片长 38cm，绿色。假茎高 20cm，浅绿色，叶面蜡粉少。蒜头扁圆形，高 2.2cm，横径 3cm，外皮紫红色，每个蒜头有 8～9 瓣，单个蒜头重约 10g。从播种至收获 210 天左右。耐寒、耐热性较强。薹质细嫩，味香甜，辣味重，蒜苗香味浓。适宜收蒜薹或蒜苗。

云顶早：该品种在当地以收获蒜薹为主时，8 月中旬播种，行距 20cm，株距 7cm，3 月上旬收获蒜薹，单产 250kg/亩。以收获青蒜苗栽培时，6 月下旬～7 月上旬播种，行距约 5cm，成行密植。施肥后覆以稻草，出苗 20～30 天追肥。11 月上旬～第二年 1 月收获。

二水早：四川省彭州市和金堂县地方品种，属于硬秸大蒜品种类型。

二水早：株高 60～80cm，假茎长 30～40cm，粗 1.2cm 左右。最大叶长 35～40cm，最大叶宽 2.5cm，颜色绿，叶面蜡粉较多。蒜薹色浅绿，味浓，品质好。蒜头圆形，外皮浅紫色，单头重 13～20g。每个蒜头有 8～9 瓣，多分两层排列，外层为 6 瓣，内层为 2～3 瓣，瓣衣 2 层，紫红色，较厚，干后易变成褐色。

二水早：该品种早熟、耐热、耐寒、适应性强、抽薹早，适宜作青蒜和蒜薹栽培。作青蒜栽培在当地于 8 月中下旬播种，第二年 1～2 月收青蒜；作蒜薹栽培时 9 月中旬播种，第二年 3 月下旬～4 月上旬抽薹，5 月上旬收蒜头。

温江红七星：又名硬叶子、刀六瓣，四川省温江县地方品种。成都郊区各县均有分布。

温江红七星：株高 50～60cm，开展度 18cm，叶片长 30～40cm。假茎高 20cm，叶色略带紫色，蜡粉少。蒜头扁圆形，高 3cm，横径 4～5cm，皮白带紫色，每个蒜头有 7～8 瓣，分两层排列，蒜衣 2 层，浅紫色，不易剥离。单个蒜头重 20～30g。

温江红七星：该品种从播种至收获约需 240 天。耐寒性较强，稍耐旱而不耐涝。薹质细嫩，香味浓郁，蒜薹辣味较重，以收蒜薹和蒜苗为主。

温江红七星：该品种在当地 9 月下旬播种，行距约 20cm，株距约 12cm。蒜薹 4 月收获，蒜头 5 月上旬上市，单产蒜薹 200kg/亩，蒜头 1000kg/亩

舒城大蒜:安徽省舒城县地方品种。

舒城大蒜:该品种属于白皮蒜，中熟。植株高 80～90cm，全株 8～9 片叶，叶片深绿色，最大叶长约 46cm，宽约 3cm，表面无蜡粉。抽薹较早，味浓，品质优。蒜头扁圆形，纵径 4.5～5cm，横径 6cm，单头重 50g 左右。每个蒜头有 9～13 枚蒜瓣，蒜衣白色，蒜瓣大而均匀，辣味浓。

舒城大蒜:该品种宜作蒜头和青蒜栽培。在当地于 8 月下旬～9 月中旬播种，第二年 4 月下

旬～5 月上中旬采收蒜薹，5 月下旬～6 月上旬收蒜头，生育期 240～260 天。
隆回大蒜:湖南省隆回县农家品种，分布于邵阳地区及湘西地区。
隆回大蒜:株高 63cm，叶片先端向上，叶片长披针形，浅绿色，蜡粉少，叶鞘绿白色。蒜头高圆形，皮紫红色，肉白色，单株重 25～62g，单个蒜头重 30～50g，每个蒜头有 10～14 瓣。
隆回大蒜:该品种早熟，不耐旱，耐热，较耐寒，抗病性强，青蒜及蒜薹在常温条件下不耐储藏。含水分少，香气浓，蒜头较耐储藏。品质较好
隆回大蒜:该品种在当地 9 月上旬用蒜瓣点播。开定植沟播种，播后盖草，经常保持土壤湿润。第二年 4 月下旬～5 月上旬收蒜薹，6 月上旬收蒜头
东安大蒜:湖南省东安县地方品种，该省部分城市郊区有栽培。
东安大蒜:株高 55cm，开展度 30cm，叶片长披针形，绿色，长 50cm、宽 2cm，叶片稍下垂，蜡粉少，叶鞘浅紫红色。假茎长 10cm，粗 1.3cm。蒜茎上部绿色，下部白绿色，长 65cm，粗 0.6cm。每个蒜头有 8～12 瓣。外皮白色稍带浅红色，蒜头高圆形，底平，单个蒜头重 40～50g。中晚熟，耐寒、耐肥，不易散瓣。辛辣味较浓，水分少，香气浓，肉质细，品质好。
东安大蒜:该品种在当地 8 月上旬～9 月中旬播种。行距 17cm，株距 15cm，播种后用稻草覆盖。单产蒜薹 200～300kg/亩，蒜头 700～750kg/亩
茶陵紫皮蒜湖南省茶陵县农家品种，湘中地区多有分布。
茶陵紫皮蒜株高 45～50cm，叶片长披针形，长 42cm、宽 2.4cm，深绿色，叶面有少量蜡粉，叶鞘绿白色。基部紫红色。假茎长 12cm。蒜头扁圆形，上尖下大，紫红褐色。纵径 4.2cm，横径 4.8cm。单头重 30～45g，每个蒜头有 8～10 瓣。蒜薹长 50cm，粗 0.8cm。
茶陵紫皮蒜中晚熟，播种至收青蒜 60～100 天。到收蒜头需 250～270 天。植株生长势较弱，前期生长慢，春暖后生长快。耐寒性较弱，较耐热，耐涝性中等，不耐旱，抗病虫能力强。蒜薹脆嫩香甜，蒜头紧凑，瓣大匀称，辛辣味强，品质较好。
茶陵紫皮蒜该品种在当地 8 月上旬～9 月中旬播种。从 11 月～第二年 4 月可收青蒜。5 月上旬～6 月上旬采收蒜薹，6 月下旬采收蒜头。
中牟大蒜（宋城大蒜）河南省中牟县主栽品种，引进已有数十年，开封、郑州等地也有大量种植。
中牟大蒜（宋城大蒜）株高 50～60cm，开展度 25cm。叶为长扁条形，深绿色，蜡粉中等，叶背呈龙骨状，叶鞘绿白色，假茎粗 1.7cm，蒜头扁圆形，外皮浅紫红色，纵径 4.2cm，横径 5～7cm，一侧内生小蒜头，鲜蒜头重 80g 左右，干蒜头重 40～50g。每个蒜头有 10～12 瓣。该品种耐寒性强，不耐热。产量高，辣味淡，可生食、熟食或腌渍。
中牟大蒜（宋城大蒜）该品种在河南地区于 9 月上、中旬播种，平畦栽培，行距 20cm，株距 10cm，第二年 5 月上、中旬收获蒜薹，6 月上、中旬收获蒜头。收获前 15 天应停止浇水，同时要及时收获，防止散瓣。蒜薹产量 300kg/亩，鲜蒜头产量 1300～2000kg/亩。
嘉祥大蒜山东省嘉祥县地方品种，栽培历史悠久，在本县及鲁西南部分地区种植。
嘉祥大蒜在良好栽培条件下，株高 50cm 左右，叶半直立，长披针形，深绿色，中部以上大叶长 35cm，宽 2.2cm。鲜蒜头外皮灰紫色，鳞芽外皮浅紫色，蒜头平均重 30～40g。蒜薹较发达，叶鞘以上、总苞以下的蒜薹长 30cm，单根蒜薹重 8～10g。
嘉祥大蒜中熟，秋季播种到蒜头成熟 255 天左右。抗寒性强，耐热，蒜薹较细，品质较好。蒜头休眠期长，蒜头耐储性强，辛辣味浓，品质好。适合鲜食和加工。
嘉祥大蒜该品种在山东主产区 9 月下旬～10 月上旬播种，行距 18cm，株距 10cm。5 月下旬收蒜薹，一般产量 250kg/亩。6 月上旬收蒜头，一般产量 1200kg/亩
上高大蒜江西省上高县农家品种，上高县主产，全省部分县有栽培。

上高大蒜株高 62cm，叶长披针形，长约 50cm、宽 2.5cm，绿色。蒜头外皮紫色，纵径 4.9cm，横径 5.4cm，每个蒜头有蒜瓣 8 枚左右。有蒜薹，无复瓣。蒜头平均重约 50g。中熟，生育期 240～250 天。耐寒性强，不耐热，耐储藏，抽薹性中等。辛香味较浓，叶肉肥厚，纤维少，品质优良。主要以青蒜熟食。

上高大蒜该品种应秋播越冬栽培。当地适宜播期为 9 月上旬～10 月中旬。作青蒜栽培的，行距 13cm，株距 7cm。11 月上旬～第二年 3 月采收青蒜，单产 3000kg/亩，4 月中、下旬收获蒜薹，单产 250kg/亩。5 月中、下旬采收蒜头，单产 500kg/亩。

都昌大蒜江西省都昌县农家品种，都昌县主产，九江地区普遍栽培。

都昌大蒜株高 52cm，叶长披针形，长约 43cm、宽 3.2cm，深绿色。蒜头皮紫红色，纵径 3.5cm，横径 4.7cm，每个蒜头有蒜瓣约 8 枚。有蒜薹，无复瓣。蒜头平均重约 30g。

都昌大蒜晚熟，生育期 260 天左右。耐寒性强，耐储藏，抽薹晚。辛香味浓，品质优良

都昌大蒜该品种应秋播越冬栽培。当地适宜播期 9 月上、中旬。作青蒜栽培的，行距 17cm，株距 13cm，12 月上旬～第二年 3 月采收青蒜，单产 2000～2500kg/亩，4 月中、下旬收获蒜薹，单产 200～300kg/亩。6 月上旬收蒜头。

苍山大蒜山东省苍山县地方品种，是山东省传统名特优蔬菜之一。苍山大蒜又分为（1）苍山蒲棵（2）苍山糙蒜　（3）苍山高脚子三个品种。

苍山蒲棵属于硬秸大蒜品种类型，是目前山东省苍山蒜区种植面积最大的秋播品种，约占苍山县种植面积的 90%以上。株高 80～90cm，株幅 36cm 左右。叶色深绿，蒜头近圆形，皮白色，纵径 3.5～4.0cm，横径 3.5～4.5cm，有蒜瓣 4～6 瓣，大而整齐，蒜衣 2 层，稍呈红色，平均单瓣重 3.5g 左右，辛辣味浓。蒜薹长 35～65cm，单薹重 15～35g，一般每亩产蒜薹 500kg 左右，蒜头 800～900kg。品质优，耐储藏。耐寒性较强，适应性强，生育期 240 天左右。

苍山糙蒜　属于硬秸大蒜品种类型。株高 75～80cm，叶色深绿；蒜头近圆形，皮白色，纵径 3.5～4.0cm，横径 3.5～4.5cm，有蒜瓣 4～6 瓣，大而整齐，蒜衣 2 层，稍呈红色，平均单瓣重 3.5g 左右，辛辣味浓。蒜薹长 35～50cm，单薹重 15～35g，品质优，耐储藏。耐寒性较强，生育期 235 天左右

苍山高脚子　属于硬秸大蒜品种类型。株高 85～95cm，叶色深绿；蒜头近圆形，皮白色，纵径 3.5～4.0cm，横径 3.5～4.5cm，有蒜瓣 4～6 瓣，大而整齐，蒜衣 2 层，稍呈红色，平均单瓣重 3.5g 左右，辛辣味浓。蒜薹长 35～70cm，单薹重 15～35g，品质优，耐储藏。耐寒性较强，生长势强，生育期 250 天左右。

.哈密白皮大蒜新疆农家品种，栽培历史悠久，巴里坤、伊吾县及哈密县均有栽培

.哈密白皮大蒜株高 50～60cm，叶片长披针形，叶长 40cm，宽 2～3cm，叶色绿，蒜头外皮白色，蒜头纵径 3.5cm，横径 5～6cm，每个蒜头 20 瓣左右。有蒜薹，有复瓣。蒜头重 50g。

.哈密白皮大蒜该品种晚熟，春播生育期 140～150 天。耐寒性较强，耐热性中等，耐储性强，抽薹晚，辛辣味中等，品质好。鲜、熟食均可。

哈密白皮大蒜该品种在当地以春栽为主，4 月中旬播种，行距 20cm，株距 8～10cm，9 月上、中旬开始收获。

呼沱大蒜陕西省洋县农家品种，主要分布在本县境内。

呼沱大蒜株高 66cm，叶为长披针形，长 45cm、宽 1.9cm，绿色；蒜头皮为白色，纵径 3.5～4cm，横径 4～6cm，每个蒜头有 6～8 瓣，有蒜薹，有复瓣。蒜头平均重 30～40g。

呼沱大蒜中晚熟，生育期 260 天，耐寒性强，耐热性、耐储性、抽薹性均为中等，辛辣味浓，品质好。鲜、熟食及加工均可。

呼沱大蒜该品种在当地适宜播期为 8 月中旬～9 月中旬，行距 25cm，株距 10cm，第二年 6 月中旬收获。蒜薹单产 500kg/亩，蒜头单产 1500kg/亩

留坝白蒜陕西省留坝县农家品种，主要分布在本县境内。
留坝白蒜株高 44cm，叶为长披针形，长 30cm、宽 1.2cm，绿色，蒜头外皮为白色，纵径 3cm，横径 3.5cm，每个蒜头有 8～9 瓣，有蒜薹，有复瓣。蒜头平均重 20～30g。
留坝白蒜该品种在当地适宜播期为 8 月下旬～9 月上旬。行距 20～25cm，株距 6～8cm。收获期在第二年 6 月上旬。蒜薹单产 300kg/亩，蒜头单产 500kg/亩。
留坝白蒜中晚熟，生育期 250～260 天，耐寒性、耐热性、耐储性、抽薹性以及辛辣味均居中等，品质一般。鲜、熟食及加工均可。
兴平白皮蒜陕西省兴平县农家品种，主要分布在本县境内
兴平白皮蒜株高 60～70cm，叶为长披针形，长 40～50cm、宽 1.5cm，叶色深绿。蒜头外皮为白色，纵径 3.5～4cm，横径 4cm，每个蒜头有 7～9 瓣，有蒜薹，有复瓣。蒜头平均重 30g。
兴平白皮蒜晚熟，生育期 260～270 天，耐寒性强，耐热性中等，耐储性强，抽薹晚，辛辣味浓，品质中等。鲜、熟食及加工均可
兴平白皮蒜该品种在当地适宜播期为 9 月上旬。行距 20～25cm，株距 8～10cm，第二年 6 月中、下旬收获。蒜薹单产 300kg/亩，蒜头单产 800kg/亩。
陇县大蒜陕西省陇县农家品种，主要分布在本县境内。
陇县大蒜株高 70cm，叶为长披针形，长 40～50cm、宽 1.5～1.8cm，绿色。蒜头外皮为白色，纵径 3.5～4cm，横径 5～6cm，每个蒜头有 6～8 瓣，有蒜薹，有复瓣。蒜头平均重 30～35g。
陇县大蒜中熟，生育期 240～250 天，耐寒性强，耐热性、耐储性、抽薹性均属中等，辛辣味浓，品质较好。鲜、熟食及加工均可
陇县大蒜该品种在当地适宜播期为 9 月中旬，行距 25cm，株距 8～10cm，收获期为第二年 6 月。蒜薹单产 300kg/亩，蒜头单产 1300kg/亩
太仓白蒜（粳蒜 1 号）江苏省太仓县地方农家品种，主要分布在太仓及周边市、县及上海嘉定。
太仓白蒜（粳蒜 1 号）株高 61cm，成株有叶 10 片。叶呈条带披针形，绿色，叶长 4149cm，蒜薹粗壮，长 49cm，横径 0.71cm。蒜头近圆球，扁圆形，外被白色膜质鳞片，内有蒜瓣 7～8 枚，单个蒜头重 52g。早熟。为青蒜、蒜薹、蒜头兼用品种，嫩鳞茎还可腌渍或加工糖醋蒜。耐寒性强，辛辣味浓郁，商品性优良
太仓白蒜（粳蒜 1 号）该品种在江苏太仓县，一般施农家优质腐熟有机肥作基肥，深翻、整平，精选蒜种，合理密植，适时早播，一般于 9 月下旬播种，5 月下旬，可收获蒜头。
嘉定大蒜（嘉定白蒜、老脱须）上海市嘉定区地方品种，有百余年的栽培历史，嘉定区娄塘乡一带大面积种植。
嘉定大蒜（嘉定白蒜、老脱须）植株高 60～80cm，较粗壮。叶片绿色，长剑形，叶长 40cm，宽 2～3cm，蒜薹长 70cm 左右，横径 0.7cm。蒜头纵径 3～4cm，横径 4～5cm，蒜皮白色，蒜瓣肥大，7～8 瓣。蒜头重 75～100g。晚熟，生长期 240～250 天，生长势强，抗寒力强。蒜头品质好，色泽洁白，蒜瓣粗壮，排列均匀，肉质脆嫩，辛辣味浓。成熟后其须根自行脱落，故名老脱须。蒜苗、蒜薹及蒜头品种，供熟食、调味或加工
嘉定大蒜（嘉定白蒜、老脱须）该品种在上海地区于 9 月下旬～10 月播种，用种量 120kg/亩。5 月上旬采收蒜薹，5 月下旬～6 月上旬收获蒜头。
临颍大蒜河南省临颍县农家品种，栽培历史悠久，属河南名特产品。
临颍大蒜株高 45～50cm，开展度 20cm。叶为长披针形，深绿色，蜡粉多，叶背呈龙骨状，叶鞘绿白色，假茎粗 1.2cm。蒜头扁圆形，外皮白色，纵径 2.9cm，横径 4.2cm，每个蒜头一般有 4～6 瓣，鲜蒜头重 30g，干蒜头重 16g。耐寒性强，不耐热，播种到收获蒜薹 240

天左右，播种到收获蒜头 270 天左右。产量较低，辣味浓，可生食、熟食或腌渍。
临颍大蒜该品种在河南省中牟县一带于 9 月上、中旬播种，平畦栽培，行距 20cm，株距 10cm，第二年 5 月上、中旬收获蒜薹，6 月上、中旬收获蒜头。
金山火蒜（开平大蒜）广东省开平市农家品种。主栽于广东省开平市、台山市。
金山火蒜（开平大蒜）植株高 50～70cm。叶长披针形，长 60cm、宽 2.5cm，黄绿色，较柔软。假茎长约 16cm，下部较粗，横径 3cm，上部横径 1.7cm，白色。蒜头扁球形，颈部细小，基部凹入，纵径 4cm，横径 6cm。鳞片较薄，白中带紫。每个蒜头有 6～8 瓣。
金山火蒜（开平大蒜）早中熟，生长期 140～150 天。耐寒，稍耐湿，不耐热。不抽薹，以收获蒜头为主。辣味浓，品质优。
金山火蒜（开平大蒜）该品种在当地播种期为 10 月中旬～12 月上旬，收获期为 3～4 月。
开原大蒜辽宁省开原市地方品种，具有极早熟、生长快等特点，属于低温反应迟钝型品种
开原大蒜株高 89cm，开展度 34cm，假茎长 34cm、粗 1.4cm。单株叶数 10～11 片，最大叶长 60.5cm，最大叶宽 2.7cm。蒜头近圆形，外皮灰白色带紫色条纹，横径 4.7cm，平均单头重 32g。每个蒜头有 7～11 瓣，分两层排列，平均单瓣重 3.5g。蒜衣 1 层，暗紫色，易剥离。
开原大蒜该品种在当地行春播，于 3 月下旬播种，6 月下旬～7 月上旬收获蒜头，生育期 100 天左右。
海城大蒜辽宁省海城市耿家庄地方品种，又名耿庄大蒜。其具有极早熟、头大瓣少、品质佳等特点，销往黑龙江、吉林、内蒙古等省（自治区）及加拿大、罗马尼亚、日本等国，属于低温反应迟钝型品种。
海城大蒜株高 75cm，开展度 47cm，株型较开张。叶片浅绿色，叶面有蜡粉。蒜头近圆形，外皮灰白色带紫色条纹，平均单头重 50g 左右，最大的达 100g。每个蒜头有 5～6 瓣，瓣形整齐，蒜瓣肥大，香辣味浓，捣出的蒜泥不易变味。宜作蒜头栽培。
海城大蒜该品种在当地行春播，于 3 月下旬播种，6 月上旬采收蒜薹，7 月上旬收获蒜头，生育期 110 天左右。每亩产蒜薹 100kg、蒜头 1000kg。
临洮红皮蒜甘肃省临洮县地方品种，为当地主栽品种，有临洮大麻蒜和临洮红蒜两个品系，二者性状基本相似，属于低温反应迟钝型品种
临洮红皮蒜株高 73cm，开展度 28cm。假茎长度 21cm、茎粗 1.5cm。单株叶片 15～16 片，最大叶长 57cm，最大叶宽 2.8cm。可抽薹，但蒜薹短小。蒜头近圆形，外皮浅褐色带紫色条纹，横径 4.5cm 左右。平均单头蒜重 30g 左右，每个蒜头有 12 瓣，多者 14 瓣，分两层排列，外层蒜瓣数少但较大，平均单瓣重 2.3g 左右。主要作蒜头栽培。
临洮红皮蒜该品种在当地行春播，一般于 3 月上中旬播种，7 月中下旬收获蒜头，生育期约 130 天。
土城大蒜内蒙古自治区呼和浩特市和林格尔县土城子乡地方品种，属于低温反应迟钝型品种。
土城大蒜株高 75cm，开展度 30cm，假茎长 15cm、粗 1.1cm。单株叶数 8～9 片，最大叶长 57cm，最大叶宽 2.6cm。蒜头近圆形，外皮灰白色带紫色条纹，横径 4.6cm，平均单头蒜重 28g 左右，大者达 50g。每个蒜头有 8～9 瓣，一般分 3 层排列。
宁蒜 1 号黑龙江省宁安市农科所由当地农家品种紫皮蒜为材料，经辐射处理选育而成，于 1990 年通过黑龙江省农作物品种审定委员会审定。
宁蒜 1 号植株生长旺盛，叶片呈条带披针形，绿色，较直立，株高 65cm 左右，抽薹时上冲，薹长 42cm，后期蒜薹尖端下垂，蒜头纵径 4.0～4.5cm，横径 4.5～5.5cm，单头重 45g 左右，蒜头扁圆形，外皮紫红色。抗旱、抗病力强，耐储运，喜肥水、疏松肥沃沙性土壤，辛辣味浓，口感好，品质佳。生长期 95～100 天

宁蒜 1 号该品种在黑龙江省牡丹江地区一般于 3 月下旬或 4 月上旬播种。施足基肥深翻，整平、耙细后，可按大垄 50cm，单条播，株距 6cm；双条播，株距 8cm。也可平畦平作，行距 15cm，株距 8cm 栽种。7 月中旬收蒜头。
早薹蒜一号由西北农林科技大学园艺系和山东农业大学园艺系选育。
早薹蒜一号株高 75～80cm，最大叶长 65cm，叶宽 2.2cm。蒜薹直径为 0.6～0.8cm，长 55～65cm，蒜薹口感细腻、味甜，品质佳。鲜蒜头平均横径 5cm，纵径 3.5～4cm。外皮紫红色，每个蒜头有 9～10 瓣，瓣紧，单头重 20～30g。
早薹蒜一号该品种为蒜薹、蒜头兼用的早熟品种。一般在 8～9 月，第二年 4 月中旬～5 月初收获蒜薹，5 月上中旬收蒜头
早薹蒜二号由山东农业大学和西北农业大学共同选育的大蒜品种，于 1997 年通过山东省品种审定委员会认定
早薹蒜二号植株生长势强，高 75～80cm，假茎粗约 1.8cm。单株叶片数 12 片左右，最大叶长约 54cm，最大叶宽 4cm。抽薹早、抽薹率高，可达 100%，蒜薹长 50cm 左右，粗约 0.9cm。蒜头近圆形，外皮灰白色带紫色条斑，横径 4～5cm，单头蒜重 30～35g，每个蒜头有 7～8 瓣，分两层排列。蒜衣 2 层，紫色，易剥离。
早薹蒜二号该品种适宜作早薹蒜和蒜头栽培，在陕西杨陵和山东泰安、成武、巨野等地 9 月中旬播种，第二年 4 月中旬采收蒜薹，5 月中旬采收蒜头。生育期为 240 天。
金蒜 3 号由山东润丰种业有限公司从金乡紫皮变异株无性系选育成。
金蒜 3 号该品种生育期 243 天，株高约 100cm，株型较大，假茎粗 1.8～2.0cm；叶色深绿，总叶片数 17 片；蒜头外皮微紫红，高 4.9～5.4cm，单头直径 5.5～6.0cm，单头重 70～80g；蒜瓣外皮紫红色，大小均匀，排列整齐而紧凑；单头瓣数外缘 9～10 瓣，内层 3～5 瓣。蒜薹直径约 0.6cm，长度约 70cm；抽薹率 96.4%。
=鲁蔬白蒜由山东省农业科学院蔬菜研究所育成的大蒜品种，蒜头全白皮品种。
鲁蔬白蒜株高 75～95cm；蒜头近圆形，外皮白色，纵径 3.5～4.0cm，横径 5.0～6.5cm，单头重 50～100g，每个蒜头有 8～12 瓣，大而整齐，有少许复瓣，肉质细嫩、辛辣味淡。蒜衣 1 层，浅黄色。
鲁蔬白蒜该品种具有耐热、皮白、耐储性中等等特点，适宜作蒜头栽培，在山东省适宜播种时间为 9 月下旬～10 月上旬。生育期 240 天左右。
鲁蒜 2 号由山东省农业科学院蔬菜研究所育成的蒜头用大蒜品种。
鲁蒜 2 号株高 80～95cm，茎较粗壮，叶片 9～11 片，绿且厚，叶长 55cm，宽 2.8cm。蒜薹收获期一致。薹色浅绿，长 55～60cm，粗 0.6cm。蒜头扁圆形，外皮白色略有微紫斑，蒜头横径 5.5～6.5cm，每个蒜头有 9～12 瓣，1～2 枚夹瓣，单头重 60～100g。蒜衣 1 层，浅红色
鲁蒜 2 号该品种具有抗病、耐退化等特点，适宜作蒜头栽培。在山东省适宜播种时间为 9 月下旬～10 月上旬，生育期 250 天左右
成蒜早该品种是成都市农业科学研究所从地方良种“二水早”中经多代选育而成的大蒜品种
成蒜早植株紧凑，株高约 60cm，假茎长 40cm 左右，粗约 1.3cm。单株叶数 14 片，叶色深绿，蜡粉多，叶长 50cm，叶宽 2.1cm 左右。薹长约 55.6cm，粗约 0.7cm，薹色绿。蒜头横径 3.23cm 左右，纵径约 3.36cm，外皮紫色。每个蒜头有 10～11 瓣，蒜衣深紫色。
成蒜早该品种是蒜薹和蒜头兼用品种，具有中早熟特性。在四川省最佳播种期为立秋到处暑，全生育期约 199 天。
大蒜鳞茎膨大和抽薹都对生态条件有严格的要求，而且引种优良实践证明，一般大蒜品种的生态适应性不强。因此，生产中品种的选择首先应该因地制宜，优先考虑就地选用的原则，充分发掘地方名优大蒜品种资源。

在当地优良品种资源不足，需要引进优良品种时，应该考虑从地理位置相近的地区引种，并且经引种试验筛选。地理位置包括纬度和海拔，纬度相近的地区，光周期相近；海拔相近的地区，气候条件比较相似，一般可以满足大蒜生长发育对环境的要求，引种和选用品种的成功率相对较高。

由于长期生长在一个地区的品种，对当地或育种地的生态环境产生了一定的适应性，或形成了一定的需要，当引入其他地区种植时，有的适应性差的品种可能会因生长环境的改变而不能适应，因而表现不良。

所以，在品种选择时要充分了解品种的生态适应性和生物学适应性，避免因盲目引种和未经引种试验而大面积使用新品种带来的生产损失。

一般来说，选用来自生态型相近地区的品种容易成功，而生态型差异太大的品种引种容易失败。但是，也要注意通过生态型差异较大地区品种的引进和种植试验，发现异地品种的生物学适应性的特点，以充分挖掘适应性强的异地品种。

大蒜产品多样，每茬生产要收获的主要产品不同，品种选择应注意生产的主要目的

作青蒜苗栽培的应选择休眠期短、发芽和苗期生长快、叶色绿、质脆味浓的品种；以蒜头生产为主要目的的，应选择蒜瓣肥大、品质优良、休眠期较长的品种；以蒜薹生产为主要目的的，应选择抽薹率高、蒜薹粗细适中、品质鲜嫩、耐储运的品种。

各地人口组成不同，消费习惯也有一定的差异。有的地区人们喜食红皮蒜，有的则喜食白皮蒜；有的喜食辛辣味浓的，有的喜食味道柔和的。品种选择时首先要考虑这一因素。当地销售的产品，品种要适合销售地人的消费习惯。

一个地区规模化生产时，应注意品种早、中、晚熟型的搭配，蒜薹、蒜头等不同用途品种的搭配，鲜食与储藏加工品种的搭配等，既有主栽品种，又有配套品种。

青蒜是以食用鲜嫩的大蒜假茎和叶片为主的。

因此在品种布局安排上：要选择出苗快、苗期生长发育快、组织鲜嫩、叶色翠绿、外观商品性好的大蒜品种；要选择休眠期较短、易醒眠的品种，以便于炎夏栽培早秋青蒜，或选择休眠期长、耐储存的品种，以便早春栽培青蒜；要选择瓣数较多、蒜瓣壮实而营养储存充足的品种；要选择耐热抗寒，适应性、抗逆性较强的品种，供反季节青蒜栽培之用。如软叶蒜、三月黄、雪里青、都昌紫皮蒜、隆安红蒜、蔡家坡红皮蒜、衡阳早薹蒜、二水早、嘉祥大蒜、太仓白蒜、鲁农大蒜、宋城大蒜、徐州白蒜、金乡大蒜、临洮白蒜和白皮狗牙蒜等，以及当地名优青蒜品种。

蒜黄是青蒜软化栽培的结果，也是以食用大蒜的假茎和叶片为主的，不仅要求组织脆嫩，而且要求假茎洁白、叶色金黄。因此在品种布局安排上：要选择休眠期较短、醒眠早且快、耐热性较强的品种，以供秋季蒜黄栽培用；或选择休眠期较长、耐储存、抗寒性较强的品种，以供冬、春季蒜黄栽培用；或选择头大、瓣壮、出苗快且苗期生长发育快、株高、茎粗、叶肥厚宽大的品种。如前苏联红皮蒜、鲁农大蒜、徐州白蒜、金乡大蒜、嘉祥大蒜、太仓白蒜、来安大蒜、超化大蒜及当地名优蒜种。

薹蒜是以食用和加工脆嫩、粗壮的大蒜花茎（蒜薹）为主的。

因此在品种布局安排上：要选择苗期生长发育快、生长势强、易通过低温春化阶段、抽薹早的中早熟品种，力争早上市；要选择营养优势强，蒜薹粗壮、脆嫩、色绿、耐储藏的中熟或中晚熟品种。如二水早、彭县中熟蒜、衡阳早薹蒜、早薹蒜二号、都昌紫皮蒜、来安大蒜、青龙白蒜、太仓白蒜、嘉定大蒜、苍山大蒜等，以及当地名优大蒜

蒜头是以食用和加工大蒜头为主的。

因此在品种布局安排上：48 要求选择营养体优势强、抽薹性较弱、蒜头大而圆整的品种；要选择瓣数不多且瓣形周正、较耐储存的品种；要选择抗逆性较强、蒜味浓香、蒜汁黏稠、蒜肉洁白有咬劲的品种。如前苏联红皮蒜、金乡大蒜、宋城大蒜、鲁农大蒜、嘉祥大蒜、嘉

定大蒜、太仓白蒜、苍山大蒜、青龙白蒜等，以及当地名优头蒜。

在大蒜生长发育过程中，正常情况下生长到一定时期，鳞芽开始分化，叶片退化，鳞芽逐渐形成蒜瓣，退化的鳞芽外的鳞片形成了蒜瓣的外皮。

在不利的环境条件下，大蒜在蒜薹露出之前，蒜轴周围长出 5～6 片小蒜叶，围着蒜轴生长。新蒜瓣的外皮顺着蒜薹长出的一条长长的新叶，而新蒜瓣的生长点并没有萌动，称为二次生长现象，也称为马尾蒜、大蒜发叉。

侧芽在形成蒜瓣的过程中，不经过休眠，顶芽又萌发长出许多细长的丛生叶，状如马尾和松毛，这种出叶现象对蒜薹和蒜头的产量影响较大。

分杈的马尾蒜到后期也能抽生蒜薹，称为“二次抽薹”，但都很细小，无商品价值。

这些抽薹蒜的基部也能形成蒜头，但多为畸形的复瓣蒜或散瓣蒜

外层型二次生长：大蒜植株外层叶片的叶腋中萌生一至数个鳞芽，鳞芽延迟进入休眠而继续分化和生长，形成独瓣蒜，或没有花薹的分瓣蒜，或有花薹的分瓣蒜，结果在蒜头的外围着生一些排列错乱的蒜瓣或小蒜头，使整个蒜头成为畸形。这种类型的二次生长对商品品质的影响最大。

内层型二次生长：在大蒜植株内层叶片的叶腋中，正常分化的鳞芽延迟进入休眠，鳞芽外围的保护叶继续生长，从植株的叶鞘口伸出，形成多个分杈。有的分杈发育成正常的蒜瓣；有的分杈发育成分瓣蒜，其中有少数分瓣蒜还形成了花薹。轻度的内层型二次生长对蒜头的外形影响不大，发生严重时，蒜薹变短，薹重降低，蒜瓣排列松散，蒜头上部易开裂，所形成的分瓣蒜外观酷似一个肥大的正常蒜瓣，常被选作蒜种，但播种后由一个种瓣中长出二至多株蒜苗，从而影响所生蒜头的产量和质量。

气生鳞茎型二次生长：蒜薹总苞中的气生鳞茎延迟进入休眠而继续生长成小植株，甚至抽生细小的蒜薹。发生气生鳞茎型二次生长的植株，常使蒜薹短缩，丧失商品价值，但对蒜头的影响不大。这种类型的发生率一般很低。

一些学者根据二次生长在大蒜植株上发生的部位，可将其分为外层型二次生长内层型二次生长气生鳞茎型二次生长三种类型。

大蒜二次生长类型及发生的严重程度与品种遗传性有关。

大蒜二次生长的产生原因有只发生内层型二次生长，不发生外层型二次生长的品种内层型及外层型二次生长均可发生的品种不发生二次生长的品种三种情况

只发生内层型二次生长，不发生外层型二次生长的品种　有软叶、温江红七星、前苏联红皮蒜系统的品种（改良蒜、徐州白蒜、鲁农大蒜、宋城大蒜等）、天津红皮、上海嘉定蒜、新疆伊宁红皮、新疆吉木萨尔白皮、青海格尔木红皮、甘肃民乐大蒜、乐都大蒜、临洮白蒜、临洮红蒜、辽宁开原犬蒜、江苏太仓白蒜、内蒙古 102 土城小瓣、土城大瓣、延安白皮、银川紫皮、白皮狗牙蒜、黑龙江阿城白皮、阿城紫皮、广西紫皮、陕西耀县红皮、榆林白皮、商南笨黑皮、陕西陇县大蒜、清涧紫皮等

内层型及外层型二次生长均可发生的品种　有金堂早、二水早、彭县蒜、蔡家坡红皮、兴平白皮、苍山大蒜、普陀大蒜、商县黑皮、白河白皮、襄樊红蒜、毕节大蒜、山西紫皮、宝鸡火蒜、呼沱大蒜等。

不发生二次生长的品种　有陕西宁强山蒜、广东新会火蒜、广东金山火蒜、广东普宁大蒜、广东韶关忠信蒜等。其中的宁强山蒜如果在播种前将种瓣用 5℃低温处理 40 天或者在鳞茎分化期至收获期给予 8h 短日照处理，均会发生内层型二次生长

大蒜产区对当地的大蒜品种或引进的外地品种，在了解其丰产性和商品性的同时，还应了解其二次生长情况，尽量选择不易发生二次生长，特别是不易发生外层型二次生长的品种

大蒜二次生长类型虽然主要取决于品种的遗传性，但不同品种间的遗传稳定性有差异。

一般只发生内层型二次生长、不发生外层型二次生长的品种及不发生二次生长的品种，遗传

性较稳定，在田间栽培条件下，在不同年份中，均可保持其遗传特性
内层型和外层型二次生长均可发生的品种，遗传性不够稳定，有时二者同时发生，有时只发生外层型二次生长或只发生内层型二次生长。
二次生长发生的严重程度则与栽培技术和气候状况有密切关系。
蒜种储藏期间的温度对二次生长有显著影响，低温有促进作用，但不同品种对低温的反应程度有差异。
蔡家坡红皮蒜对低温（0～5℃）和冷凉（14～16℃）条件的反应最敏感，外层型和内层型二次生长均大幅度增加，其中内层型二次生长的增加幅度更大。苍山大蒜对低温和冷凉条件的反应次之，外层型和内层型二次生长也有较大幅度的增加，其中外层型二次生长的增加幅度较大。
改良蒜"对低温和冷凉条件的反应较迟钝，内层型二次生长有所增加，不发生外层型二次生长的特性仍未改变。
秋播地区蒜头收获后，多在室温下储藏，储藏期间不会遇到容易诱发二次生长的低温和冷凉条件。
秋季播种后，从苗期到花芽、鳞芽分化发育期都会遇到低温和冷凉条件，所以蒜种即使不进行低温和冷凉处理，也有发生二次生长的可能，只是发生的严重程度较进行过低温和冷凉处理的要轻一些。
春播地区蒜头收获后要储藏到第二年 3～4 月播种，为了使蒜头不致受冻，储藏场所的最低温度多控制在 0℃左右，在长达 7～8 个月的储藏期间以及早春露地播种后的一段时间，都具备诱发二次生长的低温和冷凉条件。
蒜种储藏场所除温度对二次生长有影响外，空气相对湿度也有影响，而且温度与空气相对湿度之间有互作关系。
苍山大蒜于播种前 30 天在 5℃和 75%～100%空气相对湿度下储藏的蒜种，秋播后，第二年外层型二次生长指数比在 5℃和 25%～50%空气相对湿度下储藏的蒜种增加 3.3 倍，内层型二次生长指数增加 1.9 倍。而在 15℃和 25℃下储藏的蒜种，不同空气相对湿度（25%～100%）间，无论是外层型二次生长还是内层型二次生长的发生程度均无显著差异。所以，为了减少二次生长的发生，在蒜种储藏期间不但要避免低温，而且要避免 75%以上的空气相对湿度。
国内有关大蒜二次生长的报道，多认为播种期早是发生二次生长的重要原因之一
播种期与二次生长的关系因品种、蒜种休眠程度、蒜种储藏环境、播种后出苗快慢以及土壤湿度的不同而异，而且播种期早晚对同一品种的不同的二次生长类型的影响也不尽相同
在外层型和内层型二次生长均可发生的品种中，如蔡家坡红皮蒜，在陕西关中地区，较正常播种期（9 月中下旬）提早播种，同时蒜种的休眠期已结束，播种后出苗快，苗的长势强时，外层型二次生长严重发生；如果播期虽然提早，但蒜种尚在休眠状态，播种后迟迟不出苗，苗的长势弱，则早播并不会造成外层型二次生长的大发生。
容易发生内层型二次生长的品种，如苍山大蒜，在陕西关中地区，播期早晚对外层型二次生长的发生无显著影响，但在 10 月中旬以前，晚播比早播更容易发生内层型二次生长，蒜种如果经过冷凉处理而且提早播种时，则会促进外层型和内层型二次生长的发生。
播种期对二次生长的影响还和土壤湿度有关，据报道苍山大蒜的播种期和土壤湿度对内层型二次生长的发生有影响。
播期无论早晚，当土壤湿度高（土壤相对含水量为 90%）时，内层型二次生长发生株率比土壤湿度低（土壤相对含水量为 50%）的极显著增高。土壤湿度高而且播期早时，对内层型二次生长的发生更有利；播期虽然早，但土壤湿度低时，则不利于内层型二次生长的发生
大蒜属于百合科葱属植物，其基因组相对较小，大约含有 16 条染色体。研究表明，大蒜基因组中含有丰富的基因家族，如蒜氨酸酶基因家族，这些基因家族在大蒜的药用价值和风味

形成中起着重要作用。
大蒜基因组中含有多种生物活性成分的合成途径基因，如大蒜素、蒜氨酸等。这些基因参与调控大蒜的生长发育、抗病性及抗氧化能力，使得大蒜在食品和医药领域具有较高的应用价值。
大蒜的植物学分类，属于植物界，被子植物门，单子叶植物纲，百合目，百合科，葱属，蒜种，又分为多个亚种和小种。
大蒜喜凉爽怕炎热，10-12℃可以发芽，15-22℃生长发育最快最好。5℃左右停止发育，-8—-10℃可以忍耐，超过-10℃开始死亡。高温 25℃以上根系不能吸水肥，自然死亡。
大蒜是食品的一种调料，广泛用于吃食当中，具有消毒、杀菌、健肠胃等多种功能，称为四季良药大蒜生姜。
大蒜适宜的酸碱度 PH 值，应该在 6-7.5 最好。过酸或过碱，PH 低于 5 高于 8.5 不宜种植大蒜。
大蒜种植|时间，在黄淮流域以秋播为主。气温降至 20℃左右，10 日份在寒露节前后种植，翌年 5 月中下、旬收获，生长期 210-230 天左右，其它地区根据气候而定，也可以春播。
大蒜的播种及出苗期：一般大蒜从播种到齐苗应该是 7-8 天，胚芽鞘出土保护第一片叶伸出地面
大蒜喜凉爽，怕炎热，可以耐-10℃的低温不死，冬季温度-15℃以下全部冻死，不能越冬
大蒜是需要春化的作物,需要在 5-6 片叶以前终过 0 一 12℃的低温两个月左右，渡过春化，如果春化不好或不能顺利的通过春化，指种过早或过晚，容易出现独头或形成面包蒜和毛蒜，失去商品价值
全国大部分区都有大蒜种植，黑龙江、云南、西藏、新疆都能种植大蒜，但是各地都有自己的品种
大蒜喜凉爽，怕炎热，可以耐-10℃的低温不死，冬季温度-15℃以下全部冻死，不能越冬。
大蒜存放应该在 25℃从上进入休眠期，-1℃—-5℃也可以存放，最好在-1℃—-3℃冷库储存，5℃以上芽萌动
、春化阶段：大蒜发芽以后在五、六片以前，经过 0 至 12℃的低温 2 个月以上，分化成新的蒜头，形成蒜瓣，这就叫春化(同小麦一样)。没有通过春化的大蒜形成独头，春化不好的大蒜形成气包蒜或马尾蒜。主要是特定的温度、时间、蒜苗的生长期所决定的
光照阶段:大蒜完成春化以后，随着气温的升高进入光照阶段，这个时期大量生长根、茎、叶，积累营养，准备结实。这个时期，需要充足的适宜的光照，大蒜返青以后，叶片达到 18-20 片，单株根系达到 180 一 200 条，茎粗大到直径 1.5-1.8 厘米，叶片厚度可达 2 一 3 毫米，长度达到 40-60 厘米，叶面积指数达到 4 一 5 个，叶色浓绿，也是大蒜需肥零水最多的时期。3 月中下旬-4 月上中旬。
结实阶段:也叫生殖生长阶段，这个时期营养生长完成，根、茎、叶不在增加，体内积累了大量的碳水化合物，抽完蒜苔以后，全部转移到蒜头，迅速膨大，形以蒜头等茎叶全部枯黄、萎缩发黄转移完成收获。
越冬期：大雪节至冬至节进入全年最寒冷阶段，到了 12 月中下旬，到了来年三月份中下旬，大体 90 天为越冬期。
返青期：春季随气温升高，稳定在 12℃以上，开始生长，15℃以上加快，18-22℃最旺，3 月中下旬-4 月上中旬
蒜头膨大期：抽苔以后，整株大蒜的营养都回流向蒜头，这就是大蒜膨大期，4 月下旬-6 月上中旬。
抽苔期：4 月上中旬以后，随着最后一片叶的伸长，蒜苔开始生长，4 月的中下旬，蒜苔成熟，为抽苔期。

收获期：大蒜叶片由青变黄，假茎用脚踩踏，棉软不断，养分已全部回收，开始收获，5 月中下旬，大蒜的生育期没有明显的分界线，从一个生育期逐步进入另一个生育期前后重叠，只有初期、盛期和末期。
大蒜是高含水的作物，一般含量 80%以上。晾晒非常重要，稍微不注意，容易霉变腐烂。收获后最好就地晾晒 3-5 天后再装袋，放在通风的地方，不能暴晒，一般不能少于 10-15 天,基部表皮都干了以后再入库诸存。商品大蒜要根系全部切除
蒜头中的碳水化合物、蛋白质、磷、维生素 B1(硫胺素)及烟酸含量,蒜苗中的蛋白质、钾、胡萝卜素(维生素 A 原)、维生素 B1、维生素 B2(核黄素)、维生素 C(抗坏血酸)及烟酸含量,蒜薹中的蛋白质及维生素 C 含量,蒜黄中的维生素 B1 及磷的含量在大宗蔬菜中是比较高的,并含有人体必需的多种氨基酸
新鲜蒜头中微量元素硒的含量在蔬菜中是最高的,达到 0.276 微克/克,一般蔬菜的含硒量仅为 0.01 微克/克。
大蒜含有 0.2%的挥发油,内含蒜氨酸。蒜氨酸没有挥发性,也没有臭味,只有在切蒜时蒜氨酸在蒜酶的作用下才分解成有臭味的蒜辣素(大蒜素)。
大蒜是广谱杀菌素,对危害人或畜、禽的多种病原菌(如葡萄球菌、化脓性球菌、痢疾杆菌、大肠杆菌、伤寒杆菌、结核杆菌、白喉杆菌、炭疽杆菌、霍乱弧菌、脑膜炎及肺炎双球菌、链球菌等)有抑杀作用
大蒜鳞茎盘顶部的生长锥分化为花芽后,逐步发育抽生蒜薹。蒜薹的下部为花茎,顶端着生花苞称总苞。
总苞成熟后开裂,可以看到许多小的鳞茎,称气生鳞茎、空中鳞茎,俗称蒜珠或天蒜。
蒜珠的构造与蒜瓣基本相同,也可以用作播种材料。总苞中除了蒜珠以外,还有一些紫色小花,与蒜珠混生在一起。
。小花有花瓣 6 片,分两轮排列;雄蕊 6 枚,呈两轮排列;有 1 枚柱头,子房 3 室。但花的发育多不完全,一般不能形成种子。即使形成少量发育不良的种子,也难以成苗,没有利用价值
通常所称的蒜头,植物学名词是鳞茎,其构造见图 3-2。构成鳞茎的各个蒜瓣,植物学名词叫鳞芽。
。蒜头和蒜瓣的基部都有一个扁平的盘状致密组织,称茎盘。
281 茎盘与植物正常的茎不同,属于茎的变态,又称变态茎。
蒜头成熟以后,茎盘木质化,有保护蒜瓣、减少水分散失的作用,所以大蒜贮藏时要用完整的蒜头。
鳞芽是由大蒜植株叶片叶腋处的侧芽发育而成,所以又称“鳞腋芽”
鳞芽由 2~3 层鳞片和 1 个幼芽构成。外面 1~2 层鳞片起保护作用,称保护鳞片或保护叶。最内一层是贮藏养分的部分,称贮藏鳞片或贮藏叶。
在鳞茎肥大时,保护叶中的养分逐渐转运到贮藏叶中,最终形成干燥的膜,俗称蒜衣,贮藏叶则发育成肥厚的肉质食用部分。贮藏叶中包藏 1 个幼芽,称发芽叶
鳞芽发生的位置因品种而异。蒜瓣大而少、分两层排列的品种,蒜瓣多发生在花茎外围第一至第二层的叶腋中;蒜瓣小而多、呈多层排列的品种,多发生在花茎外围第一至第五层的叶腋中。
大蒜的根是从蒜瓣基部的茎盘上发生的,为弦线状须根,称为不定根,没有主根。
蒜瓣背部(外侧)的茎盘边缘发根较多,腹部(内侧)发根较少。主要根系分布在 5~25 厘米深的土层中,横向分布范围约 30 厘米,属浅根性蔬菜。须根上的根毛少,吸水力弱,所以喜湿、喜肥,不耐旱。
播种以前,蒜瓣的茎盘上已出现米粒状的根突起;播种以后,在适宜的温度和湿度条件下迅速长出新根。早发生的根随茎盘的增大而逐渐衰老、死亡,新发生的根取而代之,不断进行更新。

大蒜的全生育期有 2 次发根高峰:第一次在发芽期,发根数为 20~30 条;第二次在“退母”后,发根数为 50~80 条。1 棵成龄植株的发根数为 100 条左右。蒜薹采收以后,根系不再增长,并逐渐衰亡。
播种用的蒜瓣中除肉质贮藏叶和发芽叶外,到播种时一般还已分化出 2~3 片普通幼叶
播种后,最先长出的 1 片叶,只有叶鞘,没有叶身,为发芽叶,称初生叶。以后陆续长出普通叶,既有叶鞘,又有叶身。在新叶生长的同时,生长锥继续分化叶片,叶片数逐渐增加。待生长锥分化花芽后,叶片的分化结束,叶片数不再增加。最终的叶片数因品种而异。
大蒜的叶片包括叶鞘和叶身两部分。叶鞘呈圆筒形,着生在茎盘上。每一片叶均由先发生的前一片叶的出叶口伸出,许多层叶鞘套在一起,形成直立的圆柱形茎秆,由于它不是真正的茎,故称“假茎”
叶与叶之间的叶鞘长度随叶位的升高而增加。一般在花茎伸出最后一片叶的叶鞘口以后,叶鞘停止生长。
叶鞘的长短和出叶口的粗细,与抽取蒜薹的难易有关。叶鞘越长、出叶口越细的品种,蒜薹越难抽出。
大蒜的叶片互生,对称排列。叶片的排列方向与蒜瓣的背腹连线垂直。
大蒜叶片的颜色多为绿色至深绿色。叶面一般有白粉。叶片绿色的深浅,叶片的长度和宽度,叶片质地的软硬与白色蜡粉的多少,叶鞘的长短和粗细,叶片数目的多少以及叶片的开张程度等,都与品种有关。
大蒜从用蒜瓣播种到收获蒜头,重新获得新的蒜瓣,进入休眠状态称为一个生育周期。
大蒜完成一个生育周期需要顺序经历出苗期、幼苗期、花芽及鳞芽分化期、花茎伸长期、鳞茎肥大期和休眠期
在栽培上,一般将播种至收获蒜头所需天数称为生育期。生育期的长短因地区和栽培季节的不同而有很大差异。
由于不论春播或秋播,鳞茎都是在温度升高、日照加长的条件下成熟,在同一地区,即使播期不同,但蒜头成熟期基本相同。所以,播种过晚时大蒜生育期缩短,产量必然降低。
春播地区的大蒜,从播种到收获蒜头,在 1 年以内完成,需 100~120 天;秋播地区的大蒜,播种后要经过一段较长的越冬期,到翌年才收获蒜头,生育期长达 210~270 天。即使在同一地区,由于品种的熟性不同,生育期也有差异。
在出苗期中,蒜瓣基部的茎盘上发生弦线状须根,蒜瓣发芽叶中的生长锥继续分化新的幼叶。
在出苗期中发根和长叶所需的养分主要来源于蒜瓣中贮藏的养分,所以播种所用蒜瓣的大小对大蒜以后的生长发育状况有很大影响。
从初生叶展开到生长锥停止分化幼叶而分化为花芽、总叶片数不再增加时,为幼苗期。
幼苗期大蒜生长主要依靠种瓣中贮藏的养分。
在幼苗期内,根系继续扩展,生长锥继续分化幼叶,幼叶逐渐长大,露出土面,进行光合作用,制造养分。
从花芽和鳞芽分化开始到分化结束为花芽和鳞芽分化期。
。花芽和鳞芽的开始分化期,从蒜苗的外观难以判断,要用双面剃须刀片从蒜苗基部向上纵切成薄片,放在 80~100 倍的显微镜下才能观察到。花芽和鳞芽分化期结束将蒜苗的叶鞘由外向内一层一层地剥去,就可以观察到。大蒜花芽和鳞芽的分化过程
从花芽分化结束到蒜薹采收为花茎伸长期。
在花茎伸长期内,叶片全部展开,叶面积迅速增长,花茎生长加快,鳞芽逐渐增大,是大蒜植株生长量最大的时期,也是生产上水、肥管理的关键时期。
从鳞芽分化结束到鳞茎成熟为鳞茎肥大期。
在鳞茎肥大期内,当鳞芽分化结束后,因花茎生长占优势,鳞芽生长缓慢;花茎伸长后期,鳞芽生

长开始加快;花茎采摘后,叶片中的养分集中运输到鳞芽中,鳞芽重量迅速增加,直至鳞茎成熟前 1 周左右,增长速度逐渐减慢,所以及时采收蒜薹有助于鳞茎的肥大。
鳞茎肥大后期,根系大量死亡,叶片枯黄,叶鞘中的养分由于向鳞芽中转移而变薄变软,乃至倒伏。包围鳞芽的数层叶鞘变成薄膜状,以保护鳞芽,防止失水干燥
大蒜鳞茎成熟后即进入休眠状态,这时即使给予适宜的温度和水分,鳞芽也不会萌芽和发根,这段时间称为大蒜的生理休眠期
通过生理休眠期以后,在适宜的温、湿度条件下就可以萌芽和发根。如果人为控制其萌发条件,也可使鳞芽继续休眠,称强迫休眠。
大蒜是喜好冷凉气候的蔬菜,通过休眠期的蒜瓣,在 3℃~5℃的低温下便可开始萌发。
大蒜花芽和鳞芽的分化都需要低温,在蒜头贮藏期间或栽种以后,经受 10℃以下的低温 1~2 个月,除了很小的蒜瓣外,都可以分化花芽和鳞芽。
花茎和鳞茎发育除了受温度的影响外,还与光照时间的长短有关。不同生态型品种对光照时间长短的反应不完全相同。
大蒜的根系浅,根毛少,吸水范围较小,所以不耐旱,但不同生育期对土壤湿度的要求有差异。
大蒜的幼苗期要适当降低土壤湿度,防止苗子徒长,促进根系向纵深发展,避免蒜种因土壤过湿而提早腐烂,对幼苗生长造成不利影响。
花茎伸长和鳞茎膨大期是大蒜生长日趋旺盛的时期,要求较高的土壤湿度。
当鳞茎充分膨大,大蒜根系逐渐变黄枯萎,鳞茎外面数层叶鞘逐渐失去水分变成膜状时,应降低土壤湿度,防止鳞茎外皮
大蒜叶片呈带状,较厚,表面积小,尤其是叶表面有蜡粉等保护组织,地上部具有耐旱的特征。
大蒜对富含腐殖质的有机肥反应良好,增产效果显著。全生育期吸收的氮最多,钾次之,磷最少。
大蒜的遗传特性之一是其基因组中存在大量的基因家族，这些家族成员在功能上具有多样性，决定了大蒜在生长、发育和抗逆性方面的复杂调控机制。
大蒜的抗病性遗传特性表现在其基因组中含有一系列抗病相关基因，这些基因通过不同的途径增强大蒜对病原菌的抵抗力。
耐旱性是大蒜的重要遗传特性，相关基因通过调节细胞膜的稳定性和渗透调节物质的合成，帮助大蒜在干旱环境下生存。
大蒜的鳞茎大小和形状受到多个遗传因素的影响，这些基因通过调控细胞分裂和伸长，共同决定了鳞茎的最终形态。
大蒜的遗传多样性体现在其丰富的表型变异，如鳞茎颜色、叶片形态和花器官结构等，这些变异为大蒜的遗传改良提供了宝贵资源。
大蒜的繁殖方式为无性繁殖，通过鳞茎分株进行，这种繁殖方式保证了遗传信息的稳定传递，降低了基因突变的可能性。
大蒜基因组中的重复序列含量较高，这些重复序列在基因组结构和功能上发挥着重要作用，影响大蒜的遗传稳定性和进化。
大蒜的耐寒性遗传特性表现在其能够通过调节细胞膜的流动性、增加抗冻蛋白含量等方式，适应低温环境。
大蒜的遗传特性还包括其代谢途径的多样性，这些代谢途径受基因调控，决定了大蒜中活性成分的含量和种类。
大蒜的叶片形态遗传特性表现在其叶片大小、形状和排列方式上，这些特征有助于提高光合效率和适应不同环境条件。
大蒜的花器官发育受遗传调控，包括花色、花型和开花时间等，这些特征对大蒜的繁殖和传粉具有重要影响。
大蒜基因组中含有多种抗逆境基因，这些基因通过调节抗氧化酶活性、渗透调节物质合成等

途径，提高大蒜的抗逆能力。
大蒜的产量性状受多个基因控制，包括鳞茎大小、重量和产量等，这些基因的遗传改良对提高大蒜产量具有重要意义。
大蒜的遗传特性还包括其基因组中的基因表达调控机制，这些机制确保了大蒜在不同生长发育阶段和环境条件下的基因表达平衡。
大蒜基因组中的抗虫基因通过调控次生代谢产物的合成，提高大蒜对害虫的防御能力，降低害虫对大蒜的危害。
大蒜的遗传特性体现在其具有独特的蒜素合成途径，这些途径中的关键基因决定了大蒜特有的辛辣味和健康功效。
大蒜的遗传多样性使得不同品种在耐盐性上存在差异，这些差异由基因组的盐敏感性相关基因所控制。
大蒜的遗传特性还包括其能够通过基因调控，在土壤中形成抗菌圈，抑制病原菌的生长，保护植株健康。
大蒜的休眠和萌发过程受遗传控制，相关基因决定了鳞茎在特定条件下的休眠深度和萌发速度。
大蒜的遗传特性表现在其能够通过基因调控，积累大量的硫化合物，这些化合物对大蒜的风味和保健功能至关重要。
大蒜的遗传特性还体现在其基因组中的抗老化基因，这些基因有助于保持大蒜在储存过程中的品质和营养价值。
大蒜的遗传特性包括其基因组中的光周期敏感性基因，这些基因影响大蒜的开花时间和地理适应性。
大蒜的遗传特性表现在其能够通过基因调控，产生多种次生代谢产物，这些产物对提高大蒜的药用价值具有重要意义。
大蒜的遗传特性还包括其基因组中的非编码 RNA，这些 RNA 在基因表达调控和生长发育过程中发挥重要作用。
大蒜的遗传特性体现在其能够通过基因调控，适应不同的土壤类型和 pH 值，表现出较强的土壤适应性。
大蒜的遗传特性还包括其基因组中的抗重金属基因，这些基因帮助大蒜在重金属污染的土壤中生长。
大蒜的遗传特性表现在其基因组中的抗紫外线基因，这些基因保护大蒜免受紫外线辐射的伤害。
大蒜的遗传特性还包括其基因组中的抗病基因多样性，这些基因通过不同的机制抵抗多种病原菌的侵染。
大蒜的遗传特性体现在其能够通过基因调控，影响根际微生物群落结构，从而改善土壤环境和促进植物生长。
大蒜的遗传特性还包括其基因组中的抗寒基因，这些基因通过调节细胞膜的脂肪酸组成，增强大蒜的耐寒性。
大蒜的遗传特性表现在其基因组中的抗热基因，这些基因帮助大蒜在高温环境下维持正常的生理功能。
大蒜的遗传特性还包括其基因组中的抗盐基因，这些基因通过调节离子平衡和渗透调节物质的合成，提高大蒜的耐盐性。
大蒜的遗传特性体现在其基因组中的抗干旱基因，这些基因通过调节水分利用效率和气孔运动，帮助大蒜适应干旱环境。
大蒜的遗传特性还包括其基因组中的抗病相关转录因子，这些转录因子在调控大蒜抗病反应

中起到关键作用。
大蒜的遗传特性表现在其基因组中的基因表达可塑性,这种可塑性使得大蒜能够适应多变的环境条件
大蒜的遗传特性包括其基因组中的多个基因位点参与调控蒜瓣的数量和排列,这些位点对大蒜的商品性状有显著影响。
大蒜的遗传特性体现在其基因组中存在一系列与光合作用效率相关的基因,这些基因优化了光合作用过程,提高了大蒜的生长速度。
大蒜的遗传特性还包括其基因组中的抗病基因簇,这些基因簇通过协同作用,增强了大蒜对多种病原体的抵抗力。
大蒜的遗传特性表现在其基因组中的耐储藏基因,这些基因通过减少代谢活动,延长大蒜鳞茎的储存时间。
大蒜的遗传特性还包括其基因组中的逆境响应基因,这些基因使得大蒜能够在极端环境下维持基本的生理功能。
大蒜的遗传特性体现在其基因组中的基因变异,这些变异导致了大蒜在形态、风味和抗逆性方面的多样性。
大蒜的遗传特性还包括其基因组中的甲基化模式,这些模式在基因表达调控和表观遗传变异中起着重要作用。
大蒜的遗传特性表现在其基因组中的激素信号途径相关基因,这些基因调控着大蒜的生长发育和应激响应。
大蒜的遗传特性还包括其基因组中的细胞周期调控基因,这些基因影响着大蒜细胞的分裂和增殖。
大蒜的遗传特性体现在其基因组中的基因重组事件,这些事件为大蒜的遗传改良提供了丰富的遗传资源。
大蒜的遗传特性还包括其基因组中的转运蛋白基因,这些基因在大蒜吸收和运输营养物质过程中发挥关键作用。
大蒜的遗传特性表现在其基因组中的抗虫基因,这些基因通过产生驱虫或毒杀作用,保护大蒜免受害虫侵害。
大蒜的遗传特性还包括其基因组中的耐低温基因,这些基因帮助大蒜在低温环境下保持生长活力。
大蒜的遗传特性体现在其基因组中的耐高温基因,这些基因使得大蒜能够在高温季节保持正常的生长状态。
大蒜的遗传特性还包括其基因组中的耐盐基因,这些基因通过调节细胞内离子平衡,帮助大蒜适应盐碱土壤。
大蒜的遗传特性表现在其基因组中的耐旱基因,这些基因通过改善水分利用效率,增强大蒜的耐旱能力。
大蒜的遗传特性还包括其基因组中的抗病相关蛋白编码基因,这些基因通过直接或间接作用,提高大蒜的抗病性。
大蒜的遗传特性体现在其基因组中的次生代谢途径相关基因,这些基因参与合成大蒜中的活性成分,如蒜素。
大蒜的遗传特性还包括其基因组中的基因表达节律性,这种节律性确保了大蒜在昼夜交替环境中的正常生长。
大蒜的遗传特性表现在其基因组中的基因多态性,这种多态性为大蒜的品种改良和抗性育种提供了丰富的遗传材料
大蒜的遗传特性体现在其基因组中的转录因子基因,这些基因通过调控下游基因的表达,参

与大蒜的生长发育和逆境响应。
大蒜的遗传特性还包括其基因组中的 microRNA 编码基因，这些小分子 RNA 在调控基因表达和生长发育中扮演重要角色。
大蒜的遗传特性表现在其基因组中的 DNA 修复基因，这些基因帮助大蒜在遭受 DNA 损伤时进行修复，维持基因组的稳定性。
大蒜的遗传特性还包括其基因组中的糖代谢相关基因, 这些基因调控着大蒜的糖类合成与分解，影响其能量供应。
大蒜的遗传特性体现在其基因组中的脂肪酸合成基因, 这些基因参与大蒜不饱和脂肪酸的合成，影响其品质和营养价值。
大蒜的遗传特性还包括其基因组中的植物激素合成基因，如生长素、赤霉素等，这些激素在调控大蒜生长发育中起关键作用。
大蒜的遗传特性表现在其基因组中的抗逆相关蛋白编码基因, 这些蛋白帮助大蒜抵御生物和非生物胁迫。
大蒜的遗传特性还包括其基因组中的质体基因, 这些基因在光合作用和质体发育中发挥重要作用。
大蒜的遗传特性体现在其基因组中的细胞壁合成相关基因, 这些基因影响着大蒜细胞的结构和强度。
大蒜的遗传特性还包括其基因组中的离子通道和载体蛋白基因, 这些基因在离子运输和信号转导中起到关键作用。
大蒜的遗传特性表现在其基因组中的氮代谢相关基因, 这些基因调控着大蒜对氮素的吸收和利用效率。
大蒜的遗传特性还包括其基因组中的硫代谢相关基因, 这些基因参与大蒜中硫代葡萄糖苷的合成，影响其风味和营养。
大蒜的遗传特性体现在其基因组中的抗病基因的保守性和多样性, 这些基因使得大蒜能够抵抗多种病原菌的侵染。
大蒜的遗传特性还包括其基因组中的抗虫基因的特异性, 这些基因使得大蒜能够针对特定害虫产生防御反应。
大蒜的遗传特性表现在其基因组中的耐盐基因的调控网络, 这些网络确保大蒜在盐胁迫下能够维持正常的生理功能。
大蒜的遗传特性还包括其基因组中的耐旱基因的表达模式, 这些模式使得大蒜能够在干旱条件下调整生长策略。
大蒜的遗传特性体现在其基因组中的产量相关基因的复杂性, 这些基因共同作用决定了大蒜的产量潜力。
大蒜的遗传特性还包括其基因组中的品质相关基因的多态性, 这些基因影响着大蒜的风味和营养价值。
大蒜的遗传特性表现在其基因组中的环境适应性基因的广泛性, 这些基因使得大蒜能够适应不同的地理和气候条件。
大蒜的遗传特性还包括其基因组中的基因家族的扩展性, 这些家族的成员在进化过程中产生了新的功能。
大蒜的遗传特性体现在其基因组中的抗病基因的多样性, 这些基因通过不同的机制提供对多种病原体的防御，从而增强大蒜的整体抗病能力。
大蒜的遗传特性还包括其基因组中的耐寒性基因, 这些基因通过调节细胞膜的流动性及增加抗冻蛋白的表达，帮助大蒜在低温环境中生存。
大蒜的遗传特性表现在其基因组中的耐热性基因，这些基因通过提高热休克蛋白的表达，保

护细胞免受高温伤害。
大蒜的遗传特性还包括其基因组中的根际微生物互作基因，这些基因影响大蒜与土壤微生物的相互作用，进而影响营养吸收和生长。
大蒜的遗传特性体现在其基因组中的次生代谢途径调控基因，这些基因参与合成大蒜中的多种生物活性物质，如蒜素和黄酮类化合物。
大蒜的遗传特性还包括其基因组中的基因表达调控网络，这些网络精细调节大蒜的生长周期和应对环境变化。
大蒜的遗传特性表现在其基因组中的遗传变异，这些变异为大蒜的品种改良和适应不同生态环境提供了丰富的遗传资源。
大蒜的遗传特性还包括其基因组中的抗虫基因，这些基因通过产生驱虫或毒杀作用，保护大蒜免受害虫侵害。
大蒜的遗传特性体现在其基因组中的耐盐性基因，这些基因通过调节离子平衡和渗透调节物质的合成，提高大蒜在盐碱地的生存能力。
大蒜的遗传特性还包括其基因组中的光合作用相关基因，这些基因通过优化光合效率，促进大蒜的生长和产量。
大蒜的遗传特性表现在其基因组中的抗逆性基因，这些基因帮助大蒜在面临生物和非生物胁迫时维持正常的生理功能。
大蒜的遗传特性还包括其基因组中的基因家族的演化，这些家族的成员在进化过程中可能获得了新的功能，增强了大蒜的适应性。
大蒜的遗传特性体现在其基因组中的基因多效性，即一个基因可能影响多个性状，这种现象在大蒜的遗传改良中具有重要意义。
大蒜的遗传特性还包括其基因组中的非编码 RNA，如长非编码 RNA 和环状 RNA，这些 RNA 在基因表达调控中扮演着重要角色。
大蒜的遗传特性表现在其基因组中的基因连锁现象，这些连锁基因在遗传过程中一起传递，影响大蒜的某些复合性状。
大蒜的遗传特性还包括其基因组中的基因突变，这些突变可能是自然选择或人工选择的基础，为大蒜的品种改良提供了可能性。
大蒜的遗传特性体现在其基因组中的基因复制事件，这些事件可能导致基因功能的分化，为大蒜的适应性和多样性提供了基础。
大蒜的遗传特性还包括其基因组中的同源重组频率，这种重组有助于产生新的基因组合，增加大蒜的遗传变异性。
大蒜的遗传特性表现在其基因组中的小 RNA 途径，这些小 RNA 分子在转录后水平上调控基因表达，影响大蒜的生长和发育。
大蒜的遗传特性还包括其基因组中的表观遗传修饰，如 DNA 甲基化和组蛋白修饰，这些修饰在不改变 DNA 序列的情况下调控基因活性。
大蒜的遗传特性体现在其基因组中的基因插入和缺失变异（INDELs），这些变异是造成大蒜品种间差异的重要因素。
大蒜的遗传特性还包括其基因组中的基因转换事件，这种事件在基因家族内部传递基因片段，影响大蒜的遗传多样性。
大蒜的遗传特性表现在其基因组中的基因表达量的差异，这些差异决定了大蒜在不同环境条件下的生理反应和适应性。
大蒜的遗传特性还包括其基因组中的跨物种基因转移事件，这些事件可能引入新的功能基因，增强大蒜的环境适应能力。
大蒜的遗传特性体现在其基因组中的基因网络调控，这些网络协调多个基因的表达，确保大

蒜在复杂环境中的稳定生长和繁殖。
大蒜基因组中含有多种生物活性成分的合成基因，这些基因决定了大蒜的药用价值和保健功能，如蒜素、大蒜素等。
大蒜的基因序列显示，它与同属百合科的洋葱和韭菜有较高的基因同源性，但同时也具有独特的遗传特征。
大蒜基因组中的抗病相关基因，如 R 基因家族，帮助大蒜抵御多种病原菌的侵害，维持其健康生长。
大蒜的基因组研究揭示了其在进化过程中形成了复杂的抗逆性基因网络，这些基因有助于适应不同的环境压力。
大蒜基因组中的代谢途径相关基因，如萜类化合物合成基因，对大蒜的风味和营养价值具有重要影响。
大蒜的基因表达模式在地下鳞茎形成和发育过程中表现出特定的时空特异性，这些基因调控着大蒜的生长和产量。
大蒜基因组中的转录因子基因家族，如 MYB、WRKY 等，参与调控大蒜的生长发育和应对环境胁迫。
大蒜的基因组中含有多个与植物激素信号传导相关的基因，这些基因在调节大蒜生长发育中起着关键作用。
大蒜基因组中的耐盐基因，如 SOS 途径相关基因，使其能够在盐碱地环境中维持正常的生理功能。
大蒜基因组中的耐旱基因，如 LEA 蛋白基因，帮助其在干旱条件下保持细胞膜的稳定性，减少水分流失。
大蒜的基因多样性研究表明，不同品种的大蒜在基因水平上存在显著差异，这些差异影响了它们的表型和适应性。
大蒜基因组中的光合作用相关基因，如 Rubisco 亚基基因，对提高光合效率和生长速度具有重要作用。
大蒜基因组中的抗虫基因，如凝集素基因，能够提高大蒜对害虫的抵抗力，减少农药使用。
大蒜的基因组中含有多个与细胞周期调控相关的基因，这些基因在维持大蒜细胞分裂和生长中起着关键作用。
大蒜基因组中的糖代谢相关基因，如蔗糖合成酶基因，对大蒜的能量供应和生长发育具有重要影响。
大蒜基因组中的非编码 RNA 基因，如 microRNA 和 longnon-codingRNA，参与调控基因表达，影响大蒜的生长发育和逆境响应。
大蒜的遗传特性表现在其基因组中存在多个与抗病性相关的基因簇，这些基因簇通过协同作用提高大蒜对病原菌的抵抗力。
大蒜基因组中的基因变异，如单核苷酸多态性（SNPs），为大蒜的遗传多样性和品种改良提供了丰富的资源。
大蒜的遗传特性体现在其基因组中的基因家族扩张，尤其是与次生代谢相关的基因家族，这增加了大蒜的化学多样性。
大蒜基因组中的逆境响应基因，如低温响应基因，通过调节细胞膜的流动性和保护蛋白的合成，帮助大蒜抵御寒冷。
大蒜的遗传特性包括其基因组中的基因表达调控网络，这些网络确保了大蒜在环境变化时的适应性。
大蒜基因组中的激素信号转导基因，如赤霉素和乙烯信号途径相关基因，对大蒜的生长调节起着重要作用。

大蒜的遗传特性表现在其基因组中的基因互作网络，这些网络中的基因共同作用，影响大蒜的产量和品质。
大蒜基因组中的 DNA 甲基化模式，这种表观遗传修饰调控基因表达，影响大蒜的生长发育和逆境响应。
大蒜的遗传特性体现在其基因组中的基因重组事件，这些事件为大蒜的遗传变异和进化提供了动力。
大蒜基因组中的基因插入和缺失变异（INDELs），这些变异对大蒜的遗传多样性和品种形成具有重要影响。
大蒜的遗传特性表现在其基因组中的基因拷贝数变异，这些变异可能导致基因功能的改变，影响大蒜的表型。
大蒜基因组中的转运蛋白基因，如离子转运蛋白和氨基酸转运蛋白，对大蒜的营养吸收和逆境响应至关重要。
大蒜的遗传特性体现在其基因组中的基因沉默机制，如 RNA 干扰途径，这些机制在基因表达调控中发挥作用。
大蒜基因组中的蛋白质降解相关基因，如泛素-蛋白酶体途径相关基因，对维持蛋白质稳态和细胞功能至关重要。
大蒜的遗传特性表现在其基因组中的基因启动子和终止子的多样性，这些调控元件影响基因的表达水平。
大蒜基因组中的转录后修饰基因，如磷酸化修饰相关基因，对大蒜的信号传导和基因表达调控具有重要作用。
大蒜的遗传特性体现在其基因组中的基因上下游调控序列，这些序列的变异可能导致基因表达模式的改变。
大蒜基因组中的端粒和端粒酶相关基因，这些基因在维持染色体稳定性和细胞寿命方面发挥作用。
大蒜的遗传特性表现在其基因组中的基因连锁不平衡现象，这种现象在遗传研究中用于追踪基因的传递和关联分析。
大蒜基因组中的逆境相关转录因子基因，如 DREB/CBF 家族基因，它们在调控大蒜的耐旱、耐寒等逆境适应性中扮演关键角色，增强了植株的抗逆能力。
大蒜的遗传特性体现在其基因组中的基因内含子和外显子结构，这些结构的差异可能导致不同的剪接变异，影响蛋白质的结构和功能。
大蒜基因组中的植物激素合成基因，如生长素、细胞分裂素和脱落酸合成相关基因，这些激素的平衡对大蒜的生长发育至关重要。
大蒜的遗传特性表现在其基因组中的基因重组事件，这些事件在进化过程中产生了新的基因组合，增加了大蒜的遗传多样性。
大蒜基因组中的抗真菌基因，如几丁质酶和β-1,3-葡聚糖酶基因，它们通过破坏真菌细胞壁来提高大蒜的抗病性。
大蒜的遗传特性体现在其基因组中的基因家族收缩现象，这可能导致某些基因功能的丧失或特化。
大蒜基因组中的光合作用相关基因，如 PSII 反应中心蛋白基因，它们对提高光合效率和能量转化至关重要。
大蒜的遗传特性表现在其基因组中的基因表达量的时空特异性，这种特异性确保了大蒜在不同生长阶段和环境下的正常生理活动。
大蒜基因组中的蛋白质磷酸化相关基因，这些基因通过调节蛋白质活性，参与信号传导和细胞代谢过程。

大蒜的遗传特性体现在其基因组中的基因上下游调控序列的保守性，这些序列对维持基因的正常表达至关重要。
大蒜基因组中的小分子代谢途径相关基因，如硫代葡萄糖苷代谢途径，这些途径的产物对大蒜的风味和健康益处有显著影响。
大蒜的遗传特性表现在其基因组中的基因突变率，这些突变是进化的驱动力，为大蒜的适应性提供了基础。
大蒜基因组中的 RNA 编辑相关基因，这种编辑机制可以改变 RNA 序列，从而影响蛋白质的合成和功能。
大蒜的遗传特性体现在其基因组中的基因复制和丢失事件，这些事件在进化过程中塑造了大蒜的基因组结构。
大蒜基因组中的细胞壁合成相关基因，如纤维素合酶和果胶甲基转移酶基因，它们对维持细胞壁的结构和功能至关重要。
大蒜的遗传特性表现在其基因组中的基因多效性，即一个基因可能影响多个性状，这种现象在遗传育种中具有重要意义。
大蒜基因组中的质体基因，这些基因在植物的能量生产和代谢中起着关键作用，并且可能在质体遗传中发生水平基因转移。
大蒜的遗传特性体现在其基因组中的基因表达噪声，这种噪声可能导致同一基因在不同细胞或环境中的表达差异。
大蒜基因组中的分子伴侣蛋白基因，如 HSP 家族基因，它们在应对环境胁迫时保护蛋白质不受损伤。
大蒜的遗传特性表现在其基因组中的基因启动子区域的多样性，这些区域的变化可能导致基因在不同组织或环境中的表达差异。
大蒜基因组中的抗虫基因，如蛋白酶抑制剂基因，它们能够干扰害虫的消化系统，提高大蒜的抗虫性。
大蒜的遗传特性体现在其基因组中的基因上游调控序列的保守性，这些序列对维持基因的正常功能至关重要。
大蒜基因组中的次生代谢途径相关基因，如蒜素合成酶基因，这些基因决定了大蒜的独特风味和健康功效。
大蒜的遗传特性表现在其基因组中的基因内含子的长度和多态性，这些内含子可能含有调节基因表达的顺式作用元件。
大蒜基因组中的 DNA 甲基化相关基因，这些基因通过表观遗传调控影响基因表达和发育过程。
大蒜的遗传特性体现在其基因组中的基因连锁不平衡区块的大小和分布，这些区块在遗传研究中用于分析种群结构和进化历史。
大蒜基因组中的信号转导相关基因，如 G 蛋白偶联受体基因，它们在感知外部信号并传递到细胞内部过程中发挥作用。
大蒜的遗传特性表现在其基因组中的基因家族的保守性和进化速率，这些特征反映了基因的功能重要性和进化压力。
大蒜基因组中的激素信号转导途径相关基因，如细胞分裂素响应因子基因，它们在调控细胞分裂和生长中起关键作用。
大蒜的遗传特性体现在其基因组中的基因表达模式的组织特异性，这种特异性确保了不同组织中特定基因的功能。
大蒜基因组中的氮代谢相关基因，如硝酸还原酶和谷氨酰胺合成酶基因，它们在氮素利用和同化过程中至关重要。

大蒜的遗传特性表现在其基因组中的基因拷贝数变异（CNVs），这些变异可能导致基因功能的改变或新功能的产生。
大蒜基因组中的碳水化合物代谢相关基因，如蔗糖合成酶和淀粉合成酶基因，它们在能量储存和运输中发挥作用。
大蒜的遗传特性体现在其基因组中的基因剪接变异，这些变异增加了蛋白质的多样性。
大蒜基因组中的抗病毒基因，如 RNA 沉默相关基因，它们通过沉默病毒基因来防御病毒感染。
大蒜的遗传特性表现在其基因组中的基因上游和下游调控网络的复杂性，这些网络协调基因表达以响应环境变化。
大蒜基因组中的脂肪酸代谢相关基因，如脂肪酸合酶基因，它们在合成细胞膜结构和储存能量中起作用。
大蒜的遗传特性体现在其基因组中的基因表达量的环境依赖性，这种依赖性确保了大蒜能够适应不同的生长条件。
大蒜基因组中的转录终止和加工相关基因，如 RNA 聚合酶和核糖核酸酶基因，它们在基因表达的最后阶段发挥作用。
大蒜的遗传特性表现在其基因组中的基因启动子的环境响应性，这些启动子能够感知环境信号并调节基因表达。
大蒜基因组中的抗真菌基因，如几丁质酶和β-1,3-葡聚糖酶基因，它们通过破坏真菌细胞壁来提供防御机制。
大蒜的遗传特性体现在其基因组中的基因选择性剪接，这种剪接方式增加了蛋白质的多样性，从而适应不同的生理功能。
大蒜基因组中的光合作用相关基因，如 Rubisco 活化酶基因，它们在碳固定过程中起着关键作用。
大蒜的遗传特性表现在其基因组中的基因家族的扩张和收缩，这些变化是适应环境变化的重要途径。
大蒜基因组中的植物抗毒素合成基因，如蒜氨酸合成酶基因，它们参与产生植物自身的防御物质。
大蒜的遗传特性体现在其基因组中的基因表达的时间特异性，即基因在不同生长发育阶段的表达模式。
大蒜基因组中的糖转运蛋白基因，它们在调节植物细胞内外糖分运输中发挥作用。
大蒜的遗传特性表现在其基因组中的基因表达的组织特异性，即基因在不同组织中的表达差异。
大蒜基因组中的激素受体基因，如生长素受体基因，它们在感知和响应激素信号中起重要作用。
大蒜的遗传特性体现在其基因组中的基因拷贝数变异（CNVs）的频率，这些变异是遗传多样性的重要来源。
大蒜基因组中的次级代谢产物合成基因，如黄酮类化合物合成基因，它们参与植物防御和吸引传粉者的过程。
大蒜的遗传特性体现在其基因组中的基因上下游序列的保守性，这些序列对基因的正确表达至关重要。
大蒜基因组中的蛋白质降解相关基因，如泛素和蛋白酶体亚单位基因，它们在蛋白质质量控制中发挥作用。
大蒜的遗传特性表现在其基因组中的基因表达的空间特异性，即基因在不同器官中的表达差异。

大蒜基因组中的离子通道和转运蛋白基因，如钾离子通道基因，它们在调节细胞内离子平衡中起关键作用。
大蒜的遗传特性体现在其基因组中的基因重组事件的频率，这些事件有助于产生新的遗传组合。
大蒜基因组中的逆境响应转录因子基因，如 NAC 家族基因，它们在植物对干旱和盐胁迫的响应中起作用。
大蒜的遗传特性表现在其基因组中的基因启动子的甲基化状态，这种甲基化可以影响基因的表达。
大蒜基因组中的脂肪酸去饱和酶基因，它们在调节膜流动性和温度适应性中发挥作用。
大蒜的遗传特性体现在其基因组中的基因内含子的剪接效率，这种效率影响成熟 mRNA 的形成。
大蒜基因组中的维生素合成相关基因，如维生素 C 合成酶基因，它们在维持植物健康和人类营养中起作用。
大蒜的遗传特性表现在其基因组中的基因表达对光的响应性，即光调控基因的表达模式。
大蒜基因组中的蛋白质磷酸化相关基因，如蛋白激酶和磷酸酶基因，它们在信号转导中起关键作用。
大蒜的遗传特性体现在其基因组中的基因表达对温度的响应性，即温度调控基因的表达模式。
大蒜基因组中的抗逆相关基因，如 LEA 蛋白基因，它们在植物对干旱和低温胁迫的适应中起作用。
每生产 1000 公斤大蒜头,需要吸收纯氮 15 公斤左右，需要吸收五氧化二磷 4.5-5.5 公斤,需要吸收氧化钾 12 公斤左石;
当施入土壤 15-20 厘米深的肥料，大蒜利用率氮肥 25-30%，磷肥 10-15%，钾肥 30-35%;
大蒜的发芽期不需要吸收肥料，母体营养可生长五片叶左右;
大蒜的苗期，指越冬前需总肥量的 8-12%左石;
大蒜的越冬期基本不吸收肥迷，关键是促进内部分化;
大蒜的返青期，根茎叶开始大量生长，三月下旬 4 月上旬，需总肥量 30-40%;
大蒜的青盛期至抽苔，大蒜生长量最大内外分化期，需总肥量 30%-45%;
大蒜的抽苔后期，根茎叶停止生长，蒜头膨大期需总肥量 30%左右
蒜根茎叶的生长是以氮肥为主的生长，磷钾肥辅助作用。大蒜的蒜头膨大期是以钾为主的生长，氮磷辅助生长。
大蒜的需肥前期和中期以氮素化肥为主，开花坐果开始转向以磷肥为主，蒜头膨大期逐步转向以钾元素为主的生长发育。
大蒜的发芽水，大蒜播种后一段都要浇一次水。
大蒜的越冬期，因为温度低，大蒜不能生长，该浇一次水保护安全越冬;
大蒜返青后，气温升高消耗量大必须浇一次返青水;
大蒜开始抽苔，水肥需要量大，应该水肥齐攻，保证生长;
大蒜抽苔后，蒜头急剧膨大，生长迅速，必须浇一通透水。
大蒜的苗期，返春初期需要的是温度，要适当腔制含水。
大蒜的成熟期，要适当晾地，一般不能浇水。
大蒜的中后期，一般浇水要配比一定含量的肥料，随水带肥;
大蒜的施肥必须施用水溶性肥料，全水溶无残渣，利用率显著提高，速溶性肥料最好，效果快;
大蒜的施肥前中期以氮肥为主，中偏后期以磷肥为主，后期膨大以钾肥为主;

大蒜的应浇水均匀一致，存水多的地方，肥料也多，容易伤苗，避免水量过大
大蒜 10-12℃可以发芽，15-22℃生长发育最快最好。5℃左右停止发育，-8—-10℃可以忍耐，超过-10℃开始死亡。高温 25℃以上根系不能吸水肥，自然死亡。
大蒜在黄淮流域北纬 33-36 度较为适宜种植，超过 38 度以上，冬季最低温超过-15℃能全部冻死，只能春播。
大蒜适宜的酸碱度 PH 值，应该在 6-7.5 最好。过酸或过碱，PH 低于 5 高于 8.5 不宜种植大蒜。
大蒜蒜头为主的每亩种植应该在 3.1-3.5 万株，可以确保亩产在 3000-3500 斤以上。
大蒜种植|时间，在黄淮流域以秋播为主。气温降至 20℃左右，10 日份在寒露节前后种植，翌年 5 月中下、旬收获，生长期 210-230 天左右，其它地区根据气候而定，也可以春播。
大蒜的需肥，一般每生产 1000 公斤大蒜头，需要吸收纯氮 28-29 斤，五氧化磷 9-11 斤，氧化钾 26-27 斤，大蒜种植应该以施足底肥为主，每亩施氮磷钾的总含量 120-140 斤为宜。
大蒜种子一般要选择适合当地种植的品种
大蒜从播种到齐苗应该是 7-8 天
大蒜齐苗后下部开始长根，上部开始长叶，温度降至 10℃以下，达到六叶一心或七叶一心，停止生长为苗期。
大蒜的越冬期，在黄淮流域进入 12 日份，大雪节以后最高气温降至 10℃以下，越冬作物停止生长进入越冬期。
大蒜的返青期，翌年三月份气温开始回升，地温涿步提升到 10-12℃以上，心叶开始生长。随着地温升高，生长加快，到三月底四月初清明节前后，进入返春盛期。叶片生长出了 3-4 片以上为返青期。
大蒜的抽苔期，4 月上中旬，随着大蒜最后一片叶的生长，蒜苔开始分化形成，并且加快生长。到中下旬，蒜苔已经成熟开始抽苔，为抽苔期。
蒜头的彭大期，大蒜根茎叶的生长完全停止，所有光合作用生产的碳水化合物，大量往蒜头上集中。蒜头不断彭大有机物质积累越来越多，称为蒜头彭大期。一般抽苔后 18-20 天。
大蒜的成熟收获期：大蒜叶片由青转黄，假茎用脚踩棉软不断，养分已全部回收，开始收获。一般在 5 月中下旬。
大蒜返青盛期，内部开始分化鳞茎鳞芽，急需要大量的水肥，需要人工补充，水肥齐攻，大约在四月上旬，清明节前后，水溶肥从高氮为主，5-8 公斤。
是抽苔期，防止后期脱肥，也就是在上次浇水施肥后 8-12 天，再施一次肥，每亩 5 公斤左右水溶肥。
大蒜蒙头水，在大蒜种植完后立即浇水，确保大蒜苗齐苗壮。
大蒜的是越冬水，进入 12 月份气温降至 10℃以下停止生长，需要足墒越冬，浇一次水。常言说，大蒜不怕湿冻怕干冻，充足的水份可以保护大蒜安全越冬。
大蒜的返青盛期的返青水，这一水不能早浇，必须在地温稳定在 15℃左右，结合施肥浇一次水。
大蒜的抽苔水，也叫摇尾水。大蒜蒜苔开始伸长上次浇水后 8-12 天，促施蒜苔快速生长，结合追肥。
大蒜的蒜头膨大水，抽完蒜苔以从后，大蒜失去生长点，光合作用产生的有机物质大量向蒜头集中，促使蒜头快速彭大，必须有水配合，但是不用追肥，此时根系已老化失去生长功能。一般抽苔后十八天就成熟了，可以收获,进入收获期。
大蒜是越冬作物，病虫害都比较轻。再者大蒜抗蚜虫飞虱，很少生虫子，只有生长后期有蛆和根螨危害，一般新地没种过大蒜的地块很少生。
大蒜根腐病，由两种菌类引起，一是细菌类病害，二是疫霉菌属，真菌类病害。发病症状都

是变黄、发黑，根尖表皮先腐烂，再整体腐烂，植株枯萎死亡。造成的原因，土壤湿度大，温度高不透气，或者化肥过多、集中烧苗，或音连年重茬，土壤带菌量大。我们要对症用药。细菌类病害用抗出素或铜制剂可防治。真菌类病害可用恶霉灵，醚菌脂类药剂进行防治。
大蒜的叶枯病：表现叶片干枯、失绿、严重的发干死亡。主要是真菌类病害表现多种类型：尖枯型、白斑型、条斑型、紫斑型等，防治方法：清理田园，减少病原菌来源，实行轮作换茬减少病菌的发生，加强田间管理，合理密植，科学调节肥水，药剂防治主要用药代森锰锌，阿米西达，苯醚甲环唑，咪鲜胺等，交错施用，减少抗性。
大蒜在不同的年份，不同的气候条件，发病的情况也不同。如紫斑病、锈病、菌核病、疫病、病毒病、叶斑病等，要对症用药及时防治，不能造成危害。
大蒜除草，首先在播种期用药封闭地面，出来杂草立即死亡。主要用药乙氧氟草醚，二甲戊乐灵、恶草酮。再者大蒜的生长中后期，随着气温的升高又长出来的杂草，属于带棵除草剂，主要用药辛酰、溴苯腈等，可有效杀死各种杂草不损伤大蒜。
草铵膦铵盐(精草铵膦铵盐)含量 10%+。施用方法：每 100 毫升兑水 30 斤，对着杂草定向喷雾，不同浓度和剂型可从按照使用说明书配比使用。注意事项：均匀喷雾，把杂草全部喷湿，早晨雨后有露水不能施用，避开中午强光照，半下午以后最好，喷药后 8-10 小时不能遇雨，影响药效。
草铵磷或草甘磷，性能：可以杀灭现有的绿色植物，遇土壤分解药效，过一段时间土壤还会再生杂草，时间长了再喷药。施用方法：因为浓度和剂型不同，严格按照说明书施用。注意事项：均匀喷雾，把杂草全部喷湿，早晨雨后有露水不能施用，避开中午强光照，半下午以后最好，喷药后 8-10 小时不能遇雨，影响药效。
在大蒜播种后，出苗前使用地面封闭式除草剂，所有出来的杂草全部死亡，并且不影响大蒜生长，这也是当前大蒜种植区普遍使用的方法，大蒜整体生育期基本无杂草。选用的农药：二甲戊乐灵+乙氧氟草醚+噁草酮。施用方法：在大蒜田均匀喷雾，不重喷、不漏喷，按要求用量施用。
在大蒜生长期内有杂草，属于带苗除草，喷药不影响大蒜生长。选用农药：1 辛酰溴苯腈、2 氯氟吡氧乙酸+吡氟酰草胺。方法：按浓度和剂型以及说明书配比使用，全田均匀喷雾。注意：杂草最好 5 片叶以下，半下午以后喷药，8 小时以后遇雨效果降低。
大蒜喜凉爽，怕炎热，可以耐-10℃的低温不死，冬季温度-15℃以下全部冻死，不能越冬。大蒜的正常生长温度是 12 一 25℃，低于 12℃能发芽但是不能长叶，25℃以上不能吸肥吸水而死亡。
大蒜存放应该在 25℃从上进入休眠期，-1℃—-5℃也可以存放，最好在-1℃—-3℃冷库储存，5℃以上芽萌动。
大蒜在黄准海地区属二年生草本植物,生育期在七个月左右 210-220，在高寒地区或北纬 38°以上冬季-15℃—-20℃以上，冬前不能种植，只能春播，一般生育期 120-130 天左右。
大蒜是需要春化的作物,需要在 5-6 片叶以前终过 0 一 12℃的低温两个月左右，渡过春化，如果春化不好或不能顺利的通过春化，指种过早或过晚，容易出现独头或形成面包蒜和毛蒜，失去商品价值。
全国大部分区都有大蒜种植，黑龙江、云南、西藏、新疆都能种植大蒜，但是各地都有自己的品种。
春化阶段：大蒜发芽以后在五、六片以前，经过 0 至 12℃的低温 2 个月以上，分化成新的蒜头，形成蒜瓣，这就叫春化(同小麦一样)。没有通过春化的大蒜形成独头，春化不好的大蒜形成气包蒜或马尾蒜。主要是特定的温度、时间、蒜苗的生长期所决定的。
光照阶段:大蒜完成春化以后，随着气温的升高进入光照阶段，这个时期大量生长根、茎、叶，积累营养，准备结实。这个时期，需要充足的适宜的光照，大蒜返青以后，叶片达到 18-

20 片，单株根系达到 180 一 200 条，茎粗大到直径 1.5-1.8 厘米，叶片厚度可达 2 一 3 毫米，长度达到 40-60 厘米，叶面积指数达到 4 一 5 个，叶色浓绿，也是大蒜需肥零水最多的时期。3 月中下旬-4 月上中旬。

结实阶段:也叫生殖生长阶段，这个时期大蒜的营养生长完成，根、茎、叶不在增加，体内积累了大量的碳水化合物，抽完蒜苔以后，全部转移到蒜头，迅速膨大，形以蒜头等茎叶全部枯黄、萎缩发黄转移完成收获。

大蒜的播种及出苗期,大蒜播种一般最高气温降到 18-22℃（即可播种本地，大体时间在寒露节前后，也就 10 月 5-15 日最适，腾种后一般出苗 5-7 天。

苗期：大蒜齐苗后开始生根长叶，一般温度降到 12℃以下停止生长，当地有句谚语，小雪不生，大雪不长，12 月上旬。

越冬期：大雪节至冬至节进入全年最寒冷阶段，到了 12 月中下旬，到了来年三月份中下旬，大体 90 天为越冬期。

返青期：春季随气温升高，稳定在 12℃以上，开始生长，15℃以上加快，18-22℃最旺，3 月中下旬-4 月上中旬。

抽苔期：4 月上中旬以后，随着最后一片叶的伸长，蒜苔开始生长，4 月的中下旬，蒜苔成熟，为抽苔期。

蒜头膨大期：抽苔以后，整株大蒜的营养都回流向蒜头，这就是大蒜膨大期，4 月下旬-6 月上中旬。

收获期：大蒜叶片由青变黄，假茎用脚踩踏，棉软不断，养分已全部回收，开始收获，5 月中下旬，大蒜的生育期没有明显的分界线，从一个生育期逐步进入另一个生育期前后重叠，只有初期、盛期和末期。

土壤肥沃度有四个标准①有机质含量高 15‰以上；②PH 值中性 6.5-7.5；③土壤疏松海绵田；④病虫害少，大量增施有机肥，改良土壤结构，努力提高肥沃度。

金乡大蒜以蒜头为主要商品，密度应该在 3-3.5 万/亩，因为直径 5.5 厘米是 50 克(一两)，达到 3500 斤-4000 斤/亩很容易

大蒜适期播种很重要，过早或过晚播种，春化不好容易奇型（独头、面包蒜、毛蒜、开裂等），一般最高温 18-22℃最适，大体时间每年 10 月 5 日一 15 日，寒露节前后。

大蒜的播种深度一般在 3 厘米左右,上齐下不齐，复土一厘米，刚把蒜头埋上，浇水冲不走为宜，过深烂种不出苗。

大蒜应施足底肥，N、P、K 总含量 120-140/亩，有机质 150 斤/亩以上，配合名种微量元素。

大蒜要浇好蒙头水，打好除草剂，盖好膜。

大蒜的蒜母（蒜瓣）可以供大蒜生长五片叶，不需要从土壤中吸收营养，有水和温度即可，年前生长 6 叶一心越冬，吸肥量很少。

大蒜的营养生长是指根、茎、叶的生长，这个时期以氮肥为主，磷钾肥为辅，主要指抽苔前。

返青从后需要大量的肥水，特别是温度在 15℃以上清明前后，必须保证水,肥充足。增加氮肥可显著旺盛，提高产量。

大蒜的生殖生长:为繁衍后代的生长、开花、结果。大蒜从开始抽苔、蒜头膨大为生殖生长，根、茎、叶生长停止。这个时期以水和磷钾肥为主，很少用氮、钾肥对蒜头膨大起着很重要的作用。氮肥主要促进叶绿体合成。

八、大蒜的主要病虫害

大蒜是越冬作物，病虫害比较轻。主要虫害集中到根部。在气温回暖以后，有根蛆和根螨，以拌种防治为最好。用噻虫嗪、高巧一类的药剂防治。

根腐病：有两种情况，有疫霉菌和腐烂菌引起。疫霉菌属真菌性病害，腐烂菌属细菌性病害。为害症状基本相同，根条发黑，根尖腐烂，严重时整体腐烂，苗期氮素过多烧苗，没有腐熟

好的各种粪肥，发酵发热。连年重茬带菌量过大，土壤湿度大，温度高不透气等。可对症治疗，苗期用恶霉灵，中后期用抗生素、铜制剂防治。
叶枯病：叶片干枯失绿、发干、死亡，严重时整株死亡。表现为四种发病类型：①尖枯型；②条斑型；③紫斑型；④白斑型。防治办法：①清理田园减少病原菌；②实行轮作换荐；③加强田间管理，合理密植，调节肥水；④药剂拌种；⑤药剂防治:代蒜锰锌、阿米西达、苯醚甲环唑、咪鲜胺等。
大蒜不同年份、不同气候环境条件，发病的情况不同。有些年份紫斑病、锈病、菌核病、疫病、病毒病、叶斑病等也很严重，要对症用药，防治在发病初期，不能造成危害。
大蒜除草封闭地面，乙草胺、乙氧氟草醚、施田补、扑草等。春季带跟除草，辛酰溴苯腈。
大蒜的成熟标准:蒜叶全部发黄，假茎用脚踩棉软不断，证明体内储存的碳水化合物已全部回收到蒜头。每一个蒜瓣都能鼓起。
大蒜如果全部是青叶收获，含水量大、有机物质少，一般减产 2 成左右。
大蒜是高含水的作物，一般含量 80%以上。晾晒非常重要，稍微不注意，容易霉变腐烂。收获后最好就地晾晒 3-5 天后再装袋，放在通风的地方，不能暴晒，一般不能少于 10-15 天，基部表皮都干了以后再入库诸存。商品大蒜要根系全部切除。
大蒜的晾晒有多种形式：全部用网袋包装，支架晾晒；竹薄晾晒等。要勤翻动，多通风。
大蒜的储存：长期储存必须用冷库，用支架全部摆放均匀，温度拉到零下 2-3 度，可以存 2-3 年。短期储存可利用夏季高温 25℃以上属大蒜的休眠期。但是不能超过 35℃，容易糖蒜、灼伤，温度降至 25℃以下时容易发芽。
大蒜对乳腺癌、结肠癌、膀胱癌有预防作用。大蒜有较强的抗氧化作用,可有效清除活性氧自由基,保护生物膜结构的完整,防止细胞和组织的癌变。另外,大蒜还有阻断霉菌对亚硝胺合成的促进作用,减少因亚硝胺诱发癌症的可能性。
大蒜根腐病微生物：由两种菌类引起，一是细菌类病害，二是疫霉菌属，真菌类病害。
大蒜根腐病发病时间:通常在出苗后的 2~3 叶期开始发病或翌年 2 月中下旬为发病高峰期，3 月为发病末期。
大蒜根腐病发病症状：初生根由根尖向基部腐烂，次生根随后腐烂，部分植株连“蒜母”一起腐烂，伴有腥臭味。根及根颈部变为粉红色，根须腐烂脱落，根量减少，茎盘肥大。叶片褪绿发黄，从叶尖开始沿叶脉纵向软腐，植株矮小，生长发育失调。生长后期，下位叶变黄或枯死，植株顶端受害不明显，但鳞茎变小。
大蒜根腐病发病原因：连年重茬种植、土壤湿度过大、温度在 25～30℃时病害易发生和扩展。长期连作导致病原菌积累，种植密度过大、通风透光不良、施肥不均匀或使用未腐熟的有机肥均会加重病害。
大蒜根腐病防治措施：实行轮作，避免连作，选择抗病品种。整地时施肥均匀并翻入 10 厘米以下土壤中，避免使用未腐熟的有机肥。发病初期可使用 3%甲霜恶霉灵 500～1000 倍液灌根，或 30%甲霜恶露灵 500～1000 倍液喷蒜根，其他有效药剂包括 40%福星乳油 8000 倍液、75%百菌清可湿性粉剂 600 倍液等，每隔 3～4 天喷 1 次，视病情连续防治 2～3 次。
叶枯病微生物：由枯叶格孢腔菌引起。
叶枯病发病时间：11 月初至 12 月中旬为第一高峰期，次年 1 月初至 2 月底为第二高峰期。常发生在大蒜生长的中后期。
叶枯病发病症状：发病部位自叶尖或叶其他部位开始扩展蔓延，初期病斑为苍白色圆形小斑点，以后扩大变灰黄或灰褐色，不正形或椭圆形，致全叶枯死，病部生黑色霉状物。花梗受害易从病部折断。
叶枯病发病原因：该病在 15℃至 20℃的温度范围内最易发生，高温多湿的环境会加速病害的传播和蔓延。土壤黏重、偏酸以及多年重茬种植也会增加病害发生几率。

叶枯病防治措施：播前药剂拌种浸种。发病初期使用 70%乙膦铝锰锌可湿性粉剂 500～700 倍液，每隔 7～10 天喷施一次，连续 2～3 次。每 667 平方米选用 40%多菌灵胶悬剂 0:15 千克加磷酸二氢钾 0.15 千克，加水 60 千克均匀喷施，或用 50%异菌脲可湿性粉剂 1500 倍液、50%丁戊己二元酸铜可湿性粉剂 500 倍液、80%代森锌可湿性粉剂 600～800 倍液、75%百菌清可湿性粉剂 600～800 倍液等喷雾轮作换茬。大蒜忌连作；加强田间管理，及时发现病株，并收集后烧毁或深埋；田间操作时要避免损伤叶片，以减少伤口。
紫斑病微生物：由葱链格孢引起的。
紫斑病发病时间：大蒜紫斑病发病适温为 25～27℃，一般温暖、多雨或多湿的夏季发病重。且多于生长后期发病。
紫斑病发病症状：田间发病多始于叶尖或花梗中部，几天后蔓延到下部，初呈稍凹陷白色小斑点，中央微紫色，扩大后呈黄褐色纺锤形或椭圆形病斑，湿度大时，病部产出黑色霉状物，病斑多具同心轮纹，易从病部折断，贮藏期染病的鳞茎颈部变为深黄色到红褐色软腐状。
紫斑病发病原因：采用平畦点播，浇水后易积水，田间湿度大，利于该病的发生。连作、邻作田未破坏病原物的活动场所，利于病原菌滋生，造成病害的严重发生种植密度过大，单株所占空间小，不利于植株的通风、透光，造成植株衰弱，易发病。
紫斑病防治措施：施足基肥，加强田间管理，实行 2 年以上轮作。种子用 40%甲醛 300 倍液浸 3 小时，浸后及时洗净。鳞茎可用 40～45℃温水浸 1.5 小时消毒。发病初期喷洒 75%百菌清可湿性粉剂 500～600 倍液或 64%杀毒矾可湿性粉剂 500 倍液、40%大富丹可湿性粉剂 500 倍液、58%甲霜灵·锰锌可湿性粉剂 500 倍液、50%扑海因可湿性粉剂 1500 倍液，隔 7～10 天 1 次，连续防治 3～4 次，均有较好的效果。此外，喷洒 2%多抗霉素可湿性粉剂 30 毫克/千克也有效。
锈病微生物：由葱柄锈菌引起的。
锈病发病时间：早春低温、多雨或梅雨期间多雨或秋季多雾、多雨的年份发病重，主要发病盛期为 3～6 月、10～11 月。
锈病发病症状：发病初期，叶片表皮下产生椭圆形稍隆起橙黄色小斑点，即夏孢子堆，不久表皮破裂，散出橙黄色粉末即夏孢子，后期病部形成黑褐色疱斑即冬孢子堆，发病严重时叶片布满锈粉，发黄枯死。
锈病发病原因：田块间连作田、地势低洼、排水不畅的、田块发病重；栽培上种植过密、通风透光差、肥料不足、生长不良的田块发病重。
锈病防治措施：选用抗锈病品种。避免葱蒜混种，注意清洁田园以减少初侵染源。适时晚播，合理施肥，减少灌水次数，杜绝大水漫灌。发病初期，选用 15%三唑酮可湿性粉剂 1500 倍液或 20%三唑酮乳油 2000 倍液，97%敌锈钠可湿性粉剂 300 倍液，25%敌力脱乳油 3000 倍液，或 25%敌力脱乳油 4000 倍液加 15%三唑酮可湿性粉利 2000 倍液、70%代森锰饵可湿性粉剂 1000 倍液加 15%三唑酮可湿性粉剂 2000 倍液，隔 10～15 天 1 次，防治 1 次或 2 次。
菌核病微生物：由大蒜核盘菌引起的。
菌核病发病时间：该病菌喜低温高湿，一般温度在 15—20℃，相对湿度在 85%以上，有利于菌核萌发和菌丝生长。一般在春季 2 月下旬或 3 月上旬，气温超过 6℃时，病菌开始生长繁殖。
菌核病发病症状：大蒜菌核病的发病部位在地膜下大蒜假茎基部，初期病部水渍状，以后病斑变暗色或灰白色溃疡腐烂，腐烂部位发出强烈的蒜臭味。湿度高时，表面长出白色绵毛状的菌丝，以至病株周围的杂草也布满白色菌丝，并溃疡腐烂。大蒜叶鞘腐烂后，上部叶片表现萎蔫，并逐渐黄化枯死，蒜根须、根盘腐烂，蒜头散瓣，一般在 5 月上旬以后病部可见到不规则状的黑褐色菌核，大小 3—18 毫米，表面较光滑，萌发时其上长出了囊盘若干个。
菌核病预防措施：收获后及时深翻，深度要求 20 厘米以上，将菌核埋入深层；播前用 10%

盐水漂种 2—3 次，除去菌核。采用紫外线塑料膜高畦覆盖，抑制菌源；种子和土壤消毒，种植前每亩用 20%甲基立枯磷 0.5 千克对细土 20 千克配成药土，拌匀撒施。种子用 50℃温水浸种 10 分钟，即可杀死菌核；露地出现子囊盘时，喷撒 5%百菌清粉尘剂，每亩用 1 千克，或用 50%扑海因或 50%农利灵可湿性粉剂 1000 倍液、20%甲基立枯磷乳油 1000 倍液、50%扑海因可湿性粉剂 1500 倍液加 30%甲基硫菌灵（甲基托布津）可湿性粉剂 1000 倍液于盛花期喷雾，隔 7—8 天喷 1 次，连续防治 3—4 次；在土壤中撒施石灰，可明显抑制菌核病的发生。

叶斑病微生物：由蒜尾孢菌引起的。

叶斑病发病时间：在大蒜种植的中后期多雨、多雾、露重，日暖夜凉的天气发病重。此外，该病在种植密度过大，田间湿度大，植株长势弱的田块发病重。

叶斑病发病症状：大蒜灰叶斑病主要为害大蒜的叶片。发病初期，叶片上的病斑淡褐色，长椭圆形，后变灰白色，叶两面病斑生微细灰黑色霉状物，即病菌干实体，严重的病斑汇合，引起叶片局部枯死。

叶斑病预防措施：实行轮作，清洁田园，加强田间管理，采用配方施肥技术增强寄主抗病力。发病初期开始喷洒 50%琥胶肥酸铜可湿性粉剂 500 倍液或 77%可杀得可湿性微粒粉剂 500 倍液、75%百菌清可湿性粉剂 600 倍液、50%多·硫悬浮剂 500 倍液、65%甲霉灵可湿性粉剂 1000 倍液、30%绿叶丹可湿性粉剂 500-800 倍液、12%绿乳铜乳油 500 倍液，47%加瑞农可湿性粉剂 600～700 倍液、50%扑海因可湿性粉剂 1000～1500 倍液，隔 10 天左右 1 次，防治 1 次或 2 次。

病毒病微生物：由大蒜花叶病毒、大蒜潜隐病毒等病毒单独或复合侵染、发生在大蒜的病害。

病毒病发病时间地点：在中国上海及长江中下游地区，大蒜病毒病的主要发病盛期，秋季在 9～11 月，春季在 4～6 月。

病毒病发病症状：大蒜病毒病叶片上表现为出现黄绿相间的条纹，病株地上部叶片变小，植株表现为矮缩，新生叶片偏小不能完全展开，使新叶扭曲畸形。病株的鳞茎球略小，须根较健株明显减少。蒜瓣减少，发生严重时，蒜瓣僵硬贮藏期仍可继续发病，表现为蒜瓣僵硬，危害程度比田间更为明显。

病毒病预防措施：大蒜收获后及时清除病残体，合理轮作，施足有机肥料，选择抗病能力强的优良品种，加强田间管理，发病初期应及时喷施 20%病毒克星 400 倍液进行防治，同时配合喷施新高脂膜 800 倍液增强药效；或及时喷施 1.5%植病灵乳剂 1000 倍液加新高脂膜 800 倍液防治。

疫病微生物：由葱疫霉引起的。

疫病发病条件：病菌喜高温、高湿条件，发病适温 25～32℃，相对湿度高于 95%并有水滴存在条件下易发病，露地大蒜在多雨季节或棚室大蒜放风不及时或浇水过量，形成高温、高湿条件发病重。

疫病发病症状：叶片发病，多在叶尖或叶片中部生病斑，迅速扩展病斑不规则水渍状，苍白色或浅黄色，边缘浅绿色，不太明显。湿度大时病斑腐烂，其上产生稀疏白色霉状物（即病菌孢囊梗和孢子囊）。叶病斑扩展后，病斑相连，3～4 天后致使半叶或整叶萎垂。花梗发病病部呈渍状腐烂，花梗常从病部折倒。发病重时，全株枯死。

疫病预防措施：选用抗性强的品种，注意排涝，防止湿气滞留。发病地 2～3 年内不要种植葱蒜类蔬菜，收获后要及时清除病残体，采用小垅或高畦栽培。发病初期喷洒 72%克露可湿性粉剂 800～1000 倍液或 72-2%普力克水剂 800 倍液、58%甲霜灵锰锌可湿性粉剂 500 倍液、40%三乙膦酸铝可湿性粉剂 250 倍液、64%杀毒矾可湿性粉剂 400～500 倍液。对上述杀菌剂产生抗药性时，可选用 69%安克锰锌可湿性粉剂 1000 倍液。

灰霉病微生物：由葱鳞葡萄孢和葱腐葡萄孢引起。

灰霉病发病条件: 大蒜灰霉病在田间主要靠病原菌的无性繁殖体, 即病叶上的灰霉传播蔓延。每次收蒜都会把病菌散落于土表，致新生叶染病。蒜苔灰霉病主要在蒜苔收获后，靠潜伏在植株上的菌丝体、菌核, 在贮藏低温条件下引起发病, 病部产生的分生孢子借气流传播蔓延，从伤口或枯死部位侵入，进行多次重复再侵染，贮藏窖或贮藏库低温、高湿条件下发病重。
灰霉病发病症状: 发病初期蒜苗叶两面生有褪绿小白点，扩展后成为沿脉扩展的长形或梭形斑，大小 0.5～3 毫米，一般从叶尖向下扩展，致多数叶片一半枯黄，湿度大时，密生较厚的灰色绒霉层，致叶片变褐或呈水渍状腐烂。
灰霉病预防措施：选用抗病品种，及时清除病残体，适时通风降湿，发病初期喷洒 50%普海因可湿性粉剂 1000 倍液或 50%速克灵可湿性粉剂 1500～2000 倍液、40%多菌灵硫磺胶悬剂 800～1000 倍液、50%灭病威可湿性粉剂 600～800 倍液，隔 7～10 天一次，连续防治 2～3 次。
白腐病微生物：由白腐小核菌引起的。
白腐病发病条件: 该菌喜低温高湿，在高温季节湿度不大条件下扩展缓慢；当气温低于 20℃湿度大持续时间长易流行。生产上早春生长瘦弱的蒜田易发病，进入雨季后病势扩展迅速。此外，该病在长期连作葱蒜类、排水不良、土壤肥力不足的地块发病重。
白腐病发病症状：初染病时外叶叶尖呈条状或叶尖向下变黄，后扩展到叶鞘及内叶，植株生长衰弱，整株变黄矮化或枯死，拔出病株可见鳞茎表皮产生水渍状病斑，长有大量白色菌丝层，病部呈白色腐烂，茎基变软，鳞茎变黑腐烂。
白腐病预防措施：实行轮作，发现病株及时挖除，播种前用蒜重的 0.5～1%的 50%甲基硫菌灵或多菌灵可湿性粉剂给蒜种包粉衣后再播种。发病初期喷洒 50%多菌灵可湿性粉剂 500 倍液或 50%甲基硫菌灵可湿性粉剂 600 倍液、50%扑海因可湿性粉剂 1000～1500 倍液灌淋根茎。贮藏期也可用上述杀菌剂喷洒，其中以 50%扑海因可湿性粉剂效果好。此外，也可喷淋 20%甲基立枯磷乳油 1000 倍液，隔 10 天左右 1 次，防治 1 次或 2 次。采收前 3 天停止用药。
煤斑病微生物：由葱枝孢引起的。
煤斑病发病条件：病原萌发的温度范围 0～30℃，以 10～20℃最快。空气相对湿度 100%和有自由水存时有利于萌发。植株生长不良，阴雨潮湿多露天气及生长后期发病重。
煤斑病发病症状：发病初期，叶片上出现灰白色的点状病斑，随着病情的发展，逐渐扩大成椭圆形或梭形病斑，病斑中央为枯黄色，边缘红褐色，外围黄色，最后叶尖扭曲枯死。湿度大时病斑产生绒毛状，干燥时呈粉状。
煤斑病预防措施: 选用抗病良种，适时播种，合理密植，施足底肥，及时追肥，清除病残体，药剂防治，可选用 65%代森锌可湿性粉剂 400～600 倍液，或 1:1:100 波尔多液，于发病初期开始喷洒，隔 7～10 天 1 次，连续防治 2～3 次。
软腐病微生物：由胡萝卜软腐欧氏杆菌胡萝卜软腐致病型引起的。
软腐病发病条件：从地块选择角度来说，一般连作地块，地势低洼、排水不良的地块发病比较重。另外，如果上茬种植地块中曾经发生了软腐病，在这样的地块中种植大蒜，往往发生大蒜软腐病的概率就会比较高。
软腐病发病症状:。从脚叶开始由外向内发黄，后逐渐向上部叶片蔓延，地下茎基部呈黄褐色软腐状，根系亦出现黄褐色腐烂，有臭味，植株一拨即断。后期轻病株停止软腐，但植株生长停滞，瘦弱矮小；重病株全部叶片枯黄，根系软烂，整株塌地而死。
软腐病预防措施：重病地避免连作，清除田间病残体，要加强栽培管理，收获前一周停止浇水。播前用 72%农用链霉素可溶性粉剂的水溶液浸蒜种，每千克蒜种用药 200 毫克，加水稀释后浸蒜种 24 小时，浸后晾干播种。发病初期喷施 72%农用链霉素（或新植霉素）可溶性粉剂 4000～5000 倍液, 47%加瑞农可湿性粉剂 800 倍液, 20%噻菌铜悬浮剂 500～700 倍液,

77%氢氧化铜可湿性粉剂 800 倍液或其他有效药剂。
干腐病微生物：由洋葱尖镰孢菌引起的。
干腐病发病条件：大蒜干腐病的发病适温为 28～32℃，在临近成熟期，土壤高温高湿有利于发病。重茬地，种蝇为害造成根系和鳞茎伤口，发病加重。贮运期间温度 28℃左右时，大蒜最易腐烂，于 8℃以下较轻。
干腐病发病症状：病株叶片从叶尖迅速萎枯，根大部分腐烂，鳞茎基部有病斑，纵向切开鳞茎，可见病斑内部呈半水渍状腐烂，并向内向上蔓延扩展，病情发展缓慢。在大蒜收获季节，对新侵染的蒜头不易辨别，这些带病蒜头，在后期加重为害造成腐烂。贮运期间，多自侵染的根部开始发病，蔓延至基部，致使蒜瓣黄褐，逐渐干缩，病部可产生橙红色霉层。
干腐病预防措施：及时清除病残体，合理轮作，深翻土壤、施足基肥，加强田间管理，，发病初期应连续喷洒 1∶1∶200 波尔多液 2～3 次，喷洒 75%百菌清可湿性粉剂 700 倍液加新高脂膜 800 倍液进行防治，或 50%多福可湿性粉剂 500 倍液加新高脂膜 800 倍液进行防治。贮藏运输，蒜头在收获贮藏过程中尽力避免损伤。贮藏的室内温度保持在 0℃，相对湿度在 65%左右为好，并要注意空气流通，防治干腐病的发生。
花叶病微生物：大蒜花叶病毒
花叶病发病规律：播种带病蒜苗，出苗既染病，且有病虫以汁液摩擦传毒感染。
花叶病发病症状：发病初期，沿叶脉出现断续黄条点，后连接成黄绿相间长条纹，植抹矮化，且个别植株心叶被邻近叶片包住，呈卷曲状畸形，长期不能完全伸展，致叶片扭曲。病株鳞茎变小，或蒜办及须根减少，严重的蒜瓣僵硬，贮藏期尤为明显。
4、预防措施：严格选种，大力推广营养茎尖、生殖茎尖分生组织的离体培养，避免与大葱、韭菜等葱属植物邻作或连作，减少田间自然传播。发病初期开始喷洒 1.5%植病灵乳利 1000 倍液或 20%病毒 A 可湿性粉剂 500 倍液、83 增抗利 100 倍液、抗毒剂 1 号水剂 250～300 倍液，隔 10 天左右 1 次，连续防治 2～3 次。用抗毒剂 1 号水剂 250 倍液灌根，每株灌对好的药液 50～100 毫升，隔 10～15 天一次，共灌 2～3 次，必要时喷淋与灌根结合，效果更好。
碳疽病微生物：真菌感染
碳疽病发病规律：此病为真菌病害，菌丝体和孢子在越冬后，在高温高湿条件下易发病。
碳疽病发病症状：在半枯的叶片和花茎上，形成近梭形或不规则形褐色病斑，以后生出许多黑色小点。在大蒜蒜头和蒜瓣上生有褐色稍凹陷的圆形、近圆形斑，其上散生或轮生多数小黑点。鳞茎外侧鳞片或颈部下方生有圆形稍凹陷的褐色病斑，扩大后连接成大病斑并深入鳞茎内部，弓 I 起腐烂，病斑上散生或轮生黑色小粒点。
碳疽病预防措施：轮作，种子消毒。蒜苗生长前期，定期叶面喷施抗枯宁、疫霉清、磷酸二氢钾等，提高抗逆性。在发病初期和雨季前喷药。有效药剂有 50%甲基托布津可湿性粉剂 600 倍液，75%百菌清可湿性粉剂 600 倍液，80%炭疽福美可湿性粉剂 500 倍液和波尔多液(1:1, 160~240)等。
黑粉病微生物：由洋葱条黑粉菌引起的。
黑粉病发病规律：播种后气温 10～25℃可发病，最适发病温度 18～20℃，高于 29℃则不发病。播种过深，发芽出土迟，与病菌接触时间长或土壤湿度大发病重。由于该病是系统侵染，田间健株仍保持无病，当叶长到 10～20 厘米后，一般不再发病。
3、发病症状：病株的叶、叶鞘和鳞茎上会出现灰色条纹，条纹内病组织上充满黑色厚垣孢子，受害叶片扭曲下弯，病苗或病株枯死。
4、预防措施：进行 2～3 年轮作，对带菌种子可用种子重量 0.2%的 50%福美双或拌种双粉剂拌种，也可用 40%福尔马林 50 倍液浸种 10 分钟后，冲洗干净再催芽播种。用 5%粉锈宁可湿性粉剂 1500-2000 倍液，或 20%粉锈宁乳油 2500 倍液，或 40%多·硫悬浮剂 500 倍液，或

50%硫磺悬浮剂 300 倍液，或 2%武夷霉素水剂 200 倍液，或 30%特富灵可湿性粉剂 2000 倍液，或 40%杜邦福星乳油 8000～1000 液等药剂喷雾防治，每 7 天 1 次，连续防治 2～3 次。发现病株及时拔除，集中烧毁。葱苗长到 15 厘米，病菌停止侵染，选无病苗栽植。

青霉病微生物：由黄青霉引起的。

青霉病发病规律：病菌在土壤空气中由伤口侵入，进入并感染蒜瓣组织，再由外部因素，加速传播感染。

青霉病发病症状：初期蒜头外部出现浅黄色病斑，1 个或几个蒜瓣呈水渍状，后形成灰褐色不规则形凹陷斑，在潮湿情况下很快长出青绿色霉状物为分生孢子梗和分生孢子。贮存时间长久，霉状物加厚，呈粉块状，严重时，病菌侵入蒜瓣内部，组织变黄、松软、干腐、通常蒜头上有 1 个或数个蒜瓣干腐。

青霉病预防措施：抓好鳞茎采收和贮运，尽量避免遭受机械损伤，不宜在雨后、重雾或露水未干时采收。贮藏窖可用 10 克/平方米硫磺密闭薰蒸 24 小时。采收前一周喷洒 70%甲基硫菌灵超微可湿性粉剂 1000 倍液或 50%苯菌灵可湿性粉剂 1500 倍液。加强贮藏期管理，贮存温度控制在 5～9℃，相对湿度 90%左右。

曲霉病微生物：由黑曲霉引起的。

曲霉病发病规律：高温高湿、土壤温湿度变化激烈或有湿气滞留易发病。蒜头剪头过早，留梗过短的发生较多。一般白皮蒜比褐皮蒜、紫皮蒜易感病。

曲霉病发病症状：发病时，初期一个或几个蒜瓣发病，湿度大时病部长出白色菌丝体，使蒜瓣充满黑粉，即分生孢子。其症状与大蒜黑粉病相似。

曲霉病预防措施：可以参考黑粉病措施。

1、基本信息：蒜蛆是双翅目花蝇科的一些昆虫幼虫的统称，主要包括种蝇、葱蝇等的幼虫。

2 危害：主要危害大蒜、洋葱、大葱等百合科蔬菜。影响生长：蒜蛆以幼虫形式在土壤中蛀食大蒜的鳞茎，导致蒜瓣腐烂，地上部分叶片发黄、枯萎，严重时整株死亡，造成大蒜产量降低和品质下降。

3、体型特征成虫体型较小，一般体长在 4-6 毫米左右，外观类似家蝇但比家蝇小，多为灰色或灰黄色。幼虫呈乳白色，头部尖细，尾部钝圆，身体有明显的体节，无足，体长约 7-9 毫米。

4、生存习惯：繁殖特点：蒜蛆一年可发生多代，以蛹在土壤中越冬。成虫喜欢在大蒜植株周围的土缝或蒜根处产卵，卵期一般为 3-5 天。适宜环境：喜欢温暖潮湿的环境，一般在春季和秋季危害较重。土壤湿度在 70%-80%时有利于蒜蛆的发生，此外，未充分腐熟的有机肥也会吸引成虫产卵。

5、预防措施：农业防治：选用抗虫品种，合理轮作，避免与葱、蒜等作物连作。施用充分腐熟的有机肥，减少成虫产卵的机会。

物理防治：在成虫发生期，利用糖醋液诱杀成虫。糖醋液的配方为糖：醋：酒：水=3：3：1：10，加入少量敌百虫等杀虫剂，装入开口较大的容器中，每亩放置 3-5 个。

化学防治：播种前，可用辛硫磷等药剂进行土壤处理。在蒜蛆发生初期，用高效氯氰菊酯、吡虫啉等药剂灌根防治。

1、基本信息：豌豆潜叶蝇，属双翅目潜蝇科。其成虫是一种小型蝇类，在我国大部分地区都有分布。它不仅危害豌豆，也会对大蒜等作物造成损害。

2、危害：豌豆潜叶蝇的幼虫会潜入大蒜叶片的叶肉组织内取食，形成弯曲的白色虫道。随着幼虫的生长，虫道会逐渐加宽。这会破坏叶片的光合作用组织，导致叶片的光合能力下降，使大蒜叶片发黄、枯萎，影响大蒜的生长发育，进而降低大蒜的产量和品质。

3、体型特征：成虫体长约 2-3 毫米，翅展 5-7 毫米，体暗灰色，有稀疏的刚毛。复眼椭圆形，红褐色至黑褐色。触角短小，3 节，第 3 节近圆形，触角芒细长，着生于触角第 3 节基

部。前翅半透明，有紫色闪光，后翅退化为平衡棒。卵长椭圆形，乳白色，长约 0.3 毫米。幼虫蛆状，老熟幼虫长约 3 毫米，初孵时乳白色，渐变淡黄色，体表光滑柔软。蛹长椭圆形，长约 2-2.6 毫米，初期为淡黄色，后变为黄褐色。

4、生存习惯：豌豆潜叶蝇在温暖地区可终年繁殖，在北方以蛹在被害叶片内越冬。成虫白天活动，吸食花蜜，喜欢在幼嫩叶片上产卵。卵多产在叶背边缘的叶肉内，每处产卵 1 粒。幼虫孵化后即潜入叶内取食叶肉，老熟后在虫道末端化蛹。该虫发育最适温度为 20℃左右，高温会抑制其生长发育。

5、预防措施：农业防治：及时清除田间杂草和残株落叶，减少越冬虫源。合理密植，增强通风透光性，降低田间湿度，创造不利于豌豆潜叶蝇生长的环境。

物理防治：在田间悬挂黄板诱杀成虫，每亩挂 20-30 块，黄板高度以略高于大蒜植株为宜。

生物防治：保护和利用天敌，如姬小蜂、反颚茧蜂等，这些天敌对豌豆潜叶蝇有较好的控制作用。

化学防治：在成虫羽化盛期或幼虫初孵期，选用阿维菌素、灭蝇胺、氯虫·高氯氟等药剂喷雾防治。注意药剂要交替使用，以延缓害虫抗药性的产生。

螨类基本信息：螨类属于蛛形纲蜱螨亚纲，种类繁多，在大蒜上常见的有叶螨、根螨等。叶螨多在叶片活动，根螨主要危害大蒜根部和鳞茎。它们体型微小，需借助放大镜或显微镜才能看清。

螨类危害：叶片受损：叶螨以刺吸式口器吸食大蒜叶片汁液，使叶片出现黄白色斑点，严重时斑点连片，叶片枯黄，影响光合作用，导致大蒜生长发育不良。鳞茎受害：根螨会蛀食大蒜鳞茎，造成鳞茎腐烂，影响蒜头的品质和产量，还可能导致植株倒伏、死亡。传播病害：螨类在大蒜植株上活动时，可能会传播一些病菌，加重大蒜病虫害的发生程度。

螨类体型特征：螨类身体通常呈圆形或椭圆形，分为颚体和躯体两部分。颚体有口器，用于取食。躯体上有四对足，足上有刚毛和爪。其体长一般在 0.2-0.6 毫米左右，体色多样，如叶螨有红色、绿色等，根螨多为白色或浅黄色。

螨类生存习惯：螨类喜欢温暖干燥的环境，适宜生长温度一般在 20-30℃。叶螨多聚集在叶片背面，根螨则生活在土壤中，主要在大蒜根部和鳞茎周围活动。它们繁殖能力强，多为两性生殖，也可孤雌生殖，繁殖速度快，在适宜条件下，数天就能繁殖一代。

螨类预防措施：农业防治：选择抗螨品种，合理轮作，避免与易受螨类危害的作物连作。及时清除田间病残体和杂草，减少螨类滋生场所。

物理防治：高温闷棚，在夏季高温季节，将大棚密闭 7-10 天，使棚内温度升高到 60-70℃，可有效杀死螨类。也可使用防虫网，防止螨类进入田间。

生物防治：释放捕食螨等天敌，控制螨类种群数量。还可使用微生物制剂，如球孢白僵菌等，对螨类有一定的抑制作用。

化学防治：选用阿维菌素、螺螨酯、乙螨唑等杀螨剂，按照说明进行喷雾或灌根处理。注意药剂的轮换使用，避免螨类产生抗药性。

葱蓟马基本信息：葱蓟马又称烟蓟马、棉蓟马，属缨翅目蓟马科。它是一种世界性害虫，能为害多种蔬菜及农作物，大蒜是其为害的主要作物之一。

葱蓟马危害：叶片损伤：以锉吸式口器吸食大蒜叶片的汁液，使叶片形成许多细密的灰白色斑点，严重时叶片扭曲枯黄，影响光合作用，导致大蒜生长缓慢，植株矮小。蒜头变小：生长受抑制，蒜薹发育不良，蒜头也会变小，影响大蒜的产量和品质。传播病害：葱蓟马在取食过程中，还可能传播大蒜的病毒病，加重病害发生，造成更大的损失。

葱蓟马体型特征：成虫体长 1.2-1.4 毫米，体色从淡黄色至深褐色不等。身体细长，略扁，翅狭长，边缘有长而整齐的缘毛。复眼较大，呈紫红色。触角 7 节，细长。若虫与成虫相似，但体型较小，无翅或仅有翅芽。

葱蓟马生存习惯：温度偏好：葱蓟马喜温暖干旱的环境，适宜生长发育的温度为 23-28℃，相对湿度为 40%-70%。在高温高湿条件下，其繁殖和生存会受到一定抑制。隐蔽性：成虫和若虫多在叶片背面、叶鞘内等隐蔽处活动取食，有较强的趋嫩性，喜欢为害大蒜的幼嫩部位。繁殖特点：繁殖能力强，一年可发生多代，以成虫或若虫在大蒜植株基部、土壤缝隙或附近杂草中越冬。

葱蓟马预防措施：农业防治：合理密植，加强田间管理，及时清除田间杂草和病残株，减少葱蓟马的滋生地和越冬场所。

物理防治：利用葱蓟马的趋蓝性，在田间悬挂蓝色诱虫板诱杀成虫。

化学防治：在葱蓟马发生初期，可选用吡虫啉、啶虫脒、乙基多杀菌素等药剂进行喷雾防治，注意药剂的轮换使用，以延缓抗药性的产生。同时，要确保药剂均匀喷洒在大蒜叶片的正反面及植株基部。

蚜虫基本信息：蚜虫又称腻虫、蜜虫，是一类植食性昆虫，种类繁多，常见为害大蒜的有桃蚜、葱蚜等。它们分布广泛，几乎在所有大蒜种植区都有出现。

蚜虫危害：吸食汁液：以刺吸式口器刺入大蒜叶片、茎秆等部位，吸食植株汁液，导致叶片出现褪绿斑点，严重时叶片卷曲、发黄，甚至干枯死亡，影响大蒜的光合作用和正常生长。

传播病毒：蚜虫是大蒜病毒病的重要传播媒介，它们在不同植株间取食时，会将病毒传播扩散，使大蒜感染病毒病的几率大大增加，导致大蒜品质下降，产量降低。

分泌蜜露：蚜虫分泌的蜜露会覆盖在大蒜叶片表面，影响叶片的呼吸和光合作用，同时蜜露还容易滋生霉菌，引发煤污病，进一步影响大蒜的生长和发育。

蚜虫体型特征：一般体型较小，长度多在 1-5 毫米之间。身体柔软，多呈绿色、黄色、黑色等，有的种类体表有蜡粉或绒毛。蚜虫具有一对腹管，位于腹部第六节两侧，是其重要的鉴别特征，腹管可分泌防御物质。它们的触角较长，通常为 6 节。

蚜虫生存习惯：繁殖能力强：蚜虫具有孤雌生殖和两性生殖两种方式，繁殖速度极快。在适宜的环境条件下，雌蚜可直接产下若蚜，4-5 天即可发育为成虫，继续繁殖，短期内能形成庞大的种群。喜温暖干燥：适宜生长繁殖的温度一般为 15-25℃，相对湿度在 50%-80%。在温暖干燥的气候条件下，蚜虫繁殖活跃，为害加重。聚集性：多聚集在大蒜的嫩梢、叶片背面等幼嫩部位，以群体形式取食和活动。

蚜虫预防措施：农业防治：合理轮作，避免与易感染蚜虫的作物连作或套种。及时清除田间杂草和病残株，减少蚜虫的滋生地和食物来源。

物理防治：利用蚜虫的趋黄性，在田间悬挂黄色诱虫板诱杀成虫。也可在田间设置防虫网，防止蚜虫迁入。

生物防治：保护和利用蚜虫的天敌，如瓢虫、草蛉、食蚜蝇等，通过生物间的捕食关系控制蚜虫种群数量。

化学防治：在蚜虫发生初期，选用吡虫啉、啶虫脒、高效氯氟氰菊酯等药剂进行喷雾防治。注意药剂的浓度和使用方法，确保均匀喷洒在大蒜植株上，尤其是叶片背面和嫩梢部位。同时，要严格按照农药安全使用规定进行操作，避免农药残留。

茎线虫基本信息：茎线虫是一种植物寄生线虫，种类较多，其中危害大蒜的主要是起绒草茎线虫等。它分布广泛，在世界多个大蒜产区都有发生，主要通过土壤、蒜种、灌溉水等途径传播。

茎线虫危害：影响生长：茎线虫侵入大蒜后，会在组织内寄生并取食，刺激细胞增生和分裂，导致大蒜植株生长受阻，出现矮化、瘦弱等现象。

鳞茎病变：茎线虫会破坏大蒜鳞茎的正常结构，使鳞茎组织变软、腐烂，严重时整个鳞茎失去食用价值和种用价值。

产量降低：由于植株生长不良和鳞茎病变，大蒜的产量会显著下降，给蒜农带来较大的经济

损失。
茎线虫体型特征：茎线虫体型细小，通常呈细长线状，体长一般在 1 毫米左右。身体半透明，体表有细环纹，头部较尖，尾部较钝。具有口针，这是其穿刺植物组织和吸取汁液的重要器官。
茎线虫生存习惯：土壤栖息：茎线虫主要栖息在土壤中，尤其是在大蒜根际周围的土壤中数量较多。它们可以在土壤中存活较长时间，即使没有寄主植物，也能以休眠状态度过不良环境。
喜湿润环境：适宜在湿润的土壤中生存和繁殖，土壤湿度在 40%-70%左右有利于其活动和侵染。在干燥的土壤中，其活动会受到抑制。
寄生方式：主要通过大蒜的伤口或自然孔口侵入植株，然后在组织内迁移和繁殖。它们可以在大蒜的茎、叶、鳞茎等部位寄生，但以鳞茎受害最为严重。
茎线虫预防措施：农业防治：实行轮作倒茬，避免连作，与非寄主作物轮作 3-5 年。选用无病蒜种，播种前严格挑选，剔除有病、有伤的蒜种。收获后及时清除病残体，带出田外集中销毁，减少田间虫源。
物理防治：对土壤进行高温处理，如在夏季高温季节，将土壤深翻暴晒，使土壤温度升高到 50℃以上，可有效杀死茎线虫。也可采用水淹法，在种植前将田块灌水浸泡一段时间，使茎线虫缺氧死亡。
化学防治：播种前可用噻唑膦等药剂处理土壤，或用阿维菌素等药剂浸种，以杀死土壤中和蒜种上的茎线虫。在生长期间，如发现茎线虫为害，可选用合适的杀线虫剂进行灌根处理。
生物防治：利用一些有益微生物，如芽孢杆菌、放线菌等，它们可以抑制茎线虫的生长和繁殖，通过在土壤中添加这些有益微生物制剂，可改善土壤微生态环境，减轻茎线虫的为害。
大蒜粪蝇基本信息：大蒜粪蝇属于双翅目粪蝇科，是大蒜生长过程中常见的害虫之一。它主要分布在大蒜种植区域，尤其在温暖湿润的环境中较为常见。
大蒜粪蝇危害：幼虫蛀食：大蒜粪蝇的幼虫会蛀食大蒜的鳞茎，在鳞茎内形成隧道，导致鳞茎受损，影响大蒜的品质和产量。受损的鳞茎容易腐烂，降低大蒜的耐储存性。
传播病菌：成虫在取食和活动过程中，可能会携带病菌，如大蒜叶枯病、灰霉病等病原菌，在不同植株间传播，加重病害的发生和蔓延，进一步影响大蒜的生长和发育。
大蒜粪蝇体型特征：成虫体长约 5-8 毫米，身体呈黑色或黑褐色，具有金属光泽。头部较大，复眼发达，触角较短。翅膀透明，有明显的脉纹。足细长，跗节末端有爪和肉垫。幼虫呈乳白色，蛆状，无足，身体前端较尖，后端较钝，体表有许多横纹。
大蒜粪蝇生存习惯：繁殖习性：大蒜粪蝇一般在春季和秋季繁殖，成虫喜欢在大蒜植株周围的土壤、残株或粪肥上产卵。卵孵化后，幼虫会迅速钻入大蒜鳞茎内取食。
栖息环境：成虫喜欢栖息在潮湿、阴暗的环境中，如大蒜田的杂草丛、土缝等。它们对腐烂的有机物有趋性，常聚集在粪肥、垃圾等附近。
活动规律：成虫具有较强的飞行能力，白天活动，以花蜜、果汁等为食。在温暖、晴朗的天气里，活动更为频繁。
大蒜粪蝇预防措施：农业防治：合理密植，加强田间管理，及时清除田间杂草和病残株，减少大蒜粪蝇的滋生地。避免在大蒜田附近堆放粪肥等易吸引粪蝇的物质。
大蒜粪蝇物理防治：在大蒜田设置糖醋液诱捕器，利用成虫对糖醋液的趋性进行诱杀。也可以使用防虫网覆盖大蒜田，防止成虫进入产卵。
大蒜粪蝇化学防治：在成虫发生高峰期，可选用高效氯氰菊酯、溴氰菊酯等药剂进行喷雾防治。在幼虫孵化初期，可用辛硫磷等药剂灌根，防治幼虫为害。
大蒜粪蝇生物防治：保护和利用大蒜粪蝇的天敌，如寄生蜂、捕食性昆虫等，通过生物间的相互制约来控制其种群数量。

轮紫斑跳虫基本信息：轮紫斑跳虫隶属弹尾目，是一种常见的土壤微型节肢动物。在大蒜种植区的土壤、落叶及潮湿环境中较为常见。它具有较强的适应性，能在多种环境中生存。
轮紫斑跳虫危害：啃食组织：轮紫斑跳虫会啃食大蒜的根系、鳞茎和叶片等部位。对根系的啃食会影响大蒜对水分和养分的吸收，导致植株生长缓慢、矮小；对鳞茎的侵害会使鳞茎表面出现伤痕，影响其品质和商品价值，严重时还会导致鳞茎腐烂；对叶片的取食则会造成叶片上出现小孔洞，影响光合作用。
传播病菌：跳虫在大蒜植株间活动时，可能携带和传播一些病菌，如大蒜锈病、叶斑病等病原菌，从而加重病害的发生，进一步影响大蒜的生长和产量。
轮紫斑跳虫体型特征：轮紫斑跳虫体型微小，一般体长在 1-3 毫米左右。身体呈长椭圆形，背部略微隆起，颜色多为深灰色或紫褐色，具有光泽，体表有明显的紫斑。头部较小，触角细长，通常为 4 节。它有三对足，足上有刺毛，腹部末端有弹跳器官弹器，能使它在遇到危险时迅速跳跃逃离。
轮紫斑跳虫生存习惯：喜潮湿环境：轮紫斑跳虫喜欢生活在潮湿、阴暗的环境中，如土壤表层、落叶下、腐烂的有机物堆里等。土壤湿度在 70%-80%左右时最适宜其生存和繁殖。食性复杂：属于杂食性昆虫，主要以腐烂的植物残体、真菌菌丝、藻类等为食，也会取食大蒜等植物的活体组织。繁殖能力强：繁殖方式主要为孤雌生殖，在适宜的环境条件下，繁殖速度很快。雌虫可直接产下幼体，幼体经过几次蜕皮后即可发育为成虫。
轮紫斑跳虫预防措施：农业防治：合理密植，改善大蒜田的通风透光条件，降低田间湿度。及时清除田间杂草、落叶和病残株，减少轮紫斑跳虫的食物来源和栖息场所。
轮紫斑跳虫物理防治：在大蒜田铺设地膜，阻止跳虫从土壤中爬出为害植株。也可在田边设置一些糖醋液诱捕器，诱杀跳虫成虫。
轮紫斑跳虫化学防治：在跳虫发生严重时，可选用毒死蜱、辛硫磷等药剂进行土壤处理或喷雾防治。但要注意药剂的使用浓度和安全间隔期，避免农药残留对环境和大蒜造成污染。
轮紫斑跳虫生物防治：保护和利用轮紫斑跳虫的天敌，如蜘蛛、蚂蚁等，通过生物间的捕食关系来控制跳虫的种群数量。
蛴螬基本信息：蛴螬是鞘翅目金龟甲总科幼虫的统称，别名白土蚕、核桃虫等。种类繁多，在我国大部分地区均有分布，是地下害虫中种类最多、分布最广、为害最重的一大类群。在大蒜产区，蛴螬是常见的害虫之一。
蛴螬危害：咬食根系：蛴螬主要在地下活动，以大蒜的根系为食。它们会咬断大蒜的根，导致根系受损，影响大蒜对水分和养分的吸收，使植株生长不良，叶片发黄、矮小，甚至整株死亡。破坏鳞茎：蛴螬还会蛀食大蒜的鳞茎，在鳞茎上造成孔洞，使鳞茎腐烂，降低大蒜的产量和品质。被蛴螬为害的大蒜，不仅在生长期间表现出明显的受害症状，而且在收获后也容易因伤口感染病菌而发生腐烂，不利于储存和运输。
蛴螬体型特征：蛴螬身体呈长椭圆形，肥大弯曲，多为白色，少数为黄白色。头部褐色，较坚硬，有光泽，生有左右对称的刚毛。体壁较柔软，多皱，体表有细毛。胸足 3 对，一般后足较长。腹部 10 节，末节称为臀节，臀节上生有刺毛，其数量和排列方式是识别蛴螬种类的重要依据。
蛴螬生存习惯：栖息环境：蛴螬终生在土中生活，喜欢栖息在疏松、肥沃的土壤中。土壤的温度和湿度对其活动有很大影响，一般在 13-18℃时活动最盛，土壤过干或过湿都会影响其生存和活动。
食性特点：蛴螬是多食性害虫，除为害大蒜外，还可为害多种蔬菜、花卉、果树及农作物的地下部分。它们主要取食植物的根系、块根、块茎等地下器官，也会取食腐殖质。
繁殖规律：成虫（金龟甲）一般在 5-7 月间出土活动，交尾后产卵于土中。卵孵化后，幼虫在土中生长发育，经过 3 次蜕皮后化蛹，蛹期一般为 10-15 天。成虫羽化后，在土中越冬，

次年春天再次出土活动。
蛴螬预防措施：农业防治：合理轮作，避免与花生、大豆等蛴螬喜食的作物连作。秋季深翻土地，将越冬的蛴螬翻至地表，使其被冻死、晒死或被天敌捕食。同时，及时清除田间杂草和病残株，减少蛴螬的滋生地。
物理防治：在成虫发生期，利用金龟甲的趋光性，在田间设置黑光灯或频振式杀虫灯诱杀成虫。也可以在田间悬挂糖醋液诱捕器，诱杀金龟甲成虫，糖醋液的配方为糖：醋：酒：水=3：4：1：2，并加入少量敌百虫等杀虫剂。
化学防治：在播种前，可用辛硫磷、毒死蜱等药剂进行土壤处理，将药剂均匀撒在地表，然后翻耕入土，可有效杀死土壤中的蛴螬。在大蒜生长期间，若发现蛴螬为害，可用辛硫磷、高效氯氰菊酯等药剂灌根，将药剂稀释成一定浓度的溶液，浇在大蒜根部周围的土壤中，以毒杀蛴螬。
生物防治：保护和利用蛴螬的天敌，如土蜂、食虫虻、鸟类等，通过生物间的相互制约来控制蛴螬的种群数量。也可以使用球孢白僵菌、绿僵菌等微生物制剂进行防治，将制剂与细土混合后撒在田间，使蛴螬感染病菌而死亡。
地老虎基本信息：地老虎是鳞翅目夜蛾科切根夜蛾亚科昆虫的通称，又名土蚕、切根虫等。常见的有小地老虎、大地老虎和黄地老虎等，在世界各地均有分布，在我国大部分地区的大蒜种植区都有发生。
地老虎危害：咬断幼苗：地老虎以幼虫为害大蒜，低龄幼虫常聚集在大蒜幼苗的心叶或嫩叶上取食，将叶片咬成小孔洞或缺刻。3 龄以后的幼虫则会咬断大蒜幼苗的茎基部，使植株死亡，造成缺苗断垄，严重影响大蒜的产量。取食鳞茎：大龄幼虫还可能钻入大蒜的鳞茎中取食，导致鳞茎腐烂，降低大蒜的品质和商品价值。
地老虎体型特征：幼虫：地老虎幼虫身体呈圆筒形，体表粗糙，多为暗褐色或黑色，有光泽。头部坚硬，呈黄褐至黑褐色。身体各节有大小不等的颗粒状突起，腹部末节背板上有两条深褐色纵带。成虫：地老虎成虫为中型蛾类，体长 16-23 毫米，翅展 42-54 毫米。前翅暗褐色，上有肾形纹、环形纹和剑形纹等斑纹，后翅灰白色，边缘呈淡褐色。
地老虎生存习惯：栖息环境：地老虎幼虫白天潜伏在土中，夜晚出土活动取食，具有较强的趋光性和趋化性。成虫喜欢栖息在杂草、土块、枯枝落叶等隐蔽场所。
食性特点：地老虎是多食性害虫，除为害大蒜外，还可为害多种蔬菜、粮食作物及杂草。幼虫以植物的幼苗、嫩叶、茎基部等为食，成虫则吸食花蜜等补充营养。
繁殖规律：地老虎一年发生多代，以蛹或老熟幼虫在土中越冬。成虫在春季羽化，产卵于杂草或土块上。卵孵化后，幼虫经过 5-6 龄发育成熟，老熟幼虫在土中化蛹。
地老虎预防措施：农业防治：及时清除田间及周边杂草，减少地老虎的产卵场所和幼虫的食物来源。秋季深翻土地，将越冬的蛹和幼虫翻至地表，使其被冻死、晒死或被天敌捕食。
物理防治：利用地老虎成虫的趋光性，在田间设置黑光灯或频振式杀虫灯诱杀成虫。也可在田间放置糖醋液诱捕器，糖醋液的配方为糖：醋：酒：水=6：3：1：10，并加入少量敌百虫等杀虫剂，诱杀成虫。
地老虎化学防治：在大蒜播种前，可用辛硫磷、毒死蜱等药剂进行土壤处理。在幼虫发生期，可选用高效氯氰菊酯、甲维盐等药剂进行喷雾防治，重点喷施在大蒜幼苗的基部和周围土壤。也可将药剂与麦麸、豆饼等饵料混合制成毒饵，于傍晚撒在田间，诱杀幼虫。
地老虎生物防治：保护和利用地老虎的天敌，如寄生蜂、寄生蝇、鸟类等。也可使用苏云金芽孢杆菌等生物制剂进行防治，对幼虫有较好的杀灭效果。
韭菜迟眼蕈蚊基本信息：韭菜迟眼蕈蚊，属双翅目眼蕈蚊科，别名韭蛆，是一种重要的蔬菜害虫。在我国大部分地区均有分布，主要为害韭菜、大蒜、洋葱等百合科蔬菜，在大蒜产区发生较为普遍。

韭菜迟眼蕈蚊危害：幼虫为害：幼虫聚集在大蒜的鳞茎周围，蛀食鳞茎，使鳞茎腐烂，地上部分叶片发黄、萎蔫，生长势减弱，严重时整株死亡，导致大蒜产量大幅下降。

传播病菌：幼虫在为害过程中造成的伤口，有利于病菌的侵入，从而引发大蒜的软腐病等病害，进一步降低大蒜的品质和产量。

韭菜迟眼蕈蚊体型特征：成虫：体长 2-5 毫米，黑褐色，头部小，复眼大而突出，触角丝状，16 节。前翅透明，翅脉简单，足细长，腹部细长，8-9 节。幼虫：老熟幼虫体长 7-10 毫米，头部漆黑色，有光泽，体细长，呈乳白色，无足。

韭菜迟眼蕈蚊生存习惯：栖息环境：成虫喜欢在阴暗潮湿的环境中活动，常栖息在大蒜植株基部、土缝等隐蔽处。幼虫生活在土壤中，主要为害大蒜的地下部分。食性特点：以大蒜等百合科蔬菜的鳞茎、根为食，尤其喜食腐烂的组织。繁殖规律：在北方地区，一年发生 4-6 代，以幼虫在土壤中或蒜根周围越冬。翌年春天，当气温回升到 10℃左右时，幼虫开始活动为害，成虫羽化后在大蒜植株附近的土壤表面或植株上产卵，卵期一般为 3-5 天。幼虫孵化后即开始蛀食大蒜。

韭菜迟眼蕈蚊预防措施：农业防治：合理轮作，避免与韭菜等寄主植物连作。收获后及时清理田间病残体，深翻土地，将幼虫翻至地表，使其冻死或被天敌捕食。

物理防治：在成虫发生期，利用成虫的趋光性，在田间设置黑光灯或频振式杀虫灯诱杀成虫。也可在田间悬挂糖醋液诱捕器，糖醋液配方为糖：醋：酒：水=3：3：1：10，加入少量敌百虫等杀虫剂，诱杀成虫。

韭菜迟眼蕈蚊化学防治：在大蒜播种前，可用辛硫磷、毒死蜱等药剂进行土壤处理。在幼虫发生期，可用高效氯氰菊酯、吡虫啉等药剂灌根，将药剂稀释成一定浓度的溶液，浇在大蒜根部周围的土壤中，以毒杀幼虫。

韭菜迟眼蕈蚊生物防治：可使用球孢白僵菌、绿僵菌等微生物制剂进行防治，将制剂与细土混合后撒在田间，使幼虫感染病菌而死亡。也可释放捕食性天敌，如小花蝽等，以控制韭菜迟眼蕈蚊的种群数量。

斜纹夜蛾基本信息：斜纹夜蛾属鳞翅目夜蛾科，是一种世界性分布的多食性害虫，在我国各地均有发生。它寄主范围广，能为害多种蔬菜和农作物，包括大蒜。

斜纹夜蛾危害：叶片受损：低龄幼虫群集在大蒜叶片背面取食叶肉，使叶片呈现白纱状斑。大龄幼虫食量增大，会将叶片咬成缺刻、孔洞，严重时可将叶片吃光，仅留叶脉和叶柄，影响大蒜的光合作用，导致大蒜生长受阻，蒜薹和蒜头产量降低。传播病菌：其取食造成的伤口易使大蒜感染病菌，引发软腐病等病害，进一步降低大蒜的品质和产量。

斜纹夜蛾体型特征：成虫：体长 14-20 毫米，翅展 35-40 毫米。前翅灰褐色，内横线和外横线灰白色，呈波浪形，中间有明显的白色斜纹带，后翅白色，外缘暗褐色。幼虫：老熟幼虫体长 35-47 毫米，头部黑褐色，体色变化较大，有土黄色、青黄色、灰褐色等，体背有灰色和橙色相间的条纹，各体节亚背线内侧有近半月形或三角形黑斑。

斜纹夜蛾生存习惯：栖息环境：成虫昼伏夜出，白天隐藏在杂草、土缝等阴暗潮湿处，夜间出来活动、取食和交配。幼虫有假死性，老熟后入土化蛹。食性特点：幼虫食性杂，喜食大蒜等多种植物的叶片、花蕾等。初孵幼虫有群集性，3 龄后分散为害，4 龄后进入暴食期。繁殖规律：在我国南方一年可发生多代，北方发生 4-5 代。成虫产卵于叶片背面，卵块上覆盖有黄褐色绒毛。卵期一般为 2-3 天，幼虫期 15-20 天。

斜纹夜蛾预防措施：农业防治：及时清除田间及周边杂草，减少斜纹夜蛾的滋生地。人工摘除卵块和初孵幼虫群集的叶片，集中销毁。

物理防治：利用成虫的趋光性，在田间安装黑光灯或频振式杀虫灯诱杀成虫。也可在田间放置糖醋液诱捕器，糖醋液按糖：醋：酒：水=3：4：1：2 的比例配制，并加入少量敌百虫等杀虫剂，诱杀成虫。

斜纹夜蛾化学防治：在幼虫低龄期，选用高效氯氟氰菊酯、甲氨基阿维菌素苯甲酸盐等药剂进行喷雾防治，重点喷施在叶片背面和心叶部位。由于斜纹夜蛾幼虫对药剂易产生抗药性，应注意不同药剂的交替使用。

斜纹夜蛾生物防治：保护和利用斜纹夜蛾的天敌，如赤眼蜂、小茧蜂等。也可使用核型多角体病毒、苏云金芽孢杆菌等生物制剂进行防治，对幼虫有较好的控制效果。

金针虫基本信息：金针虫是鞘翅目叩甲科幼虫的统称，常见的有沟金针虫、细胸金针虫等。金针虫在我国分布广泛，是重要的地下害虫，能为害多种农作物，包括大蒜。

金针虫危害：金针虫主要以幼虫在土壤中咬食大蒜的根系、鳞茎等地下部分。它会将根系咬断，使大蒜植株生长不良，叶片发黄、枯萎。还会蛀食鳞茎，导致鳞茎腐烂，影响大蒜的产量和品质，严重时会造成大蒜缺苗断垄。

金针虫体型特征：金针虫幼虫细长，体壁坚硬，多为黄色或黄褐色，具有光泽。其身体呈圆柱形，略扁，体表有纵沟和毛瘤。沟金针虫幼虫体长约 20-30 毫米，最宽处约 4 毫米，体节宽大于长，从头至第九腹节渐宽。细胸金针虫幼虫体长约 23 毫米，宽约 1.3 毫米，体细长，圆筒形，淡黄褐色，有光泽。

金针虫生存习惯：金针虫长期生活在土壤中，一般在土深 20-30 厘米处活动。其活动与土壤温度、湿度关系密切，适宜的土壤湿度为 15%-20%。它是杂食性害虫，除为害大蒜外，还取食多种作物的种子、幼苗及根茎。金针虫的生活史较长，一般 2-5 年完成一代，以成虫或幼虫在土壤中越冬。春季气温回升时，幼虫向上移动至表土层为害，秋季气温下降后又下移至深层土壤。

金针虫预防措施：农业防治：实行轮作倒茬，避免连作，可与水稻等非寄主作物轮作。秋季深翻土地，将幼虫翻至地表，使其受天敌捕食和低温影响而死亡。

金针虫物理防治：在田间设置黑光灯诱杀成虫，减少虫口密度。

金针虫化学防治：播种前用辛硫磷、毒死蜱等药剂进行土壤处理，可将药剂与细土混合制成毒土，撒在播种沟内。也可用药剂拌种，常用药剂有吡虫啉等，能有效防治金针虫为害。

金针虫生物防治：可使用绿僵菌、白僵菌等微生物制剂进行防治，将制剂施入土壤中，使金针虫感染病菌而死亡。同时，保护和利用金针虫的天敌，如步甲、食虫虻等，以控制其种群数量。

杂草对大蒜的影响：竞争养分：杂草与大蒜争夺土壤中的氮、磷、钾等养分。例如，稗草、马唐等一年生杂草生长迅速，会大量吸收土壤养分，导致大蒜可获取的养分减少，生长缓慢，蒜薹细小，蒜头不饱满。

争夺水分：杂草的根系发达，会与大蒜竞争土壤中的水分。在干旱时期，狗尾草等杂草会使大蒜因缺水而出现叶片发黄、卷曲等现象，影响光合作用和正常生长发育。争夺光照：一些高大杂草如藜、苋等，会遮挡大蒜的阳光，使大蒜不能充分进行光合作用，植株变得细弱，抗逆性降低。

杂草传播病虫害：杂草是许多病虫害的中间寄主和越冬场所。例如，刺儿菜等杂草容易滋生蚜虫，而蚜虫会传播大蒜花叶病毒，导致大蒜出现花叶、畸形等症状，影响产量和品质。

杂草预防改进措施：农业防治：合理密植，增强大蒜植株的群体优势，抑制杂草生长。在大蒜种植前，通过深耕、晒垡等措施，杀灭土壤中的杂草种子和根茎。及时中耕除草，既能去除杂草，又能疏松土壤，促进大蒜根系生长。

物理防治：在大蒜田铺设黑色地膜，可有效阻挡杂草见光，抑制杂草生长。同时，人工拔除田间残留杂草，尤其是在大蒜生长前期，及时拔除杂草能减少其对大蒜的影响。

杂草化学防治：在大蒜播种后出苗前，可选用乙草胺、扑草净等芽前除草剂进行土壤封闭处理，抑制杂草种子萌发。在大蒜生长期，针对不同类型的杂草，选择合适的除草剂进行定向喷雾，如以禾本科杂草为主的田块，可使用精喹禾灵等药剂。

杂草生物防治：利用昆虫、动物或微生物来控制杂草。例如，在大蒜田释放以杂草为食的昆虫，如叶甲等，可有效控制杂草数量。还可使用一些微生物除草剂，如鲁保一号可防治菟丝子。
